# Estimation and efficient computation of the true probability of recurrence of short linear protein sequence motifs in unrelated proteins

**DOI:** 10.1186/1471-2105-11-14

**Published:** 2010-01-07

**Authors:** Norman E Davey, Richard J Edwards, Denis C Shields

**Affiliations:** 1 UCD Complex and Adaptive Systems Laboratory, University College Dublin, Dublin, Ireland; 2 UCD Conway Institute of Biomolecular and Biomedical Research, University College Dublin, Dublin, Ireland; 3 UCD School of Medicine and Medical Sciences, University College Dublin, Dublin, Ireland; 4 EMBL Structural and Computational Biology Unit, Meyerhofstrasse 1, 69117 Heidelberg, Germany; 5 School of Biological Sciences, University of Southampton, Southampton, UK

## Abstract

**Background:**

Large datasets of protein interactions provide a rich resource for the discovery of Short Linear Motifs (SLiMs) that recur in unrelated proteins. However, existing methods for estimating the probability of motif recurrence may be biased by the size and composition of the search dataset, such that *p-*value estimates from different datasets, or from motifs containing different numbers of non-wildcard positions, are not strictly comparable. Here, we develop more exact methods and explore the potential biases of computationally efficient approximations.

**Results:**

A widely used heuristic for the calculation of motif over-representation approximates motif probability by assuming that all proteins have the same length and composition. We introduce *p*_*v*_, which calculates the probability exactly. Secondly, the recently introduced SLiMFinder statistic *Sig*, accounts for multiple testing (across all possible motifs) in motif discovery. However, it approximates the probability of all other possible motifs, occurring with a score of *p *or less, as being equal to *p*. Here, we show that the exhaustive calculation of the probability of all possible motif occurrences that are as rare or rarer than the motif of interest, *Sig'*, may be carried out efficiently by grouping motifs of a common probability (i.e. those which have permuted orders of the same residues). *Sig'*_*v*_, which corrects both approximations, is shown to be uniformly distributed in a random dataset when searching for non-ambiguous motifs, indicating that it is a robust significance measure.

**Conclusions:**

A method is presented to compute exactly the true probability of a non-ambiguous short protein sequence motif, and the utility of an approximate approach for novel motif discovery across a large number of datasets is demonstrated.

## Background

Short Linear Motifs (SLiMs) are abundant and ubiquitous recurring protein subsequences that function largely independently of the tertiary structure of the containing protein. SLiMs mediate tasks such as cell signalling, act as recognition sites for protein modification and target proteins for subcellular localisation [1,2]. SLiMs are short (typically between three and ten amino acids in length) and degenerate (positions are often flexible in terms of possible amino acids) making motif context important for specificity due to the limited number of residues in the interaction interface [3]. This simplicity gives them an evolutionary plasticity that is unavailable to globular protein domains, enabling *de novo *motifs to evolve convergently through point mutations, thereby adding new functions to proteins [4]. SLiMs preferentially occur in natively disordered regions of proteins [5] challenging the long standing dogma that structure equals function [6]. With 25% - 30% of the human proteome natively disordered and large portions of the interactome unexplained by known domain-domain interactions [7] there is vast potential for novel SLiM discovery [8].

Increased knowledge of SLiM attributes, through the study of known functional motifs, has enabled advancements in computational methods for SLiM discovery. This culminated in the discovery of a Translin-binding motif, the first novel functional motif to be discovered by computational methods [9]. These methods can be split into two distinct classes; rediscovery of known SLiMs and prediction of novel SLiMs. SLiM rediscovery, which was pioneered by PROSITE [10], uses regular expression or profile matching to search for novel instances of previously known SLIMs. Tools such as the ELM [1] and MnM [11] servers use context information such as globularity, conservation or residue accessibility to improve confidence in returned motifs. Motif rediscovery techniques have also been used to search for enrichment of known SLiMs in proteins that are associated with particular protein functions, using statistical enrichment to define motifs important for these functions. These methods have been used to discover novel instance of both KEN box and EH1 transcriptional repressor motifs [12,13].

Several methods have also been suggested for the discovery of novel SLiMs. The most successful of these methods seek to find motifs that are over-represented in evolutionarily unrelated proteins sharing a common attribute (such as interacting with a common protein-binding domain [14], those which have a common post-translational modification [15] or which localise to the same sub-cellular location [16]). Dilimot [17] and SLiMDisc [18] use this technique, to build upon the scoring schemes of ASSET [19] and PRATT [20] respectively, to successfully discover new, and rediscover known, functional motifs. However, these first generation methods have two important deficiencies: (a) their scores are biased on motif length and dataset size, making them incomparable across multiple datasets and between motifs of different lengths; and (b) they do not offer an indication of the likelihood that any motif could reach such a score by chance.

In previous work, we introduced SLiMFinder [21], a probabilistic method for SLiM discovery that heuristically accounts for these shortcomings with a two-step scoring scheme. Both steps of this scoring scheme use simplifying assumptions that are known to be violated in real data. Given that motif probability calculations are relatively approximate (it is difficult to model exactly the amino acid or word count background distributions in a set of proteins or protein regions under study) these approximations seem at first glance to be satisfactory. However, in practise we noted in studies searching for motifs across many datasets that consistent biases were emerging, that related to the complexity of the motif, and to the size of the dataset searched. Since computational motif discovery is most usually struggling to identify a weak signal against a background of noise, we were motivated to try and eliminate these biases by improving the statistical calculations.

The first step of the SLiMFinder scoring scheme applies the binomial probability mass function to mean success probabilities across all proteins, denoted here as p_*μ*_, to calculate the probability of a given motif occurring by chance in the number of proteins it occurs in, or greater. Therefore, this assumes that the proteins in a search dataset all have the same length and composition. Here, we introduce a more accurate calculation, *p*_*v *_which incorporates unique success probabilities for each protein, allowing for its length and composition. The advantage of correcting for this effect is that the p-value cut-offs from datasets that have varying sizes of proteins become more comparable, as well as being more realistic.

The second step of the SLiMFinder scoring scheme computes an approximate significance, *Sig*. This is a dataset-based score, which accounts for the multiple testing inherent in motif discovery, by calculating the probability that any motif in the dataset will be returned with that p-value or less by chance. However, *Sig *approximates the probability of all other possible motifs occurring with a score of *p *or less as *p*. Here, we introduce the true calculation, *Sig'*, that replaces this approximation, and present an efficient algorithm for its calculation. The advantage of correcting for this effect is that motifs of different length, or containing amino acids of very different frequency, are treated equally, so that there is not a bias towards any class of motif.

We investigate the impact of replacing both approximations with the more exact calculations on the overall accuracy of the scoring scheme. Each step is independent allowing them to be implemented and tested separately, four significance scoring schemes are discussed (Table 1), investigating the trade-off between accuracy and efficiency. We discuss the optimal approaches for large-scale studies of multiple datasets.

**Table 1:** The four scoring schemes investigated in this study.

	*p*_*μ*_	*p*_*v*_
**Approximate probability of all possible motifs**	*Sig *(Eq. 3 & 6)	*Sig*_*v *_(Eq. 5 & 6)

**Exact probability of all possible motifs**	*Sig' *(Eq. 3 & 8)	*Sig'*_*v *_(Eq, 5 & 8)

## Methods

Motif discovery statistics can be defined by 3 basic probabilities; *p*_1+_, the probability that there are *1 or more *occurrences of a motif in a protein; *p*, the probability in a dataset of *N *proteins that a given motif would occur by chance with its observed support or higher; and *Sig*, the probability that any motif would reach p or less by chance. In this section, each probability will be introduced and discussed, focussing on the speed and accuracy of the statistics, and an efficient and accurate method of calculating *Sig *is described.

### Exact probability of a given motif support considering unique success probabilities

#### Previous work: Mean Success Probability Heuristic

The probability, *p*_1+_, that there are one or more occurrences of a motif in a protein is calculated differently by SLiMFinder and Dilimot. The former calculates *p*_1+ _based on amino acid frequencies, whereas the later calculates *p*_1+ _based on word counts. The merits of each method have been discussed previously [21]. Here, the calculation of *p*_1+_, is based on amino acid frequencies and allows ambiguous/degenerate positions to be evaluated.

where

##### Equation 1. p(1 or more instances of a given motif in a given protein)

where the success probability, *p*_*motif*_, is the probability that the motif will occur at any position in the protein, *n *is the number of positions in the protein that the motif can occur, *m *is the motif, *l *is the number of non-wildcard positions in the motif, *m*_*i *_is position *i *in motif *m*, *x *is the number of degenerate/ambiguous possibilities at position *i*, *m*_*ik *_is the *k*^*th *^ambiguous possibility at position *i*, *f*(*m*_*ik*_) is the background frequency of the amino acid *m*_*ik *_and *B *is the binomial function. The calculation is independent of alphabet and allows the use of terminal characters "^" and "$" denoting N- and C- termini respectively, which is taken into account in calculating motif significance.

Equation 1 considers both amino acid composition and the length of the protein to calculate the probability. By considering the length (the variable *n *accounts for the number of positions in a protein at which a motif can occur) the calculations allow short proteins/peptides (for example peptides returned by phage display screens) to be scored without bias. The amino acid composition allows for any background amino acid frequency to be incorporated, including the distribution from within a protein, adding flexibility to the scoring scheme.

Generally, each protein (or cluster of proteins whenever several homologous proteins are grouped or weighted to correct dependencies introduced by divergent evolution [22]) will have a unique success probability, *p*_1+_. Previously, the mean success probability for the motif to occur in any given protein in the dataset, *p*_1+*μ*_, was approximated as the mean of the *p*_1+ _values.

##### Equation 2. Approximate p (1 or more instances of a given motif in any given protein)

The probability of *k or more *proteins in a dataset containing a given motif one or more times, *p*_*μ*_, may be estimated using *I*, the incomplete beta function, the cumulative distribution function of the binomial probability mass function.

##### Equation 3. Approximate p(k or more proteins with 1 or more occurrences of a given motif)

where *k *is the support of the motif (i.e. the number of proteins containing it), *N *is the number of proteins in the dataset and *p*_1+*μ *_is the mean success probability of a motif occurring in any protein in the dataset.

#### More exact calculation

The binomial function calculation in Equation 3, assumes all success probabilities are equal to *p*_1+*μ *_(Equation 2). The binomial equation can be expanded to allow unique success probabilities for each protein, *p*_*v*_, by summing the product of success and failure probabilities for each possible combination of *k *successes, *t*, and *N-k *failure probabilities, *f*. This computation increases the number of calculations necessary ^*N*^*C*_*k *_fold.

##### Equation 4. Set of all possible combinations of k successes from N trials

where

##### Equation 5. Exact p(k or more proteins with 1 or more occurrences of a given motif)

#### Example

The error associated with *p*_*μ *_is most easily explained in terms of occasions where *p*_1+ _for a protein is 0, a frequent occurrence when masking techniques are used to improve motif discovery (e.g., a protein entirely lacking W has zero probability of having the motif WxxS). Consider a set of 6 proteins, with success probabilities for a motif *p*_1+ _of {0.1, 0.1, 0.1, 0.0, 0.0, 0.0}. 3 proteins contain an occurrence of the motif. The mean success probability *p*_1+ _is 0.05 and the motif has a *p*_*μ *_of 1.64^e-3^. When unique success probabilities are used a *p*_*v *_of 7.29^e-4 ^is calculated. In this case, the motif occurring *k *times or more is roughly twice as unlikely to occur as approximated by *p*_*μ*_. The bias introduced by *p*_*μ *_can therefore be considered conservative estimate.

### Exact probability that any motif will be returned with a given *p*-value or less

#### Previous work: Motif Binomial p-value Significance Heuristic

SLiMFinder introduced *Sig*, the probability of any motif, with the same number of non-wildcard positions, occurring with that probability *p *or less by chance.

##### Equation 6. Approximate probability (any motif will occur with a binomial p-value of p or less)

where R is calculated as *20*^*l*^*(x+1)*^*l*-1^, *l *is the number of non-wildcard positions in the motif and *x *is the maximum length of a wildcard region allowed. Effectively, this score assumes that all other possible motifs are equally likely to give rise to a score equal to, or lower than, the motif of interest. However, this is not always true. For example, more likely motifs may have, for all supports, a p-value greater than the observed motif's p-value, hence such motifs may never actually occur with a probability as low as *p*. *Sig *will always score a given *p *as being as likely or more likely than it is, making *Sig *a conservative approximation.

#### More Accurate Significance

To calculate *Sig'*, the true probability of 1 or more fixed position motifs (i.e. with the same number of non-wildcard positions) in a dataset occurring with the observed *p *or lower, it is necessary to calculate, for each possible motif *p'*, the probability that the motif will occur with a score of *p *or less. An efficient algorithm for the calculation of *Sig' *is available in the Appendix.

where

##### Equation 7. probability (any given motif will occur with a p-value less than or equal to the p of the observed motif)

##### Equation 8. probability (any motif will occur with a p-value of p or less)

#### Example

Consider a search for dimers in a dataset with an alphabet, A = {L, W}, of size a = 2 and proteins of length 5. For the purposes of simplicity, the example uses global amino acid frequencies for p_1+ _calculations, whereas the typical case described earlier in the manuscript has individual p_1+ _probabilities for each protein. The dataset contains 4 proteins (N = 4) with sequences (LLLLL, LLLLL, LWWLL, LLLLL) giving global amino acid frequencies of *f*_*L *_= 0.9 and *f*_*W *_= 0.1. The probability that any of the 4 possible dimer motifs in the motif space, M, (M = {LL, WW, LW, WL}, *S *= 4) will occur at any position in a protein are *p*_*LL *_= 0.81, *p*_*WW *_= 0.01 *p*_*WL *_= *p*_*LW *_= 0.09 (see Equation 1, *p*_*motif*_). The probability that a protein will contain one or more occurrences of a motif is *p*_1+ _(LL) = 0.9987, *p*_1+ _(WW) = 0.0394, *p*_1+ _(WL) = *p*_1+ _(LW) = 0.3143 (see Equation 1). Using the binomial and cumulative binomial we can calculate, using *p*_1+ _as the mean success probability (all protein sequences are the same length and use the same global amino acid frequencies so the success probabilities for each protein for a given motif will be the same), the probability that each motif will occur *k *times (Binomial in Table 2) and the probability that each motif will occur *k *or more times (*p*_*μ *_in Table 2).

**Table 2:** Cumulative binomial and binomial p-values for motif example described in Methods

Motif (k)	p_*μ*_					*B*(*k*, *N*, *p*1 +_*motif*_)				
k	0	1	2	3	4	0	1	2	3	4
**LL(4)**	1	1	1	1	0.9948	0	0	0.0001	0.005	0.9948
**WW(1)**	1	***0.1485***	**0.0088**	**0.0002**	**0**	0.8515	**0.1397**	**0.0086**	**0.0002**	**0**
**WL(1)**	1	0.7789	0.3736	**0.0949**	**0.0098**	0.2211	0.4053	0.2787	**0.0852**	**0.00948**
**LW(1)**	1	0.7789	0.3736	**0.0949**	**0.0098**	0.2211	0.4053	0.2787	**0.0852**	**0.00948**

Incomplete beta and binomial shows the cumulative binomial and binomial *p*-values respectively for each motif for all supports between 0 and N = 4. p_1+*μ *_is the success probability of the motif considered. The value in italics indicates the highest scoring motif in the example described. The bold values indicate the values of *k *for which *I(k, N, pm) *< = 0.1485, the *p*_*μ *_value of the highest ranking motif. The five right hand columns are only shown to illustrate how the probabilities in the left hand columns are calculated (with sums across the bold values in the right hand columns cells totaling the values in the left hand columns).

Searching the dataset for over-represented motifs, the support *k *for the motifs are LL = 4, WW = 1, WL = 1 and LW = 1. *p*_*μ *_of each motif in the motif space is *p*_*μ LL *_= 0.9948, *p*_*μ WW *_= 0.1485, *p*_*μ LW *_= *p*_*μ WL *_= 0.7789 (Equation 3), making WW the top ranking motif occurring in 1 protein with a probability, *p*, of 0.1485. From Equation 6, the significance, *Sig*, of *p*_*μ WW *_is 0.474. For the example in Table 2, the set of support values (*S*_*motif*_) are shown in bold. This gives the values of k for which I(*k, N, p*_*m*_) < = *p*, as being *S*_*LL *_= {}, *S*_*WW *_= {1,2,3,4} and *S*_*WL *_= *S*_*LW *_= {3,4}, therefore *p'*_*LL *_= 0, *p'*_*WW *_= 0.1485 and *p'*_*WL *_= *p'*_*LW *_= 0.0949 (see Equation 7). As expected, the p' values are all less than or equal to *p*, illustrating the conservative nature of the *Sig *score. Calculating Sig', once the individual *p' *values are known, is straightforward. The *Sig' *for WW is 1 - (1 - 0)(1-0.0949)(1-0.0949)(1-0.1485) = 0.302 (Equation 8).

### Randomised test datasets

To test each of the 4 significance scoring schemes, 3 sets of 100 datasets containing 10, 30 and 60 proteins were picked at random from the UNIPROT database [22]. These datasets model random interaction networks that are likely to have little or no enrichment for any functional motifs. To allow for the calculation of *Sig'*_*v*_, a process that is currently computationally intractable using the calculations described here, a similar set of proteins was created to artificially force *p*_1+ _values to be equal for each protein thereby testing the scoring schemes accuracy when no bias is present. A human protein was chosen at random from the UNIPROT database and the residues where shuffled to create datasets of 10, 30 and 60 proteins, therefore each protein is same length and has the same amino acid frequency and hence, for any given motif, has the same *p*_1+ _for each protein.

Datasets were analysed using SLiMFinder with default settings with the exception of statistical calculations that were completed as described in this paper. The statistics described in this paper are implemented in freely downloadable software, as a modification to version 4.0 of the previously described SLiMFinder software [21], with additional command line switches introduced for the two calculations (sigprime = T/F and sigv = T/F).

The programme is available at http://bioinformatics.ucd.ie/shields/software/slimfinder. The version of the SLiMSuite software package available at time of submission is also included as supplemental information with this paper (Additional File 1).

## Results and Discussion

### Comparing the four statistical scoring schemes to expectation

When tested on randomly created datasets, significance scores for fixed motifs should ideally be uniformly distributed [23] (i.e. if 100 datasets are analysed we would expect to see 1 motif with a significance of 0.01 or less, and 50% of the datasets should have a significance of 0.5 or greater). Using datasets of proteins randomly selected from UniProt, we tested to what extent each of the 4 scoring schemes rejected the null hypothesis, that the top ranking significance scores from these random datasets are uniformly distributed.

**Figure 1:**
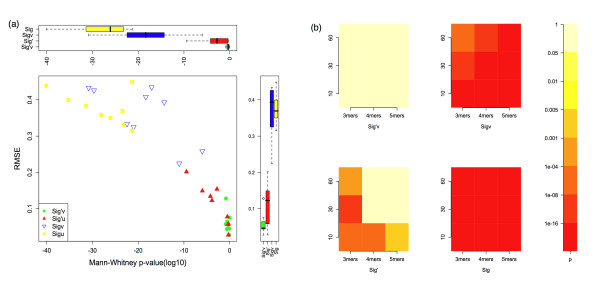
**Comparing the distribution of returned top ranking fixed position motifs to the uniform distribution for the 4 tested significance scoring methods**. (a) Scatterplot of the root mean square error (RMSE) for the distribution of returned top ranking motifs for each dataset size and motif length (nine combinations in total) from the uniform distribution versus the *p*-value of a Mann-Whitney test for rejection of the hypothesis that the distribution of top ranking motifs significance values were sampled from the uniform distribution. The top boxplot describes the *p*-value of a Mann-Whitney test and the boxplot on the right describes the RMSE data. (b) Comparison of 4 tested significance scoring methods for probability of being sampled from the uniform distribution. The heatmap plots, for each dataset size (horizontal axis) and motif length (vertical axis), the *p*-value of a Mann-Whitney test for rejection of the hypothesis that the distribution of top ranking motifs significance values was sampled from the uniform distribution.

For all motif lengths and dataset sizes tested, *Sig'*_*v *_did not reject the null hypothesis, consistent with it being a true significance measure (Fig. 1a, top panel). The other three statistics all reject the null hypothesis to differing degrees, with *Sig' *showing less departure from expectation compared to *Sig and Sig*_*v*_. *Sig *rejects the null hypothesis for all the datasets. The statistics showed similar effects when considering the divergence of the observed *p*-values (root mean squared error) from the uniform distribution (Fig. 1a, side panel). The degree of rejection of the null hypothesis varies considerably according to the dataset size and the motif length (Fig 1a, main panel). A heatmap illustrates these effects (Fig 1b), showing that the degree of rejection of the null hypothesis for each of the three approximate statistics depends on both the dataset size, and on the length of the motif under consideration. Overall, the greatest improvement over the *Sig *statistic is provided by considering the exact probability that any motif may have a *p*-value of the observed significance or less (implemented in *Sig' *and in *Sig'*_*v*_); while the correction for varying protein lengths and composition in a dataset has a lesser effect when calculating the p-value of the observed motif (implemented in *Sig*_*v *_and in *Sig'*_*v*_).

### How comparable are significance values of motifs of differing length, or motifs discovered in different sized datasets?

One of the major biases with using a binomial p-value, *p*_*μ*_, which SLiMFinder attempted to improve upon, was the incomparability of *p*-values for motifs of different lengths. *p*_*μ *_is strikingly different for random datasets, depending on whether it is the value for a 3mer or a 5mer (SF2 in Additional File 2). We were interested to discover to what extent a given motif significance score might be comparable for different discovered motifs, and to what extent the score is biased, depending on dataset size and motif length. For each scoring scheme, we compared the returned significance scores obtained with each motif size in each dataset size.

**Figure 2:**
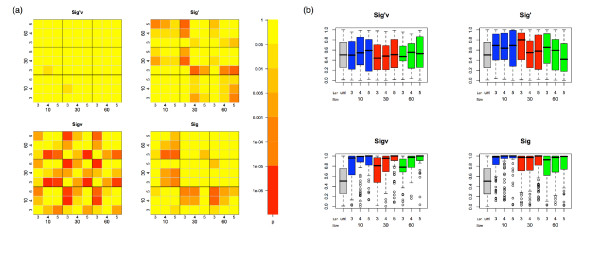
**Test for the comparability of the significance scores, for the 4 tested significance scoring methods, between fixed position motifs of different length and datasets of different size**. (a) Boxplot comparisons of the 4 tested significance scoring schemes, for each dataset size and motif length, of the distribution of top ranking motifs significance values. The first boxplot in each panel is the uniform distribution. (b) The heatmap describes the Mann-Whitney *p*-value for all-by-all comparisons of the 3 dataset sizes and 3 motifs lengths. The Mann-Whitney, in this case, tests for rejection of the hypothesis that the distributions of *Sig *values of top ranking motifs of different length and dataset size are sampled from the same distribution.

No biases are evident with the *Sig'*_*v *_scoring scheme where the hypothesis that the significance scores were sampled from the same distribution was only rejected (p < 0.05) for one of the 36 comparisons (Fig. 2b), consistent with expectations. The *Sig *statistic showed some biases, but somewhat surprisingly, the *Sig*_*v *_statistic appeared more biased than the *Sig *statistic (Fig. 2b): in particular, the larger datasets seemed to be biased in a way that the smaller datasets are not (Fig 2a). However, It can be seen that the majority of *Sig *correlation between datasets relates to p-values in the range of 0.8 to 1.0 (Fig 2a) and is a related to the schemes conservative bias. The box-plots in Fig 2a indicate that *Sig' *is reasonably similarly distributed for all motif lengths and dataset sizes, but it is noticeable that the mean *p*-value varies more across datasets and motif sizes than *Sig'*_*v *_does. What is not clear from these random datasets is the extent to which these two statistics are biased for motifs that are significantly over-enriched (i.e. in ranking true positives, at the other end of the p-value scale).

### Ambiguity

SLiMFinder considers subsets of pre-defined residue groupings to allow ambiguity in motif positions; this further adds to the complications of significance calculations and the probability calculations have not been extended to consider these. In general, the true significance calculations described in this paper, when used to calculate the significance of an ambiguous motif, will over-estimate the significance of the motif. In SF3 in Additional File 2, this is illustrated. For these examples, ambiguous motifs were defined as those with one or more ambiguous position or with at least one variable length spacer, using the default set of ambiguous groupings (KRH, DE, ILMV, FYWH, AGS, ST). *Sig'*_*v *_overestimates the significance, ignoring the greatly increased search space of potential ambiguous motifs, in addition to the search space of fixed-position motifs (SF3 in Additional File 2). We note that under the conditions tested, the *Sig *calculation is also conservative in all of the datasets considered here, even in the presence of ambiguity. Thus, for ambiguous motifs the *Sig *statistic may provide a more robust heuristic for very roughly approximating absolute significance. However, it must be noted that in general ambiguous motifs will still have a relatively higher *Sig *score than non-ambiguous motifs, so that a single model for ranking of ambiguous and non-ambiguous motifs is not ideal. Accordingly, search results within a dataset or across many datasets for ambiguous and non-ambiguous motifs should be considered as two separate rankings, given the current available models.

### Calculation redundancy

The level of redundancy in the brute force calculation of *Sig' *compared to the use of non-redundant motif groupings (Appendix, Equations 9-13) is provide in Table 3. For 5mer motifs there is a 75-fold decrease in the number of calculations necessary for the calculation of the same value. Since the level of redundancy increases as the length of the motif increases, true *Sig' *calculations are possible for motifs which would been computationally infeasible using brute force calculations.

**Table 3:** Redundancy of motif probabilities (see Equations 9-13).

Motif length	Number of partitions	Number of motifs	Number of non-redundant motifs	Proportion of non-redundant motifs
3	3	8000	1540	19.25%

4	5	160000	8855	5.53%

6	11	64000000	177100	0.27%

*Motif length *is the length of the motif. *Number of partitions *is the number of solutions in the set *M *for motifs of length *n. Number of motifs *is the number of distinct motifs of length *l *where the order of the residues is important. *Number of non-redundant motifs *is the number of the non-redundant distinct motifs of length *l *where the order of the residues is not important. *Proportion of non-redundant motifs *is the percentage of motif calculations needed to calculate the true *p*-value *Sig' *distribution.

### Computational efficiency

For each motif length considered, the number of calculations necessary to calculate *Sig'*, with the algorithmic speedup described (Appendix, Equations 9-13), increases the number of calculations ^a+l-1^C_l _fold, compared to the *Sig *calculation, where *a *is the alphabet size and *l *is the motif length (without the speedup the increase is *a*^*l *^fold; see ST2 in Additional File 2). While *Sig' *is in these examples 3-50 times more computationally expensive than *Sig*, its calculation is tractable, increasing approximately linearly as dataset size increases (ST2 in Additional File 2). For very large datasets, the computational costs seem to be much less. *Sig*_*v *_increases the number of calculations ∑^*n*^*C*_*j *_fold, for all values of *j *between *k *and *n*, where *n *is the number of clusters in the dataset and *k *is the support of the motif. The rapid increase of *Sig*_*v *_calculation runtime as the dataset size increases makes it impractical for larger datasets (ST2 in Additional File 2). *Sig'*_*v *_performs poorly compared to *Sig*, it increases the number of calculations ^*a*+*l*-1^*C*_l_*∑^*n*^*C*_*j *_fold, for all values of *j *between *k *and *n*, for each motif length considered. The computational cost of *Sig'*_*v *_calculation is unsuitable for high throughput analysis in spite of its superior accuracy; it took over a day on a standard workstation for datasets with more than 10 proteins.

### Comparison of the four scoring schemes with the ELM benchmarking dataset

The ELM benchmarking dataset is a widely used standard by which motif discovery methods can be tested, compared and trained. Although the dataset is biologically unrealistic for most discovery situations (each of the proteins in each dataset is guaranteed to contain one or more instances of the functional motif), the data allows direct comparison of the attributes of scoring schemes. Table 4 compares the *Sig' *and *Sig *scoring schemes on the 16 ELM benchmarking datasets (version - 8 April 2009). Residues with an IUPRED [24] score of less then 0.2 were masked, as were under-conserved residues by relative local conservation masking using metazoan orthologues alignments retrieved by GOPHER [24].

**Table 4:** Comparison of the *Sig *and *Sig' *scoring for the top ranking motifs matching the known interaction motif.

Dataset^a^	Sig'^b^	Sig^c^	ELM^d^	Motif^e^	k (N)^f^
LIG_CtBP	**3.4E-13**	**2.5E-09**	P. [DEN]L [VAST]	P [ILM]DL (1)	15(30)

TRG_ER_KDEL_1	**1.9E-11**	**3.0E-15**	[KRHQSAP] [DENQT]EL$	DE.$ (1)	9(11)

LIG_PCNA	**2.2E-11**	**1.7E-09**	Q.. [ILM].. [FHM] [FHM]	Q.. [IL]..FF (1)	11(19)

MOD_SUMO	**5.2E-11**	**1.1E-05**	[VILAFP]K. [EDNGP]	V.VK.EP (1)	4(29)

LIG_SH3_2	**6.7E-05**	**5.1E-04**	P..P. [KR]	P. [LV]P. [KR] (1)	5(7)

LIG_AP_GAE_1	**1.9E-04**	**4.0E-03**	[DE] [DES].F. [DE] [LVIMFD]	D.F..F.S..P (1)	3(7)

LIG_Dynein_DLC8_1	**6.0E-04**	**7.0E-03**	[KR].TQT	K.TQ.P (1)	3(7)

LIG_RGD	**9.6E-04**	**5.0E-03**	RGD	RGD (1)	6(13)

LIG_CYCLIN_1	**2.0E-03**	0.012	[RK].L.{0,1} [FYLIVMP]	RR.L.{0,1}F (1)	4(18)

LIG_Clathr_ClatBox_1	0.011	0.054	L [ILM]. [ILMF] [DE]	[FL].D [FLM] (1)	8(14)

LIG_14-3-3_1	0.013	0.186	R. [^P] [ST] [^P]P	R.R..S (1)	4(4)

LIG_NRBOX	0.014	0.082	L..LL	L..LL. [ST] (2)	5(8)

LIG_RB	0.96	1.00	[LI].C. [DE]	E.L.C.E (29)	3(25)

LIG_14-3-3_3	0.95	1.00	[RHK] [STALV]. [ST]. [PESRDIF]	R [ST].S (13)	7(7)

LIG_HP1_1			P.V. [LM]		0(8)

MOD_N-GLC			N [^P] [ST]		0(5)

TRG_LysEnd_APsAcLL_1			[DER]...L [LVI]		0(10)

(a) The ELM dataset used. (b) The *Sig' *score of the top ranking motif matching the known interaction motif. (c) The *Sig *score of the top ranking motif matching the known interaction motif. (d) The regular expression of the true functional motif. (e) The regular expression of the top ranking motif that matches the known ELM. Significant motifs (*p *< 0.01) are shown in bold. (f) The number of proteins in the dataset containing the variant of the motif discovered and the number of proteins in the dataset (in brackets)

*Sig' *scored the true positive motif as more significant than the *Sig *scoring scheme in all datasets with the exception of the highly significant TRG_ER_KDEL motif which was scored as more significant by *Sig*. For one of these, LIG_CYCLIN_1, this resulted in the returned motifs becoming significant (cut-off of 0.01). Two false positives (LIG_14-3-3_3 and LIG_NRBOX) were also returned as significant by *Sig' *which were not returned by *Sig*, however in both cases these motifs were off target motifs, true functional motifs involved in the regulation of the proteins in the datasets (See ST1 in Additional File 2). One other example of an off target motifs was significantly over represented by both scoring schemes, two motifs with strong nuclear localisation, LIG_RB and LIG_PCNA, returned arginine/lysine rich nuclear localisation motifs.

## Conclusions

We have shown how more exact calculations of short non-ambiguous protein motif probabilities may be calculated efficiently, and the computational cost associated with these calculations. We propose the use of *Sig' *calculations as the scoring scheme for motif discovery with current typical computational resources, as it offers the best trade off between speed and accuracy. Although not as accurate, the *Sig *score will, in circumstances where a motif is obviously enriched, return a close approximation of the true score. Thus, a useful technique for high-throughput analyses is to recover datasets of interest using the quicker *Sig *statistical framework at low stringency (it is a conservative score) before re-running the data using the more accurate statistics. However, for more general use the *Sig' *statistical framework is both efficient and accurate enough to suffice.

Improving these statistical measures has a strong bearing on how to efficiently rank potential novel motifs discovered in searches of many datasets derived from across the interactome. Future work will need to focus on developing better statistical measures for ambiguous motifs (ambiguous in terms of both alternative amino acids at certain positions, and in terms of variable length spacers). The problem is non-trivial as the combinatorial explosion introduced by ambiguity and the complex protein groupings that allow a motif to reach a given support makes a true ambiguous motif significance computationally intractable. Advances in methodology [21], dataset design [25] and motif enrichment [26], in association with the statistical framework described here, have put computational SLiM discovery in a position where it can complement experimental methods for the discovery of novel functional SLiMs. As our knowledge of protein-protein interaction, cellular localisation and keyword classification of proteins improves, these methods should aid in the discovery of the vast number of SLiMs remaining to be discovered by pinpointing particular residues of strong biological interest within a robust statistical framework.

## Abbreviations

SLiM: Short Linear Motif; Sig: Significance.

## Authors' contributions

ND formulated, developed, refined and tested the methods, and wrote the manuscript. DS was involved in initial discussions of the problem and in the writing of the manuscript. RE was involved in the initial formulation, and in the writing of the manuscript. All authors read and approved the final manuscript.

## Appendix

### Improving the computational efficiency of the *Sig' *calculation

The major bottleneck in the calculation of *Sig' *for a motif p-value, *p*_*μ*_, is the calculation of *p' *of all motifs with a cumulative *p*-value equal to or less than *p*. For longer motifs the complexity can make search times unfeasable, since the number of possible motifs grows exponentially with motif length. However, methods can take advantage of the inherent redundancy in the motif space due to the commutative nature of the motif occurrence probability calculations, *p*_*motif *_(Equation 1). Motifs which contain the same number of each residue from the alphabet A (where each residue can have between 0 and *l *occurrences in the motif) will have the same *p*_*motif*_. This means that the order of the residues in the motif has no effect on a motif's occurrence probability (e.g. RGG, GRG, GGR all have the same *p*_*motif*_). Exploiting the redundancy by calculating motif groupings with the same value *p*_*motif *_yields large increases in the speed of calculations. The value *p' *will be the same for each motif in a grouping and therefore it is necessary to only calculate this value once for each group, *p'*_*group*_, rather than for each motif.

#### Equation 9. *Sig' *calculation for non-redundant motif groupings

A non-redundant *Sig' *can be calculated using Equation 9. G is the set of all distinct unordered groupings of residues for each partition in *P*. A partition describes the number of unique residues occurring in a grouping. For example, there are 3 possible partitions for motifs of length 3, *q *= {(3),(1,2),(1,1,1)} meaning a 3-mer can have 3 residues which are the same, two residues which are the same and another different residue or 3 residues which are different respectively. The set *q *corresponds to the set *P *= {(0,0,1),(1,1,0),(3,0,0)}. For example, RGG, GRG, GGR can be considered as a grouping R|GG, which has a partition *P *= (1,1,0) and *q *= (1,2). RGD, RDG, GRD, GDR, DGR and DRG can be considered as a grouping R|G|D, which has a partition *P *= (3,0,0) and *q *= (1,1,1) and RRR, can be considered as a grouping RRR, which has a partition *P *= (0,0,1) and *q *= (3).

#### Equation 10. Set of all possible partitions for a motif of length *l*

The number of possible partitions for a given motif length can be described as the cardinality of set P and follows the series {1,2,3,5,7,11,15,22} as l goes from 1 to 8. Each partition has *Cg*_*i *_distinct unordered groupings of residues and each of these groupings has *Cm*_*i *_possible ordered motif combinations. For example, a grouping with P = (0,0,1), 3 of the same amino acids eg. RRR, can only be chosen 1 way while P = (3,0,0), 3 different amino acids, can be chosen 6 ways.

#### Equation 11. Number of distinct ordered groupings for a partition

where

#### Equation 12 Number of ordered motif combinations in a grouping

To calculate the *p'*_*group *_value for each non-redundant motif group, it is first necessary to define all such groupings. This can be achieved efficiently using the recursive algorithm described in the pseudocode found in SF1 in Additional File 2. The total number of distinct non-redundant motifs of length l and an alphabet of size *a*, *R*_*nr*_, is calculated as:

Equation 13. Number of non-redundant motifs of length l

## Supplementary Material

Additional file 1 **Slimsuite software package**. This ZIP file contains the slimsuite software package used with this submission. Click here for file

Additional file 2 **Supplemental materials**. This file contains all supplementary tables and figures. Click here for file
