# Supplementary material for: Estimation and efficient computation of the true probability of recurrence of short linear protein sequence motifs in unrelated proteins
Source: BMC Bioinformatics. 2010 Jan 7;11:14. doi: 10.1186/1471-2105-11-14 (PMC2819990; doi:10.1186/1471-2105-11-14)
Supplement: Additional file 1 — Slimsuite software package. This ZIP file contains the slimsuite software package used with this submission. [file 1471-2105-11-14-S1.ZIP › slimsuite/GOPHER Manual.pdf]

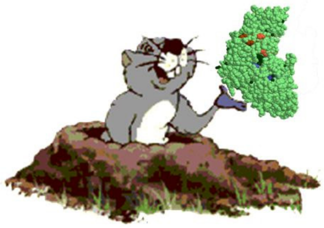

Generation of Orthologous Proteins from High-throughput Evolutionary Relationships

Richard J. Edwards (2006)

1: Introduction 2
1.1: Version 2
1.2: Using this Manual 2
1.3: Getting Help 2
1.4: Why use GOPHER? 3
1.5: Installation 3
1.5.1: Files Required 3
1.5.2: Programs Used by GOPHER 4
1.5.3: Setting up the INI File 4
2: Fundamentals 5
2.1: Running GOPHER 5
2.1.1: The Basics 5
2.1.2: Interactivity and Verbosity settings 5
2.1.3: Forking 5
2.1.4: Options 5
2.2: Input 5
2.2.1: Input sequences 5
2.2.2: Removing 'Variant'/In-Paralogue Queries 6
2.2.3: Limiting Orthologues by Species/Database 6
2.3: Output 7
2.3.1: Log Files 7
3: The GOPHER Algorithm 8
3.1: Algorithm Overview 8
3.1.1: Controlling Which Parts of GOPHER Run 10
3.1.2: Saving disk space 10
3.2: GABLAMO Sequence Similarity 11
3.3: Recommended BLAST Database 12
4: Appendices 13
4.1: Appendix I: Command-line Options 13
4.1.1: How to Use this Section 13
4.1.2: Option Types 13
4.1.3: INI Files 13
4.1.4: Option Precedence 13
4.1.5: Forking Options 14
4.1.6: Command-line Options 14
4.2: Appendix II: Distributed Python Modules 17
4.3: Appendix III: Log Files 17
4.4: Appendix IV: Species Codes for IPI & EnsEMBL 18
4.5: Appendix V: Troubleshooting 19
4.6: Appendix VI: References 19

1: Introduction

Software manuals are boring: boring to write and probably even more boring to read. I have therefore tried to keep this one concise. However, given (a) my propensity to waffle, (b) the fact that I am a biologist and not a computer scientist, and (c) my lack of experience in writing manuals, there is a good chance that the pleiotropic affect of this is a lack of clarity and/or coherence. For this I apologise, and encourage anyone out there to send in errata and/or suggested improvements. The fundamentals should be covered under the Section 2 **Fundamentals**, including **Input**, and **Output**. More details can be found in later sections and gluttons for punishment can get even more information in the **Appendices**.

Like the software itself, this manual is a 'work in progress' to some degree. If the version you are now reading does not make sense, then it may be worth checking the website to see if a more recent version is available, as indicated by the **Version** section of the manual. Good luck.

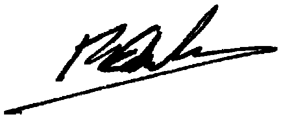

Rich Edwards, 2006.

1.1: Version

This manual is designed to accompany GOPHER version 1.7.

The manual was last edited on 07 February 2006.

1.2: Using this Manual

As much as possible, I shall try to make a clear distinction between explanatory text (this) and text to be typed at the command-prompt etc. Command prompt text will be `written in Courier New` to make the distinction clearer. Program options, also called 'command-line parameters', will be **`written in bold Courier New`** (and coloured **`red`** for fixed portions or **`dark red`** for user-defined portions, such as file names etc.). Command-line examples will be given in (purple) *`italicised Courier New`*. Optional parameters will (where I remember) be [in square brackets]. Names of files will be marked in normal text by (dark yellow) **Bold Times New Roman**.

1.3: Getting Help

Much of the information here is also contained in the XXX website (http://www.bioinformatics.rcsi.ie/~redwards/) and the documentation of the Python modules themselves. A full list of command-line parameters can be printed to screen using the **`help`** option, with short descriptions for each one.

```
python gopher.py help
```

If none of the above are of help, then please e-mail me (richard.edwards@ucd.ie) whatever question you have. If it is the results of an error message, then please send me that and/or the log file (see **Output**) too. Usually, it will be a problem with the input files (possibly formatting) but there are probably still a few bugs in there somewhere too.

1.4: Why use GOPHER?

The rapid and automated generation of orthologous protein datasets is useful for many bioinformatics applications. True orthologue identification requires the use of phylogenetic information, in which outgroup sequences are used to identify the closest clustering homologues in the different species. Phylogenetic analysis can also identify "in-paralogues" – lineage-specific gene duplications – which can hinder compilation of orthologues as they are both true orthologues to the same protein in a different species. For high-throughput analyses, this can be impractical. Identifying suitable outgroups is not a trivial task and phylogenetic inference has its own weaknesses and biases. As a result, pairwise sequence comparisons are routinely used in its place.

One common approach is to use BLAST (Altschul et al. 1990) scores or e-values. For example, orthologues are often identified using the "Mutual Best Hit" (MBH) approach. Under this model, human sequence A and mouse sequence B are only considered orthologues if A has the best score when B is BLASTed against the human genome, and B has the best score when A is BLASTed against the mouse genome. MBH has severe flaws, however. In-paralogues in one species will be missed, with only one identified as an orthologue, while in-paralogues in both the species being compared may disrupt the MBH assignment totally. Using BLAST for this purpose introduces even more problems. BLAST scores are notoriously sensitivity to sequence length and multi-domain proteins. If multiple regions of one protein are homologous to the same region of the other then the BLAST score will be artificially amplified. At the other end of the scale, unrelated proteins will generate BLAST hits if they both contain similar low complexity regions, such as poly-glutamine repeats or leucine-rich regions. This can be avoided using the Complexity Filter but at the risk of artificially decreasing the BLAST scores of closely related sequences with low complexity regions, which are of most interest for orthologue assignment.

GOPHER attempts to combine the high speed of BLAST MBH-based methods with the accuracy obtained by phylogenetic inference. By considering the relative sequence similarities of a query sequence, different putative orthologues, and paralogous sequences, GOPHER identifies those proteins that, assuming an approximately constant rate of evolution within the protein family, should be true orthologues of the query sequence. Details are given in section **3: The GOPHER Algorithm**.

1.5: Installation

GOPHER is distributed as a number of open source Python modules. It should therefore work on any system with Python installed without any extra setup required – simply copy the relevant files to your computer and run the program (see **Running GOPHER**, below.)

If you do not have Python, you can download it free from www.python.org at http://www.python.org/download/. The modules are written in Python 2.4. The Python website has good information about how to download and install Python but if you have any problems, please get in touch and I will help if I can.

1.5.1: Files Required

The following files are required for the program to run correctly. All these files should have been provided in the download zip file. The Python Modules are open source and may be changed if desired, although please give me credit for any useful bits you pillage. I cannot accept any responsibility if you make changes and the program stops working, however!

Note that the organisation of the modules and the complexity of some of the classes is due to the fact that most of them are designed to be used in a number of different tools. As a result, not all the options listed in the `__doc__()` (**`help`**) will be of relevance. If you want

some help understanding the way the modules and classes are set up so you can edit them, just contact me.

The additional files may all be replaced with other files in the correct format. These files are described later in this manual and/or in the Appendix.

Python Modules (*.py): gopher, rje, rje_blast, rje_dismatrix, rje_seq, rje_pam, rje_sequence, rje_uniprot

1.5.2: Programs Used by GOPHER

In addition to the python modules listed above, GOPHER makes use of the following published programs. These are freely available for downloading and installing. It is recommended that the user downloads and installs these programs according to the instructions given on the appropriate website.

BLAST: BLAST (Altschul, et al. 1990) is freely available for download from NCBI at: http://www.ncbi.nlm.nih.gov/blast/download.shtml.

CLUSTALW: ClustalW (Higgins and Sharp 1988, Thompson et al. 1994) is an old stalwart for bioinformatics and is freely available from EMBL: ftp://ftp-igbmc.u-strasbg.fr/pub/ClustalW/. Note that CLUSTALW is used as a backup for MUSCLE (below).

MUSCLE: MUSCLE (Edgar 2004) is a newer multiple alignment program available from http://www.drive5.com/muscle.

1.5.3: Setting up the INI File

It is recommended that a gopher.ini file is made and placed in the same directory as the gopher.py program. This file should contain the paths to the above programs:

```
blastpath=PATH
clustalw=COMMAND
muscle=COMMAND
```

Note that the first is a path to the BLAST programs, while for ClustalW and MUSCLE the actual program commands themselves must be included. This is to make it easier to replace these programs with alternatives. (See **Replacing Components with Other Programs**.) See the included gopher.ini file for an example. If running in windows, it is also advisable to add the `win32=T` command to the *.ini file.

NB. For `PATH` variables, directories should be separated by a forward slash (/). If paths contain spaces, they should be enclosed in double quotes: `path="example path"`. It is recommended that paths do not contain spaces as function cannot be guaranteed if they do.

2: Fundamentals

2.1: Running GOPHER

2.1.1: The Basics

If you have python installed on your system (see **Installation**), you should be able to run GOPHER directly from the command line in the form:

`python gopher.py gopher=FILENAME orthdb=FILENAME`

If running in Windows, you can just double-click the **gopher.py** file provided that there is a **gopher.ini** file with all the commands. GOPHER is not currently implemented to be menu driven.

IMPORTANT: If filenames contain spaces, they should be enclosed in double quotes: **gopher="example file"**. That said, it is recommended that files do not contain spaces as function cannot be guaranteed if they do.

2.1.2: Interactivity and Verbosity settings

By default, GOPHER will run through to completion without any user-interaction if given all the options it needs. For more interaction with the program as it runs, use the argument '**i=1**'

python gopher.py gopher=gopher_eg.fas orthdb=metazoa.fas i=1

Both the level of interactivity and the amount printed to screen can be altered, using the interactivity [**i=X**] and verbosity [**v=X**] command-line options, respectively, where **X** is the level from none (-1) to lots (2+). Although in theory **i=-1** and **v=-1** will ask for nothing and show nothing, there is a good chance that some print statements will have escaped in these early versions of the program. There is also the possibility that accessory programs may print things to the screen beyond the control of GOPHER.

Please report any irritations and suggestions for changes to what is printed at different verbosity levels.

2.1.3: Forking

If using a multiple processor machine in UNIX, GOPHER can fork out multiple processes to increase processing speed using the **forks=X** option. Each query sequence is processed by a different fork. See **4.1.5: Forking Options** for details.

2.1.4: Options

At first, you will probably want to run the program with its default parameters. If you want to change them, there are a number of parameters that can be set by the user and other options. These are described in the relevant sections and summarised in **4.1: Command-line Options**. These may be given after the run command, as above, or loaded from one or more ***.ini** files (see for **4.1.3: INI Files** for details).

2.2: Input

2.2.1: Input sequences

The main input for GOPHER is two fasta files of protein sequences:

1. The query sequences (**gopher=FILE**)

2. The search database (**orthdb=FILE**)

To get the most out of the program, one of the set fasta formats from common sequence databases should be used. The included manual for the **rje_seq.py** sequence manipulation module has more details on formats and reformatting/filtering of input sequences.

IMPORTANT: Accession numbers in the query sequence file must be **unique** and **must be in the search database** also. The first word for each sequence name in the search database must also be unique (as for any BLAST database to function correctly.)

2.2.2: Removing 'Variant'/In-Paralogue Queries

If searching with a redundant query dataset, the **allqry=F** option can be used to only retain the "best" sequence of any "In-Paralogues" (lineage-specific duplicates), splice variants etc. (see **3: The GOPHER Algorithm**). Where such variants exist, the "best" one will be selected according the source database and, where the database is the same, the longest sequence will be retained. (The database hierarchy can be set using the **dblist=X,Y,..,Z** option, where **X,Y,..,Z** constitutes a list of databases in order of preference (good to bad). See the manual for RJE_SEQ for details.)

It is recommended to use a non-redundant database for the query input, such as IPI (Kersey et al. 2004), and leave the **allqry=T/F** option set to True. IPI can be converted into an optimal format for use with GOPHER using the supplied **fasta_reformat.pl** script:

```
perl fasta_reformat.pl FILE
```

This file can also be used to reformat EnsEMBL known, novel and abinitio downloads into more accessible fasta files from which GOPHER can read the species information. If this does not work for some files, try renaming the known, novel and abinitio files from, e.g. **Anopheles_gambiae.MOZ2a.dec.pep.abinitio.fa** to **ens_ANOGA.abinitio.fas**, or **Mus_musculus.NCBIM34.dec.pep.novel.fa** to **ens_MOUSE.novel.fa**, where **ANOGA** is the SwissProt species codes for *Anopheles gambiae* and **MOUSE** is the code for *Mus musculus* (see **4.4: Appendix IV: Species Codes for IPI & EnsEMBL** for more species codes).

2.2.3: Limiting Orthologues by Species/Database

In addition to controlling the returned orthologues by restricting the sequences in the search database, a number of filters can be applied to the orthologous dataset returned, including species and database filters, using the **goodX=LIST** (retention) and **badX=LIST** (exclusion) options, where **X** is one of the following:

- acc = list of accession numbers
- seq = list of sequence names
- spec = list of species codes
- db = list of source databases [sprot,ipi,uniprot,trembl,ens_known,ens_novel,ens_scan]
- desc = list of terms that, at least one of which must be in description line

The **LIST** element can be a list **X,Y,..,Z** or a file containing the relevant terms. E.g. *goodspec=HUMAN,MOUSE,RAT baddesc=bad_desc.txt* would only retain sequences annotated as having the species codes HUMAN, MOUSE or RAT and would exclude any of these sequences that have any of the terms from the file **bad_desc.txt** in their description lines. These filters are described in more detail in the manual for RJE_SEQ.

2.3: Output

The main output for GOPHER is series of directories containing output files from the different stages of the GOPHER Run.

1. BLAST (BLAST/)
2. Orthology (ORTH/)
3. Alignment (ALN/)
4. (Optional) paralogue alignment (PARALN/)

Within each directory, all files are named in the form **AccNum.***, where **AccNum** is the accession number of the query. Main output is the alignment of orthologues, **ALN/*.orthaln.fas**. A full list of output files is given below:

File	Description	Directory
*.blast	The main BLAST results file from the initial BLAST against the search database (one-line hits only)	BLAST
*.blast.id	The fastacmd IDs of the hits from the initial BLAST	BLAST
*.gopher_blast	Statistics on the BLAST results.	BLAST
*.qry	The query sequence	BLAST
*.para.fas	Paralogues in fasta format	ORTH
*.minsim.fas	All hits exceeding minimum similarity, in fasta format	ORTH
*.orth.fas	Putative Orthologues in fasta format	ORTH
*.gopher_orth	Statistics for the putative orthologues	ORTH
*.orthaln.fas	Alignment of orthologues	ALN
*.gopher_alnfas	Statistics of orthologue alignment	ALN
*.paraln.fas	Alignment of paralogues in fasta format	PARALN
*.gopher_paraln	Statistics of paralogue alignment.	PARALN

Statistics files are delimited text files. The headers can be found in the main directory in *.header files. When concatenated, these files can be converted to MySQL build statements using **rje_mysql.py**.

2.3.1: Log Files

The GOPHER log file records information that may help subsequent interpretation of results or identify problems. Probably it's most useful content is any error messages generated. By default the log file is **gopher.log** but this can be changed with the `log=FILE` option. Logs will be appended unless the `newlog` option is used.

3: The GOPHER Algorithm

3.1: Algorithm Overview

An overview of GOPHER is given in **Figure 1**. The default protocol is as follows:

1. BLAST the sequence database (see **3.3: Recommended BLAST Database**), reporting the first 1000 hits with a BLAST threshold of $e=10^{-4}$ and the complexity filter on.
2. Repeat the BLAST of the query against the initially detected homologues with a very relaxed threshold of e=10 and without the complexity filter, and use the GABLAMO algorithm (see **3.1.1: GABLAMO Sequence Similarity**) to filter out any sequence with less than 40% global percentage similarity with the query, i.e. at least 40% of residues in the query must be similar to each potential orthologue, and keep only the most similar sequence to the query for each species. The minimum level of similarity can be changes using the `minsim=X` option. To use percentage identity or coverage instead of similarity, use the `gablamo=X` option, where `X` can be `Sim` (similarity), `ID` (identity) or `Len` (coverage).

   By default, the focus of the similarity score is the query, *i.e.* the GABLAMO percentage similarity, identity or coverage of the query sequence is used. GABLAMO is asymmetric (see **3.1.1: GABLAMO Sequence Similarity**) and so the percentage similarity, identity or coverage of the homologue may not meet the threshold even if the query does. To change the focus of the similarity measure, use the `simfocus=X` option, where `X` can be:

   a. `query` (default): %query must > minsim. (Best if query is ultimate focus and maximises closeness of returned orthologues)
   b. `hit`: %hit must > minsim. (Best if lots of sequence fragments are in searchdb and should be retained)
   c. `either`:= %query > minsim OR %hit > minsim. (Best if both above conditions are true)
   d. `both`: %query > minsim AND %hit > minsim. (Gets most similar sequences in terms of length but can be too stringent.)
3. Filter out chimp sequences as they are too closely related too their human orthologues and can disrupt the filtering of splice variants (below).
4. Each paralogue (homologous proteins in the same species) is identified and used to query the remaining orthologues as in (2), generating GABLAMO percentage similarity statistics.
5. Filter out in-paralogues (species-specific duplicates) and splice variants, as these will share the same set of "true orthologues" as the query. These are identified as those paralogues which:

   a. Have a greater percentage similarity to the query than any sequence of a different species.
   b. The query has a greater percentage similarity to the paralogue than any sequence of a different species.
6. Putative orthologues are then identified (**Figure 2**). To be considered an orthologue:

a. If the orthologue is within the same clade as the query, i.e. diverged from the query more recently than the closest paralogue:

    i. The query must have a higher global percentage similarity with the sequence than any other sequence of same species.

    ii. The sequence must have a higher global percentage similarity with the query than with the closest paralogue.

    iii. The query must have a higher global percentage similarity with the sequence than with the closest paralogue.

b. If the orthologue is outside of the most recent post-duplication clade for the query, it must not be within the post-duplication clade for another paralogue. For such an orthologue, if the query protein is not the closest protein of the query species:

    i. The query must have a higher global percentage similarity to the putative orthologue's closest paralogue than to the putative orthologue.

    ii. The putative orthologue's closest paralogue must have a higher global percentage similarity to the query than to the putative orthologue.

If the `postdup=T` option is used, these orthologues will be ignored.

7. The remaining orthologues are then aligned using MUSCLE (Edgar 2004).

8. If `paralign=T` (default), the paralogues will also be aligned using MUSCLE (Edgar 2004). If the `parasplice=T` option is used, these will include IPI splice variants that have been filtered out as in-paralogues.

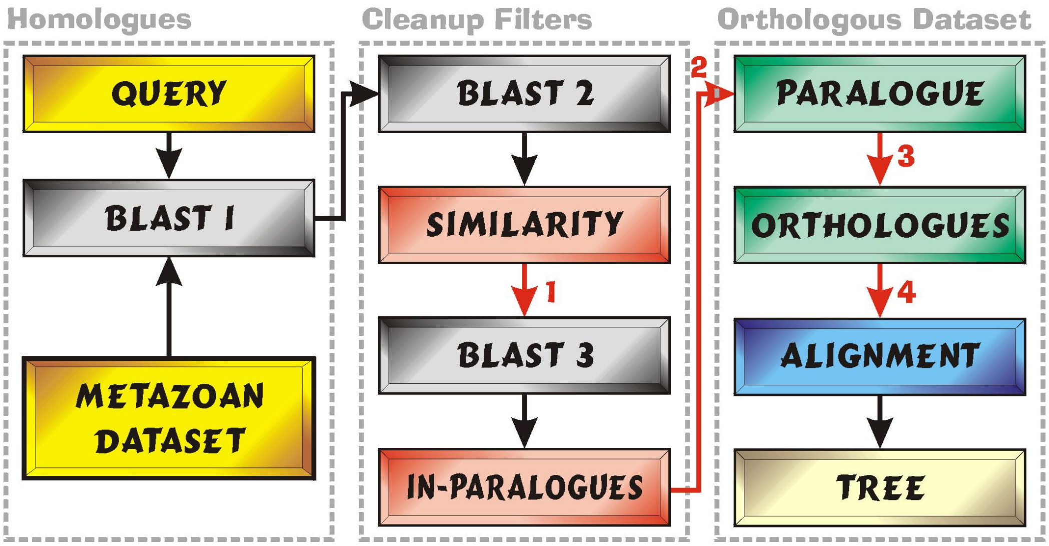

Figure 1. Overview of GOPHER process. Potential orthologues are removed at each of the four stages marked by red arrows.

(a)

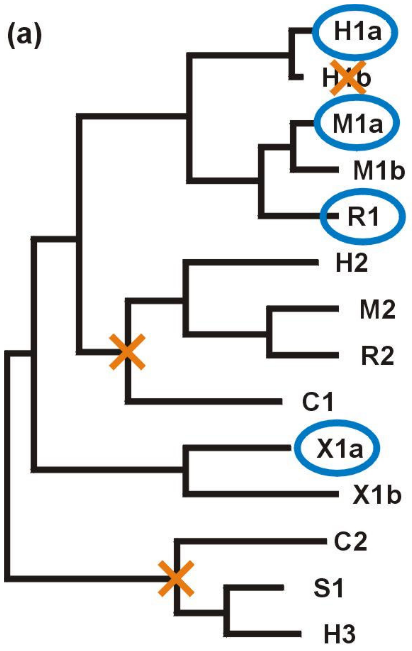

(b)

Seq	Human	Best	Orth
H1b	H1a	-	No
M1a	H1a*	Yes	Yes
R1	H1a	Yes	Yes
M1b	H1a*	No	Yes
C1	H1b	Yes	No
H2	H1a	-	No
M2	H2	No	No
R2	H2	No	No
X1a	H2	Yes	Yes
X1b	H2	No	Yes
S1	H3	Yes	No
H3	H1a	-	No
C2	H3	No	No

Figure 2. Example Orthologue Identification with GOPHER. (a) Example selection of orthologous proteins for human protein H1a, circled in blue. Inparalogue H1b is removed. Mouse sequences M1a and M1b are both orthologues of H1a but M1b is arbitrarily removed in favour of the closer M1a. C1 is the closest chicken sequence but is closer to H2. Similarly, sheep sequence S1 is part of the H3 clade. X1a is considered an orthologue, even though H2 is the closest human sequence to it, because H2 is closer to H1a than either is to X1a, while H3 is further from both H1a and X1a. H, human; M, mouse; R, rat; C, chicken; S, sheep; X Xenopus; **(b)** Table summarising relationships. Seq, sequence ID from (a); Human, closest human sequence; Best, whether closest sequence of species to H1a; Orth, whether a true orthologue of H1a.

3.1.1: Controlling Which Parts of GOPHER Run

By default, GOPHER will be run to conclusion on all query sequences. To run GOPHER up to a certain point only, use the `orthblast`, `orthfas` and `orthalign` options. To skip part of the input file and start at a particular sequence (e.g. if picking up from killing the program for any reason), use the `startfrom=X` option, where `X` is an accession number or sequence ID. When GOPHER is running at a given level of execution (`orthblast`, `orthfas` or `orthalign`) and the files from a previous level are missing, they will be generated. To turn this off, and only run the selected level, use `repair=F`. E.g. to only align those query datasets for which orthologues have already been identified, use the commands *`orthalign repair=F`*.

If GOPHER is run and some results files already exist, then those parts of GOPHER will be skipped. By default, these files will only be used if they are newer than the files needed to generate them. To over-ride the time/date check and use existing results files of any age, use the `ignoredate=T` option. To force GOPHER to regenerate results for the given level of execution (`orthblast`, `orthfas` or `orthalign`), use the `force=T` option. To force GOPHER to regenerate results for all levels, use the `fullforce=T` option.

3.1.2: Saving disk space

A full GOPHER run on a large query dataset will use up quite a lot of disk space. To reduce this somewhat, GOPHER will delete some of the intermediate files during execution. To keep these extra intermediates, use the `savespace=F` option.

3.2: GABLAMO Sequence Similarity

Although fast to generate, using BLAST scores or e-values alone for measuring relative sequence similarity introduces many problems. BLAST scores are notoriously sensitivity to sequence length and multi-domain proteins. If multiple regions of one protein are homologous to the same region of the other then the BLAST score will be artificially amplified. At the other end of the scale, unrelated proteins will generate BLAST hits if they both contain similar low complexity regions, such as poly-glutamine repeats or leucine-rich regions. This can be avoided using the Complexity Filter but at the risk of artificially decreasing the BLAST scores of closely related sequences with low complexity regions, which are of most interest for orthologue assignment.

To avoid reducing the scores associated with highly similar sequences, one alternative is to leave the Complexity Filter off and use a stricter BLAST score or e-value cut-off to filter out weak homologues. The appropriate threshold can be hard to determine, however, as neither BLAST score nor e-value translate into the degree of similarity in a particularly intuitive fashion. A more intuitive measure is the percentage sequence identity or similarity between two sequences, which can be calculated using pairwise sequence alignment programs, such as ALIGN (Pearson 2000). The disadvantage is that performing multiple pairwise sequence alignments is slow compared to a single BLAST search, which can have serious run-time issues with large genomic analyses. Furthermore, pairwise sequence alignment has well documented drawbacks (Rosenberg 2005). The most obvious is that the program will enforce an alignment, whether the sequences have reasonable similarity or not. As a result, when default settings are used, even random sequences will return a percentage identity of 20% or more for the shorter sequence. Furthermore, if one is interested in the overall similarity of two sequences incorporating domain duplications or rearrangements then pairwise alignment will fail.

GOPHER makes use the GABLAMO (Global Alignment from BLAST Local AlignMent (Ordered)) algorithm (Edwards and Davey 2006), which returns a set of informative and intuitive pairwise sequence similarity statistics using the results from a basic BLAST search. GABLAMO compiles local sequences alignments generated by BLAST and returns ordered sequence coverage, identity and similarity statistics independently for query and hit sequences (**Figure 3**).

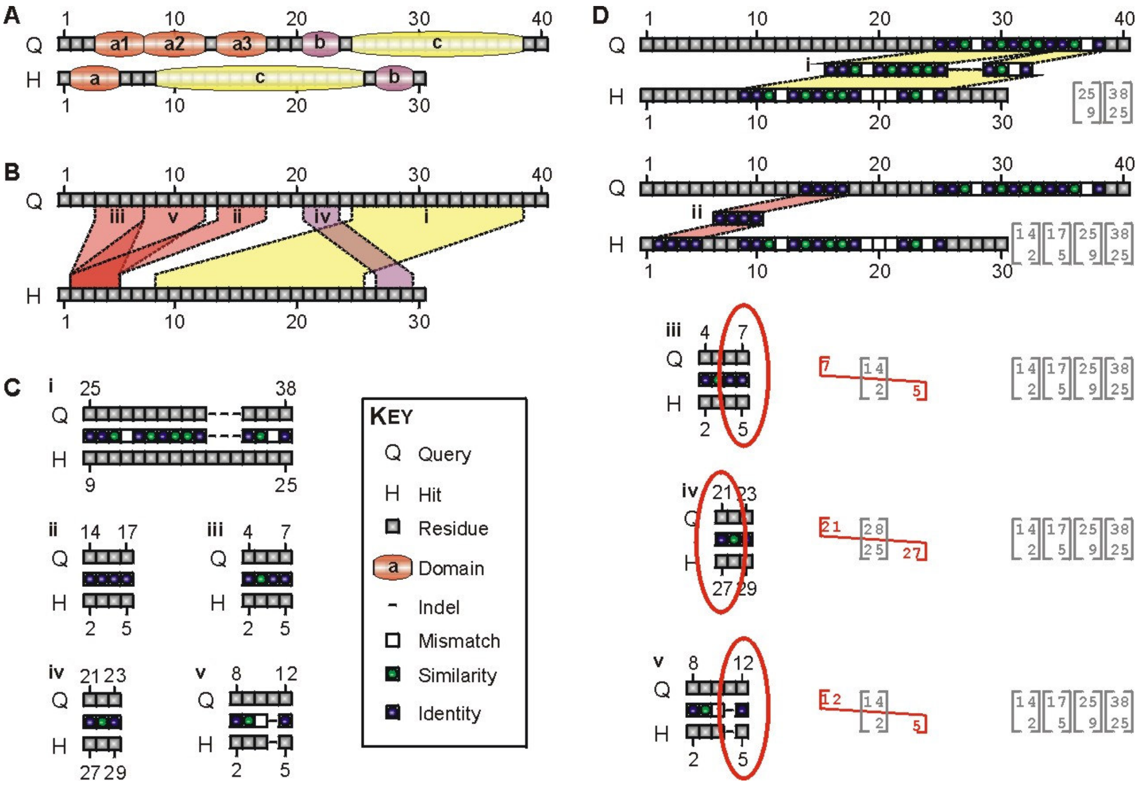

Figure 3. Overview of GABLAMO (Global Alignment from BLAST Local AlignMent (Ordered)) Percentage Identity/Similarity calculations. method. ***(A)*** Each Query-Hit pair from the BLAST search is compared in a pair-wise fashion. For simplicity, this example has used artificially short sequences and "Domains", which for this purpose are regions of local homology. ***(B)*** BLAST detects five regions of local homology, labelled i-v in order of score, producing ***(C)*** five local alignments between regions of query and hit. ***(D)*** Each local alignment is taken in order of BLAST score and mismatches, similarities and identities transposed on to the Query and Hit, assuming that residue has not already been assigned a better status. Pairs of Query and Hit positions are also stored. If, as in iii, an alignment would split an existing pair of Query-Hit positions, it is judged to be in conflict with the linear ordering of previous alignments and is not added.

3.3: Recommended BLAST Database

For mammalian analyses, it is recommended to construct a search database of the following metazoan sequences:

1. All IPI (Kersey, et al. 2004) sequences for metazoan species.
2. All EnsEMBL (Birney et al. 2006) known and novel sequences for metazoan species not downloaded from IPI.
3. All UniProt (Bairoch et al. 2005) metazoan sequences (October 2005 download), excluding those species downloaded from IPI.

Versions of this assembled database will be available from the GOPHER website (bioinformatics.ucd.ie/shields/software/gopher/) and periodically updated.

4: Appendices

4.1: Appendix I: Command-line Options

4.1.1: How to Use this Section

This section lists all the Command-line options than may be of use when using GOPHER. Note that different options are associated with different modules. These are indicated by the name of the module given (**in brackets**). Default values are given [in square brackets]. Not all the options for a given module are listed here but can be found by printing the `__doc__` attribute of the module at a Python prompt, or using the **`help`** option:

`print gopher.__doc__` (in Python)

`python gopher.py help` (commandline)

This section has not been completed. For now, the listing provided as part of the module documentation is given. This shall be expanded with time. (Hopefully soon.) In the meantime, please contact me if you want any further details of a specific option and/or advice as to when (not) to use it.

4.1.2: Option Types

There are essentially three types of command-line option:

1. Those that require a value (numerical or text), **`option=X`**. Those that require a filename as the value will be witten: **`option=FILE`**
2. True/False (On/Off) options, **`option=T/F`**. For these options:
   a. **`option=F`** and **`option=F`**alse are the same and turn the option off
   b. **`option`**, **`option=T`** and **`option=True`** are the same and turn the option on
3. List options. These are like the value options but have multiple values, separated by commas: **`option=X,Y`**. Where `..` is used, the number elements is optional, e.g. **`option=X,Y,..,Z`** could take **`option=X`** or **`option=A,B,C,D`**. Where **`option=LIST`** is used, the number of elements is optional and **`LIST`** could actually be the name of a file containing the list of elements.

4.1.3: INI Files

As well as feeding commands in on the command-line, any options listed can also be save in a plain text file and called using the option **`ini=FILE`**. Automatically, the program will read in any options from the file **gopher.ini** and **rje.ini**, if present.

4.1.4: Option Precedence

Later options will supersede earlier ones if they are mutually exclusive. Options from an ini file will be inserted into the list at the point the ini file is called. (At the start for **rje.ini**.) This means that ini file options can be over-ruled, e.g.

`gopher.py ini=eg.ini i=1` will supersede any interactivity setting in **eg.ini** with **`i=1`**.

`gopher.py i=1 ini=eg.ini` will use any interactivity setting in **eg.ini** and over-rule **`i=1`**.

4.1.5: Forking Options

Forking is controlled by three main parameters:

- `forks=X` sets the number of processes to fork at any given time.
- `killforks=X` sets the number of seconds without any activity from any forks before the program will commit suicide.
- `noforks=T/F` sets whether forks are used at all.

IMPORTANT: Forking has been implemented for UNIX only. (Python does not support forking in Windows.) If running in windows, use the `win32=T`, `forks=0` or `noforks=T` options.

4.1.6: Command-line Options

Option	Description	Default	Module
	**General Dataset Input/Output**		
`gopher=FILE`	Loads sequences from FILE	[None]	gopher
`orthdb=FILE`	BLAST database for homologue searching	[None]	gopher
`allqry=T/F`	Whether all query sequences are to have alignments etc. (If False, the 'best' in-paralogues only are used.)	[True]	gopher
`startfrom=X`	Accession Number / ID to start from.	[None]	gopher
`v=X`	Sets verbosity (-1 for silent)	[0]	rje
`i=X`	Sets interactivity (-1 for full auto)	[0]	rje
`d=X`	Data output level (0-3)	[1]	haqesac
`log=FILE`	Redirect log to FILE	[gopher.log]	rje
`newlog=T/F`	Create new log file.	[False]	rje
	**GOPHER**		
`postdup=T/F`	Restrict orthologues to within the query species' post-duplication clade	[False]	gopher
`minsim=X`	Minimum %similarity of Query for each "orthologue".	[40.0]	gopher
`gablamo=X`	Measure to use for minimum %, where `X` can be `Sim` (similarity), `ID` (identity) or `Len` (coverage).	[Sim]	gopher
`simfocus=X`	Style of MinSim used, where `X` can be:<br>`query` = %query must > minsim;<br>`hit` = %hit must > minsim;<br>`either` = %query > minsim OR %hit > minsim;<br>`both` = %query > minsim AND %hit > minsim.	[query]	gopher
`paralign=T/F`	Whether to produce paralogue alignments (>minsim) in PARALN/.	[True]	gopher

Option	Description	Default	Module
parasplice=T/F	Whether to allow splice variants in paralogue alignments (where identified).	[False]	gopher
orthblast	Run to blasting versus orthdb (Stage 1).		gopher
orthfas	Run to output of orthologues (Stages 2-6).		gopher
orthalign	Run to alignment of orthologues (Stage 7).	Default	gopher
repair=T/F	Repair mode - replace previous files if date mismatches or files missing. (Skip missing files if False)	[True]	gopher
force=T/F	Whether to force execution at current level even if results are new enough.	[False]	gopher
fullforce=T/F	Whether to force current and previous execution even if results are new enough.	[False]	gopher
ignoredate=T/F	Ignores the age of files and only replaces if missing.	[False]	gopher
savespace=X	Save space by deleting intermediate blast files during orthfas.	[True]	gopher
	**Sequence Filters**		
goodX=LIST	Only keeps sequences meeting the requirement of **LIST** (**X,Y,..,Z** or a **FILE** which contains a list).<br>➢ goodacc = list of accession numbers<br>➢ goodseq = list of sequence names<br>➢ goodspec = list of species codes<br>➢ gooddb = list of source databases<br>➢ gooddesc = list of terms that, at least one of which must be in description line	[None]	rje_seq
badX=LIST	As goodX but excludes rather than retains sequences	[None]	rje_seq
accnr=T/F	Check for redundant Accession Numbers/Names on loading sequences.	[False]	rje_seq
seqnr=T/F	Make sequence Non-Redundant	[False]	rje_seq
specnr=T/F	Non-Redundancy within same species only	[False]	rje_seq
nrid=X	%Identity (GABLAM) cut-off for Non-Redundancy	[100.0]	rje_seq
nrsim=X	%Similarity (GABLAM) cut-off for Non-Redundancy	[100.0]	rje_seq
autofilter=T/F	Whether to apply sequence filters upon loading	[True]	rje_seq

Option	Description	Default	Module
	**System Info**		
blastpath=PATH	Path to BLAST programs * Use forward slashes (/)	['c:/bioware/blast/']	rje_blast
clustalw=PATH	Path to CLUSTALW program * Use forward slashes (/)	['c:/bioware/clustalw.exe']	rje_seq
muscle=PATH	Path to MUSCLE * Use forward slashes (/)	['c:/bioware/muscle.exe']	rje_seq
win32=T/F	Run in Win32 Mode	[False]	rje
memsaver=T/F	Run in "Memory Saver" mode	[False]	rje
	**Forking**		
forks=X	Number of forks	[0]	rje
killforks=X	Number of seconds of inactivity before killing forks	[3600]	rje
noforks=T/F	Option to over-ride and cancel forking	[False]	rje

4.2: Appendix II: Distributed Python Modules

This appendix is liable to go out of date. Please look at the documentation within the modules themselves for more details. (And, if you're brave, look at the code!)

Module	Description	Classes
**gopher**	Main GOPHER module containing primary code	Gopher, GopherFork
**rje**	General module containing Classes used by all my scripts plus a number of miscellaneous methods.	RJE_Object_Shell, RJE_Object, Info, Out, Log
**rje_blast**	Performs BLAST searches and loads results into objects.	BLASTRun, BLASTSearch, BLASTHit, PWAln
**rje_dismatrix**	Contains Classes and methods for Distance Matrix	DisMatrix
**rje_pam**	This module handles functions associated with PAM matrices. A PAM1 matrix is read from the given input file and multiplied by itself to give PAM matrices corresponding to greater evolutionary distance. (PAM1 equates to one amino acid substitution per 100aa of sequence.)	PamCtrl, PAM
**rje_seq**	Contains Classes and methods for sets of DNA and protein sequences. (Currently only protein sequences supported.)	SeqList, Sequence, DisMatrix
**rje_sequence**	Contains Classes and methods for individual sequences	Sequence
**rje_uniprot**	Contains methods for parsing UniProt info	UniProt

4.3: Appendix III: Log Files

This part of the manual has not yet been written. Sorry! Contact the author if you have questions about the log files.

4.4: Appendix IV: Species Codes for IPI & EnsEMBL

The following EnsEMBL and IPI species and species codes are recognized by **rje_sequence.py**:

Species	Common Name	Species Code
Anopheles gambiae	Mosquito	ANOGA
Apis mellifera	Bee	APIME
Bos taurus	Cow	BOVIN
Caenorhabditis elegans	Nematode	CAEEL
Canis familiaris	Dog	CANFA
Ciona intestinalis	Sea squirt	CIOIN
Danio rerio	Zebrafish	BRARE
Drosophila melanogaster	Fruitfly	DROME
Fugu rubripes	Pufferfish	FUGRU
Gallus gallus	Chicken	CHICK
Homo sapiens	Human	HUMAN
Macaca mulatta	Macaque	MACMU
Monodelphis domestica	Opossum	MONDO
Mus musculus	Mouse	MOUSE
Pan troglodytes	Chimp	PANTR
Rattus norvegicus	Rat	RAT
Saccharomyces cerevisiae	Yeast	YEAST
Tetraodon nigroviridis	Pufferfish	TETNG
Xenopus tropicalis	Frog	XENTR

To add more species, the **extractDetails()** method of the **Sequence** class needs to be edited. Please contact the author for assistance.

4.5: Appendix V: Troubleshooting

Currently, this is a small section as I have not had enough feedback to have FAQs, or anything like that. Here is a list of things that I think MAY cause problems to the unwary:

- Giving file names with spaces. (Only the first word will be taken as the filename)
- Including spaces in paths to programs etc.
- Incorrect formatting of input files. Check that all the sequence names have species codes if they are not standard UniProt or GenBank downloads.
- I'm not sure when but there is a possibility of problems if running in Windows without the `win32=T` option, especially if using forks.

4.6: Appendix VI: References

Altschul SF *et al.* (1990). "Basic local alignment search tool", *J Mol Biol*, **215**, 403-410.

Bairoch A *et al.* (2005). "The Universal Protein Resource (UniProt)", *Nucleic Acids Res.*, **33**, D154-159.

Birney E *et al.* (2006). "Ensembl 2006", *Nucleic Acids Res.*, **34**, D556-561.

Edgar RC (2004). "MUSCLE: a multiple sequence alignment method with reduced time and space complexity", *BMC Bioinformatics*, **5**, 113.

Edwards RJ and Davey NE (2006). "GABLAM: Global Alignment from BLAST Local AlignMent". *http://bioinformatics.ucd.ie/shields/software/gablam/*

Higgins DG and Sharp PM (1988). "CLUSTAL: a package for performing multiple sequence alignment on a microcomputer", *Gene*, **73**, 237-244.

Kersey PJ *et al.* (2004). "The International Protein Index: an integrated database for proteomics experiments", *Proteomics.*, **4**, 1985-1988.

Pearson WR (2000). "Flexible sequence similarity searching with the FASTA3 program package", *Methods Mol Biol*, **132**, 185-219.

Rosenberg MS (2005). "Evolutionary distance estimation and fidelity of pair wise sequence alignment", *BMC Bioinformatics.*, **6**, 102.

Thompson JD, Higgins DG and Gibson TJ (1994). "CLUSTAL W: improving the sensitivity of progressive multiple sequence alignment through sequence weighting, position-specific gap penalties and weight matrix choice", *Nucleic Acids Res*, **22**, 4673-4680.
