# Supplementary material for: Estimation and efficient computation of the true probability of recurrence of short linear protein sequence motifs in unrelated proteins
Source: BMC Bioinformatics. 2010 Jan 7;11:14. doi: 10.1186/1471-2105-11-14 (PMC2819990; doi:10.1186/1471-2105-11-14)
Supplement: Additional file 1 — Slimsuite software package. This ZIP file contains the slimsuite software package used with this submission. [file 1471-2105-11-14-S1.ZIP › slimsuite/PEAT Appendices.pdf]

Protein Evolution Analysis Toolkit

Richard J. Edwards (2006)

1: Introduction 3
- 1.1: Last Edit 3
- 1.2: Using this Manual 3
- 1.3: Getting More Help 3
- 1.4: Installation 4
  - 1.4.1: Files Required 4
- 1.5: Citations 4

2: Distributed Programs 5
- 2.1: BADASP 5
- 2.2: CompariMotif 5
- 2.3: GASP 6
- 2.4: GABLAM 6
- 2.5: GOPHER 6
- 2.6: HAQESAC 7
- 2.7: PRESTO 7
- 2.8: SeqMapper 7
- 2.9: SLiMFinder 8

3: Appendices 9
- 3.1: Command-line Options 9
  - 3.1.1: How to Use this Section 9
  - 3.1.2: Option Types 9
  - 3.1.3: INI Files 9
  - 3.1.4: Setting up the INI File 9
  - 3.1.5: Interactivity and Verbosity settings 10
  - 3.1.6: Option Precedence 10
  - 3.1.7: General Command-line Options 11
- 3.2: Distributed Python Modules 12
  - 3.2.1: Main Programs 12
  - 3.2.2: Main Accessory Applications 13
  - 3.2.3: Accessory Modules 14
  - 3.2.4: Miscellaneous Additional Accessory Applications 15
- 3.3: Additional Files Required 16
  - 3.3.1: Amino Acid Property Matrix 16
  - 3.3.2: PAM Matrix File 16
  - 3.3.3: Using an unscaled matrix 17
- 3.4: External Components of PEAT Programs 17
- 3.5: Replacing Components with Other Programs 17
  - 3.5.1: Alignment programs 18
  - 3.5.2: Tree-drawing programs 18
  - 3.5.3: Wrapper scripts 18
  - 3.5.4: Incorporating Other Programs into the Python Code 18

3.6: Log Files **19**
3.7: Troubleshooting **19**
3.8: References **20**

1: Introduction

The **Protein Evolution Analysis Toolkit** is actually several different programs, each with their own identity, functionality and, where time has allowed, manuals and websites. They are packaged together, however, as many of the programs share common modules and most have the same basic structure and input/output/parameter principles. This document is designed to cover this common ground in a bit more detail so that the individual manuals do not have to. This first section deals with a few general issues. A brief description of the programs included in (and used by) the PEAT package is given in **2: Distributed Programs**. Finally, some of the common technical issues are considered in **3: Appendices**.

There is also load of modules and functionality that is eluded to but not really described here in much detail. If any of these interest you and you want to know more, or encourage development into fully functional programs, again let me know.

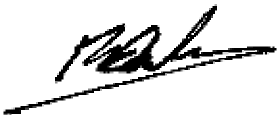

Rich Edwards, 2007.

1.1: Last Edit

This manual was last edited on 27 July 2007. While every effort has been made to ensure that all details here are correct as of this date, please report any errata.

1.2: Using this Manual

As much as possible, I shall try to make a clear distinction between explanatory text (this) and text to be typed at the command-prompt etc. Command prompt text will be `written in Courier New` to make the distinction clearer. Program options, also called 'command-line parameters', will be **`written in bold Courier New`** (and coloured **`red`** for fixed portions or **`dark red`** for user-defined portions, such as file names etc.). Command-line examples will be given in (purple) *`italicised Courier New`*. Optional parameters will (where I remember) be `[`in square brackets`]`. Names of files will be marked in normal text by (dark yellow) **Bold Times New Roman**.

1.3: Getting More Help

For detailed information on any given program, please refer to the specific manuals and/or websites accessible from my website (http://www.bioinformatics.rcsi.ie/~redwards/). There is also some documentation in the Python modules themselves. A full list of command-line parameters can be printed to screen using the **`help`** option, with short descriptions for each one.

```
python xxx.py help
```

If none of the above are of help, then please e-mail me (richard.edwards@ucd.ie) whatever question you have. If it is the result of an error message, then please send me that and/or the log file (see **3.6: Log Files**) too. Usually, it will be a problem with the input files (possibly formatting) but there are probably still a few bugs in there somewhere too.

1.4: Installation

All PEAT programs are a number of open source Python modules. They should therefore work on any system with Python installed without any extra setup required – simply copy the relevant files to your computer and run the program as described in the relevant manual. For ease of running from the command-line, it is recommended that you unzip the **peat_py.zip** into a directory near the root, e.g. **c:\bioware**. The zip will then extract into a directory called **peat**. (If you have downloaded a separate download of a specific program, this will extract into a directory named after that program.)

If you do not have Python, you can download it free from www.python.org at http://www.python.org/download/. The modules are written in Python 2.4. The Python website has good information about how to download and install Python but if you have any problems, please get in touch and I will help if I can.

1.4.1: Files Required

A full list of the Python Modules included in the PEAT download, and what functions they cover, is given in **3.2: Distributed Python Modules**. Some of the programs here also need additional input files (e.g. a PAM matrix) and/or use additional software (e.g. ClustalW) as described in **3.3: Additional Files Required** and **3.4: External Components of PEAT Programs** respectively.

If you download a specific program rather than the whole PEAT package and it fails to work because a file is missing, please contact me and/or download the package. This shouldn't happen!

1.5: Citations

When citing different elements of the PEAT package please follow the instructions given in the relevant Manuals/websites or in the appropriate part of **2: Distributed Programs**. Where external programs are called by PEAT programs, this will be indicated and suggested references given. Please also cite these programs.

2: Distributed Programs

Not all of the programs distributed with PEAT have standalone functionality. This is a list of those that do, with a brief description of the main functions of the program and the current status of documentation etc. in the following subsections. More information can be found in the relevant manuals and websites. If a program you want to use or know more about is lacking documentation, then please contact me and I shall accelerate the process!

NB. This list is incomplete but (should be) being constantly updated!

Program	Description	Manual?	Website?	Server?
BADASP	Prediction of residues conferring functional specificity in protein sequences	No	Yes	No
CompariMotif	Motif vs Motif comparisons	Yes	Yes	Yes
GASP	Ancestral sequence prediction for proteins	No	Yes	No
GABLAM	BLAST results compiler & summary tool	Yes	Yes	No
GOPHER	Protein orthologue prediction	Yes	Yes	Yes
HAQESAC	Quality protein alignment and tree generator	Yes	Yes	No
PRESTO	Peptide/Motif searches against protein databases	Yes	Yes	No
SeqMapper	Simple protein sequence mapping utility	No	Yes	No
SLiMFinder	*Ab initio* Short Linear Motif Finder	Yes	Yes	No

2.1: BADASP

Module: badasp.py

Description: BADASP uses ancestral sequence prediction from GASP (Edwards & Shields 2004) to identify large post-duplication changes in amino acid properties that have subsequently been conserved in protein subfamilies.

Website: http://bioinformatics.ucd.ie/shields/software/badasp/.

Webserver: Coming soon!

Cite: Edwards RJ & Shields DC (2005): BADASP: predicting functional specificity in protein families using ancestral sequences. *Bioinformatics* **21(22):**4190-1.

2.2: CompariMotif

Module: comparimotif.py

Description: CompariMotif is a piece of software with a single objective: to take two lists of protein motifs and compare them to each other, identifying which motifs have some degree of overlap, and identifying the relationships between those motifs. It can be used to compare a list of motifs with themselves, their reversed selves, or a list of previously

published motifs, for example (e.g. ELM (Puntervoll et al. 2003)). CompariMotif outputs a table of all pairs of matching motifs, along with their degree of similarity (information content) and their relationship to each other.

Website: http://bioinformatics.ucd.ie/shields/software/comparimotif/.

Webserver: http://bioware.ucd.ie/~comparimotif/.

Cite: Davey NE, Edwards RJ, Shields DC (2007): The SLiMDisc server: short, linear motif discovery in proteins. *Nucleic Acids Res.* **35(Web Server issue):**W455-9..

2.3: GASP

Module: gasp.py

Description: GASP uses a simple probabilistic framework to generate ancestral sequence predictions for proteins, including gapped positions in the alignment.

Website: http://bioinformatics.ucd.ie/shields/software/gasp/.

Webserver: Coming soon!

Cite: Edwards RJ & Shields DC (2004): GASP: Gapped Ancestral Sequence Prediction for proteins. *BMC Bioinformatics* **5(1):**123.

2.4: GABLAM

Module: gablam.py

Description: GABLAM performs an all-by-all BLAST (Altschul et al. 1990) and generates easily digestible summary tables based on percentage identity. GABLAM conversions of BLAST results are also used in a number of other programs, including SLiMDisc (Davey et al. 2006), SLiMFinder and GOPHER.

Website: http://bioinformatics.ucd.ie/shields/software/gablam/.

Webserver: Coming soon!

Cite: Davey NE, Shields DC & Edwards RJ (2006): SLiMDisc: short, linear motif discovery, correcting for common evolutionary descent. *Nucleic Acids Res.* **34(12):**3546-54.

2.5: GOPHER

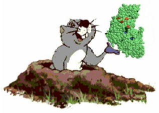

Module: gopher.py

Description: GOPHER uses GABLAM treatments of BLAST (Altschul et al. 1990) to identify putative orthologues from a protein database, and generate alignments.

Website: http://bioinformatics.ucd.ie/shields/software/gopher/.

Webserver: http://bioware.ucd.ie/~gopher/.

Cite: Please cite the website.

2.6: HAQESAC

Module: haqesac.py

Description: HAQESAC is designed for generating high quality alignments of proteins closely related to a given query protein, partitioning data into subfamilies and removing redundancy where desired. HAQESAC uses MUSCLE (Edgar 2004) or CLUSTALW (Higgins & Sharp 1988) for generate alignments, and CLUSTALW (Higgins & Sharp 1988) or PHYLIP (Felsenstein 2005) for generating phylogenetic trees. Ancestral sequences are predicted using BADASP (Edwards & Shields 2005).

Website: http://bioinformatics.ucd.ie/shields/software/haqesac/.

Webserver: None.

Cite: Edwards RJ, Moran N, Devocelle M, Kiernan A, Meade G, Signac W, Foy M, Park SDE, Dunne E, Kenny D & Shields DC (2007): Bioinformatic discovery of novel bioactive peptides. *Nature Chem. Biol.* **3(2):**108-112.

2.7: PRESTO

Module: presto.py

Description: PRESTO is a search tool for searching proteins with peptide sequences or motifs using an algorithm based on Regular Expressions. The simple input and output formats and ease of use on local databases make PRESTO a useful alternative to web resources for high throughput studies. Additionally, if you are interested in the conservation of a given motif, PRESTO can be given alignment files from which to calculate conservation statistics during the search and map motif occurrences onto. PRESTO also has the capability to calculate estimated values for Surface Accessibility, Hydrophobicity, and Disorder.

Website: http://bioinformatics.ucd.ie/shields/software/presto/.

Webserver: Coming soon!

Cite: Please cite the website.

2.8: SeqMapper

Module: seqmapper.py

Description: SeqMapper is a simple application based on GABLAM for mapping protein sequence from one dataset onto another, and replacing mapped sequences where desired. This could be used for mapping sequences onto EnsEMBL or UniProt, for example, or converting mouse proteins to their closest human orthologues.

Website: http://bioinformatics.ucd.ie/shields/software/seqmapper/.

Webserver: None.

Cite: Please cite the website.

2.9: SLiMFinder

Module: **slimfinder.py**

Description: SLiMFinder is a tool for finding over-represented protein motifs shared by unrelated proteins.

Website: http://bioinformatics.ucd.ie/shields/software/slimfinder/.

Webserver: Coming soon!

Cite: Paper in preparation. Please cite the website for now.

3: Appendices

3.1: Command-line Options

3.1.1: How to Use this Section

This section lists the general Command-line options that are used in multiple programs and may not be listed in their individual documentation. These form part of the parent RJE_Object class inherited by all PEAT classes. Default values are given [in square brackets]. This information is also available by printing the `__doc__` attribute of the **rje.py** module at a Python prompt, or using the **help** option:

print rje.__doc__ (in Python)

python rje.py help (commandline)

Please contact me if you want any further details of a specific option and/or advice as to when (not) to use it.

3.1.2: Option Types

There are essentially three types of command-line option:

1. Those that require a value (numerical or text), **option=X**. Those that require a filename as the value will be witten: **option=FILE**. Those that require a directory path as the value will be witten: **option=PATH**. Those that lead to an accessory application (rather than just its path) may also be listed as **option=COMMAND**. Paths and filenames should always use forward slash (/) separators, whatever the operating system.
2. True/False (On/Off) options, **option=T/F**. For these options:
   a. **option=F** and **option=False** are the same and turn the option off.
   b. **option**, **option=T** and **option=True** are the same and turn the option on.
3. List options. These are like the value options but have multiple values, separated by commas: **option=X,Y**. Where **..** is used, the number elements is optional, e.g. **option=X,Y,..,Z** could take **option=X** or **option=A,B,C,D**. Where **option=LIST** is used, the number of elements is optional and **LIST** could actually be the name of a file containing the list of elements.

3.1.3: INI Files

As well as feeding commands in on the command-line, any options listed can also be save in a plain text file and called using the option **ini=FILE**. Automatically, the program will read in any options from the file named after the program (e.g. **haqesac.ini**) and **rje.ini**, if present.

3.1.4: Setting up the INI File

It is recommended that a **rje.ini** file is made and placed in the same directory as the programs. This file should contain the paths to the programs listed in **3.4: External Programs Used by PEAT Programs**:

blastpath=PATH

fastapath=PATH

clustalw=COMMAND

muscle=COMMAND

Note that the first two are just paths to the programs, while for ClustalW and MUSCLE the actual program commands themselves must be included. This is to make it easier to replace these programs with alternatives. (See **3.5: Replacing Components with Other Programs**.)

If running in windows, it is also advisable to add the **win32=T** command to the ***.ini** file.

NB. For **PATH** variables, directories should be separated by a forward slash (/). If paths contain spaces, they should be enclosed in double quotes: **path="example path"**. It is recommended that paths do not contain spaces as function cannot be guaranteed if they do.

3.1.5: Interactivity and Verbosity settings

By default, the programs are generally setup to run through to completion without any user-interaction if given all the options it needs. For more interaction with the program as it runs, use the argument '**i=1**'.

python xxx.py commandlist i=1

Both the level of interactivity and the amount printed to screen can be altered, using the interactivity [**i=X**] and verbosity [**v=X**] command-line options, respectively, where **X** is the level from none (-1) to lots (2+). Although in theory **i=-1** and **v=-1** will ask for nothing and show nothing, there is a good chance that some print statements will have escaped in these early versions of the program. There is also the possibility that accessory programs may print things to the screen beyond the control of the calling program.

Please report any irritations and suggestions for changes to what is printed at different verbosity levels.

3.1.6: Option Precedence

Later options will supersede earlier ones if they are mutually exclusive. Options from an ini file will be inserted into the list at the point the ini file is called. (At the start for **rje.ini**.) This means that ini file options can be over-ruled, e.g.

xxx.py ini=eg.ini i=1 will supersede any interactivity setting in **eg.ini** with **i=1**.

xxx.py i=1 ini=eg.ini will use any interactivity setting in **eg.ini** and over-rule **i=1**.

3.1.7: General Command-line Options

The table below contains the most common command-line options used in PEAT programs. The Module column gives some information on which programs/modules are affected by the command:

- all = all modules use this command
- most = most of the programs in PEAT will use this command
- limited = only a few programs in PEAT use this command

To be safe, you should refer to the specific program documentation, especially if the program behaves counter to expectation.

Option	Description	Default	Module
	**General Input/Output Options**		
`v=X`	Sets verbosity (-1 for silent)	[0]	all
`i=X`	Sets interactivity (-1 for full auto)	[0]	all
`log=FILE`	Redirect log to FILE	[program.log]	all
`newlog=T/F`	Create new log file.	[False]	all
`help`	Prints help documentation to screen.	[False]	all
`basefile=FILE`	This will set the 'root' filename for output files (FILE.*), including the log	[None]	limited
`outfile=FILE`	This will set the 'root' filename for output files (FILE.*), excluding the log.	[None]	limited
`append=T/F`	Append to results files rather than overwrite.	[False]	most
`mysql=T/F`	"MySQL output" with lowercase headers that lack spacers.	[False]	most
`delimit=X`	Sets standard delimiter for results output files.	[varies]	most
`force=T/F`	Force to regenerate data rather than keep old results.	[False]	limited
	**System Info**		
`win32=T/F`	Run in Win32 Mode	[False]	all
`memsaver=T/F`	Run in "Memory Saver" mode	[False]	limited
	**Forking**		
`forks=X`	Number of forks	[0]	limited
`killforks=X`	Number of seconds of inactivity before killing forks	[3600]	limited
`noforks=T/F`	Option to over-ride and cancel forking	[False]	limited

3.2: Distributed Python Modules

This appendix is liable to be out of date. The **Exec** column indicates whether the module has standalone functionality. Those with an asterisk have additional documentation (manuals and/or websites) of their own. For other modules, please run with the `help` option for more details or see the distributed **readme.txt** or **readme.html** files.

The Python Modules are open source and may be changed if desired, although please give me credit for any useful bits you pillage. I cannot accept any responsibility if you make changes and the program stops working, however!

Note that the organisation of the modules and the complexity of some of the classes is due to the fact that most of them are designed to be used in a number of different tools. As a result, not all the options listed in the `__doc__()` (`help`) will be of relevance. If you want some help understanding the way the modules and classes are set up so you can edit them, just contact me.

3.2.1: Main Programs

These are the main programs that are called using the command-line and (should) have reasonable documentation including websites and manuals. See also **2: Distributed Programs** for more details.

Module	Description	Classes	Exec
**badasp**	This module contains the main BADASP method, which calls the relevant methods from the other modules and handles results output.	-	Yes*
**comparimotif**	Master module for CompariMotif program.	CompariMotif	Yes*
**compass**	Compares UniProt annotation and motif prediction etc. across homologous proteins.	Compass	Yes*?
**gablam**	Main GABLAM module containing primary code	BAM	Yes*
**gasp**	Main GASP control module.	-	Yes*
**gopher**	Main GOPHER module containing primary code	Gopher, GopherFork	Yes*
**haqesac**	Master module for HAQESAC program. Controls main thread and objects.	HAQESAC	Yes*
**presto**	Main PRESTO module containing primary code	Presto, PrestoSeqHit, PrestoHit	Yes*
**slim_pickings**	SLiMDisc results compiler and manipulator.	SlimPicker	Yes*

3.2.2: Main Accessory Applications

As well as the main applications, there are a number of subsidiary applications that, in addition to providing functional classes and methods to the above programs, have worthwhile functionality by themselves. Documentation for most of these is more limited but I am happy to tell people how to use any that sound of interest.

Module	Description	Classes	Exec
rje_blast	Performs BLAST searches and loads results into objects. Performs GABLAM calculations.	BLASTRun, BLASTSearch, BLASTHit, PWAln	Yes
rje_dbase	Module to Handle Database manipulations and generation of species-specific databases etc. Includes indexing of local UniProt for use with rje_uniprot.	DatabaseControll er	Yes
rje_mysql	Module for converting delimited text files to MySQL tables	MySQL, Table, Field	Yes
rje_pam	This module handles functions associated with PAM matrices. A PAM1 matrix is read from the given input file and multiplied by itself to give PAM matrices corresponding to greater evolutionary distance. (PAM1 equates to one amino acid substitution per 100aa of sequence.)	PamCtrl, PAM	Yes
rje_pattern_ discovery	Can be used for batch motif discovery with SLiMDisc and TEIRESIAS.	PatternDiscovery, Pattern	Yes
rje_pydocs	Extracts documentation and generates a table of method links for python modules.	PyDoc, PyModule, PyClass, PyMethod	Yes
rje_seq	Contains Classes and methods for sets of DNA and protein sequences. (Currently only protein sequences supported.)	SeqList, Sequence, DisMatrix	Yes*
rje_tree	Reads in, edits and outputs phylogenetic trees. Executes duplication and subfamily determination.	Tree, Node, Branch	Yes*
rje_uniprot	Contains methods for parsing UniProt info. Indexing performed by rje_dbase.py.	UniProt	Yes

3.2.3: Accessory Modules

Modules not for standalone running but with classes and methods used by main programs.

Module	Description	Classes	Exec
rje	General module containing Classes used by all my scripts plus a number of miscellaneous methods.	RJE_Object_Shell, RJE_Object, Info, Out, Log	No
rje_aaprop	This module Takes an amino acid property matrix file and reads into an AAPropMatrix object. Converts in an all by all property difference matrix. By default, gaps and Xs will be given null properties (None) unless part of input file.	AAPropMatrix	No
rje_ancseq	This module contains the objects and methods for ancestral sequence prediction. Currently, only GASP (Edwards & Shields 2004) is implemented. Other methods may be incorporated in the future.	Gasp, GaspNode	No
rje_conseq	Contains objects for calculating conservation stats used in BADASP.	SeqStat	No
rje_dismatrix	Contains Classes and methods for Distance Matrix	DisMatrix	No
rje_haq	Performs actual HAQ (SAQ and PAQ) algorithms.	HAQ	No
rje_menu	Contains methods for standard menu operations.	-	No
rje_motif	This module handles functions associated with Motif class along with reformatting motifs and storing variants etc.	Motif	No
rje_motif_cons	Contains methods for motif conservation scoring	-	No
rje_sequence	Contains Classes and methods for individual sequences	Sequence	No
rje_specificity	Contains objects for calculating specificity stats used in BADASP.	FuncSpec	No
rje_tree_group	Contains all the Grouping Methods for **rje_tree.py**	-	No
rje_xml	Crude XML parsing module	XML	No

3.2.4: Miscellaneous Additional Accessory Applications

These guys are not directly important for the main applications but may be of some use to someone, somewhere. See documentation in the modules themselves or e-mail me for more information.

Module	Description	Classes	Exec
**file_monster**	File and directory information extraction and summary module.	FileScout	Yes
**peptide_dismatrix**	Generates distance matrices for short peptides.	PepDis	Yes
**peptide_stats**	Generates table of statistics about input peptides (charge balance etc.)	PepStats	Yes
**pic_html**	Generates a set of linked websites from pictures in correct directory structure.	picHTML	Yes
**rem_parser**	Parses details of removed sequences from log files.	RemParser	Yes
**rje_hmm**	Performs HMM searches with HMMer	HMMRun, HMMSearch, HMMHit	Yes
**rje_markov**	Messes about with markov chains in proteins, making frequency tables etc.	Markov	Yes
**rje_seqgen**	Random sequence generation and peptide scrambling.	SeqGen	Yes
**rje_scansite**	Converts multiple scansite output files into a single table.	Scansite	Yes
**rje_tm**	Compiles TMHMM and SignalP results.	TM	Yes
**seqforker**	Splits a large dataset up, forks out processes and then sticks it back together. Designed for use with (almost) any other application.	SeqForker	Yes

3.3: Additional Files Required

The following files are required for some programs in the package to run correctly. All these files should have been provided in the download zip file. These files may all be replaced with other files in the correct format. Programs will look first in the directory from which the program is called, and then in the directory specified by **path=PATH**, which is the directory containing the Python scripts by default.

3.3.1: Amino Acid Property Matrix

By default, a property matrix based on that used by Livingstone and Barton is used (**aaprop.txt**)(Livingstone & Barton 1993). This is file can be replaced by one in the same format using the **aaprop=FILE** option. This file may have different (numbers of) properties than those used in the default file:

```
# Based on Property Matrix of Livingstone and Barton.
PROPERTY     I L V C A G M F Y W H K R E Q D N S T P
Hydrophobic  1 1 1 1 1 1 1 1 1 1 1 1 0 0 0 0 0 0 1 0
Polar        0 0 0 0 0 0 0 0 1 1 1 1 1 1 1 1 1 1 1 0
Small        0 0 1 1 1 1 0 0 0 0 0 0 0 0 0 1 1 1 1 1
Proline      0 0 0 0 0 0 0 0 0 0 0 0 0 0 0 0 0 0 0 1
Positive     0 0 0 0 0 0 0 0 0 0 1 1 1 0 0 0 0 0 0 0
Negative     0 0 0 0 0 0 0 0 0 0 0 0 0 1 0 1 0 0 0 0
Charged      0 0 0 0 0 0 0 0 0 0 1 1 1 1 0 1 0 0 0 0
Tiny         0 0 0 0 1 1 0 0 0 0 0 0 0 0 0 0 0 1 0 0
Aliphatic    1 1 1 0 0 0 0 0 0 0 0 0 0 0 0 0 0 0 0 0
Aromatic     0 0 0 0 0 0 0 1 1 1 1 0 0 0 0 0 0 0 0 0
```

This property matrix is then converted by BADASP into a property difference matrix using the **rje_aaprop.py** module.

Currently gaps are handled using a special parameter **aagapdif=X**, which assigns all comparisons between a gap and another residue (including another gap) a value of **X**. In future, gaps may be incorporated into input property matrices if desired. Please contact the author to have this implemented sooner. By default this difference is 5, which is half of the default number of properties. This parameter is independent of property number, however, and can be given a value in excess of the number of properties if it is desirable to make gapped regions extremely different from ungapped ones.

Wildcard residues such as X are handled using a special parameter **aanulldif=X**, which assigns all a set difference for each property. By default this is 0.5, so a wildcard/unknown residue will share half its properties with any other residue. A value of 0 will give no property differences, while 1 will assume all properties are different.

3.3.2: PAM Matrix File

The PAM matrix contains amino acid substitution probabilities. A basic PAM matrix (Jones et al. 1992) is available in the file **jones.pam** (and is known as JTT, I believe). The important part of this file is the top section, which has the single letter amino acid codes on the first line, followed by the PAM1 matrix, where each subsequent line consists of an amino acid code and the probability of that aa being substituted by each other aa, in the order given in the first line:

```
A   R   N   D   C   Q   E   G   H   I   L   K   M   F   P   S   T   W   Y   V
Ala 0.98754 0.00030 0.00023 0.00042 0.00011 0.00023 0.00065 …
Arg 0.00044 0.98974 0.00019 0.00008 0.00022 0.00125 0.00018 …
Asn 0.00042 0.00023 0.98720 0.00269 0.00007 0.00035 0.00036 …
```

```
…
Val 0.00226 0.00009 0.00007 0.00016 0.00012 0.00008 0.00027 …
```

Note that the amino acids must be in the same order in both columns and rows. See http://bioinformatics.ucd.ie/shields/software/gasp/gasp_pam.htm for more details.

3.3.3: Using an unscaled matrix

An unscaled matrix, such as the WAG matrix provided by the Goldman group at www.ebi.ac.uk/goldman/WAG can now also be used by **rje_pam** and converted into a PAM1 matrix. To do this, use the **altpam=FILE** option. By default, amino acid frequencies will be read from that file. If the **seqin=FILE** command is also used, the amino acid frequencies will be calculated from that sequence file instead. The **pamout=FILE** option specifies the name for the rescaled PAM matrix output. (By default this file is named after the input file with a ***.pam** extension.)

3.4: External Components of PEAT Programs

In addition to the python modules listed above, some of the programs make use of the following published programs. These are freely available for downloading and installing. It is recommended that the user downloads and installs these programs according to the instructions given on the appropriate website.

ALIGN: This is part of the Fasta package (Pearson 1994; 2000) and can be downloaded from the University of Virginia: http://fasta.bioch.virginia.edu/. Make sure that align is part of the download. For some reason it seems to have been dropped from later packages. You may need to install an earlier package first (e.g. 2.1) and then a later package.

BLAST: BLAST (Altschul et al. 1990) is freely available for download from NCBI at: http://www.ncbi.nlm.nih.gov/blast/download.shtml.

CLUSTALW: ClustalW (Higgins & Sharp 1988; Thompson *et al.* 1994) is an old stalwart for bioinformatics and is freely available from EMBL: ftp://ftp-igbmc.u-strasbg.fr/pub/ClustalW/. Note that CLUSTALW is used as a backup for MUSCLE (below) and to draw trees. See **Replacing Components with Other Programs** for details of how to incorporate other tree-drawing packages.

MUSCLE: MUSCLE (Edgar 2004) is a newer multiple alignment program available from http://www.drive5.com/muscle.

PHYLIP: Details coming soon!

TEIRESIAS: See SLiMDisc documentation for details.

It is recommended that paths to these programs are placed into an **INI** file (see **3.1.4: Setting up the INI File**).

3.5: Replacing Components with Other Programs

The most important functions performed by the external programs alignment and tree-drawing. This section lists some ways to incorporate alternative programs for these functions into PEAT programs. I am always interested to add more functionality, so if there is a program you would like to use instead of those listed, then please contact me and I may be able to add them in a more controlled fashion than below.

3.5.1: Alignment programs

By default, MUSCLE (Edgar 2004) is used for alignments as I have found this to be both fast and accurate. There can be problems with memory allocation for larger datasets and so and ClustalW (Higgins & Sharp 1988; Thompson *et al.* 1994) is used for large datasets above a certain total number of residues (as determined by the **cwcut=X** parameter). Either of these programs can be replaced, however, by another program that uses the same command-line format call the programs.

For MUSCLE, the system call is:

`muscle` **-in INFILE -out OUTFILE**, where **INFILE** and **OUTFILE** are both fasta format.

The path to MUSCLE can be changed to redirect to another program using the **muscle=PATH** option.

For ClustalW, the system call is:

`clustalw` **INFILE**, where **INFILE** is in fasta format (***.fas**) and the output file (***.aln**) is in ClustalW align format.

The path to ClustalW can be changed to redirect to another program using the **clustalw=PATH** option.

3.5.2: Tree-drawing programs

The default for PEAT programs is to use the Neighbour-joining method implemented in ClustalW for drawing trees. Although this is not the most accurate phylogeny construction algorithm around, it is fast and efficient and reasonable for trees of closely-related sequences with high bootstrap support, such as those HAQESAC was designed to build and work with. Again, this program can be replaced with another using the **maketree=PATH** option. The system call used is:

`clustalw` **-infile=INFILE -bootstrap=X -seed=X [-kimura]** for UNIX, or

`clustalw` **INFILE -bootstrap=X -seed=X [-kimura]** for Windows, where **INFILE** is in fasta format (***.fas**) and the output file (***.phb**) is in bootstrapped Phylip format (I think).

It *should* work to have a program output a Newick Standard Format tree as ***.nsf** but I have not tested that.

Phylip tree-drawing is also implemented. See module documentation for details. #!# Describe more! #!#

3.5.3: Wrapper scripts

If the chosen program does not accept the same input/output commands/formats then a wrapper script should be written. It is suggested to use Perl or Python for this. Although I cannot promise help in every suggestion, you are welcome to e-mail me for help with this and I will see what I can do.

3.5.4: Incorporating Other Programs into the Python Code

If you are feeling brave, you can actually edit the Python modules themselves. The key methods for this are **rje_seq.muscleAln()**, **rje_seq.clustalAln()** and **rje_tree.makeTree()**. Obviously, I cannot promise to give technical support for any changes that are made but, if you know what you are doing, you should be OK and I will help where I can.

3.6: Log Files

Every program generates a log file when it is run. By default, this file will be named after the calling program (e.g. **gasp.py** will produce a log called **gasp.log**) but this can be changed with the **log=FILE** option. Logs will be appended unless the **newlog** (or **newlog=T**) option is used.

The log file records information that may help subsequent interpretation of results or identify problems. Probably it's most useful content is any error messages generated, which are marked by a #ERR line header. Other information is also recorded along with the runtime (HH:MM:SS since the program started).

For help interpreting log files, please check the relevant software manual or contact me if the information is missing. (Hopefully, the log content is mostly self-explanatory but I shall add any explanations I have to send people to the relevant manual's appendix.)

3.7: Troubleshooting

Currently, this is a small section as I have not had enough feedback to have FAQs, or anything like that. Here is a list of things that I think MAY cause problems to the unwary:

- Giving file names with spaces without enclosing in double quotes "". (Only the first word will be taken as the filename.) It is not recommended to have spaces in filenames as some programs (and accessory programs) may go wrong if you do.
- Including spaces in paths to programs etc. without double quotes "". Likewise, a lack of spaces altogether is favourable.
- Incorrect formatting of input files. Check instructions in relevant manual. If things are still not working, check that the line breaks are \n and not \r or some other odd format.
- I'm not sure when but there is a possibility of problems if running in Windows without the **win32=T** option.
- Using out-of-date modules. Sometimes changes to a program will actually be made in one of the modules they import: if upgrading, upgrade all modules and not just the program being called.

3.8: References

Altschul SF *et al.* (1990). Basic local alignment search tool. *J Mol Biol* **215**: 403-10.

Davey NE, Shields DC and Edwards RJ (2006). SLiMDisc: short, linear motif discovery, correcting for common evolutionary descent. *Nucleic Acids Res.* **34**: 3546-54.

Edgar RC (2004). MUSCLE: a multiple sequence alignment method with reduced time and space complexity. *BMC Bioinformatics* **5**: 113.

Edwards RJ and Shields DC (2004). GASP: Gapped Ancestral Sequence Prediction for proteins. *BMC Bioinformatics* **5**: 123.

Edwards RJ and Shields DC (2005). BADASP: predicting functional specificity in protein families using ancestral sequences. *Bioinformatics* **13**: 13.

Felsenstein J (2005). PHYLIP (Phylogeny Inference Package) version 3.6. *Distributed by the author. Department of Genome Sciences, University of Washington, Seattle.*

Higgins DG and Sharp PM (1988). CLUSTAL: a package for performing multiple sequence alignment on a microcomputer. *Gene* **73**: 237-44.

Jones DT, Taylor WR and Thornton JM (1992). The rapid generation of mutation data matrices from protein sequences. *Comput Appl Biosci* **8**: 275-82.

Livingstone CD and Barton GJ (1993). Protein sequence alignments: a strategy for the hierarchical analysis of residue conservation. *Comput Appl Biosci* **9**: 745-56.

Pearson WR (1994). Using the FASTA program to search protein and DNA sequence databases. *Methods Mol Biol* **24**: 307-31.

Pearson WR (2000). Flexible sequence similarity searching with the FASTA3 program package. *Methods Mol Biol* **132**: 185-219.

Puntervoll P *et al.* (2003). ELM server: A new resource for investigating short functional sites in modular eukaryotic proteins. *Nucleic Acids Res* **31**: 3625-30.

Thompson JD, Higgins DG and Gibson TJ (1994). CLUSTAL W: improving the sensitivity of progressive multiple sequence alignment through sequence weighting, position-specific gap penalties and weight matrix choice. *Nucleic Acids Res* **22**: 4673-80.
