# Supplementary material for: Estimation and efficient computation of the true probability of recurrence of short linear protein sequence motifs in unrelated proteins
Source: BMC Bioinformatics. 2010 Jan 7;11:14. doi: 10.1186/1471-2105-11-14 (PMC2819990; doi:10.1186/1471-2105-11-14)
Supplement: Additional file 1 — Slimsuite software package. This ZIP file contains the slimsuite software package used with this submission. [file 1471-2105-11-14-S1.ZIP › slimsuite/PRESTO Manual.pdf]

PRESTO Protein Regular Expression Search Tool

Richard J. Edwards (2006)

1: Introduction 3
1.1: Version 3
1.2: Using this Manual 3
1.3: Getting Help 3
1.4: Why use PRESTO? 4
1.5: Availability and Local Installation 4
2: Fundamentals 5
2.1: Running PRESTO 5
2.1.1: The Basics 5
2.1.2: Interactivity and Verbosity settings 5
2.1.3: Other Options 5
2.2: Input 6
2.2.1: Motif Input 6
2.2.2: Motif Filtering options 6
2.2.3: Searching with Mismatches 7
2.2.4: Search Database 7
2.2.5: Optional Input 1: Multiple Sequence Alignments 7
2.2.6: Option Input 2: Taxonomic subgroupings 8
2.3: Output 9
2.3.1: Basic Output 9
2.3.2: Information Content 9
2.3.3: Expected Occurrence Statistics 9
2.3.4: Additional Taxonomic Conservation Output 10
2.3.5: Filtering results using PRESTO Statistics 10
2.3.6: Optional Motif Information File 11
2.3.7: Log Files 11
2.4: Information Content 11
3: Conservation Statistics 12
3.1: Conservation Output 12
3.2: Conservation Scores 12
3.2.1: Absolute Conservation [conscore=abs] 12
3.2.2: Positional Scoring [conscore=pos] 13
3.2.3: AA Property Scoring [conscore=prop] 13
3.2.4: Combined Scoring [conscore=all] 13
3.2.5: Additional output: conservation score used 14
3.3: Additional Options 14
3.3.1: Motif ambiguity 14
3.3.2: Positional Weighting by Information Content 14
3.3.3: Homology Weighting 14
3.3.4: Gap Treatment 15
3.4: Generating Alignments with GOPHER 15
4: Appendices 16
4.1: Appendix I: Command-line Options 16

4.1.1: Forking Options..16
4.2: Appendix II: Distributed Python Modules 16
4.3: Appendix III: Log Files...16
4.4: Appendix IV: Troubleshooting...16
4.5: Appendix V: References...16

1: Introduction

Software manuals are boring: boring to write and probably even more boring to read. I have therefore tried to keep this one concise. However, there is a good chance that the pleiotropic affect of this is a lack of clarity and/or coherence. For this I apologise, and encourage anyone out there to send in errata and/or suggested improvements. The fundamentals should be covered in **2: Fundamentals**, including **2.2: Input** and **2.3: Output**. More details can be found in later sections and gluttons for punishment can get even more information in **4: Appendices** and the accompanying **PEAT Appendices** documentation.

Like the software itself, this manual is a 'work in progress' to some degree. If the version you are now reading does not make sense, then it may be worth checking the website to see if a more recent version is available, as indicated by the **1.1 Version** section of the manual. Good luck.

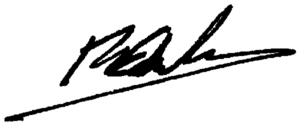

Rich Edwards, 2006.

1.1: Version

This manual is designed to accompany PRESTO version 3.2.

The manual was last edited on 02 October 2006.

1.2: Using this Manual

As much as possible, I shall try to make a clear distinction between explanatory text (this) and text to be typed at the command-prompt etc. Command prompt text will be `written in Courier New` to make the distinction clearer. Program options, also called 'command-line parameters', will be **`written in bold Courier New`** (and coloured **`red`** for fixed portions or **`dark red`** for user-defined portions, such as file names etc.). Command-line examples will be given in (purple) *`italicised Courier New`*. Optional parameters will (where I remember) be `[`in square brackets`]`. Names of files will be marked in normal text by (dark yellow) **Bold Times New Roman**.

1.3: Getting Help

Much of the information here is also contained in the PRESTO website (http://bioinformatics.ucd.ie/shields/software/presto/) and the documentation of the Python modules themselves. A full list of command-line parameters can be printed to screen using the **`help`** option, with short descriptions for each one.

`python presto.py` **`help`**

If none of the above help, then please e-mail me (richard.edwards@ucd.ie) whatever question you have. If it is the results of an error message, then please send me that and/or the log file too. Usually, it will be a problem with the input files (possibly formatting) but there are probably still a few bugs in there somewhere too.

1.4: Why use PRESTO?

PRESTO is what the acronym suggests: a search tool for searching proteins for peptide sequences or motifs using an algorithm based on Regular Expressions. The simple input and output formats and ease of use on local databases make PRESTO a useful alternative to web resources for high throughput studies. Additionally, if you are interested in the conservation of a given motif, PRESTO can be given alignment files from which to calculate conservation statistics during the search.

1.5: Availability and Local Installation

PRESTO can be run from the PRESTO webserver, available at http://bioware.ucd.ie/~presto/.

PRESTO is also distributed as a number of open source Python modules as part of the PEAT (Protein Evolution Analysis Toolkit) package. It should therefore work on any system with Python installed without any extra setup required – simply copy the relevant files to your computer and run the program (see **2.1: Running PRESTO**, below).

If you do not have Python, you can download it free from www.python.org at http://www.python.org/download/. The modules are written in Python 2.4. The Python website has good information about how to download and install Python but if you have any problems, please get in touch and I will help if I can.

All the required files should have been provided in the download zip file. Details can be found at http://bioinformatics.ucd.ie/shields/software/peat/ and the accompanying **PEAT Appendices** document. The Python Modules are open source and may be changed if desired, although please give me credit for any useful bits you pillage. I cannot accept any responsibility if you make changes and the program stops working, however!

Note that the organisation of the modules and the complexity of some of the classes is due to the fact that most of them are designed to be used in a number of different tools. As a result, not all the options listed in the `__doc__()` (**help**) will be of relevance. If you want some help understanding the way the modules and classes are set up so you can edit them, just contact me.

2: Fundamentals

2.1: Running PRESTO

2.1.1: The Basics

If you have python installed on your system (see Error! Reference source not found.**Availability and Local Installation**), you should be able to run PRESTO directly from the command line in the form:

`python presto.py` **`searchdb=FILENAME motifs=FILENAME`**

For the example provided in the distribution:

`python presto.py searchdb=blastdb_eg.fas motifs=presto_eg.motifs`

PRESTO does not currently have a menu-driven implementation but, if running in Windows with an INI file (see below), you can just double-click the **presto.py** file to run.

IMPORTANT: If filenames contain spaces, they should be enclosed in double quotes: **`searchdb="example file"`**. That said, it is recommended that files do not contain spaces as function cannot be guaranteed if they do.

2.1.2: Interactivity and Verbosity settings

By default, PRESTO will run through to completion without any user-interaction if given all the options it needs. For more interaction with the program as it runs, use the argument '**`i=1`**'

`python presto.py searchdb=blastdb_eg.fas motifs=presto_eg.motifs i=1`

Both the level of interactivity and the amount printed to screen can be altered, using the interactivity [**`i=X`**] and verbosity [**`v=X`**] command-line options, respectively, where **`X`** is the level from none (-1) to lots (2+). Although in theory **`i=-1`** and **`v=-1`** will ask for nothing and show nothing, there is a good chance that some print statements will have escaped in these early versions of the program.

Please report any irritations and suggestions for changes to what is printed at different verbosity levels.

2.1.3: Other Options

At first, you will probably want to run the program with its default parameters. If you want to change them, there are a number of parameters that can be set by the user and other options. These are described in the relevant sections and summarised in **4.1: Command-line Options**. These may be given after the run command, as above, or loaded from one or more ***.ini** files (see for **PEAT Appendices** for details).

2.2: Input

The main input for PRESTO is a motif file and a fasta format search database. Optional input for conservation analyses can be given to PRESTO in the form of protein multiple sequence alignments.

2.2.1: Motif Input

The recommended motif input format is PRESTO format. This should have a single line per motif, with the format:

```
Name Sequence # Comments
```

Comments are optional but anything after the # will be ignored.

Alternative allowed formats include: a fasta format file with motif/peptide names and sequences in the usual fasta format; a raw list of peptides/motifs (in this case the name and sequence will the same); SLiMDisc output; TEIRESIAS output; Slim Pickings output. Additional input formats can be added on request

In either case, the motif should be a peptide sequence using the standard single letter amino acid codes and the following regular expression rules:

- **A** = single fixed amino acid.
- **[AB]** = ambiguity, **A** or **B**. Any number of options may be given, e.g. **[ABC]** = **A** or **B** or **C**.
- **<R:m:n>** = At least **m** of a stretch of **n** residues must match **R**, where **R** is one of the above regular expression elements (single or ambiguity).
- **[^A]** = not **A**.
- **X** or **.** = Wildcard positions (any amino acid)
- **R{n}** = **n** repetitions of **R**, where **R** is any of the above regular expression elements.
- **R{m,n}** = At least **m** and up to **n** repetitions of **R**.
- **(AB|CD)** = **AB** or **CD**. For MSMS peptides (**msms=T**), this is also **BA** or **DC**.
- **(ABC)** = **ABC** in any order (**BAC**, **CAB** etc.).
- **^** = Beginning of sequence
- **$** = End of sequence

E.g. **[IL][^P]X{3}RG** means: "leucine or isoleucine, followed by anything but proline, followed by three residues, followed by arginine followed by glycine".

E.g. (2) **^<KR:3:5>P** means: "three of the first five amino acids must be lysine or arginine; the sixth amino acid must be proline".

2.2.2: Motif Filtering options

As well as manually editing the input files, there are a number of options for filtering the motifs/peptides that PRESTO reads in. **minpep=X** and **minfix=X** will set the minimum number of non-wildcard and fixed positions that the motif must contain, respectively. Some motifs (or MSMS peptides) have leading or trailing wildcard positions. By default, these will be included in any search as the information on the up or downstream region may be desirable. (Because PRESTO returns the sequence of the matched region, these wildcards can be used to return the sequence surrounding motif occurrences and/or to better control

the region included in any hydrophobicity/disorder etc. calculations.) To remove these wildcard positions, use `trimx=F`.

Finally, motifs can be filtered out according to the expected number of occurrences in the search database (see **2.3.3: Expected Occurrence Statistics**) using the `expcut=X` option. If the expected number of occurrences exceeds this threshold, then the motif is removed from the search. This allows the user to restrict searches to the more unlikely motifs.

2.2.3: Searching with Mismatches

PRESTO allows motifs and peptides to be searched with a number of mismatches allowed. This is set by the `mismatch=X,Y` option, which can be used several times to setup "mismatch bands". This option allows `X` mismatches when the sequence has at least `Y` non-wildcard positions. *E.g.* *`mismatch=1,6 mismatch=2,10`* would allow one mismatch for every sequence with 6+ non-wildcard positions and two mismatches for every sequence with 10+ non-wildcard positions.

Mismatches are places in every possible non-wildcard position in the motif, including degenerate positions. In the case of (AB) type elements, all possible combinations are tried (AX,BX,XA,XB). Where repetitions are allowed, these are applied before any mismatches are placed. *E.g.* P{1,2} with one mismatch would be searched with P, PP, X, PX and XP.

NB. Searching with mismatches can dramatically increase the run-time. When searching large databases it is advisable to first perform a test run with a few sequences to calculate approximate runtimes.

2.2.4: Search Database

The search database for PRESTO should be a fasta file of protein sequences and is identified with the `searchdb=FILENAME` option. Fasta format is commonly used in bioinformatics applications and a variety of subformats will be recognised. To get the most out of the program, one of the set fasta formats from common sequence databases should be used. The included manual for the **rje_seq.py** sequence manipulation module has more details on formats and reformatting/filtering of input sequences.

2.2.5: Optional Input 1: Multiple Sequence Alignments

PRESTO is designed to be able to use the output of GOPHER (Edwards 2006) alignments of orthologues to calculate the conservation of a given motif (see **3: Conservation Statistics**). Alternative sources for theses alignments can be used, as long as the format is correct.

Alignments should be in FASTA format with descriptions on one line followed by one or more lines containing the sequence. All sequences should be of the same length. The first word in each description should be unique. *e.g.*

```
>Seq1 And its description
SEQUENCE-ONE-GOES-HERE
>Seq2
---GAPS--ARE--ALLOWED-
>Seq3
---BUT---ALL-SEQUENCES
>Seq4
MUST-BE-EQUAL--LENGTHS
```

The file should be named **AccNum.X**, where **AccNum** is the accession number of the relevant protein in the search database, and **X** is given by the command `alnext=X`. Files

should be found in a directory identified with the `alndir=PATH` command. This function can be switched on using the `usealn=T` option.

2.2.6: Option Input 2: Taxonomic subgroupings

In addition to the general conservation statistics produced for the given alignments, conservation calculations can be restricted to one or more taxonomic groups. This is achieved using the `conspec=LIST` option, where `LIST` is a list of files containing the UniProt species codes for the relevant grouping. Wildcards are allowed. Conservation analysis is then limited to these species and additional columns produced in the output (see below).

e.g. If only interested in the model organisms Human, Mouse, Rat, Chicken and Xenopus, one could use the command *conspec=model.spec_code* (or *conspec=*.spec_code*), where **model.spec_code** contains the species codes:

```
HUMAN
MOUSE
RAT
CHICK
XENLA
```

This would then produce additional output columns MODEL_CONS, MODEL_HOM, MODEL_GLOB_ID and MODEL_LOC_ID (see below). Where multiple files were given, each file would have its own set of output columns.

NB. The name all is reserved as a special key in PRESTO. Do *not* use *conspec=all.spec_code*.

2.3: Output

2.3.1: Basic Output

The main output for PRESTO is a delimited file with search results:

- MOTIF = Motif name as given in input file
- LEN = Length of motif
- VARIANT = Variant of motif (closest) matching sequence
- MATCHSEQ = Matched sequence in Query
- MATCH_ID = Identity between match and closest variant (See **2.2.3: Searching with Mismatches**)
- MOTIF_CONS = Percentage conservation across homologues (see below)
- HOM_NUM = Number of homologues compared
- GLOB_ID = Mean global percentage identity between query protein and homologues
- LOC_ID = Mean local percentage identity between query protein and homologues across region of match
- SA = Surface Accessibility prediction (*details to follow*)
- HYD = Eisenberg Hydrophobicity prediction (*details to follow*)
- HIT = Name or AccNum of hit sequence
- START_POS = Start Position of match in Hit
- END_POS = End position of match in Hit

2.3.2: Information Content

If `motific=T` then an additional information content "MOTIF_IC" statistic is output. This is calculated as described in **2.4: Information Content**.

2.3.3: Expected Occurrence Statistics

Unless the `expect=F` option is used, PRESTO will calculate an additional statistic:

- EXPECT = expected number of matches for that motif in the search database given its size and amino acid composition

This is a fairly simple calculation based on the frequency of each amino acid (f_a), the number of sequences (N_S) and the total number of amino acids (N_A). For each position in a motif, the probability of occurrence at any residue in the dataset (p_i) is simply the sum of the frequencies for the possible amino acids at that position:

$p_i = \Sigma f_a$

The probability of the whole motif starting at any residue (p_m) is therefore the product of p_i over all positions:

$p_m = \Pi p_i$

The expected number of occurrences for the motif in the dataset is therefore this number multiplied by the possible number of positions at which the motif could start (N_m), which in turn is the total number of amino acids in the dataset minus a number of positions per sequence dependent on the length of the motif (L):

$N_m = N_A - (N_S(L\text{-}1))$

EXPECT = $p_m N_m$

2.3.4: Additional Taxonomic Conservation Output

In addition to the columns above, for each file of taxonomic species codes given (see **2.2.6: Option Input 2: Taxonomic subgroupings**), four more columns will be output:

- X_CONS = Percentage conservation across reduced set of homologues (see below)
- X_HOM = Number of reduced homologues compared
- X_GLOB_ID = Mean global percentage identity between query protein and reduced set of homologues
- X_LOC_ID = Mean local percentage identity between query protein and reduced ser of homologues across region of match

Where X is the leading part of the filename containing the species codes. Where multiple files were given, each file would have its own set of output columns.

2.3.5: Filtering results using PRESTO Statistics

The **statfilter=LIST** command allows the user to filter results according to any statistic included in the output, including conservation scores etc. The **LIST** is in the form **"stat1>a,stat2<b,stat3=c,stat4!=d"** etc. and should either be a comma delimited list given on the commandline, or contained in a separate file (named LIST e.g. *statcut=my_statcut_list.txt*). If not a file name, enclose in double quotes or the <> symbols will try to pipe input/output! The alllowed operators are:

Operator	Description
**>**	Filtered if the stat exceeds the cut-off
**>=** or **=>**	Filtered if the stat equals or exceeds the cut-off
**<**	Filtered if the stat is lower than the cut-off
<= or =<	Filtered if the stat is lower than or equal to the cut-off
**=** or **==**	Filtered if the stat is equal to the cut-off
**!=** or **<>**	Filtered if the stat is not equal to the cut-off

All these may be applied to any stat, included text fields. Stat names should match the column headers of the output (case-insensitive). If a stat is given that is not recognised, PRESTO will report an error but continue processing without that stat cut-off. **Warning!** Applying > or < to strings should be used with caution, though Python does seem to process them consistently with alphabetical sorting.

2.3.6: Optional Motif Information File

In addition to the main output, the `motinfo=FILE` command will output a motif summary table into the specified filename. This file consists of the following fields:

Field	Description
Motif	The name of the motif.
Pattern	The regular expression pattern of the motif (see **2.2.1: Motif Input**).
Description	The description of the motif.
MaxLength	Maximum length of the motif in terms of non-wildcard positions.
MinLength	Minimum length of the motif in terms of non-wildcard positions.
FixLength	Maximum Length of motif in terms of fixed positions.
FullLength	Maximum length of the motif, including wildcard positions.
Expect	The expected number of times the motif will occur in the search database given (`searchdb=FILE`).
IC	Information Content of motif (if `motific=T`).

2.3.7: Log Files

The presto log file records information that may help subsequent interpretation of results or identify problems. Probably it's most useful content is any error messages generated. By default the log file is presto.log but this can be changed with the `log=FILE` option. Logs will be appended unless the `newlog` option is used. (See **4.3: Appendix III: Log Files** for details.)

2.4: Information Content

Information content is calculated for each motif based on a uniform distribution of amino acids and re-scaled to give a value of 1.0 per fixed position and 0.0 for a wildcard. Ambiguous positions are given a value between 0.0 and 1.0:

$IC_i = [\log2(0.05) – \log2(1/f_a)] / \log2(0.05)$,

where IC_i is the information content for position i and f_a is the number of possible amino acids at position i. The information content for the motif is simply this score summed over all positions.

3: Conservation Statistics

3.1: Conservation Output

An important function of PRESTO that sets it apart from other such tools is the ability to calculate conservation statistics for each match, provided alignment files are provided. (see **2.2.5: Optional Input 1: Multiple Sequence Alignments**). If alignments do not exist, **GOPHER** (Edwards 2006) can be used to generate them (see **3.4: Generating Alignments with GOPHER**). If the identified file is not actually aligned, then RJE_SEQ will try to align the proteins using MUSCLE or ClustalW.

For each sequence, these alignments are used to generate the global percentage identity statistic:

- GLOB_ID = Mean global percentage identity between query protein and homologues. This is calculated direct from the alignments, excluding matches of Xs, and is the percentage of query residues that match the aligned residue in the homologue. (Note that this is an asymmetrical measurement and the percentage of the homologue that aligns with the query may be very different if the sequences are of different lengths.)

Other conservation statistics are calculated individually for each occurrence of the motif. These are based on the homologous protein sequences available *at that site*. Any homologues with masked (X) residues that coincide to non-wildcard positions of the motif occurrence will be ignored from conservation calculations. Gaps, however, shall be treated as divergence unless the `alngap=F` option is used, in which case 100% gapped regions of homologues are also ignored (see **3.3.4: Gap Treatment**). These additional statistics are:

- MOTIF_CONS = This is the conservation score across available homologues for that occurrence
- HOM_NUM = Number of available homologues for that occurrence
- LOC_ID = Mean local percentage identity between query protein and available homologues across region of match

3.2: Conservation Scores

Currently, there are three main Conservation scores implemented in PRESTO, which can be selected with the `conscore=X` option:

3.2.1: Absolute Conservation [`conscore=abs`]

For absolute conservation, PRESTO first identifies the regions of the alignment that correspond to matches in the Query protein. Each aligned sequence is then taken in turn and the relevant region extracted, de-gapped, and compared to the original regular expression, *i.e.* the *degenerate* motif. The conservation score is then the proportion of these homologues in which the degenerate motif is conserved (**Figure 1**). (To calculate conservation of the specific occurrence of the motif, use the `consamb=F` option.)

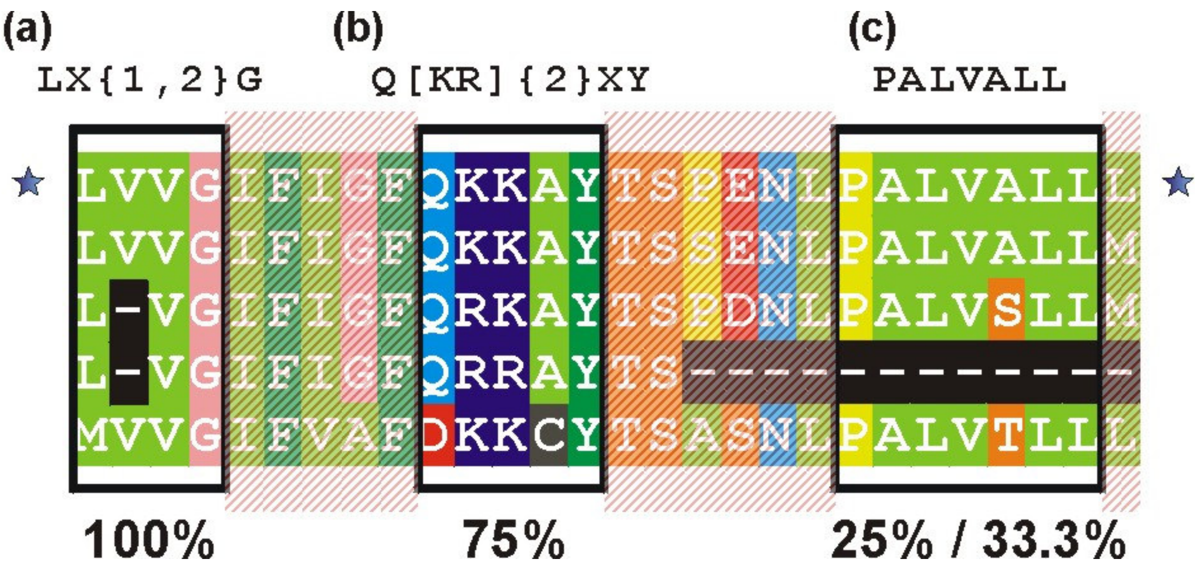

Figure 1. PRESTO Absolute Conservation Example. Three motifs are found in the query protein (marked with blue stars). This protein is ignored for conservation statistics. The black boxes represent the region of the alignment considered for each match. These matches in the homologues are then compared to the original regular expression. **(a)** Motif LX{1,2}G is 100% conserved because, once gaps are removed, all four homologous sequences match the degenerate motif. **(b)** For motif Q[KR]{2}XY, one sequence does not match the degenerate motif and the query is excluded from the calculation, giving a conservation score of ¾ = 75%. **(c)** Only one of the homologues matches the motif. By default, all four sequences are considered, giving a conservation score of ¼ = 25%. If the **alngap=F** option is used, the 100% gapped sequence is ignored and the conservation is therefore 1/3 = 33.3%

3.2.2: Positional Scoring [conscore=pos]

Positional scoring uses a graded scoring system, where each sequence gets a score between 0 (no positions conserved) and 1 (all positions conserved). By default, each matching amino acid contributes a score of 1.0 and the sum over all positions is divided by the number of positions. For a degenerate site (when the default **consamb=T** option is used), the sequence must match *any* possible amino acid at that site.

The scoring matrix used for this scoring can be altered using the **posmatrix=FILE** command, where FILE contains either lists of equivalent amino acids on each line (e.g. FYW would mean that any of F, Y or W would score 1.0 vs. any other of F, Y or W), or an all-by-all matrix of amino acids and their conservation scores, e.g. this might give F-F a score of 1.0 and F-Y a score of 0.5. This allows the method to be customised according to user-determined rules. In this case, the best score between the sequence and any variant of a degenerate position is used (unless **consamb=F**).

3.2.3: AA Property Scoring [conscore=prop]

This is really just a specific example of the **posmatrix=FILE** command, where an amino acid property matrix (**aaprop=FILE**) is converted into a similarity matrix ranging from 0.0 to 1.0. By default, the property matrix of Livingstone and Barton is used (**aaprop.txt**) (Livingstone and Barton 1993). See the **PEAT Appendices** for more information on this matrix.

3.2.4: Combined Scoring [conscore=all]

Under this scoring, the MOTIF_CONS output is the **mean** of all three methods. In addition, statistics are generated for each of the individual scores:

- CONS_POS = Positional conservation score across homologues.
- CONS_ABS = Absolute conservation score across homologues.
- CONS_PROP = Property-based conservation score across homologues.

The same additional options are applied to all three methods, with the exception that Positional Weighting by Information Content has no effect on the absolute conservation method.

3.2.5: Additional output: conservation score used

Because there are so many options, it is sometimes desirable to have a record of the conservation score used to generate a set of results. The **consout=T** option outputs an additional CONS_METHOD result field containing information on the conservation score used in the form **method**_w**X**_a**Y**_i**Z**, where **method** is the method name (**conscore=method**) **X** is the weighting used (**consweight=X**), **Y** is whether ambiguity is used (**consamb=T/F**) and **Z** is whether positional weighting by information content is used (**consinfo=T/F**). These options are described below.

3.3: Additional Options

3.3.1: Motif ambiguity

By default, PRESTO will calculate conservation using the full degeneracy of the input motif. If **consamb=F** is used, the particular matching variant will be used instead. *E.g.* in **Figure 1(b)**, the conservation of QKXXY would be calculated, rather than Q[KR]XXY. When the **ambcut=X** option is used, degenerate sites with more ambiguity than ambcut will be treated as wildcards the conservation of a particular variant (*i.e.* not count towards the conservation score).

3.3.2: Positional Weighting by Information Content

The **consinfo=T** option (the default) weights the contribution of different positions of the motif proportionally to their information content (IC). The IC of a position ranges from 0 for a wildcard position to 1 for a fixed position (see **2.4: Information Content**). For a fully fixed motif, all positions will have equal weighting. Otherwise, ambiguous positions make a smaller contribution to the score, which is normalised such that a sequence that is conserved at every position of the motif gets a score of 1.0. If **consinfo=F**, all positions contribute equally. When the **ambcut=X** option is used, degenerate sites with more ambiguity than ambcut will be treated as wildcards for IC weighting (*i.e.* not count towards the conservation score). If the "Absolute" motif conservation score is used, this weighting has no affect.

3.3.3: Homology Weighting

The **consweight=X** option controls how the conservation scores are weighted according the similarity of the homologues to the query. For each sequence *s*, the weighting W_s is calculated using the global percentage identity of the query versus that sequence, I_s, raised to the power of the **consweight=X** option, ω:

$W_s = I_s^{\omega} / \Sigma W_s$

When ω=0 (the default), W_s = 1 and all sequences are treated equally.

For ω=1, W_s = I_s, which up-weights the contribution of sequences closely related to the query. This means that the comparison of conservation scores will tend to penalise

divergence in closely related sequences and will not be so heavily influenced by incorrect orthology assignment of distantly-related sequences. This weighting can be increased further with $\omega > 1$.

For ω=-1, $W_s = 1/I_s$, which up-weights the contribution of sequences distantly related to the query. This means that the comparison of conservation scores will tend to promote conservation in distantly related sequences but may be influenced by incorrect orthology assignment, which tends to be more of a problem for distantly-related sequences. This weighting can be increased further with $\omega < -1$.

3.3.4: Gap Treatment

By default, motifs that match to 100% gaps in a homologue will be treated as divergence away from a motif and reduce the score. If, however, there are a lot of missing or truncated sequences (in a draft genome, for example,) the **alngap=F** option can be used to ignore these sequences for the relevant calculations (see **Figure 1(c)**). Note that the calculation used pretends that these homologues are not present at all, and does not count them as conserved.

3.4: Generating Alignments with GOPHER

The program **GOPHER** (Edwards 2006) is provided in the download and can be called as part of the **PRESTO** search using the **gopher=T** option. Details of how **GOPHER** works can be found in the GOPHER documentation. GOPHER will generate alignments in the directory specified by the **alndir=PATH** option. If this does not point to a subdirectory named ALN, one will be created and GOPHER alignments will be placed in here (The **alndir=PATH** parameter will be updated accordingly). Additional GOPHER output and directories will be created in the parent directory. If not already set as such, the **alnext=X** option will be set to **orthaln.fas**.

4: Appendices

4.1: Appendix I: Command-line Options

General details on command-line options can now be found in the **PEAT Appendices** document distributed with this program. A full list of options can be found in the distributed **readme.txt** and **readme.html** files.

4.1.1: Forking Options

PRESTO can run multiple forks at a time, processing a single sequence with each fork. This will speed up execution on a multiple-processor machine. Forking is controlled by three main parameters:

- **forks=X** sets the number of processes to fork at any given time.
- **killforks=X** sets the number of seconds without any activity from any forks before the program will commit suicide.
- **noforks=T/F** sets whether forks are used at all.

IMPORTANT: Forking has been implemented for UNIX only. (Python does not support forking in Windows.) If running in windows, use the **win32=T**, **forks=0** or **noforks=T** options.

4.2: Appendix II: Distributed Python Modules

Details can be found in the distributed **readme.txt** and **readme.html** files as well as the accompanying **PEAT Appendices** document.

4.3: Appendix III: Log Files

See the accompanying **PEAT Appendices** document for general information on log files.

4.4: Appendix IV: Troubleshooting

There are currently no specific Troubleshooting issues arising with PRESTO. Please see general items in the **PEAT Appendices** document and contact me if you experience any problems not covered.

4.5: Appendix V: References

Edwards, R.J. (2006) GOPHER: Generation of Orthologous Proteins using High-throughput Evolutionary Relationships. *http://bioinformatics.ucd.ie/shields/software/gopher/*

Livingstone, C.D. et al. (1993) Protein sequence alignments: a strategy for the hierarchical analysis of residue conservation, *Comput Appl Biosci*, **9**, 745-756.
