# Supplementary material for: Estimation and efficient computation of the true probability of recurrence of short linear protein sequence motifs in unrelated proteins
Source: BMC Bioinformatics. 2010 Jan 7;11:14. doi: 10.1186/1471-2105-11-14 (PMC2819990; doi:10.1186/1471-2105-11-14)
Supplement: Additional file 1 — Slimsuite software package. This ZIP file contains the slimsuite software package used with this submission. [file 1471-2105-11-14-S1.ZIP › slimsuite/readme.html]

SLIMSUITE Release ReadMe


## ReadMe documentation for release of SLIMSUITE software

**Distribution compiled:** Thu Sep 10 11:53:46 2009

**Questions/Comments?:** please contact
software@cabbagesofdoom.co.uk

---

## Installation Instructions

1. Place the slimsuite.zip file in chosen directory (e.g. c:\bioware\) and unzip.- A subdirectory slimsuite will be created containing all the necessary files to run

The software should run on any system that has Python installed.
Additional software may be necessary for full functionality. Further details can be found in the manuals supplied.

---

## GNU License

Copyright (C) 2009 Richard J. Edwards <software@cabbagesofdoom.co.uk>

This program is free software; you can redistribute it and/or modify it under the terms of the GNU General Public License as published by the Free Software Foundation; either version 2 of the License, or (at your option) any later version.

This program is distributed in the hope that it will be useful, but WITHOUT ANY WARRANTY; without even the implied warranty of MERCHANTABILITY or FITNESS FOR A PARTICULAR PURPOSE. See the GNU General Public License for more details.

You should have received a copy of the GNU General Public License along with this program; if not, write to the Free Software Foundation, Inc., 51 Franklin St, Fifth Floor, Boston, MA 02110-1301 USA

Author contact: <software@cabbagesofdoom.co.uk> / School of Biological Sciences, University of Southampton, UK.

To incorporate this module into your own programs, please see GNU Lesser General Public License disclaimer in rje.py

---

## Distributed Files

### Python Modules

- **slimfinder [version 4.0]** (Short Linear Motif Finder)- **slimsearch [version 1.3]** (Short Linear Motif Search tool)- **qslimfinder [version 1.0]** (Query Short Linear Motif Finder)- **comparimotif\_V3 [version 3.5]** (Motif vs Motif Comparison Software)- **gopher\_V2 [version 2.5]** (Generation of Orthologous Proteins from High-Throughput Estimation of Relationships)- **ned\_eigenvalues [version 1.0]** (Modified N. Davey Relative Local Conservation module)- **ned\_rankbydistribution [version 1.0]** (Modified SLiMFinder stats module)- **rje [version 3.9]** (Contains General Objects for all my (Rich's) scripts)- **rje\_aaprop [version 0.1]** (AA Property Matrix Module)- **rje\_ancseq [version 1.2]** (Ancestral Sequence Prediction Module)- **rje\_blast [version 1.10]** (BLAST Control Module)- **rje\_dismatrix\_V2 [version 2.3]** (Distance Matrix Module )- **rje\_disorder [version 0.5]** (Disorder Prediction Module)- **rje\_hmm [version 1.3]** (HMMer Control Module)- **rje\_menu [version 0.2]** (Generic Menu Methods Module)- **rje\_motif\_V3 [version 3.0]** (Motif Class and Methods Module)- **rje\_motif\_stats [version 1.0]** (Motif Statistics Methods Module)- **rje\_motiflist [version 1.0]** (RJE Motif List Module)- **rje\_motifocc [version 0.0]** (Motif Occurrence Module)- **rje\_pam [version 1.2]** (Contains Objects for PAM matrices)- **rje\_scoring [version 0.0]** (Scoring and Ranking Methods for RJE Python Modules)- **rje\_seq [version 3.6]** (DNA/Protein sequence module)- **rje\_sequence [version 1.12]** (DNA/Protein sequence object)- **rje\_slim [version 1.1]** (Short Linear Motif class module)- **rje\_slimcalc [version 0.3]** (SLiM Attribute Calculation Module)- **rje\_slimcore [version 1.4]** (Core module/object for SLiMFinder and SLiMSearch)- **rje\_slimlist [version 0.3]** (SLiM dataset manager)- **rje\_tm [version 1.2]** (Tranmembrane and Signal Peptide Prediction Module)- **rje\_tree [version 2.5]** (Phylogenetic Tree Module)- **rje\_tree\_group [version 1.2]** (Contains all the Grouping Methods for rje\_tree.py)- **rje\_uniprot [version 3.5]** (RJE Module to Handle Uniprot Files)- **rje\_xgmml [version 0.0]** (RJE XGMLL Module )- **rje\_zen [version 1.0]** (Random Zen Wisdom Generator)- **unifake [version 1.2]** (Fake UniProt DAT File Generator)

### Other Files

- SLiMFinder Manual.pdf- PEAT Appendices.pdf- PRESTO Manual.pdf- GOPHER Manual.pdf- RJE\_SEQ Manual.pdf- gnu\_general\_public\_license.txt- gnu\_lesser\_general\_public\_license.txt- aaprop.txt- jones.pam- blosum62.bla

---

## \*\*\* Module slimfinder \*\*\* [Top]

**Module:** slimfinder  
**Description:** Short Linear Motif Finder  
**Version:** 4.0  
**Last Edit:** 02/09/09  
**Copyright (C) 2007 Richard J. Edwards - See source code for GNU License Notice**  
  
**Function:**  
Short linear motifs (SLiMs) in proteins are functional microdomains of fundamental importance in many biological  
systems. SLiMs typically consist of a 3 to 10 amino acid stretch of the primary protein sequence, of which as few  
as two sites may be important for activity, making identification of novel SLiMs extremely difficult. In particular,  
it can be very difficult to distinguish a randomly recurring "motif" from a truly over-represented one. Incorporating  
ambiguous amino acid positions and/or variable-length wildcard spacers between defined residues further complicates  
the matter.  
  
SLiMFinder is an integrated SLiM discovery program building on the principles of the SLiMDisc software for accounting  
for evolutionary relationships [Davey NE, Shields DC & Edwards RJ (2006): Nucleic Acids Res. 34(12):3546-54].  
SLiMFinder is comprised of two algorithms:  
  
SLiMBuild identifies convergently evolved, short motifs in a dataset. Motifs with fixed amino acid positions are  
identified and then combined to incorporate amino acid ambiguity and variable-length wildcard spacers. Unlike  
programs such as TEIRESIAS, which return all shared patterns, SLiMBuild accelerates the process and reduces returned  
motifs by explicitly screening out motifs that do not occur in enough unrelated proteins. For this, SLiMBuild uses  
the "Unrelated Proteins" (UP) algorithm of SLiMDisc in which BLAST is used to identify pairwise relationships.  
Proteins are then clustered according to these relationships into "Unrelated Protein Clusters" (UPCs), which are  
defined such that no protein in a UPC has a BLAST-detectable relationship with a protein in another UPC. If desired,  
SLiMBuild can be used as a replacement for TEIRESIAS in other software (teiresias=T slimchance=F).  
  
SLiMChance estimates the probability of these motifs arising by chance, correcting for the size and composition of  
the dataset, and assigns a significance value to each motif. Motif occurrence probabilites are calculated  
independently for each UPC, adjusted the size of a UPC using the Minimum Spanning Tree algorithm from SLiMDisc. These  
individual occurrence probabilities are then converted into the total probability of the seeing the observed motifs  
the observed number of (unrelated) times. These probabilities assume that the motif is known before the search. In  
reality, only over-represented motifs from the dataset are looked at, so these probabilities are adjusted for the  
size of motif-space searched to give a significance value. This is an estimate of the probability of seeing that  
motif, or another one like it. These values are calculated separately for each length of motif. Where pre-known  
motifs are also of interest, these can be given with the slimcheck=MOTIFS option and will be added to the output.  
SLiMFinder version 4.0 introduced a more precise (but more computationally intensive) statistical model, which can  
be switched on using sigprime=T. Likewise, the more precise (but more computationally intensive) correction to the  
mean UPC probability heuristic can be switched on using sigv=T. (Note that the other SLiMChance options may not  
work with either of these options.) The allsig=T option will output all four scores. In this case, SigPrimeV will be  
used for ranking etc. unless probscore=X is used.  
  
Where significant motifs are returned, SLiMFinder will group them into Motif "Clouds", which consist of physically  
overlapping motifs (2+ non-wildcard positions are the same in the same sequence). This provides an easy indication  
of which motifs may actually be variants of a larger SLiM and should therefore be considered together.  
  
Additional Motif Occurrence Statistics, such as motif conservation, are handled by the rje\_slimlist module. Please  
see the documentation for this module for a full list of commandline options. These options are currently under  
development for SLiMFinder and are not fully supported. See the SLiMFinder Manual for further details. Note that the  
OccFilter \*does\* affect the motifs returned by SLiMBuild and thus the TEIRESIAS output (as does min. IC and min.  
Support) but the overall Motif StatFilter \*only\* affects SLiMFinder output following SLiMChance calculations.  
  
**Secondary Functions:**  
The "MotifSeq" option will output fasta files for a list of X:Y, where X is a motif pattern and Y is the output file.  
  
The "Randomise" function will take a set of input datasets (as in Batch Mode) and regenerate a set of new datasets  
by shuffling the UPC among datasets. Note that, at this stage, this is quite crude and may result in the final  
datasets having fewer UPC due to common sequences and/or relationships between UPC clusters in different datasets.  
  
**Commandline:** ~~~~~~~~~~~~~~~~~~~~~~~~~~~~~~~~~~~~~~~~~~~~~~~~~~~~~~~~~~~~~~~~~~~~~~~~~~~~~~~~~~~~~~~~~~~~~~~~~~~~~~~~~~~#  
### Basic Input/Output Options ###   
- **seqin=FILE :** Sequence file to search [None]  
  - **batch=LIST :** List of files to search, wildcards allowed. (Over-ruled by seqin=FILE.) [\*.dat,\*.fas]  
    - **maxseq=X :** Maximum number of sequences to process [500]  
      - **maxupc=X :** Maximum UPC size of dataset to process [0]  
        - **walltime=X :** Time in hours before program will abort search and exit [1.0]  
          - **resfile=FILE :** Main SLiMFinder results table [slimfinder.csv]  
            - **resdir=PATH :** Redirect individual output files to specified directory (and look for intermediates) [SLiMFinder/]  
              - **buildpath=PATH :** Alternative path to look for existing intermediate files [SLiMFinder/]  
                - **force=T/F :** Force re-running of BLAST, UPC generation and SLiMBuild [False]  
                  - **pickup=T/F :** Pick-up from aborted batch run by identifying last dataset output in resfile [False]  
                    - **dna=T/F :** Whether the sequences files are DNA rather than protein [False]  
                      #~~~~~~~~~~~~~~~~~~~~~~~~~~~~~~~~~~~~~~~~~~~~~~~~~~~~~~~~~~~~~~~~~~~~~~~~~~~~~~~~~~~~~~~~~~~~~~~~~~~~~~~~~~~~~~~~~~~#  
                      ### SLiMBuild Options I: Evolutionary Filtering ###  
                      - **efilter=T/F :** Whether to use evolutionary filter [True]  
                        - **blastf=T/F :** Use BLAST Complexity filter when determining relationships [True]  
                          - **blaste=X :** BLAST e-value threshold for determining relationships [1e=4]  
                            - **altdis=FILE :** Alternative all by all distance matrix for relationships [None]  
                              - **gablamdis=FILE :** Alternative GABLAM results file [None] (!!!Experimental feature!!!)  
                                - **homcut=X :** Max number of homologues to allow (to reduce large multi-domain families) [0]  
                                    
                                  ### SLiMBuild Options II: Input Masking ###  
                                  - **masking=T/F :** Master control switch to turn off all masking if False [True]  
                                    - **dismask=T/F :** Whether to mask ordered regions (see rje\_disorder for options) [False]  
                                      - **consmask=T/F :** Whether to use relative conservation masking [False]  
                                        - **ftmask=LIST :** UniProt features to mask out [EM]  
                                          - **imask=LIST :** UniProt features to inversely ("inclusively") mask. (Seqs MUST have 1+ features) []  
                                            - **compmask=X,Y :** Mask low complexity regions (same AA in X+ of Y consecutive aas) [5,8]  
                                              - **casemask=X :** Mask Upper or Lower case [None]  
                                                - **motifmask=X :** List (or file) of motifs to mask from input sequences []  
                                                  - **metmask=T/F :** Masks the N-terminal M (can be useful if termini=T) [True]  
                                                    - **posmask=LIST :** Masks list of position-specific aas, where list = pos1:aas,pos2:aas [2:A]  
                                                      - **aamask=LIST :** Masks list of AAs from all sequences (reduces alphabet) []  
                                                          
                                                        ### SLiMBuild Options III: Basic Motif Construction ###  
                                                        - **termini=T/F :** Whether to add termini characters (^ & $) to search sequences [True]  
                                                          - **minwild=X :** Minimum number of consecutive wildcard positions to allow [0]  
                                                            - **maxwild=X :** Maximum number of consecutive wildcard positions to allow [2]  
                                                              - **slimlen=X :** Maximum length of SLiMs to return (no. non-wildcard positions) [5]  
                                                                - **minocc=X :** Minimum number of unrelated occurrences for returned SLiMs. (Proportion of UP if < 1) [0.05]  
                                                                  - **absmin=X :** Used if minocc<1 to define absolute min. UP occ [3]  
                                                                    - **alphahelix=T/F :** Special i, i+3/4, i+7 motif discovery [False]  
                                                                        
                                                                      ### SLiMBuild Options IV: Ambiguity ###  
                                                                      - **preamb=T/F :** Whether to search for ambiguous motifs during motif discovery [True]  
                                                                        - **ambocc=X :** Min. UP occurrence for subvariants of ambiguous motifs (minocc if 0 or > minocc) [0.05]  
                                                                          - **absminamb=X :** Used if ambocc<1 to define absolute min. UP occ [2]  
                                                                            - **equiv=LIST :** List (or file) of TEIRESIAS-style ambiguities to use [AGS,ILMVF,FYW,FYH,KRH,DE,ST]  
                                                                              - **wildvar=T/F :** Whether to allow variable length wildcards [True]  
                                                                                - **combamb=T/F :** Whether to search for combined amino acid degeneracy and variable wildcards [False]  
                                                                                    
                                                                                  ### SLiMBuild Options V: Advanced Motif Filtering ###  
                                                                                  - **musthave=LIST :** Returned motifs must contain one or more of the AAs in LIST (reduces search space) []  
                                                                                    - **query=LIST :** Return only SLiMs that occur in 1+ Query sequences (Name/AccNum) []  
                                                                                      - **focus=FILE :** FILE containing focal groups for SLiM return (see Manual for details) [None]  
                                                                                        - **focusocc=X :** Motif must appear in X+ focus groups (0 = all) [0]  
                                                                                          \* See also rje\_slimcalc options for occurrence-based calculations and filtering \*  
                                                                                            
                                                                                          #~~~~~~~~~~~~~~~~~~~~~~~~~~~~~~~~~~~~~~~~~~~~~~~~~~~~~~~~~~~~~~~~~~~~~~~~~~~~~~~~~~~~~~~~~~~~~~~~~~~~~~~~~~~~~~~~~~~#  
                                                                                          ### SLiMChance Options ###  
                                                                                          - **slimchance=T/F :** Execute main SLiMFinder probability method and outputs [True]  
                                                                                            - **sigprime=T/F :** Calculate more precise (but more computationally intensive) statistical model [False]  
                                                                                              - **sigv=T/F :** Use the more precise (but more computationally intensive) fix to mean UPC probability [False]  
                                                                                                - **dimfreq=T/F :** Whether to use dimer masking pattern to adjust number of possible sites for motif [True]  
                                                                                                  - **probcut=X :** Probability cut-off for returned motifs [0.1]  
                                                                                                    - **maskfreq=T/F :** Whether to use masked AA Frequencies (True), or (False) mask after frequency calculations [True]  
                                                                                                      - **aafreq=FILE :** Use FILE to replace individual sequence AAFreqs (FILE can be sequences or aafreq) [None]  
                                                                                                        - **aadimerfreq=FILE:** Use empirical dimer frequencies from FILE (fasta or \*.aadimer.tdt) (!!!Experimental!!!) [None]  
                                                                                                          - **negatives=FILE :** Multiply raw probabilities by under-representation in FILE (!!!Experimental!!!) [None]  
                                                                                                            - **smearfreq=T/F :** Whether to "smear" AA frequencies across UPC rather than keep separate AAFreqs [False]  
                                                                                                              - **seqocc=T/F :** Whether to upweight for multiple occurrences in same sequence (heuristic) [False]  
                                                                                                                - **probscore=X :** Score to be used for probability cut-off and ranking (Prob/Sig/S/R) [Sig]  
                                                                                                                  #~~~~~~~~~~~~~~~~~~~~~~~~~~~~~~~~~~~~~~~~~~~~~~~~~~~~~~~~~~~~~~~~~~~~~~~~~~~~~~~~~~~~~~~~~~~~~~~~~~~~~~~~~~~~~~~~~~~#  
                                                                                                                  ### Advanced Output Options I: Output data ###  
                                                                                                                  - **clouds=X :** Identifies motif "clouds" which overlap at 2+ positions in X+ sequences (0=minocc / -1=off) [2]  
                                                                                                                    - **runid=X :** Run ID for resfile (allows multiple runs on same data) [DATE:TIME]  
                                                                                                                      - **logmask=T/F :** Whether to log the masking of individual sequences [True]  
                                                                                                                        - **slimcheck=FILE :** Motif file/list to add to resfile output []   
                                                                                                                            
                                                                                                                          ### Advanced Output Options II: Output formats ###  
                                                                                                                          - **teiresias=T/F :** Replace TEIRESIAS, making \*.out and \*.mask.fasta files [False]  
                                                                                                                            - **slimdisc=T/F :** Emulate SLiMDisc output format (\*.rank & \*.dat.rank + TEIRESIAS \*.out & \*.fasta) [False]  
                                                                                                                              - **extras=X :** Whether to generate additional output files (alignments etc.) [1]  
                                                                                                                                - 0 = No output beyond main results file  
                                                                                                                                - 1 = Generate occurrence file, alignments and cloud file  
                                                                                                                                - 2 = Generate all additional SLiMFinder outputs  
                                                                                                                                - 3 = Generate SLiMDisc emulation too (equiv extras=2 slimdisc=T)  
                                                                                                                                - **targz=T/F :** Whether to tar and zip dataset result files (UNIX only) [False]  
                                                                                                                                  - **savespace=0 :** Delete "unneccessary" files following run (best used with targz): [0]  
                                                                                                                                    - 0 = Delete no files  
                                                                                                                                    - 1 = Delete all bar \*.upc and \*.pickle and \*.occ.csv files  
                                                                                                                                    - 2 = Delete all dataset-specific files including \*.upc and \*.pickle (not \*.tar.gz)  
                                                                                                                                      
                                                                                                                                    ### Advanced Output Options III: Additional Motif Filtering ###   
                                                                                                                                    - **topranks=X :** Will only output top X motifs meeting probcut [1000]  
                                                                                                                                      - **oldscores=T/F :** Whether to also output old SLiMDisc score (S) and SLiMPickings score (R) [False]  
                                                                                                                                        - **allsig=T/F :** Whether to also output all SLiMChance combinations (Sig/SigV/SigPrime/SigPrimeV) [False]  
                                                                                                                                          - **minic=X :** Minimum information content for returned motifs [2.1]  
                                                                                                                                            \* See also rje\_slimcalc options for occurrence-based calculations and filtering \*  
                                                                                                                                              
                                                                                                                                            #~~~~~~~~~~~~~~~~~~~~~~~~~~~~~~~~~~~~~~~~~~~~~~~~~~~~~~~~~~~~~~~~~~~~~~~~~~~~~~~~~~~~~~~~~~~~~~~~~~~~~~~~~~~~~~~~~~~#  
                                                                                                                                            ### Additional Functions I: MotifSeq ###   
                                                                                                                                            - **motifseq=LIST :** Outputs fasta files for a list of X:Y, where X is the pattern and Y is the output file []  
                                                                                                                                              - **slimbuild=T/F :** Whether to build motifs with SLiMBuild. (For combination with motifseq only.) [True]  
                                                                                                                                                  
                                                                                                                                                ### Additional Functions II: Randomised datasets ###  
                                                                                                                                                - **randomise=T/F :** Randomise UPC within batch files and output new datasets [False]  
                                                                                                                                                  - **randir=PATH :** Output path for creation of randomised datasets [Random/]  
                                                                                                                                                    - **randbase=X :** Base for random dataset name [rand]  
                                                                                                                                                      #~~~~~~~~~~~~~~~~~~~~~~~~~~~~~~~~~~~~~~~~~~~~~~~~~~~~~~~~~~~~~~~~~~~~~~~~~~~~~~~~~~~~~~~~~~~~~~~~~~~~~~~~~~~~~~~~~~~#  
                                                                                                                                                        
                                                                                                                                                      **Uses general modules:** copy, glob, math, os, string, sys, time  
                                                                                                                                                      **Uses RJE modules:** rje, rje\_blast, rje\_slim, rje\_slimlist, rje\_slimcalc, rje\_slimcore, rje\_dismatrix\_V2, rje\_seq, rje\_scoring  
                                                                                                                                                      **Other modules needed:** None

---


## \*\*\* Module slimsearch \*\*\* [Top]

**Module:** SLiMSearch  
**Description:** Short Linear Motif Search tool  
**Version:** 1.3  
**Last Edit:** 29/05/09  
**Copyright (C) 2007 Richard J. Edwards - See source code for GNU License Notice**  
  
**Function:**  
SLiMSearch is a tool for finding pre-defined SLiMs (Short Linear Motifs) in a protein sequence database. SLiMSearch  
can make use of corrections for evolutionary relationships and a variation of the SLiMChance alogrithm from  
SLiMFinder to assess motifs for statistical over- and under-representation. SLiMSearch is a replacement for PRESTO  
and uses many of the same underlying modules.  
  
Benefits of SLiMSearch that make it more useful than a lot of existing tools include:  
\* searching with mismatches rather than restricting hits to perfect matches.  
\* optional equivalency files for searching with specific allowed mismatched (e.g. charge conservation)  
\* generation or reading of alignment files from which to calculate conservation statistics for motif occurrences.  
\* additional statistics, inlcuding protein disorder, surface accessibility and hydrophobicity predictions  
\* recognition of "n of m" motif elements in the form , where X is one or more amino acids that must occur n+  
times across which m positions. E.g.  must have 3+ Is and/or Ls in a 5aa stretch.  
  
Main output for SLiMSearch is a delimited file of motif/peptide occurrences but the motifaln=T and proteinaln=T also  
allow output of alignments of motifs and their occurrences.  
  
**Commandline:** ~~~~~~~~~~~~~~~~~~~~~~~~~~~~~~~~~~~~~~~~~~~~~~~~~~~~~~~~~~~~~~~~~~~~~~~~~~~~~~~~~~~~~~~~~~~~~~~~~~~~~~~~~~~#  
### Basic Input/Output Options ###  
- **motifs=FILE :** File of input motifs/peptides [None]  
  Single line per motif format = 'Name Sequence #Comments' (Comments are optional and ignored)  
  Alternative formats include fasta, SLiMDisc output and raw motif lists.  
  - **seqin=FILE :** Sequence file to search [None]  
    - **batch=LIST :** List of sequence files for batch input (wildcard \* permitted) []  
      - **maxseq=X :** Maximum number of sequences to process [0]  
        - **maxsize=X :** Maximum dataset size to process in AA (or NT) [100,000]  
          - **walltime=X :** Time in hours before program will abort search and exit [1.0]  
            - **resfile=FILE :** Main SLiMSearch results table [slimsearch.csv]  
              - **resdir=PATH :** Redirect individual output files to specified directory (and look for intermediates) [SLiMSearch/]  
                - **buildpath=PATH :** Alternative path to look for existing intermediate files [SLiMSearch/]  
                  - **force=T/F :** Force re-running of BLAST, UPC generation and SLiMBuild [False]  
                    #~~~~~~~~~~~~~~~~~~~~~~~~~~~~~~~~~~~~~~~~~~~~~~~~~~~~~~~~~~~~~~~~~~~~~~~~~~~~~~~~~~~~~~~~~~~~~~~~~~~~~~~~~~~~~~~~~~~#  
                    ### SearchDB Options I: Input Protein Sequence Masking ###  
                    - **masking=T/F :** Master control switch to turn off all masking if False [False]  
                      - **dismask=T/F :** Whether to mask ordered regions (see rje\_disorder for options) [False]  
                        - **consmask=T/F :** Whether to use relative conservation masking [False]  
                          - **ftmask=LIST :** UniProt features to mask out [EM,DOMAIN,TRANSMEM]  
                            - **imask=LIST :** UniProt features to inversely ("inclusively") mask. (Seqs MUST have 1+ features) []  
                              - **compmask=X,Y :** Mask low complexity regions (same AA in X+ of Y consecutive aas) [5,8]  
                                - **casemask=X :** Mask Upper or Lower case [None]  
                                  - **motifmask=X :** List (or file) of motifs to mask from input sequences []  
                                    - **metmask=T/F :** Masks the N-terminal M [False]  
                                      - **posmask=LIST :** Masks list of position-specific aas, where list = pos1:aas,pos2:aas [2:A]  
                                        - **aamask=LIST :** Masks list of AAs from all sequences (reduces alphabet) []  
                                            
                                          ### SearchDB Options II: Evolutionary Filtering ###  
                                          - **efilter=T/F :** Whether to use evolutionary filter [False]  
                                            - **blastf=T/F :** Use BLAST Complexity filter when determining relationships [True]  
                                              - **blaste=X :** BLAST e-value threshold for determining relationships [1e=4]  
                                                - **altdis=FILE :** Alternative all by all distance matrix for relationships [None]  
                                                  - **gablamdis=FILE :** Alternative GABLAM results file [None] (!!!Experimental feature!!!)  
                                                    #~~~~~~~~~~~~~~~~~~~~~~~~~~~~~~~~~~~~~~~~~~~~~~~~~~~~~~~~~~~~~~~~~~~~~~~~~~~~~~~~~~~~~~~~~~~~~~~~~~~~~~~~~~~~~~~~~~~#  
                                                    ### SLiMChance Options ###  
                                                    - **maskfreq=T/F :** Whether to use masked AA Frequencies (True), or (False) mask after frequency calculations [True]  
                                                      - **aafreq=FILE :** Use FILE to replace individual sequence AAFreqs (FILE can be sequences or aafreq) [None]  
                                                        - **aadimerfreq=FILE:** Use empirical dimer frequencies from FILE (fasta or \*.aadimer.tdt) [None]  
                                                          - **negatives=FILE :** Multiply raw probabilities by under-representation in FILE [None]  
                                                            - **background=FILE :** Use observed support in background file for over-representation calculations [None]  
                                                              - **smearfreq=T/F :** Whether to "smear" AA frequencies across UPC rather than keep separate AAFreqs [False]  
                                                                - **seqocc=X :** Restrict to sequences with X+ occurrences (adjust for high frequency SLiMs) [1]  
                                                                  #~~~~~~~~~~~~~~~~~~~~~~~~~~~~~~~~~~~~~~~~~~~~~~~~~~~~~~~~~~~~~~~~~~~~~~~~~~~~~~~~~~~~~~~~~~~~~~~~~~~~~~~~~~~~~~~~~~~#  
                                                                  ### Advanced Output Options II: Output formats ###  
                                                                  - **teiresias=T/F :** Replace TEIRESIAS, making \*.out and \*.mask.fasta files [False]  
                                                                    - **extras=T/F :** Whether to generate additional output files (alignments etc.) [True]  
                                                                      - **pickle=T/F :** Whether to save/use pickles [True]  
                                                                        - **targz=T/F :** Whether to tar and zip dataset result files (UNIX only) [False]  
                                                                          - **savespace=0 :** Delete "unneccessary" files following run (best used with targz): [0]  
                                                                            - 0 = Delete no files  
                                                                            - 1 = Delete all bar \*.upc and \*.pickle files  
                                                                            - 2 = Delete all dataset-specific files including \*.upc and \*.pickle (not \*.tar.gz)  
                                                                            \* See also rje\_slimcalc options for occurrence-based calculations and filtering \*  
                                                                            #~~~~~~~~~~~~~~~~~~~~~~~~~~~~~~~~~~~~~~~~~~~~~~~~~~~~~~~~~~~~~~~~~~~~~~~~~~~~~~~~~~~~~~~~~~~~~~~~~~~~~~~~~~~~~~~~~~~#  
                                                                              
                                                                            **Uses general modules:** copy, glob, os, string, sys, time  
                                                                            **Uses RJE modules:** rje  
                                                                            **Other modules needed:** None

---


## \*\*\* Module qslimfinder \*\*\* [Top]

**Module:** qslimfinder  
**Description:** Query Short Linear Motif Finder  
**Version:** 1.0  
**Last Edit:** 29/05/09  
**Copyright (C) 2008 Richard J. Edwards - See source code for GNU License Notice**  
  
**Function:**  
QSLiMFinder is a modification of the basic SLiMFinder tool to specifically look for SLiMs shared by a query sequence  
and one or more additional sequences. To do this, SLiMBuild first identifies all motifs that are present in the query  
sequences before removing it (and its UPC) from the dataset. The rest of the search and stats takes place using the  
remainder of the dataset but only using motifs found in the query. The final correction for multiple testing is made  
using a motif space defined by the original query sequence, rather than the full potential motif space used by the  
original SLiMFinder. This is offset against the increased probability of the observed motif support values due to the  
reduction of support that results from removing the query sequence but could potentially still identify SLiMs will  
increased significance.  
  
Note that minocc and ambocc values \*include\* the query sequence, e.g. minocc=2 specifies the query and ONE other UPC.   
  
**Commandline:** ~~~~~~~~~~~~~~~~~~~~~~~~~~~~~~~~~~~~~~~~~~~~~~~~~~~~~~~~~~~~~~~~~~~~~~~~~~~~~~~~~~~~~~~~~~~~~~~~~~~~~~~~~~~#  
### Basic Input/Output Options ###   
- **seqin=FILE :** Sequence file to search [None]  
  - **batch=LIST :** List of files to search, wildcards allowed. (Over-ruled by seqin=FILE.) [\*.dat,\*.fas]  
    - **maxseq=X :** Maximum number of sequences to process [500]  
      - **maxupc=X :** Maximum UPC size of dataset to process [0]  
        - **walltime=X :** Time in hours before program will abort search and exit [1.0]  
          - **resfile=FILE :** Main SLiMFinder results table [slimfinder.csv]  
            - **resdir=PATH :** Redirect individual output files to specified directory (and look for intermediates) [SLiMFinder/]  
              - **buildpath=PATH :** Alternative path to look for existing intermediate files [SLiMFinder/]  
                - **force=T/F :** Force re-running of BLAST, UPC generation and SLiMBuild [False]  
                  - **pickup=T/F :** Pick-up from aborted batch run by identifying last dataset output in resfile [False]  
                    - **dna=T/F :** Whether the sequences files are DNA rather than protein [False]  
                      #~~~~~~~~~~~~~~~~~~~~~~~~~~~~~~~~~~~~~~~~~~~~~~~~~~~~~~~~~~~~~~~~~~~~~~~~~~~~~~~~~~~~~~~~~~~~~~~~~~~~~~~~~~~~~~~~~~~#  
                      ### SLiMBuild Options I: Evolutionary Filtering ###  
                      - **efilter=T/F :** Whether to use evolutionary filter [True]  
                        - **blastf=T/F :** Use BLAST Complexity filter when determining relationships [True]  
                          - **blaste=X :** BLAST e-value threshold for determining relationships [1e=4]  
                            - **altdis=FILE :** Alternative all by all distance matrix for relationships [None]  
                              - **gablamdis=FILE :** Alternative GABLAM results file [None] (!!!Experimental feature!!!)  
                                - **homcut=X :** Max number of homologues to allow (to reduce large multi-domain families) [0]  
                                    
                                  ### SLiMBuild Options II: Input Masking ###  
                                  - **masking=T/F :** Master control switch to turn off all masking if False [True]  
                                    - **dismask=T/F :** Whether to mask ordered regions (see rje\_disorder for options) [False]  
                                      - **consmask=T/F :** Whether to use relative conservation masking [False]  
                                        - **ftmask=LIST :** UniProt features to mask out [EM]  
                                          - **imask=LIST :** UniProt features to inversely ("inclusively") mask. (Seqs MUST have 1+ features) []  
                                            - **compmask=X,Y :** Mask low complexity regions (same AA in X+ of Y consecutive aas) [5,8]  
                                              - **casemask=X :** Mask Upper or Lower case [None]  
                                                - **motifmask=X :** List (or file) of motifs to mask from input sequences []  
                                                  - **metmask=T/F :** Masks the N-terminal M (can be useful if termini=T) [True]  
                                                    - **posmask=LIST :** Masks list of position-specific aas, where list = pos1:aas,pos2:aas [2:A]  
                                                      - **aamask=LIST :** Masks list of AAs from all sequences (reduces alphabet) []  
                                                          
                                                        ### SLiMBuild Options III: Basic Motif Construction ###  
                                                        - **termini=T/F :** Whether to add termini characters (^ & $) to search sequences [True]  
                                                          - **minwild=X :** Minimum number of consecutive wildcard positions to allow [0]  
                                                            - **maxwild=X :** Maximum number of consecutive wildcard positions to allow [2]  
                                                              - **slimlen=X :** Maximum length of SLiMs to return (no. non-wildcard positions) [5]  
                                                                - **minocc=X :** Minimum number of unrelated occurrences for returned SLiMs. (Proportion of UP if < 1) [0.05]  
                                                                  - **absmin=X :** Used if minocc<1 to define absolute min. UP occ [3]  
                                                                    - **alphahelix=T/F :** Special i, i+3/4, i+7 motif discovery [False]  
                                                                        
                                                                      ### SLiMBuild Options IV: Ambiguity ###  
                                                                      - **preamb=T/F :** Whether to search for ambiguous motifs during motif discovery [True]  
                                                                        - **ambocc=X :** Min. UP occurrence for subvariants of ambiguous motifs (minocc if 0 or > minocc) [0.05]  
                                                                          - **absminamb=X :** Used if ambocc<1 to define absolute min. UP occ [2]  
                                                                            - **equiv=LIST :** List (or file) of TEIRESIAS-style ambiguities to use [AGS,ILMVF,FYW,FYH,KRH,DE,ST]  
                                                                              - **wildvar=T/F :** Whether to allow variable length wildcards [True]  
                                                                                - **combamb=T/F :** Whether to search for combined amino acid degeneracy and variable wildcards [False]  
                                                                                    
                                                                                  ### SLiMBuild Options V: Advanced Motif Filtering ###  
                                                                                  - **musthave=LIST :** Returned motifs must contain one or more of the AAs in LIST (reduces search space) []  
                                                                                    - **query=LIST :** Return only SLiMs that occur in 1+ Query sequences (Name/AccNum) []  
                                                                                      - **focus=FILE :** FILE containing focal groups for SLiM return (see Manual for details) [None]  
                                                                                        - **focusocc=X :** Motif must appear in X+ focus groups (0 = all) [0]  
                                                                                          \* See also rje\_slimcalc options for occurrence-based calculations and filtering \*  
                                                                                            
                                                                                          #~~~~~~~~~~~~~~~~~~~~~~~~~~~~~~~~~~~~~~~~~~~~~~~~~~~~~~~~~~~~~~~~~~~~~~~~~~~~~~~~~~~~~~~~~~~~~~~~~~~~~~~~~~~~~~~~~~~#  
                                                                                          ### SLiMChance Options ###  
                                                                                          - **slimchance=T/F :** Execute main SLiMFinder probability method and outputs [True]  
                                                                                            - **probcut=X :** Probability cut-off for returned motifs [0.1]  
                                                                                              - **maskfreq=T/F :** Whether to use masked AA Frequencies (True), or (False) mask after frequency calculations [False]  
                                                                                                - **aafreq=FILE :** Use FILE to replace individual sequence AAFreqs (FILE can be sequences or aafreq) [None]  
                                                                                                  - **aadimerfreq=FILE:** Use empirical dimer frequencies from FILE (fasta or \*.aadimer.tdt) (!!!Experimental!!!) [None]  
                                                                                                    - **negatives=FILE :** Multiply raw probabilities by under-representation in FILE (!!!Experimental!!!) [None]  
                                                                                                      - **smearfreq=T/F :** Whether to "smear" AA frequencies across UPC rather than keep separate AAFreqs [False]  
                                                                                                        - **seqocc=T/F :** Whether to upweight for multiple occurrences in same sequence (heuristic) [False]  
                                                                                                          - **probscore=X :** Score to be used for probability cut-off and ranking (Prob/Sig) [Sig]  
                                                                                                            #~~~~~~~~~~~~~~~~~~~~~~~~~~~~~~~~~~~~~~~~~~~~~~~~~~~~~~~~~~~~~~~~~~~~~~~~~~~~~~~~~~~~~~~~~~~~~~~~~~~~~~~~~~~~~~~~~~~#  
                                                                                                            ### Advanced Output Options I: Output data ###  
                                                                                                            - **clouds=X :** Identifies motif "clouds" which overlap at 2+ positions in X+ sequences (0=minocc / -1=off) [2]  
                                                                                                              - **runid=X :** Run ID for resfile (allows multiple runs on same data) [DATE:TIME]  
                                                                                                                - **logmask=T/F :** Whether to log the masking of individual sequences [True]  
                                                                                                                  - **slimcheck=FILE :** Motif file/list to add to resfile output []   
                                                                                                                      
                                                                                                                    ### Advanced Output Options II: Output formats ###  
                                                                                                                    - **teiresias=T/F :** Replace TEIRESIAS, making \*.out and \*.mask.fasta files [False]  
                                                                                                                      - **slimdisc=T/F :** Emulate SLiMDisc output format (\*.rank & \*.dat.rank + TEIRESIAS \*.out & \*.fasta) [False]  
                                                                                                                        - **extras=X :** Whether to generate additional output files (alignments etc.) [1]  
                                                                                                                          - 0 = No output beyond main results file  
                                                                                                                          - 1 = Generate occurrence file, alignments and cloud file  
                                                                                                                          - 2 = Generate all additional SLiMFinder outputs  
                                                                                                                          - 3 = Generate SLiMDisc emulation too (equiv extras=2 slimdisc=T)  
                                                                                                                          - **targz=T/F :** Whether to tar and zip dataset result files (UNIX only) [False]  
                                                                                                                            - **savespace=0 :** Delete "unneccessary" files following run (best used with targz): [0]  
                                                                                                                              - 0 = Delete no files  
                                                                                                                              - 1 = Delete all bar \*.upc and \*.pickle files  
                                                                                                                              - 2 = Delete all dataset-specific files including \*.upc and \*.pickle (not \*.tar.gz)  
                                                                                                                                
                                                                                                                              ### Advanced Output Options III: Additional Motif Filtering ###   
                                                                                                                              - **topranks=X :** Will only output top X motifs meeting probcut [1000]  
                                                                                                                                - **minic=X :** Minimum information content for returned motifs [2.1]  
                                                                                                                                  \* See also rje\_slimcalc options for occurrence-based calculations and filtering \*  
                                                                                                                                    
                                                                                                                                  **Uses general modules:** copy, glob, math, os, string, sys, time  
                                                                                                                                  **Uses RJE modules:** slimfinder, rje, rje\_blast, rje\_slim, rje\_slimlist, rje\_slimcalc, rje\_slimcore, rje\_dismatrix\_V2,  
                                                                                                                                  rje\_seq, rje\_scoring  
                                                                                                                                  **Other modules needed:** None

---


## \*\*\* Module comparimotif\_V3 \*\*\* [Top]

**Module:** comparimotif  
**Description:** Motif vs Motif Comparison Software  
**Version:** 3.5  
**Last Edit:** 04/08/09  
**Copyright (C) 2007 Richard J. Edwards - See source code for GNU License Notice**  
  
**Function:**  
CompariMotif is a piece of software with a single objective: to take two lists of protein motifs and compare them to  
each other, identifying which motifs have some degree of overlap, and identifying the relationships between those  
motifs. It can be used to compare a list of motifs with themselves, their reversed selves, or a list of previously  
published motifs, for example (e.g. ELM (http://elm.eu.org/)). CompariMotif outputs a table of all pairs of matching  
motifs, along with their degree of similarity (information content) and their relationship to each other.  
  
The best match is used to define the relationship between the two motifs. These relationships are comprised of the  
following keywords:  
  
Match type keywords identify the type of relationship seen:  
\* Exact = all the matches in the two motifs are precise  
\* Variant = focal motif contains only exact matches and subvariants of degenerate positions in the other motif  
\* Degenerate = the focal motif contains only exact matches and degenerate versions of positions in the other motif  
\* Complex = some positions in the focal motif are degenerate versions of positions in the compared motif, while   
others are subvariants of degenerate positions.  
  
Match length keywords identify the length relationships of the two motifs:  
\* Match = both motifs are the same length and match across their entire length  
\* Parent = the focal motif is longer and entirely contains the compared motif  
\* Subsequence = the focal motif is shorter and entirely contained within the compared motif  
\* Overlap = neither motif is entirely contained within the other  
  
This gives sixteen possible classifications for each motif's relationship to the compared motif.  
  
**Input:**  
CompariMotif can take input in a number of formats. The preferred format is SLiMSearch format, while is a single line  
motif format: 'Name Sequence #Comments' (Comments are optional and ignored). Alternative inputs include SLiMDisc and  
Slim Pickings output, raw lists of motifs, and fasta format.  
  
**Output:**  
The main output for CompariMotif is delimited text file containing the following fields:  
\* File1 = Name of motifs file (if outstyle=multi)  
\* File2 = Name of searchdb file (if outstyle=multi)  
\* Name1 = Name of motif from motif file 1   
\* Name2 = Name of motif from motif file 2   
\* Motif1 = Motif (pattern) from motif file 1  
\* Motif2 = Motif (pattern) from motif file 2  
\* Sim1 = Description of motif1's relationship to motif2  
\* Sim2 = Description of motif2's relationship to motif1  
\* Match = Text summary of matched region  
\* MatchPos = Number of matched positions between motif1 and motif2 (>= mishare=X)  
\* MatchIC = Information content of matched positions  
\* NormIC = MatchA as a proportion of the maximum possible MatchA  
\* Score = Heuristic score (MatchPos x NormIC) for ranking motif matches  
\* Info1 = Ambiguity score of motif1   
\* Info2 = Ambiguity score of motif2   
\* Desc1 = Description of motif1 (if motdesc = 1 or 3)  
\* Desc2 = Description of motif2 (if motdesc = 2 or 3)  
  
With the exception of the file names, which are only output if outstyle=multi, the above is the output for the  
default "normal" output style. If outstyle=single then only statistics for motif2 (the searchdb motif) are output  
as this is designed for searches using a single motif against a motif database. If outstyle=normalsplit or  
outstyle=multisplit then motif1 information is grouped together, followed by motif2 information, followed by the  
match statistics. More information can be found in the CompariMotif manual.  
  
**Commandline:**  
## Basic Input Parameters ##  
- **motifs=FILE :** File of input motifs/peptides [None]  
  - **searchdb=FILE :** (Optional) second motif file to compare. Will compare to self if none given. [None]  
    - **dna=T/F :** Whether motifs should be considered as DNA motifs [False]  
        
      ## Basic Output Parameters ##  
      - **resfile=FILE :** Name of results file, FILE.compare.txt. [motifsFILE-searchdbFILE.compare.txt]  
        - **motinfo=FILE :** Filename for output of motif summary table (if desired) [None]  
          - **motific=T/F :** Output Information Content for motifs [False]  
              
            ## Motif Comparison Parameters ##  
            - **minshare=X :** Min. number of non-wildcard positions for motifs to share [2]  
              - **normcut=X :** Min. normalised MatchIC for motif match [0.5]  
                - **matchfix=X :** If >0 must exactly match \*all\* fixed positions in the motifs from: [0]  
                  - 1: input (motifs=FILE) motifs  
                  - 2: searchdb motifs  
                  - 3: \*both\* input and searchdb motifs  
                  - **ambcut=X :** Max number of choices in ambiguous position before replaced with wildcard (0=use all) [10]  
                      
                    ## Advanced Motif Input Parameters ##  
                    - **minic=X :** Min information content for a motif (1 fixed position = 1.0) [2.0]  
                      - **minfix=X :** Min number of fixed positions for a motif to contain [0]  
                        - **minpep=X :** Min number of defined positions in a motif [2]  
                          - **trimx=T/F :** Trims Xs from the ends of a motif [False]  
                            - **nrmotif=T/F :** Whether to remove redundancy in input motifs [False]  
                              - **reverse=T/F :** Reverse the input motifs. [False]  
                                - If no searchdb given, these will be searched against the "forward" motifs.  
                                - **mismatches=X :** <= X mismatches of positions can be tolerated [0]  
                                  - **aafreq=FILE :** Use FILE to replace uniform AAFreqs (FILE can be sequences or aafreq) [None]   
                                      
                                    ## Advanced Motif Output Parameters ##  
                                    - **xgmml=T/F :** Whether to output XGMML format results [True]  
                                      - **pickle=T/F :** Whether to load/save pickle following motif loading/filtering [False]  
                                        - **motdesc=X :** Sets which motifs have description outputs (0-3 as matchfix option) [3]  
                                          - **outstyle=X :** Sets the output style for the resfile [normal]  
                                            - normal = all standard stats are output  
                                            - multi = designed for multiple appended runs. File names are also output  
                                            - single = designed for searches of a single motif vs a database. Only motif2 stats are output  
                                            - reduced = motifs do not have names or descriptions  
                                            - normalsplit/multisplit = as normal/multi but stats are grouped by motif rather than by type  
                                              
                                            **Uses general modules:** os, string, sys, time  
                                            **Uses RJE modules:** rje, rje\_menu, rje\_slim, rje\_slimlist, rje\_xgmml, rje\_zen  
                                            **Other modules needed:** rje\_aaprop, rje\_dismatrix, rje\_seq, rje\_blast, rje\_pam, rje\_sequence, rje\_uniprot

---


## \*\*\* Module gopher\_V2 \*\*\* [Top]

**Module:** gopher  
**Description:** Generation of Orthologous Proteins from High-Throughput Estimation of Relationships  
**Version:** 2.5  
**Last Edit:** 05/02/09  
**Copyright (C) 2005 Richard J. Edwards - See source code for GNU License Notice**  
  
**Function:**  
This script is designed to take in two sequences files and generate datasets of orthologous sequence alignments.  
The first [seqin] sequence set is the 'queries' around which orthologous datasets are to be assembled. This is now  
optimised for a dataset consisting of one protein per protein-coding gene, although splice variants should be dealt  
with OK and treated as paralogues. This will only cause problems if the postdup=T option is used, which restricts  
orthologues returned to be within the last post-duplication clade for the sequence.  
  
The second [orthdb] is the list of proteins from which the orthologues will be extracted. The seqin sequences are  
then BLASTed against the orthdb and processed (see below) to retain putative orthologues using an estimation of the  
phylogenetic relationships based on pairwise sequences similarities.  
  
NB. As of version 2.0, gopher=FILE has been replaced with seqin=FILE for greater rje python consistency. The allqry  
option has been removed. Please cleanup the input data into a desired non-redundant dataset before running GOPHER.  
(In many ways, GOPHER's strength is it's capacity to be run for a single sequence of interest rather than a whole  
genome, and it is this functionality that has been concentrated on for use with PRESTO and SLiM Pickings etc.) The  
output of statistics for each GOPHER run has also been discontinued for now but may be reintroduced with future  
versions. The phosalign command (to produce a table of potential phosphorylation sites (e.g. S,T,Y) across  
orthologues for special conservation of phosphorylation prediction analyses) has also been discontinued for now.  
  
Version 2.1 has tightened up on the use of rje\_seq parameters that were causing trouble otherwise. It is now the  
responsibility of the user to make sure that the orthologue database meets the desired criteria. Duplicate accession  
numbers will not be tolerated by GOPHER and (arbitrary) duplicates will be deleted if the sequences are the same, or  
renamed otherwise. Renaming may cause problems later. It is highly desirable not to have two proteins with the same  
accession number but different amino acid sequences. The following commands are added to the rje\_seq object when input  
is read: accnr=T unkspec=F specnr=F gnspacc=T. Note that unknown species are also not permitted.  
  
The process for dataset assembly is as follows for each protein :  
  
1. BLAST against orthdb [orthblast]  
> BLASTs saved in BLAST/AccNum.blast  
2. Work through BLAST hits, indentifying paralogues (query species duplicates) and the closest homologue from each  
other species. This involves a second BLAST of the query versus original BLAST hits (e-value=10, no complexity  
filter). The best sequence from each species is kept, i.e. the one with the best similarity to the query and not part  
of a clade with any paralogue that excludes the query. (If postdup=T, the hit must be in the query's post duplication  
clade.) In addition hits: [orthfas]  
\* Must have minimum identity level with Query  
\* Must be one of the 'good species' [goodspec=LIST]  
> Save reduced sequences as ORTH/AccNum.orth.fas  
> Save paralogues identified (and meeting minsim settings) in PARA/AccNum.para.fas  
3. Align sequences with MUSCLE [orthalign]  
> ALN/AccNum.orthaln.fas  
4. Generate an unrooted tree with (ClustalW or PHYLIP) [orthtree]  
> TREE/AccNum.orth.nsf  
  
Optional paralogue/subfamily output: (These are best not used with Force=T or FullForce=T)  
2a. Alignment of query protein and any paralogues >minsim threshold (paralign=T/F). The parasplice=T/F controls  
whether splice variants are in these paralogue alignments (where identified using AccNum-X notation).  
> PARALN/AccNum.paraln.fas  
2b. Pairwise combinations of paralogues and their orthologues aligned, with "common" orthologues removed from the  
dataset, with a rooted tree and group data for BADASP analysis etc. (parafam=T)  
> PARAFAM/AccNum+ParaAccNum.parafam.fas  
> PARAFAM/AccNum+ParaAccNum.parafam.nsf  
> PARAFAM/AccNum+ParaAccNum.parafam.grp  
2c. Combined protein families consisting of a protein, all the paralogues > minsim and all orthologues for each in a  
single dataset. Unaligned. (gopherfam=T)  
> SUBFAM/AccNum.subfam.fas  
\*NB.\* The subfamily outputs involve Gopher calling itself to ensure the paralogues have gone through the Gopher  
process themselves. This could potentially cause conflict if forking is used.  
  
**Commandline:**  
### Basic Input/Output ###  
- **seqin=FILE :** Fasta file of 'query' sequences for orthology discovery []  
  - **orthdb=FILE :** Fasta file with pool of sequences for orthology discovery []. Should contain query sequences.  
    - **startfrom=X :** Accession Number / ID to start from. (Enables restart after crash.) [None]  
      - **dna=T/F :** Whether to analyse DNA sequences (not optimised) [False]  
          
        ### GOPHER run control parameters ###  
        orthblast : Run to blasting versus orthdb (Stage 1).  
        orthfas : Run to output of orthologues (Stage 2).   
        orthalign : Run to alignment of orthologues (Stage 3).  
        orthtree : Run to tree-generation (Stage 4). [default!]  
          
        ### GOPHER Orthologue identifcation Parameters ###  
        - **postdup=T/F :** Whether to align only post-duplication sequences [False]  
          - **minsim=X :** Minimum %similarity of Query for each "orthologue" [40.0]  
            - **simfocus=X :** Style of similairy comparison used for MinSim and "Best" sequence identification [query]  
              - query = %query must > minsim (Best if query is ultimate focus and maximises closeness of returned orthologues)  
              - hit = %hit must > minsim (Best if lots of sequence fragments are in searchdb and should be retained)  
              - either = %query > minsim OR %hit > minsim (Best if both above conditions are true)  
              - both = %query > minsim AND %hit > minsim (Most stringent setting)  
              - **gablamo=X :** GABLAMO measure to use for similarity measures [Sim]  
                - ID = %Identity (from BLAST)  
                - Sim = %Similarity (from BLAST)  
                - Len = %Coverage (from BLAST)  
                - **goodX=LIST :** Filters where only sequences meeting the requirement of LIST are kept.  
                  LIST may be a list X,Y,..,Z or a FILE which contains a list [None]  
                  - goodacc = list of accession numbers  
                  - goodseq = list of sequence names  
                  - goodspec = list of species codes  
                  - gooddb = list of source databases  
                  - gooddesc = list of terms that, at least one of which must be in description line  
                  - **badX=LIST :** As goodX but excludes rather than retains filtered sequences  
                      
                    ### Additional run control options ###  
                    - **repair=T/F :** Repair mode - replace previous files if date mismatches or files missing.  
                      (Skip missing files if False) [True]  
                      - **force=T/F :** Whether to force execution at current level even if results are new enough [False]  
                        - **fullforce=T/F :** Whether to force current and previous execution even if results are new enough [False]  
                          - **ignoredate=T/F :** Ignores the age of files and only replaces if missing [False]  
                            - **savespace=T/F :** Save space by deleting intermediate blast files during orthfas [True]  
                                
                              ### Additional Output Options ###  
                              - **paralign=T/F :** Whether to produce paralogue alignments (>minsim) in PARALN/ (assuming run to orthfas+) [False]  
                                - **parasplice=T/F :** Whether splice variants (where identified) are counted as paralogues [False]  
                                  - **parafam=T/F :** Whether to paralogue paired subfamily alignments (>minsim) (assuming run to orthfas+) [False]  
                                    - **gopherfam=T/F :** Whether to combined paralogous gopher orthologues into protein families (>minsim) (assuming run to orthfas+) [False]  
                                        
                                      **Uses general modules:** copy, gc, glob, os, string, sys, threading, time  
                                      **Uses RJE modules:** rje, rje\_blast, rje\_dismatrix, rje\_seq, rje\_tree  
                                      **Other modules needed:** rje\_ancseq, rje\_pam, rje\_sequence, rje\_tree\_group, rje\_uniprot

---


## \*\*\* Module ned\_eigenvalues \*\*\* [Top]

**Module:** ned\_eigenvalues  
**Description:** Modified N. Davey Relative Local Conservation module  
**Version:** 1.0  
**Last Edit:** 03/09/09  
**Copyright (C) 2009 Norman E. Davey & Richard J. Edwards - See source code for GNU License Notice**  
  
**Function:**  
This module is not for standalone running and has no commandline options (including 'help'). All options are handled  
by the parent module.  
  
**Uses general modules:** operator, math, random

---


## \*\*\* Module ned\_rankbydistribution \*\*\* [Top]

**Module:** ned\_rankbydistribution  
**Description:** Modified SLiMFinder stats module  
**Version:** 1.0  
**Last Edit:** 03/09/09  
**Copyright (C) 2009 Norman E. Davey & Richard J. Edwards - See source code for GNU License Notice**  
  
**Function:**  
This module is a stripped down template for methods only. This is for when a class has too many methods and becomes  
untidy. In this case, methods can be moved into a methods module and 'self' replaced with the relevant object.  
  
**Commandline:**  
This module is not for standalone running and has no commandline options (including 'help'). All options are handled  
by the parent module.  
  
**Uses general modules:** re, copy, random, math, sys, time, os, pickle, sets, string, traceback  
**Uses RJE modules:** rje\_seq, rje\_uniprot, rje, rje\_blast, rje\_slim

---


## \*\*\* Module rje \*\*\* [Top]

**Module:** rje  
**Description:** Contains General Objects for all my (Rich's) scripts  
**Version:** 3.9  
**Last Edit:** 31/10/08  
**Copyright (C) 2005 Richard J. Edwards - See source code for GNU License Notice**  
  
**Function:**  
General module containing Classes used by all my scripts plus a number of miscellaneous methods.  
- Output to Screen, Commandline parameters and Log Files  
  
Commandline options are all in the form X=Y. Where Y is to include spaces, use X="Y".  
  
**General Commandline:**  
- **v=X :** Sets verbosity (-1 for silent) [0]  
  - **i=X :** Sets interactivity (-1 for full auto) [0]  
    - **log=FILE :** Redirect log to FILE [Default = calling\_program.log]  
      - **newlog=T/F :** Create new log file. [Default = False: append log file]  
        - **silent=T/F :** If set to True will not write to screen or log. (Log object attribute only.) [False]  
          help : Print help to screen  
            
          **Program-Specific Commands:** (Some programs only)  
          - **basefile=FILE :** This will set the 'root' filename for output files (FILE.\*), including the log  
            - **outfile=FILE :** This will set the 'root' filename for output files (FILE.\*), excluding the log  
              - **delimit=X :** Sets standard delimiter for results output files [\t]  
                - **mysql=T/F :** MySQL output  
                  - **append=T/F :** Append to results files rather than overwrite [False]  
                    - **force=T/F :** Force to regenerate data rather than keep old results [False]  
                      - **backups=T/F :** Whether to generate backup files (True) or just overwrite without asking (False) [True]  
                        - **maxbin=X :** Maximum number of trials for using binomial (else use Poisson) [-]  
                            
                          **System Commandline:**  
                          - **win32=T/F :** Run in Win32 Mode [False]  
                            pwin : Run in PythonWin (\*\* Must be 'commandline', not in ini file! \*\*)  
                            cerberus : Run on Cerberus cluster at RCSI  
                            - **memsaver=T/F :** Some modules will have a memsaver option to save memory usage [False]  
                              - **runpath=PATH :** Run program from given path (log files and some programs only) [path called from]  
                                - **rpath=PATH :** Path to installation of R ['c:\\Program Files\\R\\R-2.6.2\\bin\\R.exe']  
                                  - **soaplab=T/F :** Implement special options/defaults for SoapLab implementations [False]  
                                      
                                    **Forking Commandline:**  
                                    - **noforks=T/F :** Whether to avoid forks [False]  
                                      - **forks=X :** Number of parallel sequences to process at once [0]  
                                        - **killforks=X :** Number of seconds of no activity before killing all remaining forks. [3600]  
                                            
                                          **Classes:**  
                                          RJE\_Object(log=None,cmd\_list=[]):  
                                          - Metclass for inheritance by other classes.  
                                          >> log:Log = rje.Log object  
                                          >> cmd\_list:List = List of commandline variables  
                                          On intiation, this object:  
                                          - sets the Log object (if any)  
                                          - sets verbosity and interactive attributes  
                                          - calls the \_setAttributes() method to setup class attributes  
                                          - calls the \_cmdList() method to process relevant Commandline Parameters   
                                          Log(itime=time.time(),cmd\_list=[]):  
                                          - Handles log output; printing to log file and error reporting  
                                          >> itime:float = initiation time  
                                          >> cmd\_list:list of commandline variables  
                                          Info(prog='Unknown',vers='X',edit='??/??/??',desc='Python script',author='Unknown',ptime=None):  
                                          - Stores intro information for a program.  
                                          >> prog:str = program name  
                                          >> vers:str = version number  
                                          >> edit:str = last edit date  
                                          >> desc:str = program description  
                                          >> author:str = author name  
                                          >> ptime:float = starting time of program, time.time()  
                                          Out(cmd=[]):  
                                          - Handles basic generic output to screen based on Verbosity and Interactivity for modules without classes.  
                                          >> cmd:list = list of command-line arguments  
                                            
                                          **Uses general modules:** glob, math, os, random, re, string, sys, time, traceback

---


## \*\*\* Module rje\_aaprop \*\*\* [Top]

**Module:** rje\_aaprop  
**Description:** AA Property Matrix Module  
**Version:** 0.1  
**Last Edit:** 18/05/06  
**Copyright (C) 2005 Richard J. Edwards - See source code for GNU License Notice**  
  
**Function:**  
Takes an amino acid property matrix file and reads into an AAPropMatrix object. Converts in an all by all property  
difference matrix. By default, gaps and Xs will be given null properties (None) unless part of input file.  
  
**Commandline:**  
- **aaprop=FILE :** Amino Acid property matrix file. [aaprop.txt]  
  - **aagapdif=X :** Property difference given to amino acid vs gap comparisons [5]  
    - **aanulldif=X :** Property difference given to amino acid vs null values (e.g. X) [0.5]  
        
      **Uses general modules:** re, string, sys, time  
      **Uses RJE modules:** rje

---


## \*\*\* Module rje\_ancseq \*\*\* [Top]

**Module:** rje\_ancseq  
**Description:** Ancestral Sequence Prediction Module  
**Version:** 1.2  
**Last Edit:** 08/01/07  
**Copyright (C) 2005 Richard J. Edwards - See source code for GNU License Notice**  
  
**Function:**  
This module contains the objects and methods for ancestral sequence prediction. Currently, only GASP (Edwards & Shields  
2004) is implemented. Other methods may be incorporated in the future.  
  
**GASP Commandline:**  
- **fixpam=X\t:** PAM distance fixed to X [0].  
  - **rarecut=X\t:** Rare aa cut-off [0.05].  
    - **fixup=T/F\t:** Fix AAs on way up (keep probabilities) [True].  
      - **fixdown=T/F\t:** Fix AAs on initial pass down tree [False].  
        - **ordered=T/F\t:** Order ancestral sequence output by node number [False].  
          - **pamtree=T/F\t:** Calculate and output ancestral tree with PAM distances [True].  
            - **desconly=T/F\t:** Limits ancestral AAs to those found in descendants [True].  
              - **xpass=X\t:** How many extra passes to make down & up tree after initial GASP [1].  
                  
                **Classes:**  
                Gasp(log=None,cmd\_list=[],tree=None,ancfile='gasp'):  
                - Handles main GASP algorithm.  
                >> log:Log = rje.Log object  
                >> cmd\_list:List = List of commandline variables  
                >> tree:Tree = rje\_tree.Tree Object  
                >> ancfile:str = output filename (basefile))   
                GaspNode(realnode,alphabet,log):  
                - Used by Gasp Class to handle specific node data during GASP.  
                >> realnode:Node Object (rje\_tree.py)  
                >> alphabet:list of amino acids for use in GASP  
                >> log:Log Object  
                  
                **Uses general modules:** copy, sys, time  
                **Uses RJE modules:** rje, rje\_pam

---


## \*\*\* Module rje\_blast \*\*\* [Top]

**Module:** rje\_blast  
**Description:** BLAST Control Module  
**Version:** 1.10  
**Last Edit:** 09/06/09  
**Copyright (C) 2005 Richard J. Edwards - See source code for GNU License Notice**  
  
**Function:**  
Performs BLAST searches and loads results into objects. Peforms GABLAM conversion of local alignments into global  
alignment statistics.   
  
**Objects:**  
BLASTRun = Full BLAST run  
BLASTSearch = Information for a single Query search within a BLASTRun  
BLASTHit = Detailed Information for a single Query-Hit pair within BLASTRun  
PWAln = Detailed Information for each aligned section of a Query-Hit Pair  
  
**Commandline:**  
- **blastpath=X :** path for blast files [c:/bioware/blast/] \*Use fwd slashes (\*Cerberus is special!)  
    
  - **blastp=X :** BLAST program (BLAST -p X) [blastp]  
    - **blasti=FILE :** Input file (BLAST -i FILE) [None]  
      - **blastd=FILE :** BLAST database (BLAST -d FILE) [None]  
        - **formatdb=T/F :** Whether to (re)format BLAST database [False]  
          - **blasto=FILE :** Output file (BLAST -o FILE) [\*.blast]  
              
            - **blaste=X :** E-Value cut-off for BLAST searches (BLAST -e X) [1e-4]  
              - **blastv=X :** Number of one-line hits per query (BLAST -v X) [500]  
                - **blastb=X :** Number of hit alignments per query (BLAST -b X) [250]   
                    
                  - **blastf=T/F :** Complexity Filter (BLAST -F X) [True]  
                    - **blastcf=T/F :** Use BLAST Composition-based statistics (BLAST -C X) [False]  
                      - **blastg=T/F :** Gapped BLAST (BLAST -g X) [True]  
                          
                        - **blastopt=FILE :** File containing raw BLAST options (applied after all others) []   
                            
                          **Uses general modules:** os, re, string, sys, time  
                          **Uses RJE modules:** rje

---


## \*\*\* Module rje\_dismatrix\_V2 \*\*\* [Top]

**Module:** rje\_dismatrix  
**Description:** Distance Matrix Module   
**Version:** 2.3  
**Last Edit:** 09/04/08  
**Copyright (C) 2007 Richard J. Edwards - See source code for GNU License Notice**  
  
**Function:**  
DisMatrix Class. Stores distance matrix data and contains methods for extra calculations, such as MST. If pylab is  
installed, a distance matrix can also be turned into a heatmap.  
  
**Commandline:**  
- **loadmatrix=FILE :** Loads a matrix from FILE [None]  
  - **symmetric=T/F :** Whether the matrix should be symmetrical (e.g. DisAB = DisBA) [False]  
    - **outmatrix=X :** Type for output matrix - text / mysql / phylip  
        
      **Uses general modules:** copy, os, re, string, sys, time  
      **Uses RJE modules:** rje  
      **Other modules needed:** None

---


## \*\*\* Module rje\_disorder \*\*\* [Top]

**Module:** rje\_disorder  
**Description:** Disorder Prediction Module  
**Version:** 0.5  
**Last Edit:** 16/05/08  
**Copyright (C) 2006 Richard J. Edwards - See source code for GNU License Notice**  
  
**Function:**  
This module currently has limited function and no standalone capability, though this may be added with time. It is  
designed for use with other modules. The disorder Class can be given a sequence and will run the appropriate  
disorder prediction software and store disorder prediction results for use in other programs. The sequence will have  
any gaps removed.  
  
Currently two disorder prediction methods are implemented:  
\* IUPred : Dosztanyi Z, Csizmok V, Tompa P & Simon I (2005). J. Mol. Biol. 347, 827-839. This has to be installed  
locally. It is available on request from the IUPred website and any use of results should cite the method. (See  
http://iupred.enzim.hu/index.html for more details.) IUPred returns a value for each residue, which by default,  
is determined to be disordered if > 0.5.  
\* FoldIndex : This is run directly from the website (http://bioportal.weizmann.ac.il/fldbin/findex) and more simply  
returns a list of disordered regions. You must have a live web connection to use this method!  
  
For IUPred, the individual residue results are stored in Disorder.list['ResidueDisorder']. For both methods, the  
disordered regions are stored in Disorder.list['RegionDisorder'] as (start,stop) tuples.  
  
**Commandline:**  
### General Options ###  
- **disorder=X :** Disorder method to use (iupred/foldindex/parse) [iupred]  
  - **iucut=X :** Cut-off for IUPred results [0.2]  
    - **iumethod=X :** IUPred method to use (long/short) [short]  
      - **sequence=X :** Sequence to predict disorder for (autorun) []  
        - **name=X :** Name of sequence to predict disorder for []  
          - **minregion=X :** Minimum length of an ordered/disordered region [0]  
              
            ### System Settings ###  
            - **iupath=PATH :** The full path to the IUPred exectuable [c:/bioware/iupred/iupred.exe]  
              - **filoop=X :** Number of times to try connecting to FoldIndex server [10]  
                - **fisleep=X :** Number of seconds to sleep between attempts [2]  
                  - **iuchdir=T/F :** Whether to change to IUPred directory and run (True) or rely on IUPred\_PATH env variable [False]  
                      
                    **Uses general modules:** copy, os, string, sys, time, urllib2  
                    **Uses RJE modules:** rje  
                    **Other modules needed:** None

---


## \*\*\* Module rje\_hmm \*\*\* [Top]

**Module:** rje\_hmm  
**Description:** HMMer Control Module  
**Version:** 1.3  
**Last Edit:** 25/11/08  
**Copyright (C) 2005 Richard J. Edwards - See source code for GNU License Notice**  
  
**Function:**  
This module is designed to perform basic HMM functions using the HMMer program. Currently, there are three functions  
that may be performed, separately or consecutively:  
\* 1. Use hmmbuild to construct HMMs from input sequence files  
\* 2. Search a sequence database with HMMs files  
\* 3. Convert HMMer output into a delimited text file of results.  
  
**Commandline:**  
## Build Options ##  
- **makehmm=LIST :** Sequence file(s). Can include wildcards [None]  
  - **hmmcalibrate=T/F :** Whether to calibrate HMM files once made [True]  
      
    ## Search Options ##   
    - **hmm=LIST :** HMM file(s). Can include wildcards. [\*.hmm]  
      - **searchdb=FILE :** Fasta file to search with HMMs [None]  
        - **hmmoptions=LIST :** List or file of additional HMMer search options (joined by whitespace) []  
          - **hmmpfam=T/F :** Performs standard HMMer PFam search (--cut\_ga) (or processes if present) [False]  
            - **hmmout=FILE :** Pipe results of HMM searches into FILE [None]  
              - **hmmres=LIST :** List of HMM search results files to convert (wildcards allowed) []  
                - **hmmtab=FILE :** Delimited table of results ('None' to skip) [searchdb.tdt]  
                  - **cleanres=T/F :** Option to reduce size of HMM results file by removing no-hit sequences [True]  
                      
                    ## System Parameters ##  
                    - **hmmerpath=PATH :** Path for hmmer files [/home/richard/Bioware/hmmer-2.3.2/src/]   
                      - **force=T/F :** Whether to force regeneration of new HMMer results if already existing [False]  
                        - **gzip=T/F :** Whether to gzip (and gunzip) HMMer results files (not Windows) [True]  
                            
                          **Classes:**  
                          HMMRun Object = Full HMM run  
                          HMMSearch Object = Information for a single Query search within a BLASTRun  
                          HMMHit Object = Detailed Information for a single Query-Hit pair within BLASTRun  
                          rje\_blast.PWAln Object = Detailed Information for each aligned section of a Query-Hit Pair  
                            
                          **Uses general modules:** glob, os, re, string, sys, time  
                          **Uses RJE modules:** rje, rje\_blast

---


## \*\*\* Module rje\_menu \*\*\* [Top]

**Module:** rje\_menu  
**Description:** Generic Menu Methods Module  
**Version:** 0.2  
**Last Edit:** 20/08/09  
**Copyright (C) 2006 Richard J. Edwards - See source code for GNU License Notice**  
  
**Function:**  
This module is designed to contain generic menu methods for use with any RJE Object. At least, that's the plan...  
  
**Commandline:**  
This module is not for standalone running and has no commandline options (including 'help'). All options are handled  
by the parent module.  
  
**Uses general modules:** os, string, sys  
**Uses RJE modules:** rje  
**Other modules needed:** None

---


## \*\*\* Module rje\_motif\_V3 \*\*\* [Top]

**Module:** rje\_motif  
**Description:** Motif Class and Methods Module  
**Version:** 3.0  
**Last Edit:** 15/02/07  
**Copyright (C) 2006 Richard J. Edwards - See source code for GNU License Notice**  
  
**Function:**  
This module contains the Motif class for use with both Slim Pickings and PRESTO, and associated methods. This basic  
Motif class stores its pattern in several forms:  
- info['Sequence'] stores the original pattern given to the Motif object  
- list['PRESTO'] stores the pattern in a list of PRESTO format elements, where each element is a discrete part of  
the motif pattern  
- list['Variants'] stores simple strings of all the basic variants - length and ambiguity - for indentifying the "best"  
variant for any given match  
- dict['Search'] stores the actual regular expression variants used for searching, which has a separate entry for  
each length variant - otherwise Python RegExp gets confused! Keys for this dictionary relate to the number of  
mismatches allowed in each variant.  
  
The Motif Class is designed for use with the MotifList class. When a motif is added to a MotifList object, the  
Motif.format() command is called, which generates the 'PRESTO' list. After this - assuming it is to be kept -  
Motif.makeVariants() makes the 'Variants' list. If creating a motif object in another module, these method should be  
called before any sequence searching is performed. If mismatches are being used, the Motif.misMatches() method must  
also be called.  
  
**Commandline:**  
These options should be listed in the docstring of the module using the motif class:  
-- **alphabet=LIST :** List of letters in alphabet of interest [AAs]  
  -- **ambcut=X :** Cut-off for max number of choices in ambiguous position to be shown as variant (0=All) [10]  
    -- **trimx=T/F :** Trims Xs from the ends of a motif [False]  
        
      **Uses general modules:** copy, math, os, re, string, sys  
      **Uses RJE modules:** rje  
      **Other modules needed:** None

---


## \*\*\* Module rje\_motif\_stats \*\*\* [Top]

**Module:** rje\_motif\_stats  
**Description:** Motif Statistics Methods Module  
**Version:** 1.0  
**Last Edit:** 01/02/07  
**Copyright (C) 2007 Richard J. Edwards - See source code for GNU License Notice**  
  
**Function:**  
This module contains the Alignment Conservation methods for motifs, as well as other calculations needing occurrence  
data. This module is designed to be used by the MotifList class, which contains the relevant commandline options.  
  
**Commandline:**  
This module is not for standalone running and has no commandline options (including 'help'). All options are handled  
by the parent module.  
  
**Uses general modules:** copy, os, string, sys  
**Uses RJE modules:** gopher\_V2, rje, rje\_blast, rje\_disorder, rje\_motif\_V3, rje\_seq, rje\_sequence  
**Other modules needed:** rje\_seq modules

---


## \*\*\* Module rje\_motiflist \*\*\* [Top]

**Module:** rje\_motiflist  
**Description:** RJE Motif List Module  
**Version:** 1.0  
**Last Edit:** 03/04/07  
**Copyright (C) 2007 Richard J. Edwards - See source code for GNU License Notice**  
  
**Function:**  
This module contains the MotifList Class, which is designed to replace many of the functions that previously formed  
part of the Presto Class. This class will then be used by PRESTO, SLiMPickings and CompariMotif (and others?) to  
control Motif loading, redundancy and storage. MotifOcc objects will replace the previous PrestoSeqHit objects and  
contain improved data commenting and retrieval methods. The MotifList class will contain methods for filtering motifs  
according to individual or combined MotifOcc data.  
  
The options below should be read in by the MotifList object when it is instanced with a cmd\_list and therefore do not  
need to be part of any class that makes use of this object unless it has conflicting settings.  
  
The Motif Stats options are used by MotifList to calculate statistics for motif occurrences, though this data will  
actually be stored in the MotifOcc objects themselves. This includes conservation statistics.  
  
Note. Additional output parameters, such as motifaln and proteinaln settings, and stat filtering/novel scores are not  
stored in this object, as they will be largely dependent on the main programs using the class, and the output from  
those programs. (This also enables statfilters etc. to be used with stats not related to motifs and their occurrences  
if desired.)  
  
**MotifList Commands:**  
## Basic Motif Input/Formatting Parameters ##  
- **motifs=FILE :** File of input motifs/peptides [None]  
  Single line per motif format = 'Name Sequence #Comments' (Comments are optional and ignored)  
  Alternative formats include fasta, SLiMDisc output and raw motif lists.  
  - **minpep=X :** Min length of motif/peptide X aa [2]  
    - **minfix=X :** Min number of fixed positions for a motif to contain [0]  
      - **minic=X :** Min information content for a motif (1 fixed position = 1.0) [2.0]  
        - **trimx=T/F :** Trims Xs from the ends of a motif [False]  
          - **nrmotif=T/F :** Whether to remove redundancy in input motifs [False]  
            - **minimotif=T/F :** Input file is in minimotif format and will be reformatted (PRESTO File format only) [False]  
              - **goodmotif=LIST :** List of text to match in Motif names to keep (can have wildcards) []  
                - **ambcut=X :** Cut-off for max number of choices in ambiguous position to be shown as variant [10]  
                  - **reverse=T/F :** Reverse the motifs - good for generating a test comparison data set [False]  
                    - **msms=T/F :** Whether to include MSMS ambiguities when formatting motifs [False]  
                        
                      ## Motif Occurrence Statistics Options ##  
                      - **winsa=X :** Number of aa to extend Surface Accessibility calculation either side of motif [0]  
                        - **winhyd=X :** Number of aa to extend Eisenberg Hydrophobicity calculation either side of motif [0]  
                          - **windis=X :** Extend disorder statistic X aa either side of motif (use flanks \*only\* if negative) [0]  
                            - **winchg=X :** Extend charge calculations (if any) to X aa either side of motif [0]  
                              - **winsize=X :** Sets all of the above window sizes (use flanks \*only\* if negative) [0]  
                                - **slimchg=T/F :** Calculate Asolute, Net and Balance charge statistics (above) for occurrences [False]  
                                  - **iupred=T/F :** Run IUPred disorder prediction [False]  
                                    - **foldindex=T/F :** Run FoldIndex disorder prediction [False]  
                                      - **iucut=X :** Cut-off for IUPred results (0.0 will report mean IUPred score) [0.0]  
                                        - **iumethod=X :** IUPred method to use (long/short) [short]  
                                          - **domfilter=FILE :** Use the DomFilter options, reading domains from FILE [None] ?? Check how this works ??  
                                            - **ftout=T/F :** Make a file of UniProt features for extracted parent proteins, where possible, incoroprating SLIMs [\*.features.tdt]  
                                              - **percentile=X :** Percentile steps to return in addition to mean [0]  
                                                  
                                                ## Conservation Parameters ## ??? Add separate SlimCons option ???  
                                                - **usealn=T/F :** Whether to search for and use alignemnts where present. [False]  
                                                  - **gopher=T/F :** Use GOPHER to generate missing orthologue alignments in alndir - see gopher.py options [False]  
                                                    - **alndir=PATH :** Path to alignments of proteins containing motifs [./] \* Use forward slashes (/)  
                                                      - **alnext=X :** File extension of alignment files, accnum.X [aln.fas]  
                                                        - **alngap=T/F :** Whether to count proteins in alignments that have 100% gaps over motif (True) or (False) ignore  
                                                          as putative sequence fragments [False] (NB. All X regions are ignored as sequence errors.)  
                                                          - **conspec=LIST :** List of species codes for conservation analysis. Can be name of file containing list. [None]  
                                                            - **conscore=X :** Type of conservation score used: [pos]  
                                                              - abs = absolute conservation of motif using RegExp over matched region  
                                                              - pos = positional conservation: each position treated independently   
                                                              - prop = conservation of amino acid properties  
                                                              - all = all three methods for comparison purposes  
                                                              - **consamb=T/F :** Whether to calculate conservation allowing for degeneracy of motif (True) or of fixed variant (False) [True]  
                                                                - **consinfo=T/F :** Weight positions by information content (does nothing for conscore=abs) [True]  
                                                                  - **consweight=X :** Weight given to global percentage identity for conservation, given more weight to closer sequences [0]  
                                                                    - 0 gives equal weighting to all. Negative values will upweight distant sequences.  
                                                                    - **posmatrix=FILE :** Score matrix for amino acid combinations used in pos weighting. (conscore=pos builds from propmatrix) [None]  
                                                                      - **aaprop=FILE :** Amino Acid property matrix file. [aaprop.txt]  
                                                                          
                                                                        ## Alignment Settings ##  
                                                                        - **protalndir=PATH :** Output path for Protein Alignments [ProteinAln/]  
                                                                          - **motalndir=PATH :** Output path for Motif Alignments []  
                                                                            - **flanksize=X :** Size of sequence flanks for motifs [30]  
                                                                              - **xdivide=X :** Size of dividing Xs between motifs [10]  
                                                                                  
                                                                                ## System Settings ##  
                                                                                - **iupath=PATH :** The full path to the IUPred exectuable [c:/bioware/iupred/iupred.exe]  
                                                                                  ??- **memsaver=T/F :** Whether to store all results in Objects (False) or clear as search proceeds (True) [True] ??  
                                                                                    ?- should this be controlled purely by the calling program? Probably!  
                                                                                    - **fullforce=T/F :** Whether to force regeneration of alignments using GOPHER  
                                                                                        
                                                                                      **Uses general modules:** copy, glob, os, string, sys, time  
                                                                                      **Uses RJE modules:** rje, rje\_aaprop, rje\_disorder, rje\_motif\_V3, rje\_motif\_cons, rje\_scoring, rje\_seq, rje\_sequence,  
                                                                                      rje\_blast, rje\_uniprot  
                                                                                      **Other modules needed:** rje\_dismatrix,

---


## \*\*\* Module rje\_motifocc \*\*\* [Top]

**Module:** rje\_motifocc  
**Description:** Motif Occurrence Module  
**Version:** 0.0  
**Last Edit:** 29/01/07  
**Copyright (C) 2007 Richard J. Edwards - See source code for GNU License Notice**  
  
**Function:**  
This module contains the MotifOcc class. This class if for storing methods and attributes pertinent to an individual  
occurrence of a motif, i.e. one Motif instance in one sequence at one position. This class is loosely based on (and  
should replace) the old PRESTO PrestoHit object. (And, to some extent, the PrestoSeqHit object.) This class is  
designed to be flexible for use with PRESTO, SLiMPickings and CompariMotif, among others.  
  
In addition to storing the standard info and stat dictionaries, this object will store a "Data" dictionary, which  
contains the (program-specific) data to be output for a given motif. All data will be in string format. The  
getData() and getStat() methods will automatically convert from string to numerics as needed.  
  
**Commandline:**  
This module has no standalone functionality and should not be called from the commandline.  
  
**Uses general modules:** copy, glob, os, string, sys, time  
**Uses RJE modules:** rje, rje\_seq, rje\_sequence  
**Other modules needed:** None

---


## \*\*\* Module rje\_pam \*\*\* [Top]

**Module:** rje\_pam  
**Description:** Contains Objects for PAM matrices  
**Version:** 1.2  
**Last Edit:** 29/08/06  
**Copyright (C) 2005 Richard J. Edwards - See source code for GNU License Notice**  
  
**Function:**  
This module handles functions associated with PAM matrices. A PAM1 matrix is read from the given input file and  
multiplied by itself to give PAM matrices corresponding to greater evolutionary distance. (PAM1 equates to one amino acid  
substitition per 100aa of sequence.)   
  
**Commandline:**  
- **pamfile=X :** Sets PAM1 input file [jones.pam]  
  - **pammax=X :** Initial maximum PAM matrix to generate [100]  
    - **pamcut=X :** Absolute maximum PAM matrix [1000]  
        
      **Alternative PAM matrix commands:**  
      - **altpam=FILE :** Alternative to PAM file input = matrix needing scaling by aafreq [None]  
        - **seqin=FILE :** Sequence file from which to calculate AA freq for scaling [None]  
          - **pamout=FILE :** Name for rescaled PAM matrix output [\*.pam named after altpam=FILE]  
              
            **Classes:**  
            PamCtrl(rje.RJE\_Object):  
            - Controls a set of PAM matrices.  
            PAM(pam,rawpamp,alpha):  
            - Individual PAM matrix.  
            >> pam:int = PAM distance  
            >> rawpamp:dictionary of substitution probabilities  
            >> alpha:list of amino acids (alphabet)  
              
            **Uses general modules:** os, string, sys, time  
            **Uses RJE modules:** rje

---


## \*\*\* Module rje\_scoring \*\*\* [Top]

**Module:** rje\_scoring  
**Description:** Scoring and Ranking Methods for RJE Python Modules  
**Version:** 0.0  
**Last Edit:** 22/01/07  
**Copyright (C) 2007 Richard J. Edwards - See source code for GNU License Notice**  
  
**Function:**  
This module contains methods only for ranking, filtering and generating new scores from python dictionaries. At its  
conception, this is for unifying and clarifying the new scoring and filtering options used by PRESTO & SLiMPickings,  
though it is conceived that the methods will also be suitable for use in other/future programs.  
  
The general format of expected data is a list of column headers, on which data may be filtered/ranked etc. or  
combined to make new scores, and a dictionary containing the data for a given entry. The keys for the dictionary  
should match the headers in a \*case-insensitive\* fashion. (The keys and headers will not be changed but will match  
without using case, so do not have two case-sensitive variables, such as "A" and "a" unless they have the same  
values.) !NB! For some methods, the case should have been matched.  
  
Methods in this module will either return the input dictionary or list with additional elements (if calculating new  
scores) or take a list of data dictionaries and return a ranked or filtered list.  
  
Methods in this module:  
\* setupStatFilter(callobj,statlist,filterlist) = Makes StatFilter dictionary from statlist and filterlist  
\* statFilter(callobj,data,statfilter) = Filters data dictionary according to statfilter dictionary.  
\* setupCustomScores(callobj,statlist,scorelist,scoredict) = Checks and returns Custom Scores and related lists  
  
**Commandline:**  
This module is not for standalone running and has no commandline options (including 'help'). All options are handled  
by the parent modules.  
  
**Uses general modules:** copy, os, string, sys  
**Uses RJE modules:** rje  
**Other modules needed:** None

---


## \*\*\* Module rje\_seq \*\*\* [Top]

**Module:** rje\_seq  
**Description:** DNA/Protein sequence module  
**Version:** 3.6  
**Last Edit:** 10/06/09  
**Copyright (C) 2005 Richard J. Edwards - See source code for GNU License Notice**  
  
**Function:**  
Contains Classes and methods for sets of DNA and protein sequences.   
  
**Sequence Input/Output Commands:**   
- **seqin=FILE :** Loads sequences from FILE (fasta,phylip,aln,uniprot or fastacmd names from fasdb) [None]  
  - **query=X :** Selects query sequence by name [None]  
    - **acclist=LIST :** Extract only AccNums in list. LIST can be FILE or list of AccNums X,Y,.. [None]  
      - **fasdb=FILE :** Fasta format database to extract sequences from [None]  
        - **mapseq=FILE :** Maps sequences from FILE to sequences of same name [None]  
          - **seqout=FILE :** Saves 'tidied' sequences to FILE after loading and manipulations [None]  
            - **reformat=X :** Outputs sequence in a particular format, where X is:  
              - fasta/fas/phylip/scanseq/acclist/acc/idlist/fastacmd/teiresias/mysql/nexus/3rf/6rf/est6rf [None]  
              - if no seqout=FILE given, will use input file name as base and add appropriate exension.  
              #!# reformat=X may not be fully implemented. Report erroneous behaviour! #!#  
              - **logrem=T/F :** Whether to log removed sequences [True] - suggest False with filtering of large files!  
                  
                **Sequence Loading/Formatting Options:**  
                - **alphabet=LIST :** Alphabet allowed in sequences [standard 1 letter AA codes]  
                  - **replacechar=T/F :** Whether to remove numbers and replace characters not found in the given alphabet with 'X' [True]  
                    - **autofilter=T/F :** Whether to automatically apply sequence filters etc. upon loading sequence [True]  
                      - **autoload=T/F :** Whether to automatically load sequences upon initiating object [True]  
                        - **memsaver=T/F :** Minimise memory usage. Input sequences must be fasta. [False]  
                          - **degap=T/F :** Degaps each sequence [False]  
                            - **tidygap=T/F :** Removes any columns from alignments that are 100% gap [True]  
                              - **gnspacc=T/F :** Convert sequences into gene\_SPECIES\_\_AccNum format wherever possible. [False]   
                                - **seqtype=X :** Force program to read as DNA, RNA or Protein (case insensitive) [Protein]  
                                  - **dna=T/F :** Alternative identification of sequences as DNA [False]  
                                    - **align=T/F :** Whether the sequences should be aligned. Will align if unaligned. [False]  
                                      - **rna2dna=T/F :** Converts RNA to DNA [False]  
                                        - **trunc=X :** Truncates each sequence to the first X aa. (Last X aa if -ve) (Useful for webservers like SingalP.) [0]  
                                          - **usecase=T/F :** Whether to output sequences in mixed case rather than converting all to upper case [False]  
                                            - **case=LIST :** List of positions to switch case, starting with first lower case (e.g case=20,-20 will have ends UC) []  
                                                
                                              **Sequence Filtering Commands:**  
                                              - **filterout=FILE :** Saves filtered sequences (as fasta) into FILE. \*NOTE: File is appended if append=T\* [None]  
                                                - **minlen=X :** Minimum length of sequences [0]  
                                                  - **maxlen=X :** Maximum length of sequences (<=0 = No maximum) [0]  
                                                    - **maxgap=X :** Maximum proportion of sequence that may be gaps (<=0 = No maximum) [0]  
                                                      - **maxx=X :** Maximum proportion of sequence that may be Xs (<=0 = No maximum; >=1 = Absolute no.) [0]  
                                                        - **minorf=X :** Minimum ORF length for a DNA/EST translation (reformatting only) [0]  
                                                          - **minpoly=X :** Minimum length of poly-A tail for 3rf / 6rf EST translation (reformatting only) [20]  
                                                            - **gapfilter=T/F :** Whether to filter gappy sequences upon loading [True]  
                                                              - **dblist=LIST :** List of databases in order of preference (good to bad)  
                                                                [sprot,ipi,uniprot,trembl,ens\_known,ens\_novel,ens\_scan]  
                                                                - **dbonly=T/F :** Whether to only allow sequences from listed databases [False]  
                                                                  - **unkspec=T/F :** Whether sequences of unknown species are allowed [True]  
                                                                    - **accnr=T/F :** Check for redundant Accession Numbers/Names on loading sequences. [True]  
                                                                      - **seqnr=T/F :** Make sequences Non-Redundant [False]  
                                                                        - **nrid=X :** %Identity cut-off for Non-Redundancy (GABLAMO) [100.0]  
                                                                          - **nrsim=X :** %Similarity cut-off for Non-Redundancy (GABLAMO) [None]   
                                                                            - **nralign=T/F :** Use ALIGN for non-redundancy calculations rather than GABLAMO [False]  
                                                                              - **specnr=T/F :** Non-Redundancy within same species only [False]  
                                                                                - **querynr=T/F :** Perform Non-Redundancy on Query species (True) or limit to non-Query species (False) [True]  
                                                                                  - **goodX=LIST :** Filters where only sequences meeting the requirement of LIST are kept.  
                                                                                    LIST may be a list X,Y,..,Z or a FILE which contains a list [None]  
                                                                                    - goodacc = list of accession numbers  
                                                                                    - goodseq = list of sequence names  
                                                                                    - goodspec = list of species codes  
                                                                                    - gooddb = list of source databases  
                                                                                    - gooddesc = list of terms that, at least one of which must be in description line  
                                                                                    - **badX=LIST :** As goodX but excludes rather than retains filtered sequences  
                                                                                        
                                                                                      **System Info Commands:** \* Use forward slashes for paths (/)  
                                                                                      - **blastpath=PATH :** Path to BLAST programs ['']   
                                                                                        - **fastapath=PATH :** Path to FASTA programs ['']   
                                                                                          - **clustalw=PATH :** Path to CLUSTALW program ['clustalw']  
                                                                                            - **mafft=PATH :** Path to MAFFT alignment program ['mafft']  
                                                                                              - **muscle=PATH :** Path to MUSCLE ['muscle']   
                                                                                                - **fas=PATH :** Path to FSA alignment program ['fsa']   
                                                                                                  - **win32=T/F :** Run in Win32 Mode [False]  
                                                                                                    - **alnprog=X :** Choice of alignment program to use (clustalw/muscle/mafft/fsa) [muscle]  
                                                                                                        
                                                                                                      **Sequence Manipulation/Function Commands:**  
                                                                                                      pamdis : Makes an all by all PAM distance matrix  
                                                                                                      - **split=X :** Splits file into numbered files of X sequences. (Useful for webservers like TMHMM.)  
                                                                                                        - **relcons=FILE:** Returns a file containing Pos AbsCons RelCons [None]  
                                                                                                          - **relconwin=X :** Window size for relative conservation scoring [30]  
                                                                                                            - **makepng=T/F :** Whether to make RelCons PNG files [False]  
                                                                                                              - **seqname=X :** Output sequence names for PNG files etc. (short/Name/Number/AccNum/ID) [short]  
                                                                                                                  
                                                                                                                **DisMatrix Options:**  
                                                                                                                - **outmatrix=X :** Type for output matrix - text / mysql / phylip  
                                                                                                                    
                                                                                                                  **Special:**  
                                                                                                                  - **blast2fas=FILE1,FILE2,...,FILEn :** Will blast sequences against list of databases and compile a fasta file of results per query  
                                                                                                                    - use options from rje\_blast.py for each individual blast (blastd=FILE will be over-ridden)  
                                                                                                                    - saves results in AccNum.blast.fas and will append existing files!  
                                                                                                                    - **haqbat=FILE :** Generate a batch file (FILE) to run HAQESAC on generated BLAST files, with seqin as queries [None]  
                                                                                                                        
                                                                                                                      **Classes:**  
                                                                                                                      SeqList(rje.RJE\_Object):   
                                                                                                                      - Sequence List Class. Holds a list of Sequence Objects and has methods for manipulation etc.  
                                                                                                                      Sequence(rje\_sequence.Sequence):   
                                                                                                                      - Individual Sequence Class.   
                                                                                                                      DisMatrix(rje\_dismatrix.DisMatrix):   
                                                                                                                      - Sequence Distance Matrix Class.  
                                                                                                                        
                                                                                                                      **Uses general modules:** copy, math, os, random, re, shutil, sre\_constants, string, sys, time  
                                                                                                                      **Uses RJE modules:** rje, rje\_blast, rje\_dismatrix, rje\_pam, rje\_sequence, rje\_uniprot

---


## \*\*\* Module rje\_sequence \*\*\* [Top]

**Module:** rje\_sequence  
**Description:** DNA/Protein sequence object  
**Version:** 1.12  
**Last Edit:** 29/05/09  
**Copyright (C) 2006 Richard J. Edwards - See source code for GNU License Notice**  
  
**Function:**  
This module contains the Sequence Object used to store sequence data for all PEAT applications that used DNA or  
protein sequences. It has no standalone functionality.  
  
This modules contains all the methods for parsing out sequence information, including species and source database,  
based on the format of the input sequences. If using a consistent but custom format for fasta description lines,  
please contact me and I can add it to the list of formats currently recognised.  
  
**Uses general modules:** copy, os, random, re, sre\_constants, string, sys, time  
**Uses RJE modules:** rje, rje\_disorder

---


## \*\*\* Module rje\_slim \*\*\* [Top]

**Module:** RJE\_SLiM  
**Description:** Short Linear Motif class module  
**Version:** 1.1  
**Last Edit:** 03/08/07  
**Copyright (C) 2007 Richard J. Edwards - See source code for GNU License Notice**  
  
**Function:**  
This module contains the new SLiM class, which replaces the old Motif class, for use with both SLiMFinder and  
SLiMSearch. In addition, this module encodes some general motif methods. Note that the new methods are not  
designed with Mass Spec data in mind and so some of the more complicated regexp designations for unknown amino acid  
order etc. have been dropped. Because the SLiM class explicitly deals with \*short\* linear motifs, wildcard gaps are  
capped at a max length of 9.  
  
The basic SLiM class stores its pattern in several forms:  
- info['Sequence'] stores the original pattern given to the Motif object  
- info['Slim'] stores the pattern as a SLiMFinder-style string of defined elements and wildcard spacers  
- dict['MM'] stores lists of Slim strings for each number of mismatches with flexible lengths enumerated. This is  
used for actual searches in SLiMSearch.  
- dict['Search'] stores the actual regular expression variants used for searching, which has a separate entry for  
each length variant - otherwise Python RegExp gets confused! Keys for this dictionary relate to the number of  
mismatches allowed in each variant and match dict['MM'].  
  
The following were previously used by the Motif class and may be revived for the new SLiM class if needed:   
- list['Variants'] stores simple strings of all the basic variants - length and ambiguity - for indentifying the  
"best" variant for any given match  
  
The SLiM class is designed for use with the SLiMList class. When a SLiM is added to a SLiMList object, the  
SLiM.format() command is called, which generates the 'Slim' string. After this - assuming it is to be kept -  
SLiM.makeVariants() makes the 'Variants' list. If creating a motif object in another module, these method should be  
called before any sequence searching is performed. If mismatches are being used, the SLiM.misMatches() method must  
also be called.  
  
SLiM occurrences are stored in the dict['Occ'] attribute. The keys for this are Sequence objects and values are  
either a simple list of positions (1 to L) or a dictionary of attributes with positions as keys.  
  
**Commandline:**  
These options should be listed in the docstring of the module using the motif class:  
-- **alphabet=LIST :** List of letters in alphabet of interest [AAs]  
  -- **ambcut=X :** Cut-off for max number of choices in ambiguous position to be shown as variant (0=All) [10]  
    -- **trimx=T/F :** Trims Xs from the ends of a motif [False]  
      -- **dna=T/F :** Whether motifs should be considered as DNA motifs [False]  
          
        **Uses general modules:** copy, glob, os, string, sys, time  
        **Uses RJE modules:** rje  
        **Other modules needed:** None

---


## \*\*\* Module rje\_slimcalc \*\*\* [Top]

**Module:** rje\_slimcalc  
**Description:** SLiM Attribute Calculation Module  
**Version:** 0.3  
**Last Edit:** 13/08/08  
**Copyright (C) 2007 Richard J. Edwards - See source code for GNU License Notice**  
  
**Function:**  
This module is based on the old rje\_motifstats module. It is primarily for calculating empirical attributes of SLiMs  
and their occurrences, such as Conservation, Hydropathy and Disorder.   
  
**Commandline:**  
#~~~~~~~~~~~~~~~~~~~~~~~~~~~~~~~~~~~~~~~~~~~~~~~~~~~~~~~~~~~~~~~~~~~~~~~~~~~~~~~~~~~~~~~~~~~~~~~~~~~~~~~~~~~~~~~~~~~#  
### Motif Occurrence Attribute Options ###  
- **slimcalc=LIST :** List of additional attributes to calculate for occurrences - Cons,SA,Hyd,Fold,IUP,Chg,Comp []  
  - **winsize=X :** Used to define flanking regions for calculations. If negative, will use flanks \*only\* [0]  
    - **relconwin=X :** Window size for relative conservation scoring [30]  
      - **iupath=PATH :** The full path to the IUPred exectuable [c:/bioware/iupred/iupred.exe]  
        - **iucut=X :** Cut-off for IUPred results (0.0 will report mean IUPred score) [0.0]  
          - **iumethod=X :** IUPred method to use (long/short) [short]  
            - **percentile=X :** Percentile steps to return in addition to mean [0]  
              #~~~~~~~~~~~~~~~~~~~~~~~~~~~~~~~~~~~~~~~~~~~~~~~~~~~~~~~~~~~~~~~~~~~~~~~~~~~~~~~~~~~~~~~~~~~~~~~~~~~~~~~~~~~~~~~~~~~#  
              ### Alignment Settings ###  
              - **usealn=T/F :** Whether to search for and use alignments where present. [False]  
                - **alndir=PATH :** Path to pre-made alignment files [./]  
                  - **alnext=X :** File extension of alignment files, AccNum.X (checked before Gopher used) [aln.fas]  
                    - **usegopher=T/F :** Use GOPHER to generate missing orthologue alignments in alndir - see gopher\_V2.py options [False]  
                      - **gopherdir=PATH :** Path from which to call Gopher (and look for PATH/ALN/AccNum.orthaln.fas) [./]   
                        - **fullforce=T/F :** Whether to force regeneration of alignments using GOPHER [False]  
                          - **orthdb=FILE :** File to use as source of orthologues for GOPHER []  
                            #~~~~~~~~~~~~~~~~~~~~~~~~~~~~~~~~~~~~~~~~~~~~~~~~~~~~~~~~~~~~~~~~~~~~~~~~~~~~~~~~~~~~~~~~~~~~~~~~~~~~~~~~~~~~~~~~~~~#  
                            ### Conservation Parameters ###   
                            - **conspec=LIST :** List of species codes for conservation analysis. Can be name of file containing list. [None]  
                              - **conscore=X :** Type of conservation score used: [pos]  
                                - abs = absolute conservation of motif using RegExp over matched region  
                                - pos = positional conservation: each position treated independently  
                                - prob = conservation based on probability from background distribution  
                                - prop = conservation of amino acid properties  
                                - all = all three methods for comparison purposes  
                                - **consamb=T/F :** Whether to calculate conservation allowing for degeneracy of motif (True) or of fixed variant (False) [True]  
                                  - **consinfo=T/F :** Weight positions by information content (does nothing for conscore=abs) [True]  
                                    - **consweight=X :** Weight given to global percentage identity for conservation, given more weight to closer sequences [0]  
                                      - 0 gives equal weighting to all. Negative values will upweight distant sequences.  
                                      - **minhom=X :** Minimum number of homologues for making conservation score [1]  
                                        - **homfilter=T/F :** Whether to filter homologues using seqfilter options [False]  
                                          - **alngap=T/F :** Whether to count proteins in alignments that have 100% gaps over motif (True) or (False) ignore  
                                            as putative sequence fragments [False] (NB. All X regions are ignored as sequence errors.)  
                                            - **posmatrix=FILE :** Score matrix for amino acid combinations used in pos weighting. (conscore=pos builds from propmatrix) [None]  
                                              - **aaprop=FILE :** Amino Acid property matrix file. [aaprop.txt]  
                                                - **masking=T/F :** Whether to use seq.info['MaskSeq'] for Prob cons, if present (else 'Sequence') [True]  
                                                  - **vnematrix=FILE :** BLOSUM matrix file to use for VNE relative conservation []  
                                                    - **relgappen=T/F :** Whether to invoke the "Gap Penalty" during relative conservation calculations [True]  
                                                      #~~~~~~~~~~~~~~~~~~~~~~~~~~~~~~~~~~~~~~~~~~~~~~~~~~~~~~~~~~~~~~~~~~~~~~~~~~~~~~~~~~~~~~~~~~~~~~~~~~~~~~~~~~~~~~~~~~~#  
                                                      ### SLiM/Occ Filtering Options ###  
                                                      - **slimfilter=LIST :** List of stats to filter (remove matching) SLiMs on, consisting of X\*Y []  
                                                        - X is an output stat (the column header),  
                                                        - \* is an operator in the list >, >=, !=, =, >= ,<   
                                                        - Y is a value that X must have, assessed using \*.  
                                                        This filtering is crude and may behave strangely if X is not a numerical stat!  
                                                        !!! Remember to enclose in "quotes" for <> filtering !!!  
                                                        - **occfilter=LIST :** Same as slimfilter but for individual occurrences []  
                                                            
                                                          **Uses general modules:** copy, glob, os, string, sys, time  
                                                          **Uses RJE modules:** rje  
                                                          **Other modules needed:** None

---


## \*\*\* Module rje\_slimcore \*\*\* [Top]

**Module:** rje\_slimcore  
**Description:** Core module/object for SLiMFinder and SLiMSearch  
**Version:** 1.4  
**Last Edit:** 12/06/09  
**Copyright (C) 2007 Richard J. Edwards - See source code for GNU License Notice**  
  
**Function:**  
This module is primarily to contain core dataset processing methods for both SLiMFinder and SLiMSearch to inherit and  
use. This primarily consists of the options and methods for masking datasets and generating UPC. This module can  
therefore be run in standalone mode to generate UPC files for SLiMFinder or SLiMSearch.  
  
In addition, the secondary MotifSeq and Randomise functions are handled here.  
  
**Secondary Functions:**  
The "MotifSeq" option will output fasta files for a list of X:Y, where X is a motif pattern and Y is the output file.  
  
The "Randomise" function will take a set of input datasets (as in Batch Mode) and regenerate a set of new datasets  
by shuffling the UPC among datasets. Note that, at this stage, this is quite crude and may result in the final  
datasets having fewer UPC due to common sequences and/or relationships between UPC clusters in different datasets.  
  
**Commandline:** ~~~~~~~~~~~~~~~~~~~~~~~~~~~~~~~~~~~~~~~~~~~~~~~~~~~~~~~~~~~~~~~~~~~~~~~~~~~~~~~~~~~~~~~~~~~~~~~~~~~~~~~~~~~#  
### Basic Input/Output Options ###   
- **seqin=FILE :** Sequence file to search [None]  
  - **batch=LIST :** List of files to search, wildcards allowed. (Over-ruled by seqin=FILE.) [\*.dat,\*.fas]  
    - **maxseq=X :** Maximum number of sequences to process [500]  
      - **maxupc=X :** Maximum UPC size of dataset to process [0]  
        - **walltime=X :** Time in hours before program will abort search and exit [1.0]  
          - **resdir=PATH :** Redirect individual output files to specified directory (and look for intermediates) [SLiMFinder/]  
            - **buildpath=PATH :** Alternative path to look for existing intermediate files [SLiMFinder/]  
              - **force=T/F :** Force re-running of BLAST, UPC generation and SLiMBuild [False]  
                - **dna=T/F :** Whether the sequences files are DNA rather than protein [False]  
                  #~~~~~~~~~~~~~~~~~~~~~~~~~~~~~~~~~~~~~~~~~~~~~~~~~~~~~~~~~~~~~~~~~~~~~~~~~~~~~~~~~~~~~~~~~~~~~~~~~~~~~~~~~~~~~~~~~~~#  
                  ### Evolutionary Filtering Options ###  
                  - **efilter=T/F :** Whether to use evolutionary filter [True]  
                    - **blastf=T/F :** Use BLAST Complexity filter when determining relationships [True]  
                      - **blaste=X :** BLAST e-value threshold for determining relationships [1e=4]  
                        - **altdis=FILE :** Alternative all by all distance matrix for relationships [None]  
                          - **gablamdis=FILE :** Alternative GABLAM results file [None] (!!!Experimental feature!!!)  
                            - **domtable=FILE :** Domain table containing domain ("Type") and sequence ("Name") pairings for additional UPC [None]  
                              - **homcut=X :** Max number of homologues to allow (to reduce large multi-domain families) [0]  
                                - **extras=T/F :** Whether to generate additional output files (distance matrices etc.) [True]  
                                  #~~~~~~~~~~~~~~~~~~~~~~~~~~~~~~~~~~~~~~~~~~~~~~~~~~~~~~~~~~~~~~~~~~~~~~~~~~~~~~~~~~~~~~~~~~~~~~~~~~~~~~~~~~~~~~~~~~~#  
                                  ### Input Masking and AA Frequency Options ###  
                                  - **masking=T/F :** Master control switch to turn off all masking if False [True]  
                                    - **dismask=T/F :** Whether to mask ordered regions (see rje\_disorder for options) [False]  
                                      - **consmask=T/F :** Whether to use relative conservation masking [False]  
                                        - **ftmask=LIST :** UniProt features to mask out [EM,DOMAIN,TRANSMEM]  
                                          - **imask=LIST :** UniProt features to inversely ("inclusively") mask. (Seqs MUST have 1+ features) []  
                                            - **fakemask=T/F :** Whether to invoke UniFake to generate additional features for masking [False]  
                                              - **compmask=X,Y :** Mask low complexity regions (same AA in X+ of Y consecutive aas) [5,8]  
                                                - **casemask=X :** Mask Upper or Lower case [None]  
                                                  - **metmask=T/F :** Masks the N-terminal M (can be useful if SLiMFinder termini=T) \*Also maskm=T/F\* [True]  
                                                    - **posmask=LIST :** Masks list of position-specific aas, where list = pos1:aas,pos2:aas \*Also maskpos=LIST\* [2:A]  
                                                      - **aamask=LIST :** Masks list of AAs from all sequences (reduces alphabet) []  
                                                        - **motifmask=X :** List (or file) of motifs to mask from input sequences []  
                                                          - **logmask=T/F :** Whether to output the log messages for masking of individual sequences to screen [False]  
                                                            - **maskfreq=T/F :** Whether to use masked AA Frequencies (True), or (False) mask after frequency calculations [True]  
                                                              - **aafreq=FILE :** Use FILE to replace individual sequence AAFreqs (FILE can be sequences or aafreq) [None]   
                                                                - **smearfreq=T/F :** Whether to "smear" AA frequencies across UPC rather than keep separate AAFreqs [False]  
                                                                  #~~~~~~~~~~~~~~~~~~~~~~~~~~~~~~~~~~~~~~~~~~~~~~~~~~~~~~~~~~~~~~~~~~~~~~~~~~~~~~~~~~~~~~~~~~~~~~~~~~~~~~~~~~~~~~~~~~~#  
                                                                  ### Advanced Output Options ###  
                                                                  - **targz=T/F :** Whether to tar and zip dataset result files (UNIX only) [False]  
                                                                    - **pickle=T/F :** Whether to save/use pickles [True]  
                                                                      - **savespace=0 :** Delete "unneccessary" files following run (best used with targz): [0]  
                                                                        - 0 = Delete no files  
                                                                        - 1 = Delete all bar \*.upc, \*.pickle files (Pickle excluded from tar.gz with this setting)  
                                                                        - 2 = Delete all bar \*.upc, \*.pickle and \*.occ.csv files (Pickle excluded from tar.gz with this setting)  
                                                                        - 3 = Delete all dataset-specific files including \*.upc and \*.pickle (not \*.tar.gz)  
                                                                        #~~~~~~~~~~~~~~~~~~~~~~~~~~~~~~~~~~~~~~~~~~~~~~~~~~~~~~~~~~~~~~~~~~~~~~~~~~~~~~~~~~~~~~~~~~~~~~~~~~~~~~~~~~~~~~~~~~~#  
                                                                        ### Additional Functions I: MotifSeq ###   
                                                                        - **motifseq=LIST :** Outputs fasta files for a list of X:Y, where X is the pattern and Y is the output file []  
                                                                          - **slimbuild=T/F :** Whether to build motifs with SLiMBuild. (For combination with motifseq only.) [True]  
                                                                              
                                                                            ### Additional Functions II: Randomised datasets ###  
                                                                            - **randomise=T/F :** Randomise UPC within batch files and output new datasets [False]  
                                                                              - **randir=PATH :** Output path for creation of randomised datasets [Random/]  
                                                                                - **randbase=X :** Base for random dataset name [rand]  
                                                                                  - **randsource=FILE :** Source for new sequences for random datasets (replaces UPCs) [None]  
                                                                                    #~~~~~~~~~~~~~~~~~~~~~~~~~~~~~~~~~~~~~~~~~~~~~~~~~~~~~~~~~~~~~~~~~~~~~~~~~~~~~~~~~~~~~~~~~~~~~~~~~~~~~~~~~~~~~~~~~~~#  
                                                                                      
                                                                                    **Uses general modules:** copy, glob, math, os, string, sys, time  
                                                                                    **Uses RJE modules:** rje, rje\_blast, rje\_dismatrix\_V2, rje\_seq, rje\_scoring, rje\_slim, rje\_slimlist  
                                                                                    **Other modules needed:** None

---


## \*\*\* Module rje\_slimlist \*\*\* [Top]

**Module:** rje\_slimlist  
**Description:** SLiM dataset manager  
**Version:** 0.3  
**Last Edit:** 09/05/08  
**Copyright (C) 2007 Richard J. Edwards - See source code for GNU License Notice**  
  
**Function:**  
This module is a replace for the rje\_motiflist module and contains the SLiMList class, a replacement for the  
MotifList class. The primary function of this class is to load and store a list of SLiMs and control generic SLiM  
outputs for such programs as SLiMSearch. This class also controls motif filtering according to features of the motifs  
and/or their occurrences.  
  
**Commandline:** ~~~~~~~~~~~~~~~~~~~~~~~~~~~~~~~~~~~~~~~~~~~~~~~~~~~~~~~~~~~~~~~~~~~~~~~~~~~~~~~~~~~~~~~~~~~~~~~~~~~~~~~~~~~#  
### Basic Input/Output Options ###  
- **motifs=FILE :** File of input motifs/peptides [None]  
  Single line per motif format = 'Name Sequence #Comments' (Comments are optional and ignored)  
  Alternative formats include fasta, SLiMDisc output and raw motif lists.  
  - **reverse=T/F :** Reverse the motifs - good for generating a test comparison data set [False]  
    - **wildscram=T/F :** Perform a wildcard spacer scrambling - good for generating a test comparison data set [False]  
      - **motifout=FILE :** Name of output file for reformatted/filtered SLiMs (PRESTO format) [None]  
        - **ftout=T/F :** Make a file of UniProt features for extracted parent proteins, where possible, incoroprating SLIMs [\*.features.tdt]  
          - **mismatch=LIST :** List of X:Y pairs for mismatch dictionary, where X mismatches allowed for Y+ defined positions []   
            - **motinfo=FILE :** Filename for output of motif summary table (if desired) [None]  
              - **dna=T/F :** Whether motifs should be considered as DNA motifs [False]  
                #~~~~~~~~~~~~~~~~~~~~~~~~~~~~~~~~~~~~~~~~~~~~~~~~~~~~~~~~~~~~~~~~~~~~~~~~~~~~~~~~~~~~~~~~~~~~~~~~~~~~~~~~~~~~~~~~~~~#  
                ### Advanced Input I: Motif Filtering Options ###  
                - **minpos=X :** Min number of defined positions [0]  
                  - **minfix=X :** Min number of fixed positions for a motif to contain [0]  
                    - **minic=X :** Min information content for a motif (1 fixed position = 1.0) [0.0]  
                      - **goodmotif=LIST :** List of text to match in Motif names to keep (can have wildcards) []  
                        - **nrmotif=T/F :** Whether to remove redundancy in input motifs [False]  
                            
                          ### Advanced Input II: Motif reformatting options ###  
                          - **trimx=T/F :** Trims Xs from the ends of a motif [False]  
                            - **minimotif=T/F :** Input file is in minimotif format and will be reformatted (PRESTO File format only) [False]  
                              - **ambcut=X :** Cut-off for max number of choices in ambiguous position to be shown as variant [10]  
                                #~~~~~~~~~~~~~~~~~~~~~~~~~~~~~~~~~~~~~~~~~~~~~~~~~~~~~~~~~~~~~~~~~~~~~~~~~~~~~~~~~~~~~~~~~~~~~~~~~~~~~~~~~~~~~~~~~~~#  
                                ### Advanced Output I: Motif Occurrence Statistics Options ###  
                                - **slimcalc=LIST :** List of additional statistics to calculate for occurrences - Cons,SA,Hyd,Fold,IUP,Chg []  
                                  - **winsize=X :** Used to define flanking regions for stats. If negative, will use flanks \*only\* [0]  
                                    - **iupath=PATH :** The full path to the IUPred exectuable [c:/bioware/iupred/iupred.exe]  
                                      - **iucut=X :** Cut-off for IUPred results (0.0 will report mean IUPred score) [0.0]  
                                        - **iumethod=X :** IUPred method to use (long/short) [short]  
                                          - **percentile=X :** Percentile steps to return in addition to mean [0]  
                                            - **peptides=T/F :** Whether to output peptide sequences based on motif and winsize [False]  
                                                
                                              ### Advanced Output II: Alignment Settings ###  
                                              - **usealn=T/F :** Whether to search for and use alignemnts where present. [False]  
                                                - **gopher=T/F :** Use GOPHER to generate missing orthologue alignments in alndir - see gopher.py options [False]  
                                                  - **alndir=PATH :** Path to alignments of proteins containing motifs [./] \* Use forward slashes (/)  
                                                    - **alnext=X :** File extension of alignment files, accnum.X [aln.fas]  
                                                      - **protalndir=PATH :** Output path for Protein Alignments [ProteinAln/]  
                                                        - **motalndir=PATH :** Output path for Motif Alignments []  
                                                          - **flanksize=X :** Size of sequence flanks for motifs [30]  
                                                            - **xdivide=X :** Size of dividing Xs between motifs [10]  
                                                              - **fullforce=T/F :** Whether to force regeneration of alignments using GOPHER  
                                                                  
                                                                ### Advanced Output III: Conservation Parameters ###   
                                                                - **conspec=LIST :** List of species codes for conservation analysis. Can be name of file containing list. [None]  
                                                                  - **conscore=X :** Type of conservation score used: [pos]  
                                                                    - abs = absolute conservation of motif using RegExp over matched region  
                                                                    - pos = positional conservation: each position treated independently   
                                                                    - prop = conservation of amino acid properties  
                                                                    - all = all three methods for comparison purposes  
                                                                    - **consamb=T/F :** Whether to calculate conservation allowing for degeneracy of motif (True) or of fixed variant (False) [True]  
                                                                      - **consinfo=T/F :** Weight positions by information content (does nothing for conscore=abs) [True]  
                                                                        - **consweight=X :** Weight given to global percentage identity for conservation, given more weight to closer sequences [0]  
                                                                          - 0 gives equal weighting to all. Negative values will upweight distant sequences.  
                                                                          - **alngap=T/F :** Whether to count proteins in alignments that have 100% gaps over motif (True) or (False) ignore  
                                                                            as putative sequence fragments [False] (NB. All X regions are ignored as sequence errors.)  
                                                                            - **posmatrix=FILE :** Score matrix for amino acid combinations used in pos weighting. (conscore=pos builds from propmatrix) [None]  
                                                                              - **aaprop=FILE :** Amino Acid property matrix file. [aaprop.txt]  
                                                                                #~~~~~~~~~~~~~~~~~~~~~~~~~~~~~~~~~~~~~~~~~~~~~~~~~~~~~~~~~~~~~~~~~~~~~~~~~~~~~~~~~~~~~~~~~~~~~~~~~~~~~~~~~~~~~~~~~~~#  
                                                                                  
                                                                                **Uses general modules:** copy, glob, os, string, sys, time  
                                                                                **Uses RJE modules:** rje  
                                                                                **Other modules needed:** None

---


## \*\*\* Module rje\_tm \*\*\* [Top]

**Module:** rje\_tm   
**Description:** Tranmembrane and Signal Peptide Prediction Module  
**Version:** 1.2  
**Last Edit:** 16/08/07  
**Copyright (C) 2005 Richard J. Edwards - See source code for GNU License Notice**  
  
**Function:**  
Will read in results from tmhmm and/or signalp files as appropriate and append output to:  
- tm.tdt = TM domain counts and orientation  
- domains.tdt = Domain table  
- singalp.tdt = SingalP data (use to add signal peptide domains to domains table using mySQL  
  
**Commandline:**  
- **tmhmm=FILE :** TMHMM output file [None]  
  - **signalp=FILE:** SignalP output file [None]  
    - **mysql=T/F :** Output results in tdt files for mySQL import [True]  
        
      - **seqin=FILE :** Sequence file for which predictions have been made [None]  
        - **maskcleave=T/F :** Whether to output sequences with cleaved signal peptides masked. [False]  
          - **source=X :** Source text for mySQL file ['tmhmm']  
              
            **Uses general modules:** os, string, sys, threading, time  
            **Uses RJE modules:** rje, rje\_seq  
            **Other modules required:** rje\_blast, rje\_dismatrix, rje\_pam, rje\_sequence, rje\_uniprot

---


## \*\*\* Module rje\_tree \*\*\* [Top]

**Module:** rje\_tree  
**Description:** Phylogenetic Tree Module  
**Version:** 2.5  
**Last Edit:** 19/08/09  
**Copyright (C) 2007 Richard J. Edwards - See source code for GNU License Notice**  
  
**Function:**  
Reads in, edits and outputs phylogenetic trees. Executes duplication and subfamily determination. More details available  
in documentation for HAQESAC, GASP and BADASP at http://www.bioinformatics.rcsi.ie/~redwards/  
  
**General Commands:**  
- **nsfin=FILE :** load NSF tree from FILE  
  - **phbin=FILE :** load ClustalW Format \*.phb NSF tree from FILE  
    - **seqin=FILE :** load sequence list from FILE (not compatible with useanc)  
      - **disin=FILE :** load distance matrix from FILE (Phylip format for use with distance matrix methods) [None]  
        - **useanc=FILE :** load sequences from ancestral sequence FILE (not compatible with seqin)  
          - **deflen=X :** Default length for branches when no lengths given [0.1] (or 0.1 x longest branch)  
            \*Note that in the case of conflicts (e.g. seqin=FILE1 useanc=FILE2), the latter will be used.\*  
            - **autoload=T/F :** Whether to automatically load sequences upon initiating object [True]  
                
              **Rooting Commands:**  
              - **root=X :** Rooting of tree (rje\_tree.py):  
                - mid = midpoint root tree.  
                - ran = random branch.  
                - ranwt = random branch, weighted by branch lengths.  
                - man = always ask for rooting options (unless i<0).  
                - FILE = with seqs in FILE as outgroup. (Any option other than above)  
                - **rootbuffer=X :** Min. distance from node for root placement (percentage of branch length)[0.1]  
                    
                  **Grouping/Subfamily Commands:**  
                  - **bootcut=X :** cut-off percentage of tree bootstraps for grouping.  
                    - **mfs=X :** minimum family size [3]  
                      - **fam=X :** minimum number of families (If 0, no subfam grouping) [0]  
                        - **orphan=T/F :** Whether orphans sequences (not in subfam) allowed. [True]  
                          - **allowvar=T/F:** Allow variants of same species within a group. [False]  
                            - **qryvar=T/F :** Allow variants of query species within a group (over-rides allowvar=F). [False]  
                              - **groupspec=X :** Species for duplication grouping [None]  
                                - **specdup=X :** Minimum number of different species in clade to be identified as a duplication [1]  
                                  - **group=X :** Grouping of tree  
                                    - man = manual grouping (unless i<0).  
                                    - dup = duplication (all species unless groupspec specified).  
                                    - qry = duplication with species of Query sequence (or Sequence 1) of treeseq  
                                    - one = all sequences in one group  
                                    - None = no group (case sensitive)  
                                    - FILE = load groups from file  
                                      
                                    **Tree Making Commands:**  
                                    - **cwtree=FILE :** Make a ClustalW NJ Tree from FILE (will save \*.ph or \*.phb) [None]  
                                      - **kimura=T/F :** Whether to use Kimura correction for multiple hits [True]  
                                        - **bootstraps=X :** Number of bootstraps [0]  
                                          - **clustalw=CMD :** Path to CLUSTALW (and including) program ['c:/bioware/clustalw.exe'] \* Use forward slashes (/)  
                                            - **fasttree=PATH :** Path to FastTree (and including) program [./FastTree]  
                                              - **phylip=PATH :** Path to PHYLIP programs ['c:/bioware/phylip3.65/exe/'] \* Use forward slashes (/)  
                                                - **phyoptions=FILE :** File containing extra Phylip tree-making options ('batch running') to use [None]  
                                                  - **protdist=FILE :** File containing extra Phylip PROTDIST options ('batch running') to use [None]  
                                                    - **maketree=X :** Program for making tree [None]  
                                                      - None = Do not make tree from sequences   
                                                      - clustalw = ClustalW NJ method  
                                                      - neighbor = PHYLIP NJ method   
                                                      - upgma = PHYLIP UPGMA (neighbor) method   
                                                      - fitch = PHYLIP Fitch method   
                                                      - kitsch = PHYLIP Kitsch (clock) method   
                                                      - protpars = PHYLIP MP method   
                                                      - proml = PHYLIP ML method  
                                                      - fasttree = Use FastTree  
                                                      - PATH = Alternatively, a path to a different tree program/script can be given. This should accept ClustalW parameters.  
                                                        
                                                      **Tree Display/Saving Commands**  
                                                      - **savetree=FILE :** Save a generated tree as FILE [seqin.maketree.nsf]  
                                                        - **savetype=X :** Format for generated tree file (nsf/text/r/png/te) [nsf]  
                                                          - **treeformats=LIST:** List of output formats for generated trees [nsf]  
                                                            - **outnames=X :** 'short'/'long' names in output file [short]  
                                                              - **truncnames=X :** Truncate names to X characters (0 for no truncation) [123]  
                                                                - **branchlen=T/F :** Whether to use branch lengths in output tree [True]  
                                                                  - **deflen=X :** Default branch length (when none given, also for tree scaling) [0.1]  
                                                                    - **textscale=X :** Default scale for text trees (no. of characters per deflen distance) [4]  
                                                                      - **seqnum=T/F :** Output sequence numbers (if making tree from sequences) [True]  
                                                                          
                                                                        **Classes:**  
                                                                        Tree(rje.RJE\_Object):   
                                                                        - Phylogenetic Tree class.  
                                                                        Node(rje.RJE\_Object):   
                                                                        - Individual nodes (internal and leaves) for Tree object.  
                                                                        Branch(rje.RJE\_Object):  
                                                                        - Individual branches for Tree object.  
                                                                          
                                                                        **Uses general modules:** copy, os, random, re, string, sys, time  
                                                                        **Uses RJE modules:** rje, rje\_ancseq, rje\_seq, rje\_tree\_group  
                                                                        **Other module needed:** rje\_blast, rje\_dismatrix, rje\_pam, rje\_sequence, rje\_uniprot

---


## \*\*\* Module rje\_tree\_group \*\*\* [Top]

**Module:** rje\_tree\_group  
**Description:** Contains all the Grouping Methods for rje\_tree.py  
**Version:** 1.2  
**Last Edit:** 19/08/09  
**Copyright (C) 2005 Richard J. Edwards - See source code for GNU License Notice**  
  
**Function:**  
This module is a stripped down template for methods only. This is for when a class has too many methods and becomes  
untidy. In this case, methods can be moved into a methods module and 'self' replaced with the relevant object. For  
this module, 'self' becomes '\_tree'.  
  
**Commandline:**  
This module is not for standalone running and has no commandline options (including 'help'). All options are handled  
by the parent module: rje\_tree.py  
  
**Uses general modules:** copy, re, os, string, sys  
**Uses RJE modules:** rje, rje\_seq  
**Other modules needed:** rje\_blast, rje\_dismatrix, rje\_pam, rje\_sequence, rje\_uniprot

---


## \*\*\* Module rje\_uniprot \*\*\* [Top]

**Module:** rje\_uniprot  
**Description:** RJE Module to Handle Uniprot Files  
**Version:** 3.5  
**Last Edit:** 20/10/08  
**Copyright (C) 2007 Richard J. Edwards - See source code for GNU License Notice**  
  
**Function:**  
This module contains methods for handling UniProt files, primarily in other rje modules but also with some  
standalone functionality. To get the most out of the module with big UniProt files (such as the downloads from EBI),  
first index the UniProt data using the rje\_dbase module.  
  
This module can be used to extract a list of UniProt entries from a larger database and/or to produce summary tables  
from UniProt flat files.  
  
In addition to method associated with the classes of this module, there are a number of methods that are called from  
the rje\_dbase module (primarily) to download and process the UniProt sequence database.  
  
**Input Options:**  
- **unipath=PATH :** Path to UniProt Datafile (will look here for DB Index file made with rje\_dbase)  
  - **dbindex=FILE :** Database index file [uniprot.index]  
    - **uniprot=FILE :** Name of UniProt file [None]  
      - **extract=LIST :** Extract IDs/AccNums in list. LIST can be FILE or list of IDs/AccNums X,Y,.. []  
        - **acclist=LIST :** As Extract.  
          - **specdat=LIST :** Make a UniProt DAT file of the listed species from the index (over-rules extract=LIST) []  
            - **splicevar=T/F :** Whether to search for AccNum allowing for splice variants (AccNum-X) [True]  
              - **tmconvert=T/F :** Whether to convert TOPO\_DOM features, using first description word as Type [False]  
                  
                **Output Options:**  
                - **makeindex=T/F :** Generate UniProt index files [False]  
                  - **makespec=T/F :** Generate species table [False]  
                    - **makefas=T/F :** Generate fasta files [False]  
                      - **datout=FILE :** Name of new (reduced) UniProt DAT file of extracted sequences [None]  
                        - **tabout=FILE :** Table of extracted UniProt details [None]  
                          - **linkout=FILE :** Table of extracted Database links [None]  
                            - **ftout=FILE :** Outputs table of features into FILE [None]  
                              - **domtable=T/F :** Makes a table of domains from uniprot file [False]  
                                - **cc2ft=T/F :** Extra whole-length features added for TISSUE and LOCATION (not in datout) [False]  
                                    
                                  **UniProt Conversion Options:**  
                                  - **ucft=X :** Feature to add for UpperCase portions of sequence []  
                                    - **lcft=X :** Feature to add for LowerCase portions of sequence []  
                                      - **maskft=LIST :** List of Features to mask out []  
                                        - **invmask=T/F :** Whether to invert the masking and only retain maskft features [False]  
                                          - **caseft=LIST :** List of Features to make upper case with rest of sequence lower case []  
                                              
                                            **General Options:**  
                                            - **append=T/F :** Append to results files rather than overwrite [False]  
                                              - **memsaver=T/F :** Memsaver option to save memory usage - does not retain entries in UniProt object [False]   
                                                  
                                                **Uses general modules:** glob, os, re, string, sys, time  
                                                **Uses RJE modules:** rje, rje\_sequence

---


## \*\*\* Module rje\_xgmml \*\*\* [Top]

**Module:** RJE\_XGMML  
**Description:** RJE XGMLL Module   
**Version:** 0.0  
**Last Edit:** 14/11/07  
**Copyright (C) 2007 Richard J. Edwards - See source code for GNU License Notice**  
  
**Function:**  
This module is currently designed to store data for, and then output, an XGMML file for uploading into Cytoscape etc.  
Future versions may incoporate the ability to read and manipulate existing XGMML files.  
  
**Commandline:**  
At present, all commands are handling by the class populating the XGMML object.  
  
**Uses general modules:** copy, glob, os, string, sys, time  
**Uses RJE modules:** rje  
**Other modules needed:** None

---


## \*\*\* Module rje\_zen \*\*\* [Top]

**Module:** rje\_zen  
**Description:** Random Zen Wisdom Generator  
**Version:** 1.0  
**Last Edit:** 15/04/08  
**Copyright (C) 2007 Richard J. Edwards - See source code for GNU License Notice**  
  
**Function:**  
Generates random (probably nonsensical) Zen wisdoms. Just for fun.  
  
**Commandline:**  
- **wisdoms=X :** Number of Zen Wisdoms to return [10]  
    
  **Uses general modules:** copy, glob, os, string, sys, time  
  **Uses RJE modules:** rje  
  **Other modules needed:** None

---


## \*\*\* Module unifake \*\*\* [Top]

**Module:** unifake  
**Description:** Fake UniProt DAT File Generator  
**Version:** 1.2  
**Last Edit:** 10/10/08  
**Copyright (C) 2008 Richard J. Edwards - See source code for GNU License Notice**  
  
**Function:**  
This program runs a number of in silico predication programs and converts protein sequences into a fake UniProt DAT  
flat file. Additional features may be given as one or more tables, using the features=LIST option. Please see the  
UniFake Manual for more details.   
  
**Commandline:**  
### ~ INPUT OPTIONS ~~~~~~~~~~~~~~~~~~~~~~~~~~~~~~~~~~~~~~~~~~~~~~~~~~~~~~~~~~~~~~~~~~~~~~~~~~~~~~~~~~~~~~~~~~~~~ ###  
- **seqin=FILE :** Input sequence file. See rje\_seq documentation for filtering options. [None]  
  - **spcode=X :** Species code to use if it cannot be established from sequence name [None]  
    - **features=LIST :** List of files of addtional features in delimited form []   
      - **aliases=FILE :** File of aliases to be added to Accession number list (for indexing) [None]  
        - **pfam=FILE :** PFam HMM download [None]  
          - **unipath=PATH :** Path to real UniProt Datafile (will look here for DB Index file made with rje\_dbase)  
            - **unireal=LIST :** Real UniProt data to add to UniFake output ['AC','GN','RC','RX','CC','DR','PE','KW']  
              - **fudgeft=T/F :** Fudge the real features left/right until a sequence match is found [True]  
                  
                ### ~ PROCESSING/OUTPUT OPTIONS ~~~~~~~~~~~~~~~~~~~~~~~~~~~~~~~~~~~~~~~~~~~~~~~~~~~~~~~~~~~~~~~~~~~~~~~~~~~~~~~~~ ###  
                - **unifake=LIST :** List of predictions to add to entries [tmhmm,signalp,disorder,pfam,uniprot]  
                  - **datout=FILE :** Name of output DAT file [Default input FILE.dat]  
                    - **disdom=X :** Disorder threshold below which to annotate PFam domain as "DOMAIN" [0.0]  
                      - **makeindex=T/F :** Whether to make a uniprot index file following run [False]  
                        - **ensdat=T/F :** Look for acc/pep/gene in sequence name [False]  
                          - **tmhmm=FILE :** Path to TMHMM program [None]  
                            - **cleanup=T/F :** Remove TMHMM files after run [True]  
                              - **signalp=FILE :** Path to SignalP program [None]  
                                  
                                **Uses general modules:** copy, glob, os, string, sys, time  
                                **Uses RJE modules:** rje  
                                **Other modules needed:** None

---
