# Supplementary material for: Estimation and efficient computation of the true probability of recurrence of short linear protein sequence motifs in unrelated proteins
Source: BMC Bioinformatics. 2010 Jan 7;11:14. doi: 10.1186/1471-2105-11-14 (PMC2819990; doi:10.1186/1471-2105-11-14)
Supplement: Additional file 1 — Slimsuite software package. This ZIP file contains the slimsuite software package used with this submission. [file 1471-2105-11-14-S1.ZIP › slimsuite/RJE_SEQ Manual.pdf]

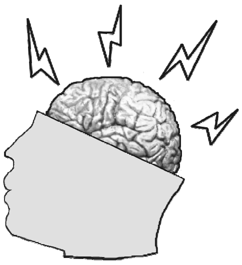

RJE_SEQ Biological sequence manipulation module

Richard J. Edwards (2005)

1: Introduction 3
1.1: Version 3
1.2: Copyright, License and Warranty 3
1.3: Using this Manual 3
1.4: Getting Help 4
1.5: Why use (and what is) RJE_SEQ? 4
1.6: Installation 4
1.6.1: Files Required for RJE_SEQ 4
1.6.2: Programs Used by RJE_SEQ 4
1.6.3: Setting up the INI File 5
1.6.4: Reducing Memory Requirements 5
2: Fundamentals 6
2.1: Running RJE_SEQ 6
2.1.1: The Basics 6
2.1.2: Interactivity and Verbosity settings 6
2.1.3: Other Options 6
2.2: Input Formats 7
2.2.1: Sequence formats 7
2.2.2: Mapping sequences from one file onto sequence details from another 7
2.2.3: Fasta format details 7
2.2.4: GnSpAcc Format (and the gnspacc=T/F option) 8
2.2.5: PAM Matrix 8
2.3: Output 9
3: Sequence Filters 10
3.1: Sequence/Database Feature Filters 10
3.1.1: Inherent Sequence Feature Filters 10
3.1.2: Filters from Lists or Files 10
3.2: Redundancy Checking 11
3.3: Sequence Filter Options 11
4: Sequence Utilities 12
4.1: Sequence Reformatting/Splitting 12
4.2: Fasta Files from BLAST 12
4.3: PAM Distance Matrix 12
5: Module Classes 12
5.1: The SeqList Class 12
5.2: The Sequence Class 12
5.3: The DisMatrix Class 12
6: Appendices 13
6.1: Appendix I: Command-line Options 13
6.1.1: How to Use this Section 13
6.1.2: Option Types 13
6.1.3: INI Files 13
6.1.4: Option Precedence 13
6.1.5: Command-line Options 14

6.2: Appendix II: Distributed Python Modules 16
6.3: Appendix III: Log Files...16
6.4: Appendix IV: Species Codes for IPI & EnsEMBL 17
6.5: Appendix V: Troubleshooting 18
6.6: Appendix VI: Glossary 18
6.7: Appendix VII: References..19

1: Introduction

Software manuals are boring: boring to write and probably even more boring to read. I have therefore tried to keep this one concise. However, given (a) my propensity to waffle, (b) the fact that I am a biologist and not a computer scientist, there is a good chance that the pleiotropic effect of this is a lack of clarity and/or coherence. For this I apologise, and encourage anyone out there to send in errata and/or suggested improvements.

This manual is designed to accompany the manuals for the programs that rje_seq.py forms a part of: BADASP, COMPASS, GABLAM, GOPHER, HAQESAC & PRESTO. Main details provided here cover sequence formats and filtering. Gluttons for punishment can get even more details in the **Appendices**. For the biological meat, however, I recommend the manuals and papers accompanying the other programs.

Like the software itself, this manual is a 'work in progress' to some degree. If the version you are now reading does not make sense, then it may be worth checking the website to see if a more recent version is available, as indicated by the **Version** section of the manual. Good luck.

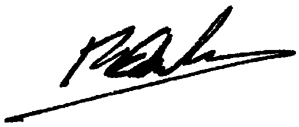

Rich Edwards, 2005.

1.1: Version

This manual is designed to accompany RJE_SEQ version 2.0.

The manual was last edited on 02 October 2006.

1.2: Copyright, License and Warranty

RJE_SEQ is Copyright © 2005 Richard J. Edwards.

This program is free software; you can redistribute it and/or modify it under the terms of the GNU General Public License as published by the Free Software Foundation; either version 2 of the License, or (at your option) any later version.

This program is distributed in the hope that it will be useful, but WITHOUT ANY WARRANTY; without even the implied warranty of MERCHANTABILITY or FITNESS FOR A PARTICULAR PURPOSE. See the GNU General Public License for more details.

The GNU General Public License should have been supplied with the RJE_SEQ program and is also available at www.gnu.org.

1.3: Using this Manual

As much as possible, I shall try to make a clear distinction between explanatory text (this) and text to be typed at the command-prompt etc. Command prompt text will be `written in Courier New` to make the distinction clearer. Program options, also called 'command-line parameters', will be **`written in bold Courier New`** (and coloured **`red`** for fixed portions or **`dark red`** for user-defined portions, such as file names etc.). Command-line examples will be given in (purple) *`italicised Courier New`*. Optional parameters will (where I remember) be `[`in square brackets`]`. Names of files supplied with RJE_SEQ will be marked in text by (dark yellow) **bold Times New Roman**. Please let me know if you find any confusing formatting errors!

1.4: Getting Help

Much of the information here is also contained in the documentation and websites for the tools that use this module (http://bioinformatics.ucd.ie/shields/redwards/) and the documentation of the Python modules themselves. A full list of command-line parameters can be printed to screen using the **help** option, with short descriptions for each one.

```
python rje_seq.py help
```

If none of the above are of help, then please e-mail me (richard.edwards@ucd.ie) whatever question you have. If it is the results of an error message, then please send me that and/or the log file (see **Output**) too. Usually, it will be a problem with the input files (possibly formatting) but there are probably still a few bugs in there somewhere too.

1.5: Why use (and what is) RJE_SEQ?

RJE_SEQ is primarily a module for use within other programs that contains classes for storing sequence data and a number of methods for manipulating sequences – loading, saving, filtering, aligning, BLASTing etc. In addition its use in these other programs, RJE_SEQ is also functional if called directly from the command-line, which is mainly of use for extracting sequences from local databases and/or filtering sequence files to contain (or avoid) specific species or accession numbers etc. or remove redundancy.

1.6: Installation

RJE_SEQ is distributed as a number of open source Python modules. It should therefore work on any system with Python installed without any extra setup required – simply copy the relevant files to your computer and run the program (see **2.1: Running RJE_SEQ**, below.)

If you do not have Python, you can download it free from **www.python.org** at http://www.python.org/download/. The modules are written in **Python 2.4**. The Python website has good information about how to download and install Python but if you have any problems, please get in touch and I will help if I can.

1.6.1: Files Required for RJE_SEQ

The following files are required for RJE_SEQ to run correctly. All these files should have been provided in the download zip file. The Python Modules are open source and may be changed if desired, although please give me credit for any useful bits you pillage. I cannot accept any responsibility if you make changes and it stops working, however! The additional files may all be replaced with other files in the correct format. These files are described later in this manual and/or in the Appendix.

Python Modules (*.py): rje, rje_blast, rje_dismatrix, rje_pam, rje_seq, rje_sequence, rje_uniprot

Additional Files: jones.pam

1.6.2: Programs Used by RJE_SEQ

In addition to the python modules listed above, RJE_SEQ makes use of the following published programs. These are freely available for downloading and installing. It is recommended that the user downloads and installs these programs according to the instructions given on the appropriate website. These programs are only needed for certain parameter settings.

ALIGN: This is part of the Fasta package (Pearson 1994, 2000) and can be downloaded from the University of Virginia: http://fasta.bioch.virginia.edu/. Make sure that align is part

of the download. For some reason it seems to have been dropped from later packages. You may need to install an earlier package first (e.g. 2.1) and then a later package. **NB.** This has been largely replaced by the GABLAMO method of generating global alignment statistics (Edwards & Davey, in prep) and will be phased out of use in RJE_SEQ.

BLAST: BLAST (Altschul et al. 1990) is a very well-known and widely used homology search tool and is freely available for download from NCBI at: http://www.ncbi.nlm.nih.gov/blast/download.shtml.

CLUSTALW: ClustalW (Higgins and Sharp 1988, Thompson et al. 1994) is an old stalwart for bioinformatics and is freely available from EMBL: ftp://ftp-igbmc.u-strasbg.fr/pub/ClustalW/. Note that CLUSTALW is used primarily as an alignment backup for MUSCLE (below) and may not be needed.

MUSCLE: MUSCLE (Edgar 2004) is a newer multiple alignment program available from http://www.drive5.com/muscle.

1.6.3: Setting up the INI File

It is recommended that a file named **rje.ini** or **rje_seq.ini** is made and placed in the same directory as the **rje_seq.py** program. This file should contain the paths to the above programs:

blastpath=PATH

fastapath=PATH

clustalw=PATH

muscle=PATH

Note that for BLAST and FASTA, the **PATH** is the directory in which programs can be found, while for ClustalW and MUSCLE the actual program commands themselves must be included. This is to make it easier to replace these programs with alternatives. (See Error! Reference source not found.**Replacing Components with Other Programs**.) See the included **haqesaq.ini** file for an example. If running in windows, it is also advisable to add the **win32=T** command to the ***.ini** file.

NB. For **PATH** variables, directories should be separated by a forward slash (**/**). If paths contain spaces, they should be enclosed in double quotes: **path="example path"**. It is recommended that paths do not contain spaces as function cannot be guaranteed if they do.

1.6.4: Reducing Memory Requirements

By default, RJE_SEQ will try to load the entire sequence dataset into memory (**autoload=T**). For very large datasets, however, this may be undesirable. The **memsaver=T** option can be used to avoid this and, as much as possible, process data on the fly without loading it all first. Note that this will not currently work for all methods. Please report any problems.

The **cwcut=X** option can also be used to make your computer use ClustalW rather than MUSCLE for its alignments. The **X** in this case is the total number of amino acids in the dataset. Below **X**, MUSCLE is used. Above **X**, ClustalW is used. Setting this quite low (**cwcut=1000**) will mean that ClustalW is used nearly all the time.

2: Fundamentals

2.1: Running RJE_SEQ

2.1.1: The Basics

If you have python and the other relevant files installed on your system (see **1.6: Installation**), you should be able to run RJE_SEQ directly from the command line in the form:

```
python rje_seq.py seqin=FILENAME
```

If running in Windows, you can just double-click the **rje_seq.py** file and enter command-line options when prompted. If running from the command-line and the named file does not exist, or contains no sequences, then this same prompt will be given (unless **i=-1** as described below).

Note: If filenames contain spaces, they must be enclosed in double quotes, e.g.

python rje_seq.py seqin="example with spaces.fas"

2.1.2: Interactivity and Verbosity settings

By default, RJE_SEQ will run through to most bits without any user-interaction and then pause to ask questions where required. Both the level of interactivity and the amount printed to screen can be altered, using the interactivity [**i=X**] and verbosity [**v=X**] command-line options, respectively, where **X** is the level from none (-1) to lots (2+). Although in theory **i=-1** and **v=-1** will ask for nothing and show nothing, there is a good chance that some print statements will have escaped in these early versions of the program. Please report any irritations.

Note: The Interactivity and Verbosity settings are still under development. Please send suggestions of things you would like more or less prompts for.

2.1.3: Other Options

There are a number of parameters that can be set by the user. These are described in **6.1: Appendix I: Command-line Options** and in the relevant sections of this manual. These may be given after the run command, as above, or loaded from a ***.ini** file (see **6.1.3: INI Files** details).

There are essentially three types of command-line option:

1. Those that require a value (numerical or text), **option=X**. Those that require a filename as the value will be witten: **option=FILE**
2. True/False (On/Off) options, **option=T/F**. For these options:
    a. **option=F** and **option=F**alse are the same and turn the option off
    b. **option**, **option=T** and **option=True** are the same and turn the option on
3. List options. These are like the value options but have multiple values, separated by commas: **option=X,Y**. Where **..** is used, the number elements is optional, e.g. **option=X,Y,..,Z** could take **option=X** or **option=A,B,C,D**. Where **option=LIST** is used, the number of elements is optional and **LIST** could actually be the name of a file containing the list of elements.

2.2: Input Formats

2.2.1: Sequence formats

RJE_SEQ is designed to deal, in some capacity, with the following sequence formats given to the program using the **seqin=FILE** command:

1. Fasta format. This is the recommended sequence format and is used for most functionality. In the majority of cases, sequences will be converted to fasta format. Sequences may be aligned or unaligned. (See **2.2.3: Fasta format details**2.2.3: for more information.)
2. UniProt download. A basic uniprot download can also be used as sequence input, providing extra information that is useful for some applications.
3. Phylip format. This should be accepted for input but is not recommended and is not fully supported. See PHYLIP documentation for details of this format.
4. ClustalW alignment format. Output from the ClustalW program can be read in by RJE_SEQ, which will then convert it to Fasta format.
5. Fastacmd format. This is a file of sequence names that can be extracted from a BLAST formatted database using the **fastacmd** utility. This database should be named using the **fasdb=FILE** command. This second file can be a fasta file rather than a BLAST database, in which case RJE_SEQ will format the database with the **formatdb** utility. This option requires correct installation of BLAST (Altschul, et al. 1990) and correct direction to the directory containing the utilities with the **blastpath=PATH** option.

To restrict input to a list of accession numbers in the given file, use the (**acclist=LIST**), where **LIST** could be a filename or a comma-separated list of accession numbers (`acclist=acc1,acc2,acc3` etc.). See also **3: Sequence Filters**.

2.2.2: Mapping sequences from one file onto sequence details from another

In certain circumstances you may wish to use the names from one sequence file and the sequences for another. The most obvious time for this is if ClustalW, which will only allow sequence names of up to 30 letters, has been used to align sequences and you wish to retain full description lines. To do this, load the file with full sequence names using **seqin=FILE** and then use the **mapseq=FILE** option to give the file with the replacement sequences. These will be mapped onto the original sequence names using either the first word of the name, which should be unique, or the accession number. The **mapseq=FILE** sequence file may contain more sequences than the **seqin=FILE** file; any extra sequences will be ignored. If the **mapseq=FILE** sequence file contains *less* sequences than the **seqin=FILE** file then an error will be returned and the option to quit given. If the option to quit is declines then any of the original sequences that are not replaced will retain their original sequence.

2.2.3: Fasta format details

RJE_SEQ is quite flexible about the precise details of the fasta format file used for sequence input. However, to get maximum utility and allow differentiation of sequences and source databases, it is recommended to use downloads from GenBank, UniProt and/or EnsEMBL. Alternatively, a file with names in 'GnSpAcc' format (see below) should be used as input. Fasta input may be aligned or unaligned.

The basic requirement for fasta sequences is that descriptions should be on one line that starts '>' and is followed by one or more lines containing the actual sequence. The first word in each description should be unique. *e.g.*

```
>Seq1 And its description
SEQUENCE-ONE-GOES-HERE
>Seq2
---GAPS--ARE--ALLOWED-
AS-ARE-MULTIPLE-LINES
```

Most databases and homology search results *etc.* can be downloaded in fasta format.

2.2.4: GnSpAcc Format (and the gnspacc=T/F option)

The **gnspacc=T** command reformats the names of the input sequences from something like:

```
>ENSP00000223233 blah blah blah
```

to

```
>ens_HUMAN__ENSP00000223233 blah blah blah
```

It is not necessary, and an be switched off with **gnspacc=F**, but does make it much easier to work out what species each sequence is from or, at least, whether they are the same species. The first word of the description always becomes:

>**GN_SP__ACC**, where:

- **GN** is a gene or database identifier.

- **SP** is a species code. This is generally the Uniprot species code or the best guess the program can make (namely the first three letters of the genus and the first two letters of the species).

- **AC** is the accession number.

This is done because the first word must be unique for each sequence and, for clustalW, the first 30 characters must be unique. It always should be but if you get an error relating to the names, this may be one of the potential causes.

2.2.5: PAM Matrix

The PAM matrix contains amino acid substitution probabilities and is used when calculating the PAM distance between any two branches. A basic PAM matrix (Jones et al. 1992) is available with the program in the file **jones.pam** (and is known as JTT, I believe). Alternative files can be given using the **pamfile=FILE** option. The important part of this file is the top section, which has the single letter amino acid codes on the first line, followed by the PAM1 matrix, where each subsequent line consists of an amino acid code and the probability of that aa being substituted by each other aa, in the order given in the first line:

```
A  R  N  D  C  Q  E  G  H  I  L  K  M  F  P  S  T  W  Y  V
Ala 0.98754 0.00030 0.00023 0.00042 0.00011 0.00023 0.00065 …
Arg 0.00044 0.98974 0.00019 0.00008 0.00022 0.00125 0.00018 …
Asn 0.00042 0.00023 0.98720 0.00269 0.00007 0.00035 0.00036 …
…
Val 0.00226 0.00009 0.00007 0.00016 0.00012 0.00008 0.00027 …
```

Note that the amino acids must be in the same order in both columns and rows. See http://www.bioinformatics.rcsi.ie/~redwards/gasp/ for more details.

2.3: Output

RJE_SEQ can save sequences in the following formats, each with its own identifying file extension:

Format	Description	Extension	Reformat
Fasta	Generally sequences all on one line and converted to GnSpAcc format unless `gnspacc=F` (see above).	*.fas	fasta
Phylip	See PHYLIP (Felsenstein 2005) documentation for details.	*.phy	phylip
Scanseq	Scanseq parallel version input format. One line per sequence: accession number, space, sequence.	*.scanseq	scanseq
Teiresias	Teiresias input format. This is a customised fasta format with accession number only in the description line	*.fas	teiresias
AccList	A list of accession numbers.	*.acc	acclist
Fastacmd	A list of fastacmd names (the first "word" of the description line).	*.txt	fastacmd

Format: Format name. **Description:** Description of format. **Extension:** Default file extension for output files. **Reformat:** option for `reformat=X` parameter.

All of these can be output, after any filtering options (see **3: Sequence Filters**) and manipulations (see **0Sequence Utilities**) using the `reformat=X` option, where `X` is one of: fasta, phylip, scanseq, teiresias, acclist, fastacmd.

Additional output may be produced by individual utilities. See **0Sequence Utilities** for details.

3: Sequence Filters

There are a number of sequence filters that can be applied to the inputted sequences. These fall broadly into two categories

1. Sequence/Database feature filters
2. Redundancy Checking

The processed dataset can then be saved using the **filterout=FILE** or **seqout=FILE** commands. **filterout=FILE** will save in fasta format, whereas **seqout=FILE** will save in the format determined by **format=X**.

3.1: Sequence/Database Feature Filters

Sequences can be filtered according to several options, both for inherent features and on the basis of filters given to the program in lists or files.

3.1.1: Inherent Sequence Feature Filters

Input may be filtered according to sequence length using the **minlen=X** and **maxlen=X** options. E.g. *minlen=10 maxlen=100*, will exclude sequences <10aa in or >100aa in length (ignoring gaps). For aligned input, gappy sequences can be removed using the **maxgap=X** option. E.g. *maxgap=0.5* will remove any sequences with gaps in over 10% of alignment positions. (This may be over-ridden by special use of **maxgap=X** by other programs using this module, such as HAQESAC, which use the **gapfilter=F** option to turn this automated filter off.)

Input can be restricted to sequences belonging to recognised databases using **dbonly=T**. These databases, and their hierarchy for redundant sequence removal (below), is set by the **dblist=X,Y,..,Z** option, where **X,Y,..,Z** constitutes a list of databases in order of preference (good to bad). Additionally, **unkspec=F** will removes any sequences for which a species code cannot be determined from the input format.

3.1.2: Filters from Lists or Files

A number of additional filters can be applied using information in lists or files as set by the **goodX=LIST** (retention) and **badX=LIST** (exclusion) options, where **X** is one of the following:

- acc = list of accession numbers
- seq = list of sequence names
- spec = list of species codes
- db = list of source databases [sprot,ipi,uniprot,trembl,ens_known,ens_novel,ens_scan]
- desc = list of terms that, at least one of which must be in description line

The **LIST** element can be a list **X,Y,..,Z** or a file containing the relevant terms. E.g. *goodspec=HUMAN,MOUSE,RAT baddesc=bad_desc.txt* would only retain sequences annotated as having the species codes HUMAN, MOUSE or RAT and would exclude any of these sequences that have any of the terms from the file **bad_desc.txt** in their description lines.

3.2: Redundancy Checking

If the same sequence may be present several times due to concatenating datasets etc. then the `accnr=T` option can be used to check for redundant Accession Numbers/Names upon loading the sequences. Further comparisons of the sequences themselves can then be used to check for redundancy using the `seqnr=T` option. By default, this will remove 100% identical sequences. This can be relaxed using `nrid=X` or `nrsim=X` to set the Sequence Identity or Similarity thresholds, respectively, as calculated by the GABLAM algorithm (Edwards and Davey 2006), which uses BLAST (Altschul, et al. 1990) local alignments as a basis. During the redundancy filter, annotated fragments are removed in preference to full length sequences. Then sequences are kept according to the database hierarchy (set by the `dblist=X,Y,..,Z` option) and, within databases, longer sequences are preferentially kept over shorter ones. The `specnr=T` option will restrict the redundancy filter to comparing sequences of the same species.

3.3: Sequence Filter Options

Option	Description	Default
`minlen=X`	Minimum length of sequences	[0]
`maxlen=X`	Maximum length of sequences (<=0 = No maximum)	[0]
`maxgap=X`	Maximum proportion of sequence that may be gaps (<=0 = No maximum)	[0]
`dbonly=T/F`	Only allow sequences from listed databases	[False]
`dblist=LIST`	List of databases in order of preference (good to bad)	[sprot,ipi,uniprot,trembl,ens_known,ens_novel,ens_scan]
`unkspec=T/F`	Sequences of unknown species are allowed	[False]
`goodX=LIST`	Only keeps sequences meeting the requirement of `LIST` (`X,Y,..,Z` or a `FILE` which contains a list).<br>➢ `goodacc` = list of accession numbers<br>➢ `goodseq` = list of sequence names<br>➢ `goodspec` = list of species codes<br>➢ `gooddb` = list of source databases<br>➢ `gooddesc` = list of terms that, at least one of which must be in description line	[None]
`badX=LIST`	As `goodX` but excludes rather than retains sequences	[None]
`accnr=T/F`	Check for redundant Accession Numbers/Names on loading sequences.	[False]
`seqnr=T/F`	Make sequence Non-Redundant	[False]
`specnr=T/F`	Non-Redundancy within same species only	[False]
`nrid=X`	%Identity (GABLAM) cut-off for Non-Redundancy	[100.0]
`nrsim=X`	%Similarity (GABLAM) cut-off for Non-Redundancy	[100.0]

4: Sequence Utilities

4.1: Sequence Reformatting/Splitting

Sequences may be reformatted (see **2.3: Output**) using the **seqout=FILE** and **format=X** options. In addition, **split=X** will split the output file into numbered files, each containing upto **X** sequences. This is useful for webservers like TMHMM, which have a maximum number of sequences that can be submitted at once. E.g. If a file had 1,300 sequences, *seqout=mysplit.fas format=fasta split=1000* would produce two files: **mysplit.1.fas** of the first 1,000 sequences and **mysplit.2.fas** of the remaining 300 sequences.

4.2: Fasta Files from BLAST

The **blast2fas=FILE1,FILE2,...,FILEn** option will BLAST (Altschul, et al. 1990) sequences against list of databases and compile a fasta file of results per query, saving the results in files named **AccNum.blast.fas**, where **AccNum** is the accession number of the query. If these files already exist, they will be appended. BLAST settings are controlled by the **rje_blast.py** module. (*python rje_blast.py help* for parameter settings.)

4.3: PAM Distance Matrix

The **pamdis** command will generate an all by all PAM distance matrix for the input sequences. PAM distances are calculated as described in the GASP (Edwards 2004, Edwards and Shields 2004) manual.

5: Module Classes

Details of the classes in this module and their implementation in other python programs will be given here. In the meantime, please contact the author with any questions.

5.1: The SeqList Class

5.2: The Sequence Class

5.3: The DisMatrix Class

6: Appendices

6.1: Appendix I: Command-line Options

6.1.1: How to Use this Section

This section lists all the Command-line options than may be of use when using RJE_SEQ. Note that different options are associated with different modules. These are indicated by the name of the module given (**in brackets**). Default values are given [in square brackets]. Not all the options for a given module are listed here but can be found by printing the `__doc__` attribute of the module at a Python prompt, or using the **`help`** option:

`print rje_seq.__doc__` (in Python)

`python rje_seq.py help` (commandline)

This section has not been completed. For now, the listing provided as part of the module documentation is given. This shall be expanded with time. (Hopefully soon.) In the meantime, please contact me if you want any further details of a specific option and/or advice as to when (not) to use it.

6.1.2: Option Types

There are essentially three types of command-line option:

1. Those that require a value (numerical or text), **`option=X`**. Those that require a filename as the value will be witten: **`option=FILE`**
2. True/False (On/Off) options, **`option=T/F`**. For these options:
    a. **`option=F`** and **`option=F`**alse are the same and turn the option off
    b. **`option`**, **`option=T`** and **`option=True`** are the same and turn the option on
3. List options. These are like the value options but have multiple values, separated by commas: **`option=X,Y`**. Where **`..`** is used, the number elements is optional, e.g. **`option=X,Y,..,Z`** could take **`option=X`** or **`option=A,B,C,D`**. Where **`option=LIST`** is used, the number of elements is optional and **`LIST`** could actually be the name of a file containing the list of elements.

6.1.3: INI Files

As well as feeding commands in on the command-line, any options listed can also be save in a plain text file and called using the option **`ini=FILE`**. Automatically, the program will read in any options from the file **haqesac.ini** and **rje.ini**, if present.

6.1.4: Option Precedence

Later options will supersede earlier ones if they are mutually exclusive. Options from an ini file will be inserted into the list at the point the ini file is called. (At the start for **rje.ini**.) This means that ini file options can be over-ruled, e.g.

`rje_seq.py ini=eg.ini i=1` will supersede any interactivity setting in **eg.ini** with **`i=1`**.

`rje_seq.py i=1 ini=eg.ini` will use any interactivity setting in **eg.ini** and over-rule **`i=1`**.

6.1.5: Command-line Options

Option	Description	Default	Module
	**General Dataset Input/Output**		
seqin=FILE	Loads sequences from FILE	[None]	rje_seq
fasdb=FILE	BLAST database for fastacmd extraction	[None]	rje_seq
query=X	Selects query sequence by name (or part of name, e.g. Accession Number)	[None]	rje_seq
basefile=X	Basic 'root' for all files X.*	['root' of seqin=FILE]	rje_seq
acclist=LIST	Extract only AccNums in list.	[None]	rje_seq
mapseq=FILE	Map sequences from FILE onto names from seqin=FILE.	[None]	rje_seq
seqout=FILE	Name for output file.	[None]	rje_seq
filterout=FILE	Name for output file after filtering.	[None]	rje_seq
reformat=X	Output format for sequences (fasta, phylip, scanseq, teiresias, acclist, fastacmd)	[None]	rje_seq
gnspacc=T/F	Convert sequences into gene_SPECIES__AccNum format wherever possible.	[True]	rje_seq
autoload=T/F	Whether to automatically load sequences	[True]	rje_seq
v=X	Sets verbosity (-1 for silent)	[0]	rje
i=X	Sets interactivity (-1 for full auto)	[0]	rje
d=X	Data output level (0-3)	[1]	haqesac
log=FILE	Redirect log to FILE	[haqesac.log or basefile.log]	rje
newlog=T/F	Create new log file.	[False]	rje
	**Sequence Filtering**		
minlen=X	Minimum length of sequences	[0]	rje_seq
maxlen=X	Maximum length of sequences (<=0 = No maximum)	[0]	rje_seq
maxgap=X	Maximum proportion of sequence that may be gaps (<=0 = No maximum)	[0]	rje_seq
dbonly=T/F	Only allow sequences from listed databases	[False]	rje_seq
dblist=LIST	List of databases in order of preference (good to bad)	[sprot,ipi,uniprot,trembl, ens_known, ens_novel,ens_scan]	rje_seq

Option	Description	Default	Module
unkspec=T/F	Sequences of unknown species are allowed	[False]	rje_seq
goodX=LIST	Only keeps sequences meeting the requirement of LIST (X,Y,..,Z or a FILE which contains a list).<br>➢ goodacc = list of accession numbers<br>➢ goodseq = list of sequence names<br>➢ goodspec = list of species codes<br>➢ gooddb = list of source databases<br>➢ gooddesc = list of terms that, at least one of which must be in description line	[None]	rje_seq
badX=LIST	As goodX but excludes rather than retains sequences	[None]	rje_seq
accnr=T/F	Check for redundant Accession Numbers/Names on loading sequences.	[False]	rje_seq
seqnr=T/F	Make sequence Non-Redundant	[False]	rje_seq
specnr=T/F	Non-Redundancy within same species only	[False]	rje_seq
nrid=X	%Identity (GABLAM) cut-off for Non-Redundancy	[100.0]	rje_seq
nrsim=X	%Similarity (GABLAM) cut-off for Non-Redundancy	[100.0]	rje_seq
autofilter=T/F	Whether to apply sequence filters upon loading	[True]	rje_seq
	**System Info**		
blastpath=PATH	Path to BLAST programs * Use forward slashes (/)	['c:/bioware/blast/']	rje_blast
fastapath=PATH	Path to FASTA programs * Use forward slashes (/)	['c:/bioware/fasta/']	rje_seq
clustalw=PATH	Path to CLUSTALW program * Use forward slashes (/)	['c:/bioware/clustalw.exe']	rje_seq
muscle=PATH	Path to MUSCLE * Use forward slashes (/)	['c:/bioware/muscle.exe']	rje_seq
win32=T/F	Run in Win32 Mode	[False]	rje
memsaver=T/F	Run in "Memory Saver" mode	[False]	rje

6.2: Appendix II: Distributed Python Modules

This appendix is also incomplete and is liable to go out of date. Please look at the documentation within the modules themselves for more details. (And, if you're brave, look at the code!)

Module	Description	Classes
rje_seq	Contains Classes and methods for sets of DNA and protein sequences. (Currently only protein sequences supported.)	SeqList, Sequence, DisMatrix
rje	General module containing Classes used by all my scripts plus a number of miscellaneous methods.	RJE_Object_Shell, RJE_Object, Info, Out, Log
rje_blast	Performs BLAST searches and loads results into objects.	BLASTRun, BLASTSearch, BLASTHit, PWAln
rje_dismatrix	Contains Classes and methods for Distance Matrix	DisMatrix
rje_pam	This module handles functions associated with PAM matrices. A PAM1 matrix is read from the given input file and multiplied by itself to give PAM matrices corresponding to greater evolutionary distance. (PAM1 equates to one amino acid substitution per 100aa of sequence.)	PamCtrl, PAM
rje_sequence	Contains Classes and methods for individual sequences	Sequence
rje_uniprot	Contains methods for parsing UniProt info	UniProt

6.3: Appendix III: Log Files

This part of the manual has not yet been written. Sorry! Contact the author if you have questions about the log files.

6.4: Appendix IV: Species Codes for IPI & EnsEMBL

The following EnsEMBL and IPI species and species codes are recognized by **rje_sequence.py**:

Species	Common Name	Species Code
Aedes_aegypti	Yellow Fever Mosquito	AEDAE
Anopheles gambiae	Malaria Mosquito	ANOGA
Apis mellifera	Bee	APIME
Bos taurus	Cow	BOVIN
Caenorhabditis elegans	Nematode	CAEEL
Canis familiaris	Dog	CANFA
Ciona intestinalis	Sea squirt	CIOIN
Ciona savignyi	Sea squirt	CIOSA
Danio rerio	Zebrafish	BRARE
Dasypus novemcinctus	Nine-banded armadillo	DASNO
Drosophila melanogaster	Fruitfly	DROME
Echinops telfairi	Madagascar Hedgehog	ECHTE
Fugu rubripes	Pufferfish	FUGRU
Gallus gallus	Chicken	CHICK
Gasterosteus aculeatus	Alaskan Stickleback	GASAC
Homo sapiens	Human	HUMAN
Loxodonta africana	Elephant	LOXAF
Macaca mulatta	Macaque	MACMU
Monodelphis domestica	Opossum	MONDO
Mus musculus	Mouse	MOUSE
Oryctolagus cuniculus	Rabbit	RABIT
Pan troglodytes	Chimp	PANTR
Rattus norvegicus	Rat	RAT
Saccharomyces cerevisiae	Yeast	YEAST
Tetraodon nigroviridis	Pufferfish	TETNG
Xenopus tropicalis	Frog	XENTR

To add more species, the **extractDetails()** method of the **Sequence** class needs to be edited. Please contact the author for assistance.

6.5: Appendix V: Troubleshooting

Currently, this is a small section as I have not had enough feedback to have FAQs, or anything like that. Here is a list of things that I think MAY cause problems to the unwary:

- Giving file names with spaces without enclosing them in "double quotes". (Otherwise only the first word will be taken as the filename.) It is recommended to use paths and filenames without spaces if possible.
- Incorrect formatting of input files (see **2.2: Input**).
- I'm not sure when but there is a possibility of problems if running in Windows without the `win32` option, especially when calling accessory applications that need different system calls.
- The `cwcut=X` option can also be used to make your computer use ClustalW rather than MUSCLE for its alignments. The `X` in this case is the total number of amino acids in the dataset. Below `X`, MUSCLE is used. Above `X`, ClustalW is used. Setting this quite low (`cwcut=1000`) will mean that ClustalW is used nearly all the time.
- Check sequence names. The first word must be unique for each sequence and, for clustalW, the first 30 characters must be unique. Special characters (other than – and _) in the first word may give some programs problems.
- The `memsaver=T` option may not work for all methods. Please report any problems.
- During loading, termination markers (*) are stripped and unrecognised characters are replaced with Xs. Although this should have no impact on most applications, if you are using customised scripts then you should be aware of this as it may cause downstream problems. All changes are logged in the log file.

6.6: Appendix VI: Glossary

Please e-mail with any terms used in the manual that you do not understand, and I will endeavour to add them to the glossary. This is not, however, designed to be a comprehensive encyclopaedia but rather a starting point for finding more information. Where possible, useful links will be suggested but a quick search on Google normally does the job nicely.

- **ML.** Maximum Likelihood
- **PAM.** Point Accepted Mutation
- **NSF.** Newick Standard Format.
- **MSA.** Multiple Sequence Alignment.

6.7: Appendix VII: References

Altschul, S.F., Gish, W., Miller, W., Myers, E.W. and Lipman, D.J. (1990) Basic local alignment search tool, *J Mol Biol*, **215**, 403-410.

Edgar, R.C. (2004) MUSCLE: a multiple sequence alignment method with reduced time and space complexity, *BMC Bioinformatics*, **5**, 113.

Edwards, R.J. (2004) GASP: Gapped Ancestral Sequence Prediction. http://www.bioinformatics.rcsi/~redwards/gasp/

Edwards, R.J. and Davey, N.E. (2006) GABLAM: Global Alignment from BLAST Local Alignment Modules. http://bioinformatics.ucd.ie/shields/software/gablam/

Edwards, R.J. and Shields, D.C. (2004) GASP: Gapped Ancestral Sequence Prediction for proteins, *BMC Bioinformatics*, **5**, 123.

Felsenstein, J. (2005) PHYLIP (Phylogeny Inference Package) version 3.6., *Distributed by the author. Department of Genome Sciences, University of Washington, Seattle.*,

Higgins, D.G. and Sharp, P.M. (1988) CLUSTAL: a package for performing multiple sequence alignment on a microcomputer, *Gene*, **73**, 237-244.

Jones, D.T., Taylor, W.R. and Thornton, J.M. (1992) The rapid generation of mutation data matrices from protein sequences, *Comput Appl Biosci*, **8**, 275-282.

Pearson, W.R. (1994) Using the FASTA program to search protein and DNA sequence databases, *Methods Mol Biol*, **24**, 307-331.

Pearson, W.R. (2000) Flexible sequence similarity searching with the FASTA3 program package, *Methods Mol Biol*, **132**, 185-219.

Thompson, J.D., Higgins, D.G. and Gibson, T.J. (1994) CLUSTAL W: improving the sensitivity of progressive multiple sequence alignment through sequence weighting, position-specific gap penalties and weight matrix choice, *Nucleic Acids Res*, **22**, 4673-4680.
