# Supplementary material for: Estimation and efficient computation of the true probability of recurrence of short linear protein sequence motifs in unrelated proteins
Source: BMC Bioinformatics. 2010 Jan 7;11:14. doi: 10.1186/1471-2105-11-14 (PMC2819990; doi:10.1186/1471-2105-11-14)
Supplement: Additional file 1 — Slimsuite software package. This ZIP file contains the slimsuite software package used with this submission. [file 1471-2105-11-14-S1.ZIP › slimsuite/SLiMFinder Manual.pdf]

SLiMFinder:

Short Linear Motif Finder

Richard J. Edwards, Norman E. Davey & Denis C. Shields (2007)

1 Introduction....4
1.1 Version....4
1.2 Using this Manual....4
1.3 What is SLiMFinder?....4
1.4 Getting Help....5
1.4.1 Something Missing?....5
1.5 Citing SLiMFinder....5
2 Fundamentals....6
2.1 Running SLiMFinder....6
2.1.1 The Basics....6
2.1.2 Options....6
2.2 Input....6
2.2.1 Optional Input I: Amino acid frequency files....6
2.2.2 Optional Input II: Specific Motifs of Interest....6
2.2.3 Optional Input III: Protein Clusters....7
2.2.4 Optional Input IV: Query Sequences....7
2.2.5 Optional Input V: Protein Alignments....7
2.3 Output....8
2.3.1 Main SLiMFinder Results Table....8
2.3.2 Sequence relationship output....8
2.3.3 SLiMDisc Output....8
2.3.4 TEIRESIAS Output....8
2.3.5 Extra Dataset-specific Output....8
2.4 Commandline Options....9
2.5 Rerunning SLiMFinder (and Pickling)....12
2.5.1 Pickle naming conventions....12
2.6 Secondary SLiMFinder Functions....12
2.6.1 Simple Identification of Sequences containing Motifs....12
2.6.2 Randomising Batch Datasets....12
2.7 The SLiMFinder Webserver....13
2.7.1 Interactive Masking....13
2.7.2 Job Submission....13
2.7.3 Webserver results page....15
3 SLiMFinder Methods & Definitions....16
3.1 SLiMFinder Program Overview....16
3.2 Unrelated Protein Clusters (UPC)....17
3.3 SLiM Definitions....18
3.3.1 SLiM Anatomy....18
3.3.2 SLiMs and SLiMBuild Dimers....19
3.3.3 Information Content (IC)....19
3.3.4 Motif Occurrences and Support....19
4 SLiMBuild Motif Construction....20
4.1 Input Masking....20
4.1.1 Disorder masking....20
4.1.2 UniProt Features....20
4.1.3 Low Complexity Masking....20
4.1.4 N-terminal Methionines....20
4.1.5 Masking by Case....20
4.1.6 Relative Conservation Masking....21
4.1.7 Masking pre-defined motifs....21
4.2 SLiM Construction....21
4.2.1 SLiMBuild dimer construction....24
4.2.2 SLiMBuild motif extension....24

4.2.3 SLiMBuild ambiguity ... 24
4.2.4 Final SLiM Support ... 25
4.3 SLiMBuild versus TEIRESIAS ... 25

5 SLiMChance Motif Probability Methods ... 26
5.1 Amino Acid Frequencies ... 26
5.2 SLiMChance motif probability estimation ... 26
5.2.1 SLiMChance probabilities per UPC ... 27
5.2.2 SLiMChance probabilities per dataset ... 28
5.2.3 SLiMChance significance values ... 28
5.3 Increasing significance with restricted searches ... 29
5.3.1 Query sequences and focus groups ... 29
5.3.2 MustHave amino acid restrictions ... 29

6 SLiMFinder Output Explained ... 30
6.1 Main SLiMFinder Output (slimfinder.csv) ... 30
6.1.1 Overwriting, appending and backing up results ... 30
6.1.2 Main Output Fields ... 30
6.2 Sequence Relationships (*.upc, *.self.blast, *.dis.txt, *.phydis.txt) ... 30
6.2.1 UPC Definitions ... 30
6.2.2 Distance Matrices ... 31
6.3 Dataset rank files (*.rank) ... 32
6.4 Motif Clouds (*.cloud.txt) ... 32
6.5 Motif Occurrence Tables (*.occ.csv, *.dat.rank & *.out) ... 32
6.6 Sequence files (*.motifaln.fas, *.maskaln.fas, *.mapping.fas, *.masked.fas, *.motifs) ... 32
6.7 CompariMotif Comparisons (*.compare.tdt) ... 33
6.8 XGMML Cytoscape Files (*.xgmml, *.cloud.xgmml) ... 33

7 SLiM Statistics and Filtering ... 35
7.1 Additional SLiM Calculations (slimcalc) ... 35
7.1.1 Surface Accessibility [SA] ... 35
7.1.2 Hydropathy [Hyd] ... 35
7.1.3 Disorder [IUP & Fold] ... 35
7.1.4 Complexity [Comp] ... 35
7.1.5 SLiM Conservation [Cons] ... 35
7.1.6 Extending Calculations to flanking regions ... 36
7.2 SLiM Conservation Calculations ... 36
7.2.1 Absolute Conservation [abs] ... 36
7.2.2 Positional Scoring [pos] ... 36
7.2.3 AA Property Scoring [prop] ... 38
7.2.4 Relative Conservation Scoring [rel] ... 38
7.2.5 Combined Scoring [all] ... 38
7.2.6 Motif ambiguity ... 38
7.2.7 Positional Weighting by Information Content ... 38
7.2.8 Homology Weighting ... 38
7.2.9 Gap Treatment ... 39
7.2.10 Taxonomic subgroupings ... 39
7.3 Protein Alignments for SLiMFinder ... 39
7.3.1 Using GOPHER to make orthologue alignments ... 39
7.4 Filtering output using SLiM Calculations ... 40
7.5 SLiM calculation/filtering options ... 40

8 Appendices ... 42
8.1 Troubleshooting & FAQ ... 42
8.2 QuickStart Guide ... 42
8.3 References ... 42

FIGURES

Figure 2.1. SLiMFinder webserver main page. 13
Figure 2.2. Interactive SLiMFinder input masking. 14
Figure 2.3. SLiMFinder webserver queue page. 14
Figure 2.4. Main SLiMFinder webserver results page. 15
Figure 3.1. SLiMFinder Overview. 16
Figure 3.2. Generation of UPC from BLAST results. 17
Figure 3.3. Anatomy of a SLiM. 18
Figure 4.1. SLiMBuild construction of motifs. 22
Figure 4.2. SLiMBuild Ambiguity. 23
Figure 6.1. Cytoscape visualisation of SLiMFinder XGMML output for LIG_CYCLIN_1. 33
Figure 6.2. Cytoscape visualisation of XGMML output for LIG_PCNA results. 34
Figure 7.1. Absolute Conservation. 37
Figure 7.2. Position-speciffc Conservation. 37

TABLES

Table 2.1. List of optional extra dataset-specific SLiMFinder outputs. 9
Table 2.2. SLiMFinder Commandline Options. 9
Table 4.1. Major advantages of SLiMFinder over TEIRESIAS for SLiM Discovery. 25
Table 5.1. Suggested use of Amino Acid Frequency options. 26
Table 6.1. Fields for main SLiMFinder Output. 31
Table 7.1. SLiM calculation/filtering options. 41

1 Introduction

This manual gives an overview of SLiMFinder as implemented in the slimfinder.py module. Because there are many options, this manual will probably not be fully comprehensive but aims to cover the basics and the most useful of the more advanced stuff. If anything is missing or needs clarification, please contact me. The fundamentals are covered in Chapter 2, Fundamentals, including input and output details. Later sections give more details on how the methods work and statistics are generated. General details about Command-line options can be found in the PEAT Appendices document included with this download. Details of command-line options specific to Slim Pickings can be found in the distributed readme.txt and readme.html files

Like the software itself, this manual is a 'work in progress' to some degree. If the version you are now reading does not make sense, then it may be worth checking the website to see if a more recent version is available, as indicated by the Version section of the manual. Furthermore, many options have been added to Slim Pickings over the past few weeks and not all of them have found their way into the manual yet. Check the readme on the website for up-to-date options etc. In particular, default values for options are subject to change and should be checked in the readme.

Good luck.

Rich Edwards, 2007.

1.1 Version

This manual is designed to accompany **SLiMFinder version 3.5**.

The manual was last edited on 25 June 2008.

1.2 Using this Manual

As much as possible, I shall try to make a clear distinction between explanatory text (this) and text to be typed at the command-prompt etc. Command prompt text will be `written in Courier New` to make the distinction clearer. Program options, also called 'command-line parameters', will be **`written in bold Courier New`** (and coloured **`red`** for fixed portions or **`dark red`** for user-defined portions, such as file names etc.). Command-line examples will be given in (purple) *`italicised Courier New`*. Optional parameters will (if I remember) be `[`in square brackets`]`. Names of files will be marked in normal text by (blue-grey) Times New Roman.

1.3 What is SLiMFinder?

Short linear motifs (SLiMs) in proteins are functional microdomains of fundamental importance in many biological systems. SLiMs typically consist of a 3 to 10 amino acid stretch of the primary protein sequence, of which as few as two sites may be important for activity, making identification of novel SLiMs extremely difficult. In particular, it can be very difficult to distinguish a randomly recurring "motif" from a truly over-represented one. Incorporating ambiguous amino acid positions and/or variable-length wildcard spacers between defined residues further complicates the matter.

SLiMFinder is an integrated SLiM discovery program building on the principles of the SLiMDisc software for accounting for evolutionary relationships (Davey et al. 2006). SLiMFinder is comprised of two algorithms:

1. SLiMBuild identifies convergently evolved, short motifs in a dataset. Motifs with fixed amino acid positions are identified and then combined to incorporate amino acid ambiguity and variable-length wildcard spacers. Unlike programs such as TEIRESIAS (Rigoutsos & Floratos 1998), which return all shared patterns, SLiMBuild accelerates the process and reduces returned motifs by explicitly screening out motifs that do not occur in enough unrelated proteins. For this, SLiMBuild uses the "Unrelated Proteins" (UP) algorithm of SLiMDisc in which BLAST is used to identify pairwise relationships. Proteins are then clustered according to these relationships into "Unrelated Protein Clusters" (UPCs), which are defined such that no protein in a UPC has a BLAST-detectable relationship with a protein in another UPC. If desired, SLiMBuild can be used as a replacement for TEIRESIAS in other software (use **`teiresias=T slimchance=F`** to emulate TEIRESIAS output).

2. SLiMChance estimates the probability of these motifs arising by chance, correcting for the size and composition of the dataset, and assigns a significance value to each motif. Motif occurrence probabilities are calculated independently for each UPC, adjusted the size of a UPC using the Minimum Spanning Tree algorithm from SLiMDisc. These individual occurrence probabilities are then converted into the total probability of seeing the observed motifs the observed number of (unrelated) times. These probabilities assume that the motif is known before the search. In reality, only over-represented motifs from the dataset are looked at, so these probabilities must be adjusted for the total number of motifs in the dataset. SLiMChance calculates the size of the "motif space" searched and corrects the significance accordingly. The returned corrected probability is an estimate of the probability of seeing ANY motif with that significance (or greater) from the dataset.

Where significant motifs are returned, SLiMFinder will group them into Motif "Clouds", which consist of physically overlapping motifs (2+ non-wildcard positions are the same in the same sequence). This provides an easy indication of which motifs may actually be variants of a larger SLiM and should therefore be considered together. Where pre-known motifs are also of interest, these can be given with the **`slimcheck=MOTIFS`** option and will be added to the output.

Additional Motif Occurrence Statistics, such as motif conservation, are handled by the rje_slimlist and rje_slimcalc modules. Please see the documentation for these modules for a full list of commandline options. Note that **`occfilter=LIST`** *does* affect the motifs returned by SLiMBuild and thus the TEIRESIAS output (as does min. IC and min. Support) but the overall Motif **`slimfilter=LIST`** *only* affects SLiMFinder output following SLiMChance calculations.

1.4 Getting Help

Much of the information here is also contained in the documentation of the Python modules themselves. A full list of command-line parameters can be printed to screen using the **`help`** option, with short descriptions for each one:

```
python slimfinder.py help
```

General details about Command-line options can be found in the PEAT Appendices document included with this download. Details of command-line options specific to Slim Pickings can be found in the distributed readme.txt and readme.html files.

If still stuck, then please e-mail me (**r.edwards@soton.ac.uk**) whatever question you have. If it is the results of an error message, then please send me that and/or the log file (see 2.3) too.

1.4.1 Something Missing?

As much as possible, the important parts of SLiMFinder are described in detail in this manual. If something is not covered, it is generally not very important and/or still under development, and can therefore be safely ignored. If, however, curiosity gets the better of you, and/or you think that something important is missing (or badly explained), please contact me.

1.5 Citing SLiMFinder

Please cite the main SLiMFinder publication (Edwards et al. 2007):

- Edwards RJ, Davey NE and Shields DC (2007). SLiMFinder: A Probabilistic Method for Identifying Over-Represented, Convergently Evolved, Short Linear Motifs in Proteins. *PLoS ONE*, **2**, e967.

When using **CompariMotif** (Edwards et al. 2008) results, or alignments generated using **GOPHER** (Edwards 2006), please cite the **SLiMDisc Webserver** paper (Davey et al. 2007). Disorder predictions should cite **IUPRED** (Dosztanyi et al. 2005).

2 Fundamentals

2.1 Running SLiMFinder

2.1.1 The Basics

If you have python installed on your system, you should be able to run SLiMFinder directly from the command line in the form:

```
python slimfinder.py
```

By default, SLiMFinder will run on all *.dat and *.fas files in the run directory. To run on a single file, use the **seqin=FILE** option. For the example provided in the distribution:

```
python slimfinder.py seqin=slim_eg.fas
```

A SLiMFinder webserver is also available at http://bioware.ucd.ie. See 2.7 for details.

2.1.2 Options

Command-line options are suggested in the following sections. General details about Command-line options can be found in the PEAT Appendices document included with this download. Details of command-line options specific to Slim Pickings can be found in the distributed readme.txt and readme.html files. These may be given after the run command, as above, or loaded from one or more *.ini files (see PEAT Appendices for details).

2.2 Input

Basic input for SLiMFinder is one or more sequence files in Fasta or UniProt format. This input will be masked according to the program's settings. See Chapter 4 for details. Sequence names may be altered by the program for compatibility (using the **gnspacc=T** option of rje_seq.py (see RJE_SEQ Manual for details).

2.2.1 Optional Input I: Amino acid frequency files

SLiMChance can optionally take external amino acid frequency files for its probability calculations, using the **aafreq=FILE** option. This can either be a fasta format sequence file, or a plain text file containing amino acid frequencies in two columns, with the headings "AA" and "FREQ", *e.g.*:

```
AA      FREQ
A       0.055854
C       0.020012
…
Y       0.026583
```

2.2.2 Optional Input II: Specific Motifs of Interest

SLiMFinder has a **slimcheck=FILE** option to search for specific motifs during SLiMFinder runs, regardless of their significance. This should be a file with a format recognised by **SLiMSearch** (Edwards 2007), which is a replacement for **PRESTO** (Edwards 2006) (see PRESTO documentation for details), which in its simplest form is a plain list of motif patterns. If there are not variable-length wildcards ({m,n}) then **FILE** can be replaced with a comma-separated list of motifs of interest. The *.motifs file produced if **extras=T** (see below) is in a compatible format and can be used directly. SLiMCheck motifs are added to the end of the main results file (see 2.3.1 and 6.1) but are not given a rank. For a more detailed analysis of sequences with a dataset of known motifs, please use **SLiMSearch**, which has many of the same input and processing options as SLiMFinder (Edwards 2007).

2.2.3 Optional Input III: Protein Clusters

SLiMFinder uses **BLAST** (Altschul et al. 1990) and **GABLAM** (Edwards & Davey 2006) to cluster related proteins into "Unrelated Protein Clusters" (UPCs) as described in 3.3.3. UPCs are treated as units such that multiple SLiM occurrences within a UPC are treated as a single occurrence. The UPCs generated by SLiMFinder are output into a summary file (see 2.3.2). If, for whatever reason, the user wishes to define their own UPCs, this file can be replaced with one in the same format. This file should be in one of the paths indicated by the **resdir=PATH** or **buildpath=PATH** options.

This might be particularly useful in cases where the homology detection by BLAST is wrong. (Either insufficiently sensitive to weak relationships or confused by low complexity regions *etc*. By default the BLAST complexity filter is on but this can be toggled with **blastf=T/F**. BLAST sensitivity can be altered using **blaste=X** to adjust the e-value threshold. (1e-4 by default.))

2.2.4 Optional Input IV: Query Sequences

SLiMFinder can be limited to return only those motifs in a given sequence or set of sequences. This is set by **query=LIST**, where **LIST** is a comma-separated list of protein names (just the first word) or ID/accession numbers (for standard input formats). (*e.g.* *query=P04049,P30307*) This can be very powerful in larger datasets as it will increase the significance of returned motifs based on how likely they are to occur in the query sequence. (See 5.3 for details.)

This method has been further extended to allow multiple groups and definition options, using the **focus=FILE** option. This file should be in plain text format, consisting of a number of focus group entries:

```
#GroupName:Type
Entry1
…
EntryN
//
```

Each GroupName must be unique and represents a single Focus group. (The **query=LIST** option actually generates a "Query" focus group.) The Type refers to an rje_seq filter type (Seq/Spec/Desc/DB/Acc). Groups are actually made using the **goodTYPE=LIST** filter of **RJE_SEQ** to pull out the sequences of interest – see the RJE_SEQ Manual for details. Entry 1 to N then list the N entries to be added to this group. Note that each entry could pull out multiple sequences. *E.g.* to put all Human sequences from your dataset into a Group "Homo_sapiens" (assuming a properly formatted file as input, which would have "HUMAN" in each human sequence name), you could use:

```
#Homo_sapiens:Seq
HUMAN
//
```

Most commonly, Type would be "Acc" and each Entry would be an Accession Number of an input protein. Where multiple groups are given, the min. number of groups that must contain a returned motif is set with **focusocc=X**. If **X** is 0 (the default) then a motif must be present in all groups.

2.2.5 Optional Input V: Protein Alignments

To use some of the advanced SLiMFinder functions, such conservation calculations and alignment outputs, SLiMFinder must be given the location and naming convention for protein alignments. These can also be generated by SLiMFinder using GOPHER (Edwards 2006) and a sequence database. Details are given in 7.3.

2.3 Output

SLiMFinder has multiple outputs, many of which are optional. An overview of the possible outputs is given here. Unless otherwise specified, only motifs meeting the **probcut=X** threshold will be output. It is also possible to restrict output to the top X motifs, using **topranks=X**. Note that *both* these options apply to a dataset, so to return the top X motifs regardless of their probability, set probcut very high (*e.g*. *probcut=1.0*). With the exception of the main results table, all (dataset-specific) output is created in the directory specified by **resdir=PATH**.

NB. To output this file into a directory other than the run directory, use the full path in the name (*e.g.* *resfile=SLiMFinder/slimfinder.csv*).

2.3.1 Main SLiMFinder Results Table

The main output of SLiMFinder is a single delimited text file containing a list of significant motifs and dataset statistics for all datasets analysed. By default, this is a comma-separated *.csv file specified by **resfile=FILE** [default slimfinder.csv – set *resfile=None* for no output]. Name this file *.csv for comma separated output and *.tdt for tab delimited output. All other extensions will result in space-delimited output. If no extension (*.*) is found then *.csv will be appended. (*E.g*. *resfile=eg* will output to eg.csv.)

2.3.2 Sequence relationship output

Sequence relationships are defined using **BLAST** (Altschul et al. 1990) and **GABLAM** (Edwards & Davey 2006). A *.self.blast file is created during this process, containing the actual BLAST alignments. This is deleted unless **extras=T**. This file is converted into a GABLAM distance matrix, which is output into a tab-delimited distance matrix file *.dis.tdt. If **extras=T** then a PHYLIP format distance matrix is also output as *.phydis.txt, which can be used to draw trees with **PHYLIP** (Felsenstein 2005) (using **RJE_TREE** (Edwards 2006) if desired). This matrix in turn is used to define Unrelated Protein Clusters (UPCs; see 3.3.3), which are output to a relationship summary file *.upc. This file lists each cluster, the number of sequences it contains, the MST-corrected size of the UPC (see 3.3.3) and the short names of the sequences in the UPC. Note that when this file exists, it will be read for a dataset instead of regenerating with BLAST (unless **force=T**). This means that it is possible to create custom UPC groupings if desired by hacking this file (see 6.2.1).

2.3.3 SLiMDisc Output

If **slimdisc=T** then SLiMFinder will attempt to emulate **SLiMDisc** (Davey et al. 2006) output, producing *.rank and *.dat.rank files as well as a **TEIRESIAS** (Rigoutsos & Floratos 1998) format output (below).

2.3.4 TEIRESIAS Output

If **teiresias=T** then SLiMFinder will output a *.masked.fas fasta file of the *masked* sequences and a *.out file of all motifs exceeding the given **minocc=X** and **minic=X** thresholds in TEIRESIAS (Rigoutsos & Floratos 1998) output format. This is an additional output. To cancel the normal output, set **slimchance=F** and **slimdisc=F**. If **slimdisc=F** the *.masked.fas fasta file will simply be called *.fasta (to emulate SLiMDisc).

2.3.5 Extra Dataset-specific Output

If **extras=T** then a number of additional data files are created in the directory specified by **resdir=PATH** in addition to the files described above. These outputs are listed in Table 2.1 and described in more detail in Chapter 0.

Table 2.1. List of optional extra dataset-specific SLiMFinder outputs.

File	Description	Requirements
*.rank	Text file containing significant motifs and a reduced (tab-delimited) data output from the main results file	None
*.cloud.txt	Text file containing extra information on the significant motif "clouds", including the sequences containing motifs in each cloud and their overlap in terms of sequence coverage.	1+ Significant SLiMs
*.occ.csv	Comma-separated file listing the individual occurrences of these motifs (and their stats)	1+ Significant SLiMs
*.motifaln.fas	Alignment file of each significant motif across its occurrences in the context of the parent sequences	1+ Significant SLiMs. GOPHER and/or alignments for orthologues
*.maskaln.fas	Same as *.motifaln.fas except that the parent sequences are masked	
*.mapping.fas	Fasta file containing sequences for each input sequence. These sequences are present in threes for each input sequence:<br>1. The significant motifs found in that sequence, aligned to (2) and (3)<br>2. The full-length unmasked sequence<br>3. The full-length masked sequence	1+ Significant SLiMs
*.motifs	Plain text file in PRESTO-compatible format. This file can be used directly with the **slimcheck=FILE** option to search for specific motifs during SLiMFinder runs. (Note that this file also includes any motifs given by the **slimcheck=FILE** option for this run!)	1+ Significant SLiMs and/or **slimcheck=FILE** motifs
*.compare.tdt	Results from an all-by-all CompariMotif (Edwards et al. 2008) analysis of significant and **slimcheck=FILE** motifs to identify similar patterns.	1+ Significant SLiMs and/or **slimcheck=FILE** motifs
*.xgmml	This outputs a file in XGMML format that can uploaded into **Cytoscape** (Shannon et al. 2003) for visualisation. All returned SLiMs and all proteins are present as nodes. CompariMotif matches between SLiMs, UPC relationships between proteins, and occurrences of SLiMs in proteins are all marked as edges.	None. (Proteins only if no significant SLiMs.)
*cloud.xgmml	This is the same as the basic *.xgmml file, except that returned SLiMs are compressed into clouds, represented by their most significant member.	1+ Significant SLiMs

2.4 Commandline Options

Table 2.2 lists the commandline options for SLiMFinder (See also 3.1 and Figure 3.1). Please see also the PEAT Appendices document for additional general commandline options and the RJE_SEQ Manual for further input data options. The documentation (help) for rje_slimcalc.py also gives more details on options for additional SLiM statistic calculations and filtering (See Chapter 7). Beginners will probably want to leave the default settings unchanged.

Table 2.2. SLiMFinder Commandline Options.

Option	Description	Default
	Basic Input/Output Options	
**seqin=FILE**	Sequence file to search	[None]
**batch=LIST**	List of files to search, wildcards allowed. (Over-ruled by **seqin=FILE**)	[*.dat,*.fas]
**maxseq=X**	Maximum number of sequences to process	[500]
**maxupc=X**	Maximum UPC size of dataset to process	[0]
**walltime=X**	Time in hours before program will abort search and exit	[1.0]
**resfile=FILE**	If **FILE** is given, will also produce a table of results in resfile	[slimfinder.csv]
**resdir=PATH**	Redirect individual output files to specified directory	[SLiMFinder/]
**buildpath=PATH**	Alternative path to look for existing intermediate files	[SLiMFinder/]

Option	Description	Default
force=T/F	Force re-running of BLAST, UPC generation and SLiMBuild	[False]
pickup=T/F	Pick-up from aborted batch run by identifying last dataset output in resfile.	[False]
	SLiMBuild Options I: Evolutionary Filtering	
efilter=T/F	Whether to use evolutionary filtering	[True]
blastf=T/F	Use BLAST Complexity filter when determining relationships	[True]
blaste=X	BLAST e-value threshold for determining relationships	[1e^{-4}]
altdis=FILE	Alternative all by all distance matrix for relationships	[None]
gablamdis=FILE	Alternative GABLAM results file (!!!Experimental feature!!!)	[None]
homcut=X	Max number of homologues to allow (to reduce large multi-domain families)	[0]
	SLiMBuild Options II: Input Masking	
masking=T/F	Master control switch to turn off all masking if False	[True]
consmask=T/F	Mask residues based on relative conservation.	[False]
dismask=T/F	Whether to mask ordered regions (see rje_disorder for options)	[False]
ftmask=T/F	UniProt features to mask out	[EM,DOMAIN,TRANSMEM]
imask=T/F	UniProt features to inversely ("inclusively") mask (Seqs MUST have 1+ features)	[]
compmask=X,Y	Mask low complexity regions (same AA in X+ of Y consecutive aas)	[5,8]
casemask=X	Mask Upper or Lower case of input sequence (see 4.1.5)	[None]
motifmask=X	List (or file) of motifs to mask from input sequences	[]
metmask=T/F	Masks the N-terminal M (can be useful if termini=T)	[True]
posmask=LIST	Masks list of position-specific aas, where list = pos1:aas,pos2:aas	[2:A]
	SLiMBuild Options III: Basic Motif Construction	
termini=T/F	Whether to add termini characters (^ & $) to search sequences	[True]
minwild=X	Min. no. of consecutive wildcard positions to allow	[0]
maxwild=X	Max. no. of consecutive wildcard positions to allow	[2]
slimlen=X	Maximum length of SLiMs to return (no. non-wildcard positions)	[5]
minocc=X	Minimum number of unrelated occurrences for returned SLiMs. (Proportion of UP if < 1)	[0.05]
absmin=X	Used if minocc<1 to define absolute min. UP occ	[3]
alphahelix=T/F	Special i, i+3/4, i+7 motif discovery. (!!!Experimental!!!)	[False]
	SLiMBuild Options IV: Ambiguity	
preamb=T/F	Whether to search for ambiguous motifs during motif discovery	[True]
ambocc=X	Min. UP occurrence for subvariants of ambiguous motifs (minocc if 0 or > minocc)	[0.05]
absminamb=X	Used if ambocc<1 to define absolute min. UP occ	[2]
equiv=LIST	List (or file) of TEIRESIAS-style ambiguities to use	[AGS,ILMVF,FYW,FYH,KRH,DE,ST]
wildvar=T/F	Whether to allow variable length wildcards	[True]
combamb=T/F	Whether to search for combined amino acid degeneracy and variable wildcards	[False]
	SLiMBuild Options V: Advanced Motif Filtering	
musthave=LIST	Returned motifs must contain one or more of the AAs in LIST.	[]
query=X	Return only SLiMs in the Query sequence X	[None]
focus=FILE	FILE containing focal groups for SLiM return	[None]
focusocc=X	Motif must appear in X+ focus groups (0 = all)	[0]

Option	Description	Default
	SLiMChance Options	
**slimchance=T/F**	Execute main SLiMFinder probability method and outputs	[True]
**probcut=X**	Probability cut-off for returned motifs	[0.1]
**maskfreq=T/F**	Whether to mask input before any analysis, or after frequency calculations	[True]
**aafreq=FILE**	Use **FILE** to replace individual sequence AAFreqs (**FILE** can be sequences or aafreq)	[None]
**aadimerfreq=FILE**	Use empirical dimer frequencies from **FILE** (fasta or *.aadimer.tdt) (!!!Experimental!!!)	[None]
**negatives=FILE**	Multiply raw probabilities by under-representation in **FILE** (!!!Experimental!!!)	[None]
**smearfreq=T/F**	Whether to "smear" AA frequencies across UPC rather than keep separate AAFreqs	[False]
**seqocc=T/F**	Whether to upweight for multiple occurrences in same sequence (heuristic)	[False]
**probscore=X**	Score to be used for probability cut-off and ranking (Uncorrected/Sig)	[Sig]
	Output Options I: Output Data	
**clouds=X**	Identifies motif "clouds" which overlap at 2+ positions in X+ sequences (0=minocc)	[2]
**runid=X**	Run ID for resfile (allows multiple runs on same data)	[DATE:TIME]
**logmask=T/F**	Whether to log the masking of individual sequences	[True]
**slimcheck=FILE**	Motif file (PRESTO formats) or list of patterns to add to resfile output	[]
	Output Options II: Output Formats	
**teiresias=T/F**	Replace TEIRESIAS only, making *.out and *.mask.fas files	[False]
**slimdisc=T/F**	Output in SLiMDisc format instead of SLiMFinder format (*.rank & *.dat.rank)	[False]
**extras=T/F**	Whether to generate additional output files (alignments etc.)	[True]
**targz=T/F**	Whether to tar and zip dataset result files (UNIX only)	[False]
**savespace=X**	Delete "unneccessary" files following run (best used with targz):<br>- 0 = Delete no files<br>- 1 = Delete all bar *.upc and *.pickle files<br>- 2 = Delete all dataset-specific files including *.upc and *.pickle (not *.tar.gz)	[0]
	Output Options III: Additional Motif Filtering	
**topranks=X**	Will only output top X motifs meeting probcut	[0]
**minic=X**	Minimum information content for returned motifs (See 3.3.3Information content)	[2.1]
**slimcalc=LIST**	List of additional statistics to calculate for occurrences, out of Cons,SA,Hyd,Fold,IUP,Chg,Comp. See the documentation (help) for rje_slimcalc.py and 7Additional Statistics and Filtering for more details on options for additional SLiM statistic calculations and filtering.	[]
	Additional Functions	
**motifseq=LIST**	Outputs fasta files for a list of X:Y, where X is the pattern and Y is the output file	[]
**slimbuild=T/F**	Whether to build motifs with SLiMBuild. (For combination with motifseq only.)	[True]
**randomise=T/F**	Randomise UPC within batch files and output new datasets	[False]
**randir=PATH**	Output path for creation of randomised datasets	[Random/]
**randbase=X**	Base for random dataset name	[rand]

2.5 Rerunning SLiMFinder (and Pickling)

The longest part of SLiMFinder is generally the SLiMBuild portion, which assembles the actual motifs. In order to accelerate future analyses, SLiMFinder (a) outputs a *.*.pickle file following SLiMBuild if none exists, or (b) reads in an existing *.*.pickle file in place of regenerating motifs using SLiMBuild. (This uses Python's "pickle" method for saving a mid-run version of the program and all its objects.) This file could be in one of the paths indicated by the **resdir=PATH** or **buildpath=PATH** options. (If created in (a), it will always be output into the **resdir=PATH** directory.) Unless running in Windows (set **win32=T**), the pickle will be compressed with gzip.

2.5.1 Pickle naming conventions

Things obviously get complicated because of the wide array of options when building motifs. Each pickle is therefore named with the most important parameters used during SLiMBuild.

lXwXoXaX

lX : slimlen=X
wX : maxwild=X
oX : adjusted ambocc=X value
aX : a code for which ambiguity is used: 0=None, 1=equiv, 2=wildvar, 3=both, 4=both+combined

NB. It is assumed that the input dataset itself does not change! If you change the dataset but keep the same name, things may go very badly wrong! The sequences from the original run, including any masking of the data, will be re-loaded from the pickle. (If i>=0 then the option to replace the pickle with the new settings will be given.)

2.6 Secondary SLiMFinder Functions

In addition to the main SLiM discover function of SLiMFinder, a couple of additional tools are included to help analyses.

2.6.1 Simple Identification of Sequences containing Motifs

The **motifseq=LIST** option will output fasta files for a list of X:Y, where X is a motif pattern and Y is the output file. Each motif must be given a separate output file. (You can always concatenate these afterwards.)

E.g. *motifseq=P..P:sh3.fas,RGD:rgd.fas* will output all PxxP-containing sequences to a file called sh3.fas and all RGD-containing sequences to a file called rgd.fas.

By default, SLiMFinder will also run as normal. To stop this, use the **slimbuild=F** option. This is not compatible with batch running.

2.6.2 Randomising Batch Datasets

SLiMFinder incorporates a simple algorithm for randomly combining UP clusters from input datasets into a new set of fasta files with the same numbers of UPC. This is switched on with the **randomise=T** option. New datasets will be output into the directory determined by **randir=PATH** [Random/ by default] and be given a name in the form:

RAND_X_Y-Z, where RAND is the file prefix given by **randbase=X** [rand by default], X is the number of the dataset, Y is the number of sequences and Z is the number of UPC.

Note. When making these random datasets, SLiMFinder does not check whether multiple UPCs contain identical and/or related sequences. If the input datasets contain overlapping/related UPCs then it is possible that some actual random datasets will have fewer sequences and/or UPCs than listed. This can be identified in the main SLiMFinder output when the SeqNum and/or UP values do not match the dataset name.

2.7 The SLiMFinder Webserver

The SLiMFinder webserver is currently under development but can be found (soon) at **http://bioware.ucd.ie**. This gives access to the main input page (Figure 2.1). Sequences can either be pasted into the text box or files uploaded. Formatting restrictions match that of the main SLiMFinder program. Additional parameter settings can be accessed using the tabs on the input page. These match the commandline options listed above (2.4) and should be set accordingly. Each page has a "submit" button, which will proceed to the masking stage.

2.7.1 Interactive Masking

Like the SLiMDisc webserver (Davey et al. 2007), the SLiMFinder webserver has interactive masking options. If a UniProt flat file has been given as input, then key features will be parsed and presented for optional masking. In additional, IUPred (Dosztanyi *et al.* 2005) predictions are used to generate an additional "FOLD" feature if predicted globular (folded) regions (Figure 2.2(a)).

Once general features have been selected, the "Submit" button will proceed to a second round of interactive masking where each protein can be independently fine-tuned (Figure 2.2(b)). Each protein in the dataset can be expanded to give a full list of annotated features and individual domains etc. selected for masking. Furthermore, features can be manually added to proteins. (See the SLiMDisc server for more details.)

2.7.2 Job Submission

Once interactive masking is complete, the "Submit" button will submit the dataset to the SLiMFinder queue (Figure 2.3). This page will refresh automatically, or a link to the page can be downloaded for later viewing. Note that, depending on the dataset size and parameter settings, jobs may take several minutes (or occasionally hours) to complete.

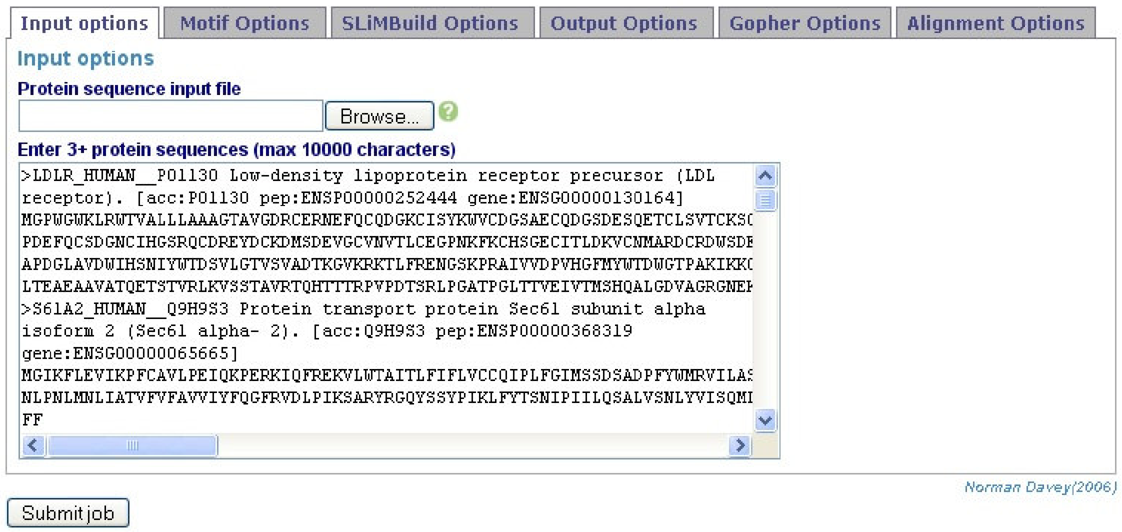

Figure 2.1. SLiMFinder webserver main page.

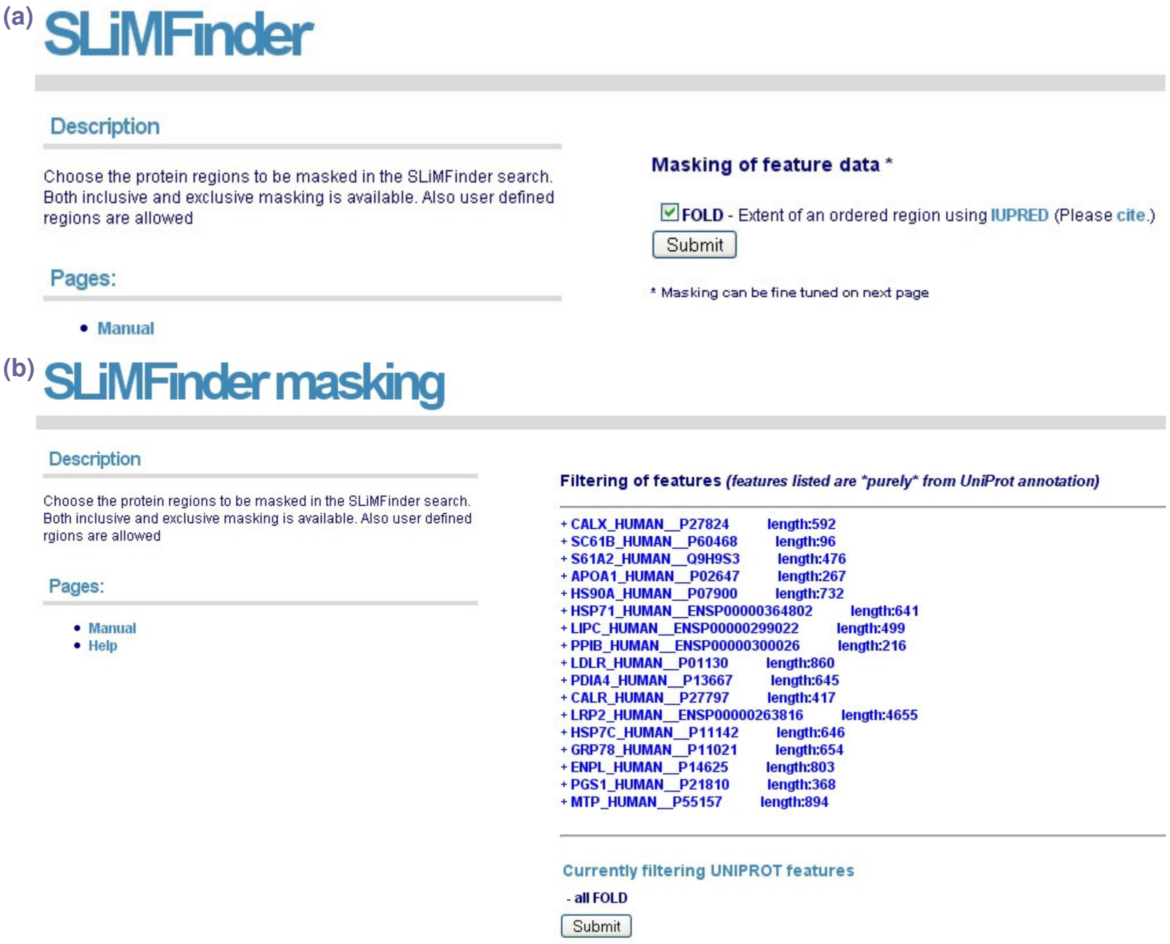

Figure 2.2. Interactive SLiMFinder input masking.
(a) UniProt features can be selected for masking for the entire dataset. **(b)** Individual features and regions for proteins can be masked for each protein.

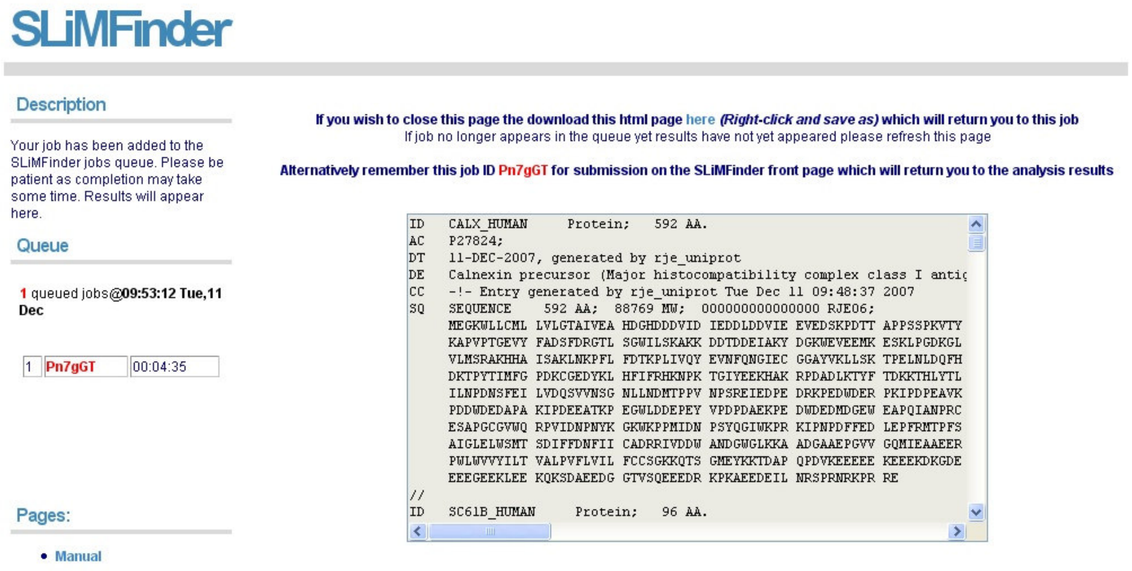

Figure 2.3. SLiMFinder webserver queue page.

(a)

Results

Rank	Sig	Pattern	Alignment	IC	Occ	Sup	UP	ExpUP	Prob	Extra data
(1)	4.05e-04	K.EL$	PDF TEXT	4.000	4	4/17	4/12	0.008	9.37e-11	+ Proteins Data |+ 2 CompariMotif Hits
(2)	0.007	KDEL$	PDF TEXT	5.000	3	3/17	3/12	5.83e-04	2.52e-11	+ Proteins Data |+ 3 CompariMotif Hits

(b)

Results for *Input.dat*

Remember this *jobId* **Pn7gGT** as it can be used to re-access results and run SLiMPickings jobs.

Results
Text|HTML
- Ranks
- Tab-delimited Ranks
- Motif Probabilities

Alignments
PDF|Text - Dataset
- Motifs Alignments

Motif Maps
Concise|Detailed
- Motifs Position Maps
- Charge Plot
- Disorder Plot
- Hydropathy

Job Details
Input|Log
- Input Dataset
- Run Details

Tarred Job Data
tar
- Local version
- All files

Figure 2.4. Main SLiMFinder webserver results page.
(a) Results table for returned motifs. The **Alignment** column contains clickable links to motif occurrence alignments, while additional information can be obtained in the **Extra data** column. **(b)** Additional output for the entire dataset is also given. This includes motif alignments and motifs marked on schematics of the input proteins.

2.7.3 Webserver results page

Once the run has finished, the main results page will appear (Figure 2.4). Returned motifs are displayed in a table, which contains links to more information (Figure 2.4(a)). The **Description** section gives an overview of the additional whole-dataset output (Figure 2.4(b)). This is subject to refinement and is suggestions for improvement are welcome. All the usual SLiMFinder output files can be downloaded as a **TAR** file.

3 SLiMFinder Methods & Definitions

This section describes the SLiMFinder methods in more detail. Please also see the SLiMFinder paper (Edwards *et al.* 2007).

3.1 SLiMFinder Program Overview

A schematic of SLiMFinder with the main options is given in Figure 3.1. The SLiMBuild and SLiMChance algorithms are explored in more detail in Chapters 4 and 5, while the output is explained in Chapter 6.

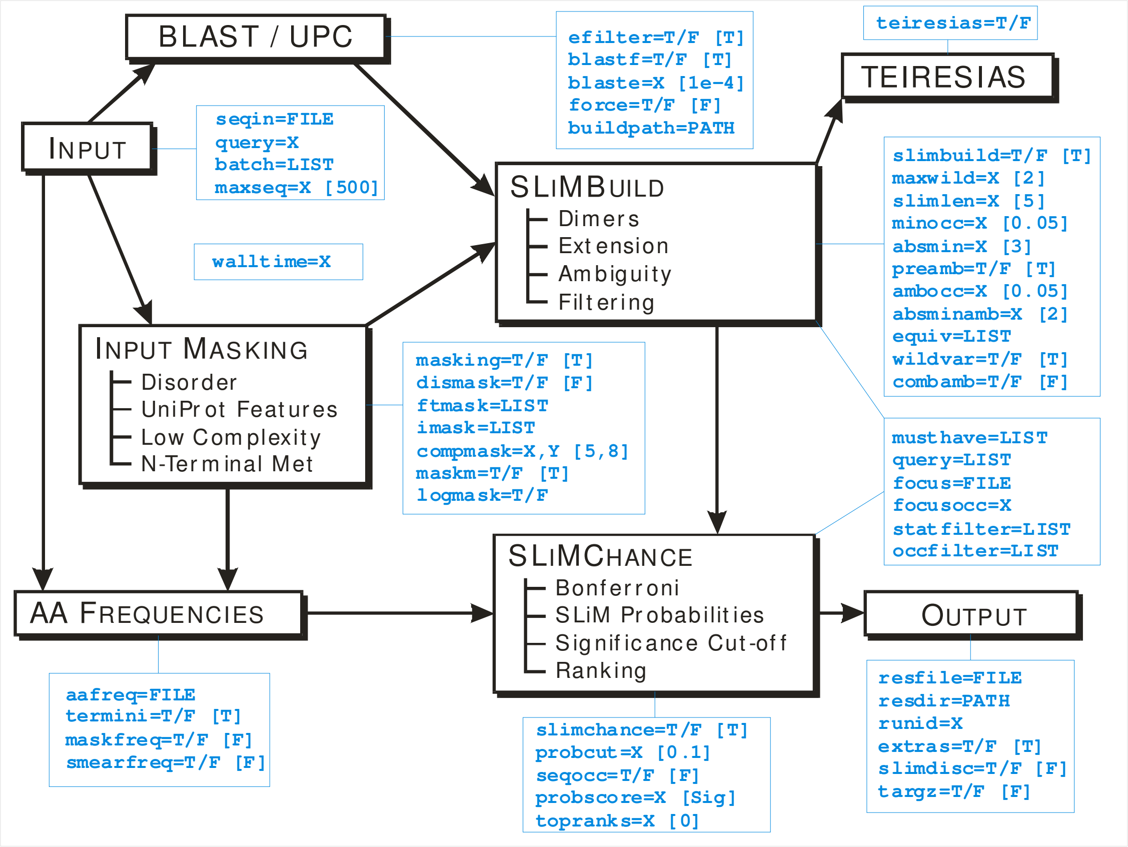

Figure 3.1. SLiMFinder Overview.
Outline of main SLiMFinder program workflow. The main commandline options are shown in blue linked to the sections they influence. See 2.4 & 7.5 for a more complete list. Details are given in the text.

The main SLiMFinder workflow is as follows:

1. SLiMFinder uses the RJE_SEQ (Edwards 2006) module to read in the input dataset. This may therefore be in any format recognised by RJE_SEQ, although UniProt or Fasta format are recommended. If no sequence file is given with the **seqin=FILE** command, then SLiMFinder will default to "batch" mode and run on all files specified with the **batch=LIST** command. By default, this equates to all UniProt (*.dat) and Fasta (*.fas) files in the run directory.

2. If present, the UPCs will be read in from a file (*.upc). Otherwise, the (unmasked) input sequences are saved as *.slimdb in fasta format and an all-by-all BLAST search performed to define Unrelated Protein Clusters (UPCs) (see 3.2). If there are insufficient UPCs for SLiMFinder to meet user parameters, it will exit.

3. If the appropriate *.pickle file present, this will be loaded. Otherwise, the input data will be masked according to user choices, masking out predicted ordered regions, selected UniProt features, low complexity regions and/or N-terminal methionines (see **4.1**).
4. If the appropriate *.pickle file was not present, the main SLiMBuild algorithm is executed. First, all iXj dimers are found using the **maxwild=X** parameter setting (4.2.1). These dimers are then reduced to those that meet the support requirement set by the **ambocc=X** option. iXj dimers are then assembled into longer SLiMs (upto the length defined by **slimlen=X**) by matching iXj and jYk dimers with the appropriate occurrences (4.2.2). Where **preamb=T**, motif ambiguity is introduced during this phase (4.2.3).
5. If **teiresias=T** then SLiMFinder will run in TEIRESIAS Mode, outputting a file of masked sequences and a TEIRESIAS-format file of shared motifs.
6. Amino acid frequencies (**5.1**) are then calculated on the dataset for each sequence and each UPC. This may use masked sequences (**maskfreq=T**) or unmasked (**maskfreq=F**) sequences, or an external source of frequency information (**aafreq=FILE**). Frequencies may be UPC-specific (**smearfreq=F**), or averaged over the whole dataset (**smearfreq=T**).
7. The SLiMChance algorithm estimates motif significance. The probability of each motif is then calculated using its support and amino acid frequencies (5.2). Probabilities are then corrected for the size of motif space searched and the **probcut=X** cut-off to identify the subset of "Significant" motifs.
8. Lastly, significant motifs are ranked and output (Chapter 6).

3.2 Unrelated Protein Clusters (UPC)

SLiMFinder is concerned in finding motif occurrences in "Unrelated Protein Clusters" (UPCs). Each UPC is a group of proteins that are not related to any proteins in the dataset outside of their own UPC (Figure 3.2). BLAST (Altschul et al. 1990) is first used to identify which proteins are related to which other proteins. Each protein is put in a cluster with all the other proteins which are hit during a BLAST search. If the BLAST results for any of these proteins include proteins not hit by the original sequence, then these too are added to the UPC. This is repeated until none of the proteins within the UPC hit, or are hit by, proteins in another UPC.

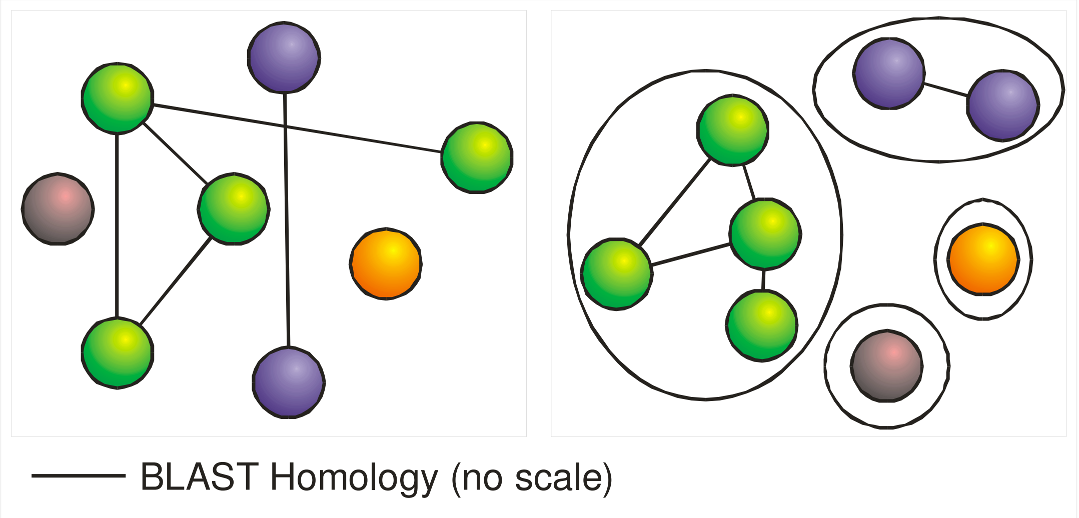

Figure 3.2. Generation of UPC from BLAST results.
An all-by-all BLAST is performed, which identifies all protein pairs with detectable homology. These are then clustered such that no protein in a cluster has BLAST homology with a protein in another cluster, while every protein in a cluster has homology with at least one other protein in its cluster.

Within each UPC a modification of Primm's algorithm (Davey et al. 2006) is used to determine the "Minimum Spanning Tree" (MST) adjusted size of that UPC in terms of sequences. This MST value varies from 1 to *N*, where *N* is the number of proteins in the UPC. If all proteins are 100% identical, the MST is 1. If all proteins were 100% different (never true for a UPC!) the MST is *N* (see SLiMDisc (Davey et al. 2006) for more details). This MST value is then converted into an MST correction *M* by dividing the MST value by *N*.

SLiMFinder uses the largest GABLAM ordered identity (Edwards & Davey 2006) to generate the distance matrix for MST calculations.

3.3 SLiM Definitions

This covers the basic definitions needed to understand this manual. The term "motif" can be used in a number of different contexts with different meanings. In this manual, I use motif to mean a short, linear motif (SLiM) in a protein. In biology, SLiMs are functional microdomains with three main properties:

Short – generally less than 10aa long with five or less defined residues.

Linear – comprised of adjacent amino acids in a protein's primary sequence. While three-dimensional conformation may be important for function, it is not necessary for definition.

Motifs – there are some defined sequence patterns that are necessary for function and will therefore recur in the relevant proteins, allowing identification.

In this manual, "SLiM" may describe a true functional motif with these properties, or simply a SLiM-like sequence pattern that may be functional or may simply be a chance occurrence.

3.3.1 SLiM Anatomy

The basic anatomy of a SLiM is shown in Figure 3.3.

The number of positions for a SLiM, *L* is the number of defined (*i.e.* non-wildcard) positions (upto `slimlen=X`). The total length of a SLiM is the number of defined (*i.e.* non-wildcard) positions plus the number of wildcard positions:

$L + \sum_{w=1}^{L-1} X_w$, where X_w is the number of wildcard positions at position *w*.

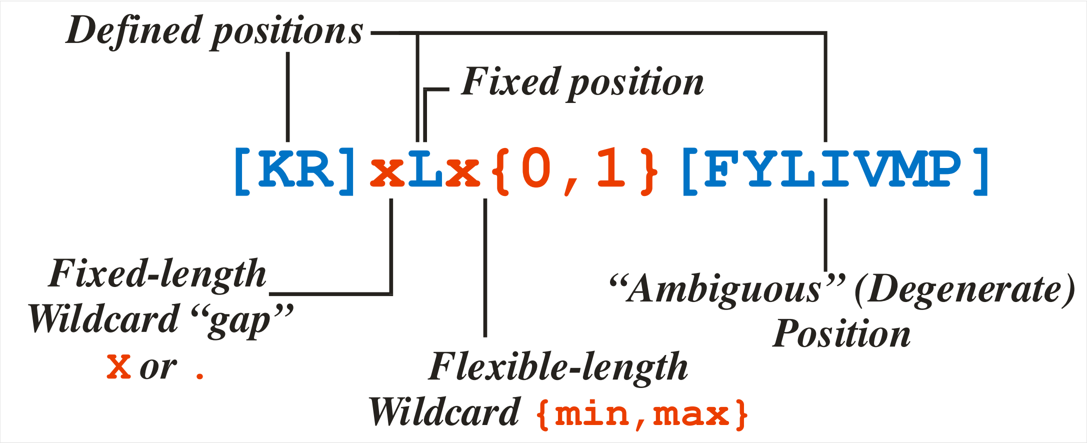

Figure 3.3. Anatomy of a SLiM.
Definitions of different properties of SLiM have been marked on the example ELM, LIG_CYCLIN_1 (Puntervoll et al. 2003). This motif has three defined positions (one fixed and two degenerate) and two wildcard spacers (one fixed, one flexible-length) for a total length of 4-5aa.

3.3.2 SLiMs and SLiMBuild Dimers

SLiMs are defined in terms of the number of non-wildcard positions and the number of wildcards between each pair of positions. Each SLiM is therefore made up of a number of overlapping dimers *i-X-j* where *i* and *j* are non-wildcard positions and *X* is the number of wildcard characters separating them. *E.g.* the well-known RGD motif is considered by SLiMFinder to be R-0-G-0-D, which in turn is comprised of the SLiMBuild Dimers R-0-G and G-0-D.

3.3.3 Information Content (IC)

Information content is calculated for each motif based on a uniform distribution of amino acids and re-scaled to give a value of 1.0 per fixed position and 0.0 for a wildcard. Ambiguous positions are given a value between 0.0 and 1.0:

$$IC_i = -\log_N(f_a)$$

where IC_i is the information content for position *i*, f_a is the summed frequency for the amino acids (or nucleotides) at position *i* and *N* is number of amino acids (or nucleotides) in the alphabet, *i.e.* N=4 for DNA and N=20 for proteins. This is a modification of Shannon's Information Content (Shannon 1997) such that a wildcard receives 0.0 and a fixed position scores 1.0 when a uniform frequency distribution is used. Ambiguous positions score between 0.0 and 1.0. When non-uniform frequencies are used, fixed rare amino acids ($f_a < 1/N$) will score above 1.0, while fixed common amino acids ($f_a > 1/N$) will score less than 1.0. Termini always get an IC_i score of 1.0. For each comparison, the lower IC_i value is where IC_i is the information content for position *i* and f_a is the number of possible amino acids at position *i*. The information content for the motif is simply this score summed over all positions.

3.3.4 Motif Occurrences and Support

An occurrence of a motif in SLiMFinder is a single “instance” of that motif in a given protein and is identified using the sequence identifier and starting position of the motif. Motifs may have multiple occurrences with a UPC or even within a single sequence.

The support of a motif in SLiMFinder is defined as the number of sequences that motif occurs in. Note that support in this context does not make any distinction between motifs with multiple occurrences in the same UPC. For this reason, SLiMFinder will output the number of occurrences, the support, and the normalised UP support, which is the number of *unrelated proteins* (UPCs) in which the motif is found. The uncorrected support is of interest for identifying the dataset coverage for each motif, while the corrected support is important for determining its significance (see Chapter 5).

4 SLiMBuild Motif Construction

4.1 Input Masking

SLiMFinder masks input by replacing regions and residues with Xs. Note that SLiMFinder masking is performed after UPC definition and therefore masking will not affect the UP relationships between sequences. If you *want* to affect UP definition, then sequences must be masked manually *before* running SLiMFinder. SLiMFinder includes several masking options (below). In addition, the rje_seq module provides additional input data filters (see RJE_SEQ Manual for details). If UniProt files are not available, the unifake utility can be useful in generating files to maximise the potential of masking (See website).

4.1.1 Disorder masking

SLiMs tend to occur in disordered regions of proteins. SLiMFinder makes use of **IUPRED** (Dosztanyi et al. 2005) and/or **FoldIndex** (Prilusky et al. 2005) to predict regions of disorder. IUPRED must be installed locally, while FoldIndex can be run over the web. Residues predicted to be "intrinsically ordered" are masked out. The IUPRED -off threshold can be altered using **`iucut=X`** (0.2 by default). To use FoldIndex instead of IUPRED, use the **`disorder=foldindex`** command. You must have an active internet connection.

Because disorder masking utilises a per-residue score, there are often single residues that are just above/below the threshold in a region that is otherwise (dis)ordered. Regions can therefore be smoothed out using the **`minregion=X`** option, which stipulates the minimum number of consecutive residues that must have the same disorder state. (Dis)ordered regions smaller than this are assimilated into the neighbouring regions, starting with the smallest (1aa regions) and working up until all regions are large enough; within each region size, the sequence is traversed from N-terminal to C-terminal.

4.1.2 UniProt Features

If the input dataset is in UniProt format then features can be masked out. Any feature types given by the **`imask=LIST`** feature will be "inclusively masked", *i.e.* any sequence not part of one of these features will be masked out. Feature types given by **`ftmask=LIST`** (EM, DOMAIN, and TRANSMEM by default) will then be masked out.

4.1.3 Low Complexity Masking

SLiMFinder uses a simple complexity filter. If any amino acid occur N+ times in a stretch of L amino acids (**`compmask=N,L`**) then the central (N-2) occurrences of that amino acid are replaced with Xs. *E.g.* PFPPIPLP would become PFXXIXLP.

4.1.4 N-terminal Methionines

If using terminal motif searching (**`termini=T`**) then there is a high risk of artefactually returned `^M*` motifs due to the high occurrence of Met at position 1. The **`maskm=T`** option masks any position 1 Ms to remove this artefact. (Of course, real `^M*` motifs may be missed as a result.)

4.1.5 Masking by Case

SLiMFinder can also mask out Upper or Lower case sequences as set by the **`usecase=T`** and **`casemask=X`** option, where **`X`** is *`Upper`* or *`Lower`*. The case of the sequences can be changed by the additional option **`case=LIST`**, where **`LIST`** is the positions to switch case, starting with first lower case (*e.g.* *`case=20,-20`* will have twenty amino acids of Upper case at each end of each sequence).

Example. To search only in the C-terminal 30aa of each sequence, use:

```
python slimfinder.py casemask=Lower usecase=T case=0,-30
```

NB. In this case, it is recommended to change the way amino acid frequencies are used by adding the options *`maskfreq=T smearfreq=T`* (see 5.1). This will use the amino acid frequencies from just the

unmasked portions of the sequences. Because these are quite short, they are prone to random fluctuations, so the smearfreq option will average the frequencies over the sequences.

4.1.6 Relative Conservation Masking

Sites of functional importance are likely to exhibit as much, or more, conservation than those around them. SLiMFinder incorporates a masking strategy that removes residues that violate this assumption by masking out any positions that are less conserved than flanking regions as assessed using a score based on Shannon's entropy. A conservation score is first calculated for each position, *i*, of each sequence using the optional multiple sequence alignment (MSA) input of orthologues (see 2.2.5 and 7.3):

$$c_i = (1 - \sum f_a \log_{20}(f_a)).g_i$$

Where f_a is the frequency of each (non-X) amino acid *a* in the MSA column *i* and g_i is the proportion of sequences in *i* that are not gaps. This will be 1.0 for a fixed (100% conserved) residue and tend towards 0.0 for a totally variable residue in a large alignment (i.e. all 20 aa have a frequency of 1/20). The "gap penalty" reduces the score for columns with indels and includes Xs as non-gapped residues, even though these are not included in the entropy calculation. If all the non-gap sequences in a column are Xs, the amino acid frequencies from the whole alignment are used to calculate the entropy for that position.

This is then converted into a relative conservation score, r_i, which is based on the mean conservation score across a window flanking the residue, normalised by the standard deviation:

$$r_i = \frac{c_i - c_w}{SD_w}$$

Where c_w and SD_w are the mean and standard deviation respectively of the c_i scores across a window of 30aa either side of position *i*. The resultant score, r_i will be positive for residues that are more conserved than their flanking residues and negative for those less conserved (mean 0.0, standard deviation 1.0). Any residues that have a different disorder state from residue *i* are excluded from the calculation. (Ordered regions generally show a higher level of conservation than disordered regions.)

Any residues with $r_i < 0.0$ are masked out. (If there is no MSA for a protein, all residues will have a score of 0.0 and no residues will be masked.)

4.1.7 Masking pre-defined motifs

Certain commonly recurring motifs (e.g. [KR][KR] or RSRS) can dominate results from large-scale analyses. These motifs can now be masked from the input dataset using **`motifmask=X`**, where **`X`** is a file containing motifs or simply a list of motifs (see 2.2.2). Wildcard positions in such motif occurrences will *not* be masked out. (e.g. PxxP would only mask the two prolines.)

4.2 SLiM Construction

SLiMBuild uses four basic sets of parameters for generating motifs from the dataset:

w, the maximum number of wildcard positions allowed between any adjacent pair of defined positions.

The maximum number of defined positions. (Sometimes referred to as the "length" of the motif, although the "true length" of a SLiM would include both defined and wildcard positions.)

s, the minimum support for the motif, i.e. the number of unrelated proteins that motif occurs in.

Ambiguity options, including an equivalency file of allowed ambiguities.

An optional minimum variant support, *v*, used in extending ambiguity.

Motifs are constructed by first identifying all possible "*i*-*x*-*j* dimers", which consist of two amino acids *i* and *j* separated by *x* wildcards, up to the maximum allowed value, *w* (Figure 4.1(a)). Motifs are then extended by joining appropriate dimers together (Figure 4.1(b)). Finally, SLiMBuild incorporates ambiguity into the motifs (Figure 4.2).

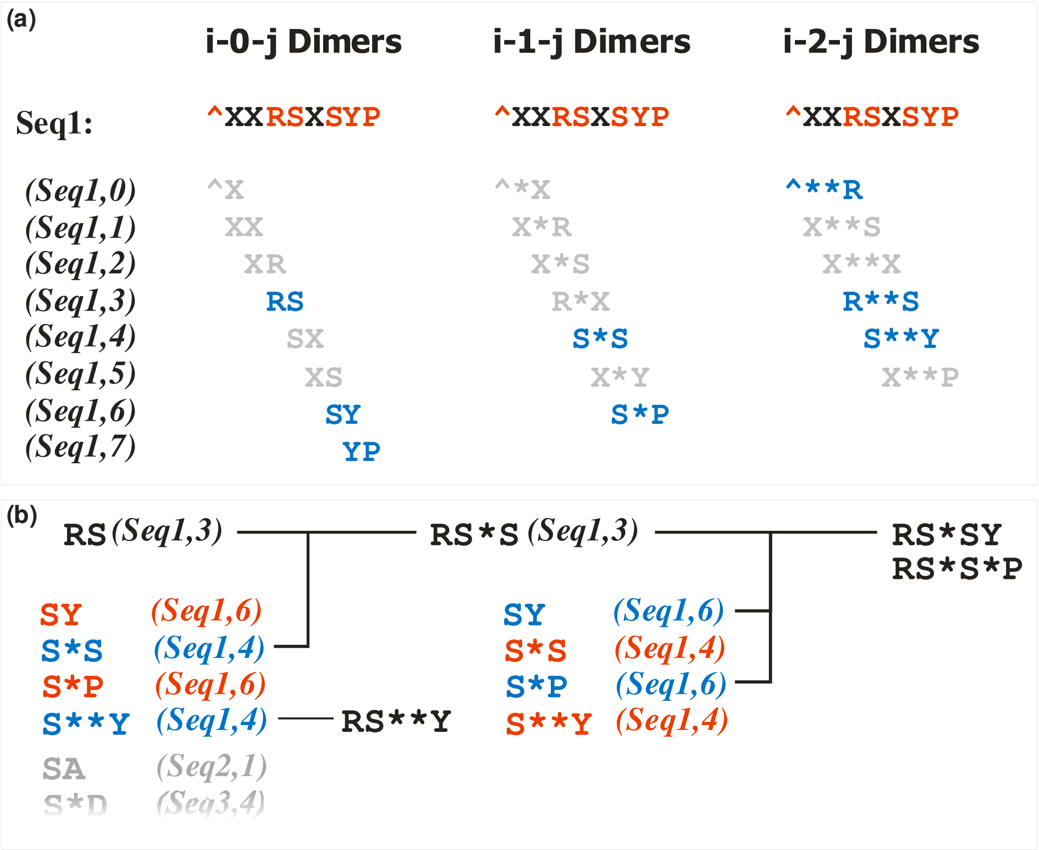

Figure 4.1. SLiMBuild construction of motifs.
(a) Dimer construction. For each position in a sequence, each possible wildcard length x is used to find possible "i-x-j" dimers. Dimers containing masked ("X") residues are ignored (greyed dimers). Note that the n-terminal "^" marker is treated as any other amino acid. **(b)** Motif extension. Longer SLiMs are constructed during the SLiMBuild process by matching the occurrences of shorter SLiMs with the relevant i-x-j dimers. At each stage, only SLiMs with sufficient unrelated protein support are retained, making the algorithm very efficient.

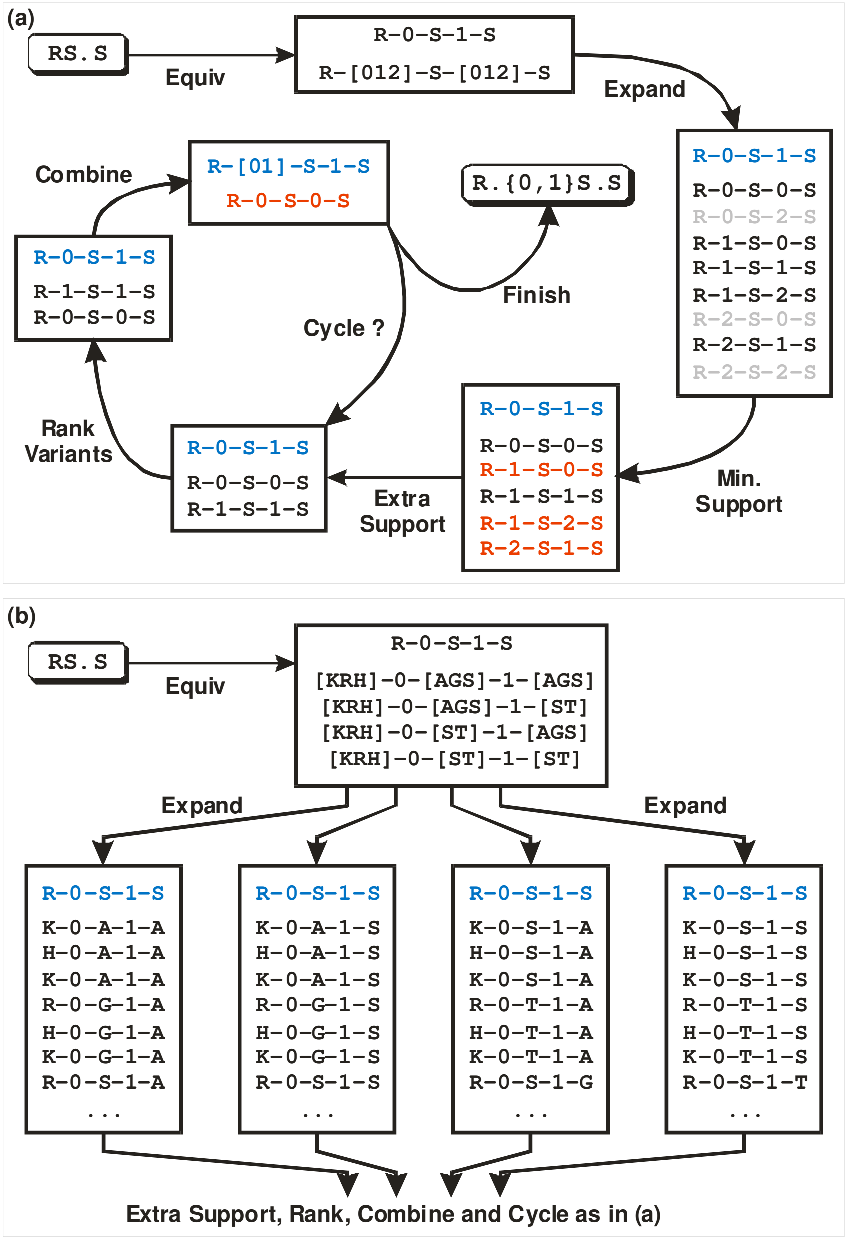

Figure 4.2. SLiMBuild Ambiguity.
(a) Wildcard ambiguity. Ambiguity is added in a multi-stage process. First, the motif is broken up into its component parts, consisting of alternate defined and wildcard positions. These are then replaced by the appropriate equivalency group, which in the case of wildcards is the full range of wildcard lengths from 0 upto the maximum length allowed. These equivalencies are then expanded to all possible variants. Variants with insufficient support (grey) are ignored and any variants (red) not increasing the UPC support of the motif are dropped. The remaining variants are ranked (see text) and the best variant combined with the original motif (blue). The remaining variants are re-assessed for increasing UPC support and any failing to do so are again removed. If any remain, the ranking and combining cycle repeats. If not, the finished degenerate motif is returned. **(b)** Amino acid ambiguities. These are handled in the same way as wildcard ambiguities, except that this time equivalencies are defined by the given equivalency list. If a given amino acid belongs to multiple equivalency groups, such as serine ([AGS] and [ST]) then all possible combinations of these equivalency groups (four in this case) are considered separately, thus multiple ambiguous SLiMs can potentially be produced. (Expansion of these combinations has been truncated in the figure.)

4.2.1 SLiMBuild dimer construction

Dimers are constructed simply by taking each position i of each protein in turn to define the first amino acid, a_i. Each wildcard length x from 0 to W, where W is the maximum wildcard length is then taken in turn and used to define the second amino acid in the dimer, a_j where $j = i + x$. If a_i or a_j are masked (an 'X') then that dimer is rejected, else the dimer is added to the stored list, along with information on the protein and position i of its occurrence (Figure 4.1(a)). Symbols representing N- and C-termini (^ and $) are added to each sequence prior to dimer construction and thereon considered as additional amino acids.

After all dimers have been found in all sequences, any with a support below the minimum support threshold are removed. (For a motif to exceed a given support, each of its component dimers must also exceed that support.) This greatly increases the efficiency of the algorithm. The efficiency is further increased by establishing the evolutionary relationships first, thus screening out all those motifs that only occur multiple times because of shared ancestry.

4.2.2 SLiMBuild motif extension

Motifs are extended by concatenating i-x-j dimers (Figure 4.1(b)). For each dimer $a_ix_1a_j$ all $a_zx_2a_k$ dimers are examined, where $a_z = a_j$ ($k = z + x_2$, a_z and a_k are amino acids at positions z and k). Where the two dimers have occurrences in the same protein and $z = j$, the two dimers are compiled to make a single $a_ix_1a_jx_2a_k$ trimer. If this trimer occurs in s or more unrelated sequences, it is retained and extended in the same way to make 4mers. This continues until the maximum motif length is reached (length 5 by default) or until there are no more motifs with the desired support to extend.

4.2.3 SLiMBuild ambiguity

SLiMBuild considers two types of ambiguity: amino acid degeneracy at a given position, and flexible length wildcard "gaps". A similar logic is applied in considering both these forms of ambiguity by carefully combining appropriate motifs generated during SLiMBuild extension. Each fixed motif is considered in turn as a seed for adding ambiguity in terms of degenerate non-wildcard positions and/or flexible wildcard lengths (Figure 4.2). Ambiguity is considered in three phases: wildcards only, amino acids only and combined wildcard and amino acid degeneracy. (Combined ambiguity can be computationally intensive and is switched off by default.)

In each case, the motif being considered is broken down into individual elements, consisting of alternate amino acids and/or wildcard lengths. Each element is then replaced by its "equivalencies". For wildcards, this consists of single wildcard equivalency "01..*W*", where W is the maximum wildcard length allowed; *e.g.* for the default maximum wildcard length of 2, the wildcard equivalencies are 0, 1 and 2, and a variable length gap of 1 or 2 is represented by the equivalency [12]. (Figure 3A). For amino acid positions, SLiMFinder makes use of an "Equivalency list" for ambiguity in a similar way to TEIRESIAS, although the actual application of this file is quite different. This equivalency list contains a number of amino acid groups that may be substituted in degenerate positions; *e.g.* KR would allow for [KR] degeneracy, while FYW, would facilitate [FY], [YW], [FW] and [FYW]. A single amino acid can have multiple equivalency groups, which are analysed separately. *E.g.* AGS,ST would permit serine [AS], [GS], [AGS] and [ST], but <u>not</u> [AGST]. Where multiple equivalency groups exist for one or more amino acids in a SLiM, all possible combinations of equivalency group are considered (Figure 4.2(b)).

The idea of ambiguity is to try to increase the coverage within a dataset for a given motif. This is achieved by adding ambiguity that increases support (no of unrelated proteins) for the motif. Thus, returned motifs need to have been initially seeded by a non-ambiguous motif (with lower support) before it is extended to consider ambiguity. For each ambiguity combination, all possible variants (excluding the original motif) are then considered. *E.g.* [KR]-0-[ST]-1-P yields variant motifs K0S1P, R0S1P, K0T1P and R0T1P, the second of which is ignored as it is the original motif. Any variants that do not meet the minimum support requirement are also rejected. Remaining variants are then ranked according to the following criteria:

1. Number of "new" UP clusters. (The number of UPCs in which the variant is found but the original motif is not.) If the variant provides no new UPCs then it is rejected.
2. Total (UPC) support for the variant, if tied for 1.

3. Total number of occurrences for the variant (in different sequences, regardless of homology relationships), if tied for 1 & 2.

If tied for 1-3, the variant that is most unlikely, given the amino acid frequencies of the whole dataset, is ranked higher.

The top-ranked variant is retained and its UPCs added to those of the original motif. The ranking is then repeated using this new UP support, *i.e.* further variants are not added if their "extra" support has already been provided by previous variants. This continues until all variants have been retained, or rejected (Figure 4.2). Finally, retained variants are combined to make an ambiguous motif. *E.g.* if R0T1P had been retained then it would be combined with the original R0S1P SLiM to make R0[ST]1P (R[ST].P). In the case of flexible wildcards, the minimum and maximum length variants retained are used. *i.e.* R0S1P+R2S1P = R[02]S1P (R.{0,2}S.P). Note that because different equivalency combinations are examined separately, one SLiM may spawn several ambiguous motifs (*e.g.* R [ST].P and R [AGS].P) but only one ambiguity will be produced per equivalency group (*i.e.* R[AS].P and R[AGS].P will not both be produced using a single AGS equivalency group).

To increase the flexibility of ambiguity, SLiMFinder has an option for reducing the minimum support needed for each variant to be retained by SLiMBuild. The final ambiguous motif must, however, meet the basic support criteria. *i.e.* The user could specify a minimum basic support *s* of three but a minimum variant support *v* of two. Ambiguous motifs could therefore be constructed from variants that each occurred in only two unrelated proteins, though the final motif produced (as well as any fixed motifs being kept) would occur in at least three unrelated proteins.

4.2.4 Final SLiM Support

After ambiguity has been added (if any), SLiMs are filtered to only retain those with enough support to satisfy the `minocc=X` setting. If the `query=X` option is used, only those motifs in the query sequence are retained.

4.3 SLiMBuild versus TEIRESIAS

Ignoring the extra utilities of the SLiMChance scoring metrics and additional outputs, the SLiMBuild approach has a number of advantages for SLiM discovery over TEIRESIAS (Rigoutsos & Floratos 1998) and would therefore make a worthwhile replacement of TEIRESIAS for other SLiM discovery methods, such as LMD (Neduva et al. 2005) or SLiMDisc (Davey et al. 2006). These are explored in Table 4.1.

Table 4.1. Major advantages of SLiMFinder over TEIRESIAS for SLiM Discovery.

SLiMFinder	TEIRESIAS
Explicitly looks for shared motifs in unrelated proteins: the more relationships in a dataset, the less motifs returned (and the shorter the run time).	Looks for all shared motifs: the more relationships in the dataset, the more patterns returned (and the *longer* the run time).
Support is in terms of unrelated proteins – reduced post-filtering.	Support is in terms of any occurrences – many irrelevant motifs returned that must be filtered.
Building method allows good estimation of "motif space" searched, permitting strong statistical treatment of results.	Search space covered by parameter settings is not clear, hampering statistical treatment of results. (Crude restrictions will miss some motifs of interest and/or return too many (related) motifs.)
Ambiguity is added only when it will increase the number of unrelated proteins for a motif.	It is not very clear which ambiguous motifs will be returned.
Flexible-length wildcards are allowed.	No flexible-length wildcards.
Advanced filtering and masking options allow the search-space to be reduced.	No inherent filtering/masking options. Must all be post-processed.
A probabilistic method (based on binomial theory) calculates expectations and estimates significance of results.	Results are returned in a jumble and without any scoring metric.

5 SLiMChance Motif Probability Methods

5.1 Amino Acid Frequencies

By default, amino acid frequencies are calculated from the dataset, individually for each sequence. These frequencies can be over-ridden using the `aafreq=FILE` command, where FILE is either a fasta file (this can be the input file to even aa frequencies across sequences) or an aa frequency file (see PRESTO for details). If `maskfreq=T` then the AA frequencies used will not include any masked residues. This can lead to amino acid bias problems if the unmasked sequences are quite short, which may in turn lead to artefactually poor significance values for returned motifs. This in turn can be rectified using the `smearfreq=T` option, which will calculate AA frequencies for each UPC and then use mean frequencies across all UPCs for the SLiMChance calculations. Suggested use of these options is listed in Table 5.1.

The default uses amino acid frequencies for the whole dataset before masking. This can return significant values as artefacts if the masking itself has a compositional bias. One way to test for this is to run SLiMFinder with both settings and compare the results. In each case, only known one-letter amino acids (*i.e.* no masked residues) contribute to the total.

Table 5.1. Suggested use of Amino Acid Frequency options.

Scenario	Suggested settings
Dataset consists of full-length proteins, without masking.	`maskfreq=F smearfreq=F`
Dataset is masked but unmasked regions are still long.	`maskfreq=T smearfreq=F`
Dataset is masked and unmasked regions may be short. Masking is independent of AA composition.	`maskfreq=F smearfreq=T`
Dataset is masked and unmasked regions may be short. Masking itself may have a compositional bias, such as masking ordered regions or UniProt domain features.	`maskfreq=T smearfreq=T`
Dataset consists of a compositionally biased subset of the "true search space", such as a set of phage display peptides.	`aafreq=FILE`

5.2 SLiMChance motif probability estimation

SLiMBuild returns the list of motifs that occur in the desired number of unrelated sequences. However, because only a few amino acids define each motif, there is a reasonably high chance of a motif recurring in unrelated sequences purely by chance. This is further complicated by the fact that a large number motifs are being considered for any given dataset and each of these motifs has a chance of recurring. The SLiMChance algorithm therefore consists of two layers (described in detail below):

SLiMChance calculates the absolute probability for each motif returned by SLiMBuild. If a motif occurs in *k* unrelated proteins, the absolute probability is the probability of exactly that motif occurring in *k*+ UPCs in the dataset. This calculation is based on the amino acid composition of the dataset.

SLiMChance adjusts the absolute probabilities in an attempt to account for the fact that multiple motifs are being considered and these motifs are only being considered (*i.e.* only being returned by SLiMBuild) because they occur a certain number of times in the dataset. This calculates an estimate of how many motifs with a given absolute probability or smaller should be returned by the dataset, or the probability of seeing that motif or another one like it. This is based on the Bonferroni correction for multiple testing.

5.2.1 SLiMChance probabilities per UPC

SLiMChance first calculates the probability of seeing each motif in each UPC, given its amino acid composition and *i-x-j* dimer frequencies. This probability is calculated using the binomial distribution and the expectation of the motif occurring at each site in the UPC, which is a simple calculation based on the frequency of each amino acid (f_a), and the total number of positions that a motif can occur (N_m). By default, amino acid frequencies are calculated from the dataset, individually for each UPC, before any masking takes place. Additional options allow amino acid frequencies to be adjusted for masking, averaged over all UPCs, or read from a file.

For each defined position in a motif with d alternative (degenerate) amino acids, the probability of occurrence at any residue in the dataset (p_i) is the sum of the frequencies for the possible amino acids at that position:

$$p_i = \sum_{a=1}^{d} f_a$$

The probability p_m of the whole motif starting at any residue is therefore the product of p_i over all L positions in a motif:

$$p_m = \prod_{i=1}^{L} p_i$$

(Wildcard positions do not contribute to this value, as the probability of matching a wildcard is 1.0.).

This defines the probability for each “Bernoulli trial” in the binomial distribution. What remains is to define appropriately the number of trials for the motif in the UPC. There are two features of the UPC that complicate estimation (for the probability calculation) of the number of positions that a motif might arise at: firstly, some but not all regions of the UPC proteins are related by evolution, and secondly, the particular pattern of masking may alter the number of positions available for motifs with a particular distribution of non-wildcard positions.

Because the proteins within a UPC are evolutionarily related, they do not contribute to the motif space searched by SLiMFinder in the same way as unrelated proteins, for which the motifs found would be independent. However, unless all the sequences are 100% identical, there are still more independent positions at which a given motif could occur than in any of the individual sequences within the UPC. The UPC must therefore be rescaled to represent its true contribution to the dataset. This is performed using the “Minimum Spanning Tree” (MST) correction used by SLiMDisc (Davey *et al.* 2006) to correct for evolutionary relationships. This MST value varies from 1 to N, where N is the number of proteins in the UPC. If all proteins are 100% identical the MST value is equal to 1 (and the UPC is exactly equivalent to a single sequence). As the proteins become more dissimilar, MST tends towards N (see SLiMDisc (Davey *et al.* 2006) for more details). This is converted into an “MST correction”, M, for the UPC by dividing the MST value by N. The total size of the UPC is therefore adjusted by multiplying N_{aa} (the total number of unmasked residues in the UPC) by M. (This is equivalent to the mean number of amino acids per sequence in the UPC, multiplied by the MST-corrected size of the UPC.) SLiMFinder uses the largest GABLAM (Davey *et al.* 2006) ordered percentage identity between each pair of sequences to generate the distance matrix for MST calculations.

The distribution of masking may influence the potential for a particular type of motif (e.g. L..LY..L) to occur. For a dimer motif with a given wildcard length x, SLiMChance directly observes the frequency of positions in the dataset that could accommodate a dimer motif of that wildcard length. Then, for longer motifs, it estimates the frequency of potential sites as the product of the fraction of dimer sites for all the dimers that constitute the motif. This has the numerical advantage that the frequencies of dimer types are previously available from the SLiMBuild computation. The number of trials is then estimated as the possible number of positions at which the motif could start (N_m). N_m is calculated empirically from the dataset. During dimer generation, (D_x) the fraction of unmasked residues that start with a dimer of that particular wildcard-length x is calculated as a proportion of non-masked positions in the UPC.

$$D_x = \frac{N_{ixj}}{N_{aa}}$$

Where N_{ixj} is the count of such dimers in the UPC for which neither *i* nor *j* are masked, and N_{aa} is the total number of unmasked residues in the UPC.

N_m, the number of positions at which a motif may potentially occur is then calculated from the product of the motif's component dimer frequencies and the MST-adjusted number of unmasked residues in the UPC:

$$N_m = N_{aa} M \prod_{w=1}^{L-1} D_{xw}$$

where M is the MST correction for that UPC, L is the length (no. of positions) of the motif and D_{xw} is the dimer frequency for that wildcard length x at wildcard position w. (For flexible-length wildcards, this is the mean dimer frequency of the length variants at w.)

If there are wildcard length variants, each length variant has a chance of occurring and so this effectively increases the number of possible motif positions via a simple multiplication, where x_j is number of wildcard variants at wildcard position j:

$$N_m = N_{aa} M \prod_{w=1}^{L-1} D_{xw} \prod_{j=1}^{L-1} x_j$$

It could be argued that this multiplier should apply to the probability of the motif at each position, rather than the number of motif positions. (In reality, each motif "position" is a starting residue. Obviously, there cannot be more starting residues than the length of the sequence, whereas this multiplication implies that there can be.) The reason for applying the correction to N_m, however, is that this value has no upper bound for the binomial calculation. The probability p_m, in contrast, must be ≤ 1.0, whereas the multiplier for numerous variable-length wildcards could cause it to exceed 1.0.

The probability of 1+ occurrences of the motif in the UPC is calculated using the binomial:

$$p_{1+} = 1 - (1 - p_m)^{N_m}$$

5.2.2 SLiMChance probabilities per dataset

The individual p_{1+} values are then used to calculate the motif probability for the entire dataset, p, where N_U is the number of UPCs in the dataset and K_U is the number of UPC containing the motif. Again, this is calculated using the binomial, where p_u is the mean p_{1+} value for each UPC:

$$p_u = \frac{\sum_{u=1}^{N_U} p_{1+}}{N_U}$$

$$p = 1 - \sum_{k<K_U} \frac{N_U!}{k!(N_U - k)!} . p_u^{\,k} . (1 - p_u)^{N_U k}$$

In addition, the expected number of UP occurrences is calculated, which is simply the sum of the p_{1+} values.

5.2.3 SLiMChance significance values

The probability calculated above is the estimated probability of seeing a given motif with its observed support (or greater) given the dataset. However, the calculations implicitly assume that the motif was defined before anything was known about the dataset. In reality, SLiMFinder is looking for all possible motifs and only actually returning those at the "top end of the distribution", *i.e.* the over-represented motifs. In reality, each motif in the "motif space" searched has a chance of being stochastically over-represented, so it is important to adjust for this and establish a significance value for each motif.

The *a priori* probability of each motif in motif space being over-represented with a probability p is itself (perhaps obviously) p. Because of the wall that SLiMBuild generates motifs using a maximum wildcard spacer length, X, it is possible to calculate exactly the size of the motif space, B_L, for each length of motif L:

$$B_L = 20^L (X+1)^{L-1}$$

The significance of a motif with occurrence probability p can therefore be calculated using the binomial distribution as the probability of getting one or more successes given B_L trials of probability p.

$$Sig = 1-(1-p)^{B_L}$$

This significance ranges from zero to one can be thought of as a true p-value. Because different lengths of motifs are not independent of each other, this significance value is calculated independently for each number of defined positions. The motif space calculation only calculates the number of fixed-position motifs in the search space. Allowing ambiguities obviously increases the size of the search space and very relaxed ambiguous searches may need to use a more stringent p-value accordingly.

5.3 Increasing significance with restricted searches

The SLiMChance calculations assume that nothing is known about the motif *a priori*. Sometimes, however, additional information may be used to restrict the search space and thus increase the significance of returned motifs. The most common ways to do this are to specify query sequences, that must contain the motif, or to specify particular amino acids that the motif must contain. These are described below.

5.3.1 Query sequences and focus groups

Sometimes, you are only interested in motifs that occur in a particular sequence, or group of sequences. These can be given using the **`query=LIST`** option. In this case, only motifs that occur in at least one query sequence will be returned. Sometimes, this will increase the significance of the returned motifs. This significance adjustment is performed using, once again, the binomial distribution, where:

- the number of trials n is the observed UP support of the motif
- the probability of each trial p is the proportion of UPC that contain one of the query sequences
- the number of successes k is 1 (the number of UPC that the motif must occur)

The SLiMChance significance is multiplied by this probability. In small datasets, this will probably have little impact as significant motifs will tend to occur in all UPC, and the probability of seeing them in the query UPC is therefore 1.0. (If they occur in all UPC, they must occur in the query.) For larger datasets, the impact can be quite strong. If a motif occurs in 10 of 50 UPC, for example, the probability of it occurring in your query sequence is approx 18%, so the significance of the motif will be increased roughly five times.

This can be extended further using the **`focus=FILE`** command (see 2.2.4). This allows you to set multiple query groups and specify how many of them must have the motif using **`focusocc=X`**. In this case the binomial is still used but the number of successes is determined by the **`focusocc`** setting.

5.3.2 MustHave amino acid restrictions

Sometimes you want to limit motifs returned to contain a given amino acid, *e.g.* tyrosine if looking for tyrosine phosphorylation motifs. This can be done quite simply by using the **`musthave=LIST`** command, where **`LIST`** a list of amino acids. Returned motifs must contain at least one of these amino acids. This increases the significance of motifs by reducing the motif space.

If a is the number of amino acids in the MustHave list, the proportion of motifs that contain 1+ MustHave amino acids, H, for a motif of length L, is given by:

$$H = 1-(1-\tfrac{a}{20})^L$$

The size of motif space is then corrected to be $B_L H$.

6 SLiMFinder Output Explained

One of the features of SLiMFinder is the wealth of information available in the various outputs. For an initial inspection of the results, the basic results file (slimfinder.csv or **resfile=FILE**) should be the first port of call and contains all the results for all datasets and their summary statistics. For further evaluation of specific results, however, the additional outputs (see **2.3.3**) can be extremely useful. These outputs are described in detail below and are saved in the directory specified by **resdir=PATH** (SLiMFinder/ by default).

6.1 Main SLiMFinder Output (slimfinder.csv)

The main output for SLiMFinder is a delimited file containing statistics on each dataset and any returned SLiMs. If no SLiMs are returned, a number of the columns will be blank but run- and dataset-specific information will still be output. The name of this file is set using the **resfile=FILE** option. If no file extension is given, ".csv" will be added. If the file extension is ".csv", the file will be comma delimited. If it is ".txt" then it will be space delimited. All other extensions will result in a tab delimited file. (".tdt" is recommended.)

6.1.1 Overwriting, appending and backing up results

By default, the main results file will be overwritten and a backup (optionally) saved as *.bak. If **append=T** then the file will be appended instead, allowing multiple runs to be examined together with ease. (Whatever this setting, multiple datasets within a single batch run will be output into a single file.) All dataset-specific files will be overwritten regardless of the append setting, so multiple runs should be redirected into different output directories using **resdir=PATH**. Previous results, namely the *.upc and *.*.pickle files can still be read in with the appropriate redirection by the **buildpath=PATH** option. (SLiMFinder will first look in the results directory and then in the build path. Both are set to SLiMFinder/ by default.)

6.1.2 Main Output Fields

The main output file consists of a number of dataset-specific and motif-specific fields. These are outlined in Table 6.1.

6.2 Sequence Relationships (*.upc, *.self.blast, *.dis.txt, *.phydis.txt)

Sequence relationships are very important for defining which motifs have sufficient support and for correctly adjusting the occurrence probabilities.

6.2.1 UPC Definitions

UPC definitions are made using an all-by-all BLAST, the results of which may be found in the *.self.blast file. UPCs are then saved in the *.upc file, which is a simple text file in the form:

```
#LIG_14-3-3_1# 4 Seq; 3 UPC; 3.897 MST
UP      N       MST     Seqs
1       2       1.897   RAF1_HUMAN__P04049 M3K5_HUMAN__Q99683
2       1       1.000   BAD_RAT__O35147
3       1       1.000   MPIP3_HUMAN__P30307
```

The first row contains the dataset name, the number of sequences, the number of UPC and the MST corrected size for the whole dataset. The closer the MST value is to 1, the more related the proteins are. A totally unrelated dataset will have an MST value equal to the number of sequences. The rest of the file is a simple table of the UPCs themselves: UP = UPC identifier; N = number of sequences in UPC; MST = corrected size of UPC; Seqs = List of sequences in that UPC.

This file can be manually edited to modify the way that SLiMFinder uses the dataset. (If present, this file will be read in by SLiMFinder rather than regenerated, unless **force=T**.)

Table 6.1. Fields for main SLiMFinder Output.

Field	Type†	Description	Help‡
Dataset	Dataset	Dataset name. Generally input filename without its file extension. This will be the first part of any dataset-specific file names in the output directory.	2.2
RunID	Run	Run identifier set by **runid=X**. The date & time is used if no ID is given. This allows the results of several runs to be compiled in a single results file and easily distinguished.	
Masking	Run	Summary of masking options: 'Dis' = disorder [**dismask=T**]; 'Comp' = complexity [**compask=X,Y**]; 'FT' = UniProt features [**ftmask=LIST**]; 'Inc' = inclusive features [**imask=LIST**]; 'Freq' = Mask AA frequencies [**maskfreq=T**]; 'None' = None. *cntd over page…*	4.1
Build	Run	SLiMBuild settings. Also used for pickle naming. lXwXoXaX, where lX = SLiM length [**slimlen=X**]; wX = max. wildcard [**maxwild=X**]; oX = adjusted ambiguous occurrence [**ambocc=X**] value; aX = which ambiguity is used: 0=None, 1=**equiv=LIST**, 2=**wildvar=T**, 3=both, 4=both+**combamb=T**.	2.5.1
RunTime	Dataset	The time taken for the dataset to run (HH:MM:SS).	
SeqNum	Dataset	Number of sequences in dataset.	
UPNum	Dataset	Number of UPC in dataset.	3.2
AANum	Dataset	Total number of unmasked AA in dataset.	4.1
MotNum	Dataset	Number of motifs with minimum support requirement (*i.e.* would be output if no probability cut-off.	4
Rank	Motif	Rank of returned SLiM. If a **slimcheck=LIST** motif, this will have a value of "*". If no motifs of any kind are returned, the dataset will have a rank of 0 and no other Motif fields will have values.	
Pattern	Motif	Pattern of returned SLiM.	3.3.1
IC	Motif	Information content.	3.3.3
Occ	Motif	Total number of occurrences across all sequences.	3.3.4
Support	Motif	Total number of sequences containing motif.	3.3.4
UP	Motif	Total number of unrelated proteins containing motif.	3.2
ExpUP	Motif	Expected number of unrelated proteins containing motif.	5.2
Prob	Motif	The uncorrected probability of the motif. (The probability of *k+* observations of a pre-defined motif.)	5.2
Sig	Motif	The corrected *p*-value of the motif	5.2
Cloud	Motif	Identifier of Motif Cloud to which the SLiM belongs. (Numbered starting at 1 for the most significant motif.)	6.4
CloudSeq	Motif	Number of sequences covered by that motif cloud.	6.4
CloudUP	Motif	Number of unrelated protein clusters covered by motif cloud.	6.4

†Field content either pertains to the specific Motif returned, the Dataset searched, or the Run settings that yielded those particular results.
‡Section of manual containing more information.

6.2.2 Distance Matrices

In addition to this file, two distance matrices are output: a plain tab delimited file *.dis.tdt and a **PHYLIP** (Felsenstein 2005) format *.phydis.txt file. These files contain the pairwise GABLAM sequence identities. (For the PHYLIP file, sequence names may be replaced by the number of the sequence in the input file.)

6.3 Dataset rank files (*.rank)

The *.rank file is similar to the output from SLiMDisc. This consists of a self-explanatory file header and a number of tab delimited fields from the main results output (See above).

6.4 Motif Clouds (*.cloud.txt)

Where significant motifs are returned, SLiMFinder will group them into Motif "Clouds" that consist of physically overlapping motifs (2+ non-wildcard positions are the same in the same sequence). This provides an easy indication of which motifs may actually be variants of a larger SLiM and should therefore be considered together.

The *.cloud.txt file contains information about the Significant Motif Clouds. The top of this file contains a self-explanatory header listing numbers of sequences, UPCs, SLiMs and clouds as well as motif cloud summaries listing the SLiMs making each cloud and the sequences containing one or more SLiMs in that cloud.

The rest of the file contains matrices listing the proportion of the entire dataset and of each other cloud contained by each motif cloud. Each row is a cloud, identified by its ID number and most significant SLiM in the first column. The second column is proportion of the sequences or UPCs in the whole dataset that contains one or more SLiMs in that cloud. Subsequent columns contain the same calculation but for the other clouds rather than the entire dataset.

These are not necessarily reciprocal. *E.g.*

```
Cloud                       Dataset      1        2
1:[DE]D[DE]F..F             0.88         1.00     0.75
2:Q.KR..Q.{0,1}Q          0.50           0.43     1.00
```

In this case 43% of the sequences in cloud 1 are also in cloud 2, while 75% of the sequences in cloud 2 are also in cloud 1. (This is because the clouds are different sizes.)

In addition to these matrices, tables are given with the statistical significance of any observed (lack of) overlap between clouds. The first is the probability of seeing that much overlap or more, given the proportion of the total dataset covered by each cloud. The second gives the probability of seeing that little overlap or less.

6.5 Motif Occurrence Tables (*.occ.csv, *.dat.rank & *.out)

In addition to the overall SLiM tables, statistics for the individual motif occurrences are also produced. The *.dat.rank (SLiMDisc emulator) and *.out (TEIRESIAS emulator) files simply contain a list of the proteins and then the protein and position for each motif occurrence in a simple one-line-per-motif format. The *.occ.csv file contains more detailed data on each occurrence in a comma separated file. These field headings are mostly reasonably obvious but see the PRESTO and SLiMPickings manuals for more details.

6.6 Sequence files (*.motifaln.fas, *.maskaln.fas, *.mapping.fas, *.masked.fas, *.motifs)

Assuming any motifs are returned, SLiMFinder outputs a number of sequence files to aid exploration of the results:

*.motifaln.fas = Alignment of all occurrences for each returned SLiM, with the surrounding sequence context (set using **`flanksize=X`** [default 30])

*.maskaln.fas = Same as above but showing masking of residues

*.mapping.fas = Fasta file containing sequences for each input sequence. These sequences are present in threes for each input sequence:
1. The significant motifs found in that sequence, aligned to (2) and (3)
2. The full-length unmasked sequence
3. The full-length masked sequence
4. In addition, if alignments are used (7.3), each protein will have its homologues aligned.

*.masked.fas = The input dataset, masked.

*.motifs = A file containing all significant motifs, plus any motifs given with the **slimcheck=LIST** option. This file is in the correct format for PRESTO or CompariMotif, or to be used as a **slimcheck=LIST** or **motifseq=LIST** input for future runs.

6.7 CompariMotif Comparisons (*.compare.tdt)

All significant motifs, plus any motifs given with the **slimcheck=LIST** option, are compared with each other using **CompariMotif** (Edwards et al. 2008) and the results output to *.compare.tdt. This allows the user to check for previously known motifs in their results. See the CompariMotif manual and/or website for more details.

6.8 XGMML Cytoscape Files (*.xgmml, *.cloud.xgmml)

For further visualisation, SLiMFinder outputs two XGMML format files that can be opened with **Cytoscape** (Shannon et al. 2003). Cytoscape is a free network visualisation tool. SLiMFinder outputs the input proteins and returned SLiMs as nodes of the network. UPC relationships, CompariMotif matches and occurrences of SLiMs in proteins are all represented as different edges (Figure 6.1). The file can be uploaded into Cytoscape using the File -> Import -> Network (Multiple File Types) command (CTRL+L). When first loaded, nodes will be displayed in a simple, uninformative, grid. Use one of the Cytoscape Layouts (e.g. Layout -> yFiles -> Organic) to make it clearer. Node (motif/protein) and edge (match/upc/occurence) can be viewed for selected nodes/edges using the Cytoscape Data Panel (Figure 6.2). See the Cytoscape documentation for details.

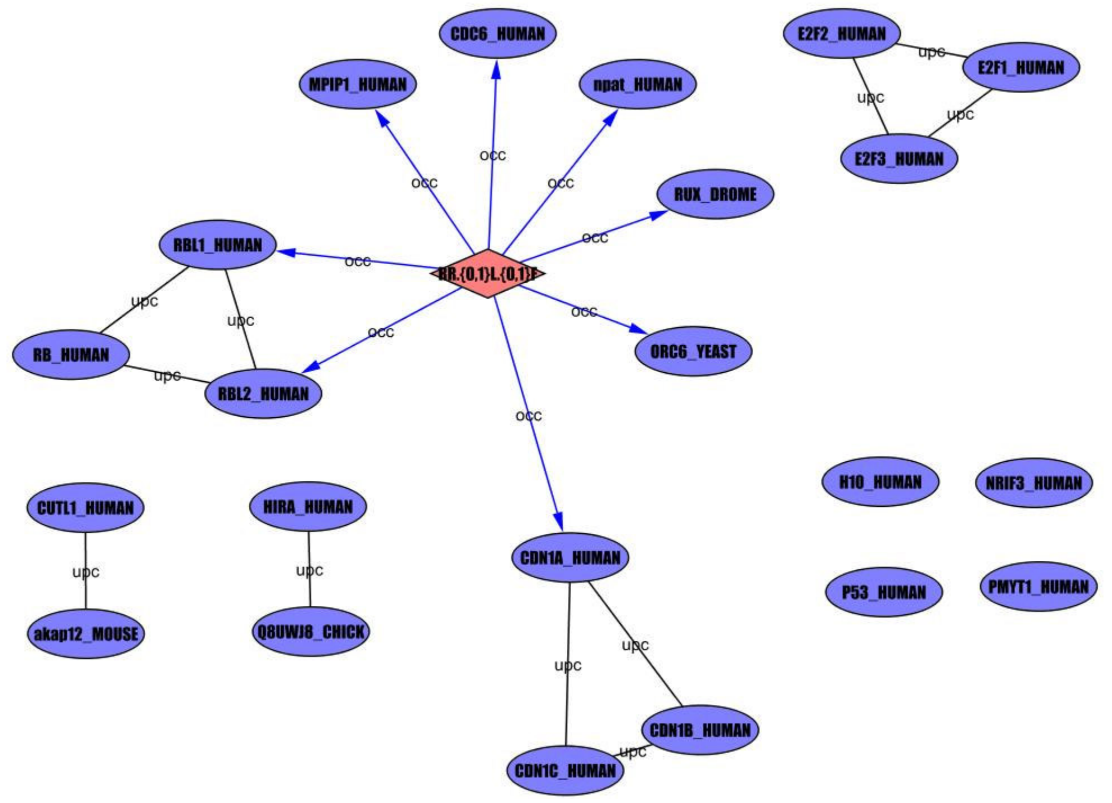

Figure 6.1. Cytoscape visualisation of SLiMFinder XGMML output for LIG_CYCLIN_1. Proteins are blue ellipses and SLiMs are red diamonds. Black lines indicate UPC relationships between proteins. Blue arrows indicate occurrences of motifs in proteins.

(a) (b)

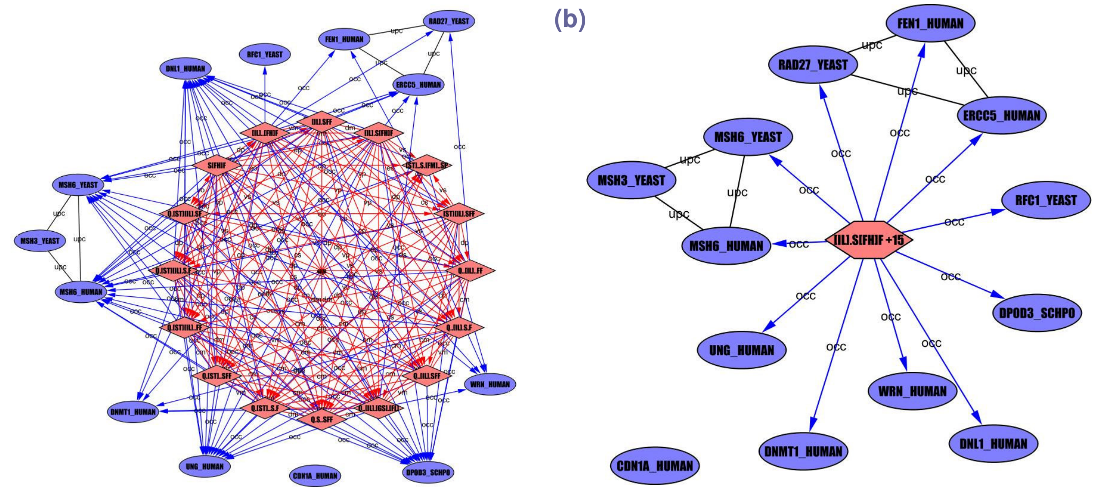

(c)

ID	AccNum	Dataset	Description	MaskLen	Protein	Species	UnmaskLen
UNG HUMAN	P13051	LIG_PCNA	Uracil-DNA glycosylase (EC 3.2.2.-) (UDG).	206.0	UNG_HUMAN__P13051	Homo sapiens (Human).	313.0
WRN HUMAN	Q14191	LIG_PCNA	Werner syndrome helicase.	671.0	WRN_HUMAN__Q14191	Homo sapiens (Human).	1432.0
MSH6 YEAST	Q03834	LIG_PCNA	MUTS protein homolog 6.	605.0	MSH6_YEAST__Q03834	Saccharomyces cerevisiae (Baker's yeast).	1242.0
RAD27 YEAST	P26793	LIG_PCNA	Structure-specific endonuclease RAD27.	212.0	RAD27_YEAST__P26793	Saccharomyces cerevisiae (Baker's yeast).	382.0
DPOD3 SCHPO	P30261	LIG_PCNA	DNA polymerase delta subunit 3 (Cell divisio...	266.0	DPOD3_SCHPO__P30261	Schizosaccharomyces pombe (Fission yeast).	372.0

(d)

ID	Dataset	Rank	IC	FixLength	PosLength	MinLength	MaxLength	Pattern
Q.[ST][IL]..FF	LIG_PCNA	5.0	4.537	3.0	5.0	8.0	8.0	Q.[ST][IL]..FF
Q..[IL]..FF	LIG_PCNA	4.0	3.769	3.0	4.0	8.0	8.0	Q..[IL]..FF
Q..[IL].SFF	LIG_PCNA	3.0	4.769	4.0	5.0	8.0	8.0	Q..[IL].SFF
Q.[ST]..SFF	LIG_PCNA	2.0	4.769	4.0	5.0	8.0	8.0	Q.[ST]..SFF
[IL].S[FH]F	LIG_PCNA	1.0	3.537	2.0	4.0	5.0	5.0	[IL].S[FH]F

(e)

ID	TYPE	Start_Pos	Variant	Match	MatchPos	NormIC	Score
[IL].S[FH]F (ep) S[FH]F	ep				3.0	1.0	3.0
[IL]..[FH]F (dp) S[FH]F	dp				2.0	0.697	1.394
MSH6 YEAST (occ) [IL].S[FH]F	occ	30.0	[IL].S[FH]F	LLSFF			
MSH6 YEAST (occ) [IL].SFF	occ	30.0	[IL].SFF	LLSFF			
MSH6 YEAST (occ) [IL]..[FH]F	occ	30.0	[IL]..[FH]F	LLSFF			
MSH6 HUMAN (upc) MSH6 YEAST	upc						

Figure 6.2. Cytoscape visualisation of XGMML output for LIG_PCNA results.
(a) Standard XGMML can get cluttered if lots of overlapping motif variants are produced. **(b)** The cloud XGMML simplifies this output by collapsing SLiMs into their clouds. Details for **(c)** Proteins, **(d)** Motifs, and **(e)** different kinds of edges can all be accessed using the Cytoscape data panel.

NB. One current limitation of the XGMML file is that only one occurrence for each Motif-Sequence pair is displayed, regardless of how many occurrences there actually are.

Sometimes, such as the results using proteins annotated with LIG_PCNA ELMs, the standard XGMML can become very cluttered because of all the similar, overlapping motifs that are returned (Figure 6.2(a)). Just as the motif cloud output (6.4) can simplify interpretation of raw results, so the cloud XGMML output (Figure 6.2(c)) can clarify visualisation in such cases. Note that occurrences are compressed to each cloud and so individual occurrence information is no longer available. Cytoscape allows multiple networks to be displayed simultaneously, however, and so occurrence details can be obtained through cross-referencing the standard XGMML file.

7 SLiM Statistics and Filtering

This part of the manual is incomplete. Please feel free to experiment with the filtering options, many of which are shared with PRESTO, SLiMPickings and SLiMSearch. (These manuals may have more information.) These options are under development and should therefore be used with an element of caution. The rje_slimcalc.py module contains the fullest list of command-line options but see also rje_slimlist.py.

7.1 Additional SLiM Calculations (slimcalc)

The additional SLiM calculations implemented by SLiMFinder are controlled with the **slimcalc=LIST** option, where **LIST** is one or more of SA, Hyd, IUP, Fold, Comp and Cons. In each case, an additional column will be added to the *.occ.csv output (6.5) with the relevant calculation for each SLiM occurrence. In addition, a **STAT_mean** column will be added to the main **resfile=FILE** output (2.3.1 & 6.1), containing the mean of the relevant stat across all occurrences of the SLiM. Percentiles can also be returned in steps defined using the **percentile=X** option, giving addition **STAT_pcX** columns. *E.g.* *percentile=25* will return the 0th, 25th, 50th, 75th and 100th percentile in columns *_pc0, *_pc25, *_pc50, *_pc75, *_pc100. This can be useful, for example, for identifying SLiMs for which at least 50% of occurrences meet a given criteria.

7.1.1 Surface Accessibility [SA]

This is calculated using a very crude SA estimate based on Janin & Wodak (Janin & Wodak 1978) over a 7 aa window. Each amino acid gets a SA value based on it and the 3 amino acids either side. These values are then averaged over the length of the SLiM.

7.1.2 Hydropathy [Hyd]

This is calculated using the Eisenberg scale (Eisenberg et al. 1984) over an 11 aa window, centred on each amino acid. The mean is then taken across the SLiM.

7.1.3 Disorder [IUP & Fold]

The same disorder methods used for filtering are used to calculate the mean disorder across the SLiM/window (see 4.1.1.). Each amino acid gets its own disorder score, ranging from 0 (ordered) to 1 (disordered), which is then averaged over the length of the SLiM.

7.1.4 Complexity [Comp]

The complexity measure calculated by slimcalc is very crude. It is simply the number of different amino acids observed across the length of the SLiM occurrence, divided by the maximum possible number, which is the length of the motif or twenty, whichever is smaller. E.g. a PxxPx[KR] motif occurrence with a sequence PASPPR would have a complexity of 4/6 = 0.6667.

7.1.5 SLiM Conservation [Cons]

Calculating the conservation of a SLiM occurrence is a complicated business with no clear best methodology. The relatedness of the proteins in the alignment is obviously expected to impact on any calculation, as is the general conservation of the protein as a whole. In addition, there are different ways to deal with ambiguity in motif definitions and/or amino acid substitutions that change only part of the SLiM. SLiMFinder implements a number of SLiM conservation strategies and parameters, which are covered in more detail in 7.2. In each case, however, a Cons value will be produced that ranges from 0.0 (not at all conserved) to 1.0 (completely conserved). In addition, the number of homologues present in the alignment used for the calculation (HomNum), the mean global percentage identity of these homologues (GlobID) and the mean percentage identity across the motif only (LocID) will be outputted. If the protein had no homologues, HomNum will be 0.0 but the conservation will be given as an arbitrary 1.0.

7.1.6 Extending Calculations to flanking regions

For the returned occurrence statistics, a window comprising of the motif + **winsize=X** is used. If winsize < 0 then *only* the flanks are used and not the motif itself. This does not apply to SLiM conservation scores or to the Local percentage identity returned.

7.2 SLiM Conservation Calculations

An important function of SLiMFinder is the ability to calculate conservation statistics for each match, provided alignment files are provided. (see 7.3). If alignments do not exist, **GOPHER** (Edwards 2006) can be used to generate them (see 7.3.1). If the identified file is not actually aligned, then RJE_SEQ will try to align the proteins using MUSCLE (Edgar 2004) or ClustalW (Higgins & Sharp 1988).

For each sequence, these alignments are used to generate the global percentage identity statistic:

- **GlobID** = Mean global percentage identity between query protein and homologues. This is calculated direct from the alignments, excluding matches of Xs, and is the percentage of query residues that match the aligned residue in the homologue. (Note that this is an asymmetrical measurement and the percentage of the homologue that aligns with the query may be very different if the sequences are of different lengths.)

Other conservation statistics are calculated individually for each occurrence of the motif. These are based on the homologous protein sequences available *at that site*. Any homologues with masked (X) residues that coincide to non-wildcard positions of the motif occurrence will be ignored from conservation calculations. Gaps, however, shall be treated as divergence unless the **alngap=F** option is used, in which case 100% gapped regions of homologues are also ignored (see 7.2.9). These additional statistics are:

- **Cons** = This is the conservation score across available homologues for that occurrence
- **HomNum** = Number of available homologues for that occurrence
- **LocID** = Mean local percentage identity between query protein and available homologues across region of match

Currently, there are four main Conservation scores implemented, which can be selected with the **conscore=X** option:

7.2.1 Absolute Conservation [abs]

For absolute conservation (**conscore=abs**), SLiMFinder first identifies the regions of the alignment that correspond to matches in the Query protein. Each aligned sequence is then taken in turn and the relevant region extracted, de-gapped, and compared to the original regular expression, *i.e.* the *degenerate* motif. The conservation score is then the proportion of these homologues in which the degenerate motif is conserved (Figure 7.1). (To calculate conservation of the specific occurrence of the motif, use the **consamb=F** option.)

7.2.2 Positional Scoring [pos]

Positional scoring (**conscore=pos**) uses a graded scoring system, where each sequence gets a score between 0 (no positions conserved) and 1 (all positions conserved). Each matching amino acid contributes a score of 1.0 (if **consinfo=F** (see 7.2.7)) and the sum over all positions is divided by the number of positions. For a degenerate site (when the default **consamb=T** option is used), the sequence must match *any* possible amino acid at that site.

The scoring matrix used for this scoring can be altered using the **posmatrix=FILE** command, where FILE contains either lists of equivalent amino acids on each line (e.g. FYW would mean that any of F, Y or W would score 1.0 vs. any other of F, Y or W), or an all-by-all matrix of amino acids and their conservation scores, e.g. this might give F-F a score of 1.0 and F-Y a score of 0.5. This allows the method to be customised according to user-determined rules. In this case, the best score between the sequence and any variant of a degenerate position is used (unless **consamb=F**).

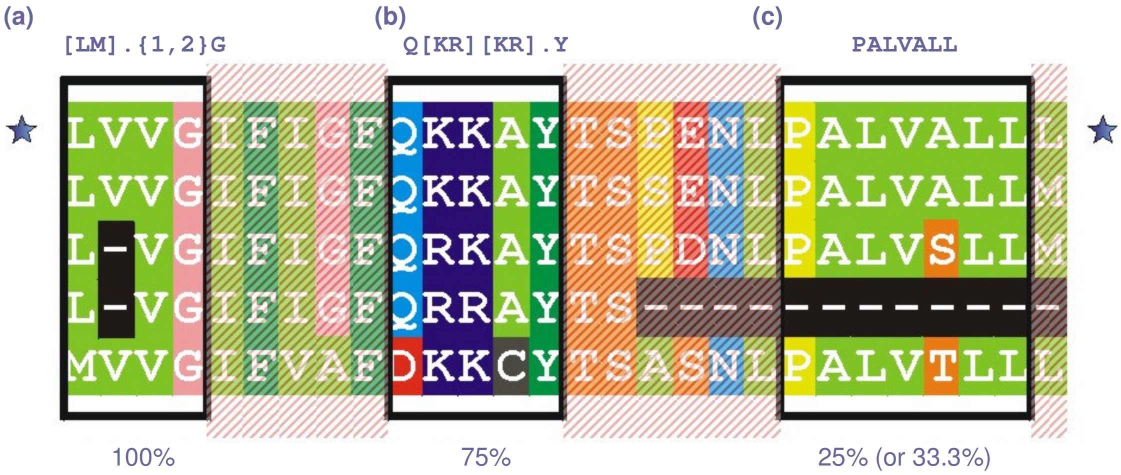

Figure 7.1. Absolute Conservation.
Three motifs are found in the query protein (marked with blue stars). This protein is ignored for conservation statistics. The black boxes represent the region of the alignment considered for each match. These matches in the homologues are then compared to the original regular expression. **(a)** Motif [LM]X{1,2}G is 100% conserved because, once gaps are removed, all four homologous sequences match the degenerate motif. **(b)** For motif Q[KR][KR]XY, one sequence does not match the degenerate motif and the query is excluded from the calculation, giving a conservation score of ¾ = 75%. **(c)** Only one of the homologues matches the motif. By default, all four sequences are considered, giving a conservation score of ¼ = 25%. If the `alngap=F` option is used, the 100% gapped sequence is ignored and the conservation is therefore 1/3 = 33.3%

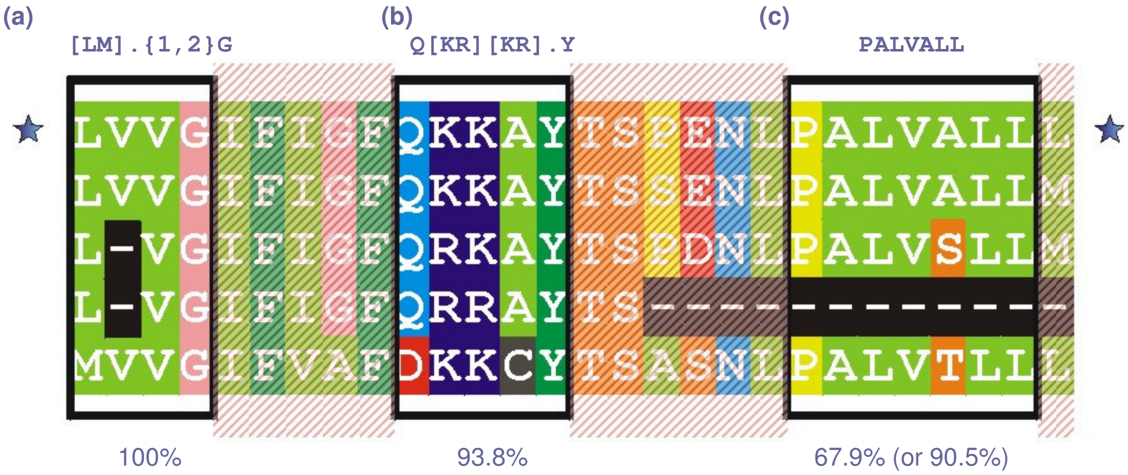

Figure 7.2. Position-speciffc Conservation.
The same motifs and alignments from Figure 7.1 are shown but this time position-specific conservation has been calculated. For simplicity, position-specific information content weighting has been switched off (consinfo=F) and so each position is weighted equally. **(a)** All positions in all orthologues still match, giving 100%. **(b)** Only one sequence does not match but now only one of the positions is a mismatch (D for Q) and so that sequence still gets an individual score of 75%, giving a total conservation of 93.8%. **(c)** Whereas the absolute conservation score penalises the two mismatches heavily, the position-specific score gives each of these sequences an individual conservation of 6/7 = 85.7%, for a total conservation score of (2x6 + 0 + 7 / 28 =) 67.9% (or 90.5% if the `alngap=F` option is used.)

7.2.3 AA Property Scoring [prop]

This (**conscore=prop**) is really just a specific example of the **posmatrix=FILE** command, where an amino acid property matrix (**aaprop=FILE**) is converted into a similarity matrix ranging from 0.0 to 1.0. By default, the property matrix of Livingstone and Barton is used (aaprop.txt) (Livingstone & Barton 1993). See the PEAT Appendices for more information on this matrix.

7.2.4 Relative Conservation Scoring [rel]

The same calculation as used for conservation masking (4.1.6) can also be used to calculate a conservation score for the motif. This is exception to the 0 < Cons < 1 rule. Relative conservation is centred around 0.0, with a standard deviation of 1.0, so all positive scores are good and all negative scores are bad.

7.2.5 Combined Scoring [all]

Under this scoring (**conscore=all**), the Cons output is the **mean** of the abs, pos and prop methods. In addition, statistics are generated for each of the individual scores:

- **Pos_Cons** = Positional conservation score across homologues.
- **Abs_Cons** = Absolute conservation score across homologues.
- **Prop_Cons** = Property-based conservation score across homologues.
- **Rel_Cons** = Relative conservation score across homologues.

The same additional options are applied to all methods, with the exception that Positional Weighting by Information Content has no effect on the absolute conservation method.

7.2.6 Motif ambiguity

By default, conservation will use the full degeneracy of the input motif. If **consamb=F** is used, the particular matching variant will be used instead. *E.g.* in Figure 7.1(b), the conservation of QKXXY would be calculated, rather than Q[KR]XXY.

7.2.7 Positional Weighting by Information Content

The **consinfo=T** option (the default) weights the contribution of different positions of the motif proportionally to their information content (IC). The IC of a position ranges from 0 for a wildcard position to 1 for a fixed position (see 3.3.3). For a fully fixed motif, all positions will have equal weighting. Otherwise, ambiguous positions make a smaller contribution to the score, which is normalised such that a sequence that is conserved at every position of the motif gets a score of 1.0. If **consinfo=F**, all positions contribute equally. If the “Absolute” motif conservation score is used, this weighting has no affect.

7.2.8 Homology Weighting

The **consweight=X** option controls how the conservation scores are weighted according the similarity of the homologues to the query. For each sequence s, the weighting W_s is calculated using the global percentage identity of the query versus that sequence, I_s, raised to the power of the **consweight=X** option, ω:

$$W_s = I_s^{\omega} / \Sigma W_s$$

When ω=0 (the default), W_s = 1 and all sequences are treated equally.

For ω=1, $W_s = I_s$, which up-weights the contribution of sequences closely related to the query. This means that the comparison of conservation scores will tend to penalise divergence in closely related sequences and will not be so heavily influenced by incorrect orthology assignment of distantly-related sequences. This weighting can be increased further with $\omega > 1$.

For ω=-1, $W_s = 1/I_s$, which up-weights the contribution of sequences distantly related to the query. This means that the comparison of conservation scores will tend to promote conservation in distantly related sequences but may be influenced by incorrect orthology assignment, which tends to be more of a problem for distantly-related sequences. This weighting can be increased further with $\omega < -1$.

7.2.9 Gap Treatment

By default, motifs that match to 100% gaps in a homologue are assumed to be due to missing or truncated sequences (as in the case of a draft genome, for example,) and do not contribute toward the relevant calculation (see Figure 7.1(c) and Figure 7.2(c)). Note that the calculation used pretends that these homologues are not present at all, and does not count them as conserved. If **alngap=T**, however, such sequences will be treated as divergence away from a motif and reduce the score. Sequences that are entirely Xs across the motif are always ignored.

7.2.10 Taxonomic subgroupings

In addition to the general conservation statistics produced for the given alignments, conservation calculations can be restricted to one or more taxonomic groups. This is achieved using the **conspec=LIST** option, where **LIST** is a list of files containing the UniProt species codes for the relevant grouping. Wildcards are allowed. Conservation analysis is then limited to these species and additional columns produced in the output (see below).

e.g. If only interested in the model organisms Human, Mouse, Rat, Chicken and Xenopus, one could use the command *conspec=model.spec_code* (or *conspec=*.spec_code*), where model.spec_code contains the species codes:

```
HUMAN
MOUSE
RAT
CHICK
XENLA
```

This would then produce additional output columns MODEL_Cons, MODEL_HomNum, MODEL_GlobID and MODEL_LocID. Where multiple files were given, each file would have its own set of output columns.

NB. The name all is reserved as a special key. Do *not* use *conspec=all.spec_code*.

7.3 Protein Alignments for SLiMFinder

SLiMFinder is designed to be able to use the output of GOPHER (Edwards 2006) for alignments of orthologues. Alternative sources for theses alignments can be used, as long as the format is correct.

Alignments should be in FASTA format with descriptions on one line followed by one or more lines containing the sequence. All sequences should be of the same length. The first word in each description should be unique. *e.g.*

```
>Seq1 And its description
SEQUENCE-ONE-GOES-HERE
>Seq2
---GAPS--ARE--ALLOWED-
>Seq3
---BUT---ALL-SEQUENCES
>Seq4
MUST-BE-EQUAL--LENGTHS
```

The file should be named AccNum.X, where AccNum is the accession number of the relevant protein in the search database, and X is given by the command **alnext=X**. Files should be found in a directory identified with the **alndir=PATH** command. The function to look for and use these alignments can be switched on using the **usealn=T** option.

7.3.1 Using GOPHER to make orthologue alignments

The program **GOPHER** (Edwards 2006) is provided in the download and can be called as part of the SLiMFinder search using the **usegopher=T** option. GOPHER will generate its usual files in the directory specified by the **gopherdir=PATH** option. This will generate a subdirectory named ALN, which will be set as the **alndir=PATH** parameter, if not already set. If not already set as such, the **alnext=X** option will be set to orthaln.fas.

To use GOPHER to generate alignments for SLiM conservation, you will need:

1. BLAST, MUSCLE and CLUSTALW installed on your system.
2. A sequence database containing potential orthologues. This should be identified to SLiMFinder using the `orthdb=FILE` option.

Details of how GOPHER works can be found in the GOPHER documentation.

7.4 Filtering output using SLiM Calculations

Results can be filtered at two different levels using the same basic syntax: individual motif occurrences can be rejected based on occurrence statistics, or whole motifs can be rejected based on whole-motif or combined occurrence statistics. These are controlled by the `occfilter=LIST` and `slimfilter=LIST` commands, respectively. Any usual columns of output in either the *.occ.csv (occfilter) or main results file (slimfilter) can be used, including conservation scores and percentiles *etc*. The `LIST` is in the form "`stat1>a,stat2<b,stat3=c,stat4!=d`" etc. and should either be a comma delimited list given on the commandline, or contained in a separate file (named `LIST` e.g. *statcut=my_statcut_list.txt*). If not a file name, enclose in double quotes or the <> symbols will try to pipe input/output!

The alllowed operators are:

Operator	Description
>	Filtered if the stat exceeds the cut-off
>= or =>	Filtered if the stat equals or exceeds the cut-off
<	Filtered if the stat is lower than the cut-off
<= or =<	Filtered if the stat is lower than or equal to the cut-off
= or ==	Filtered if the stat is equal to the cut-off
!= or <>	Filtered if the stat is not equal to the cut-off

All these may be applied to any stat, included text fields. Stat names should match the column headers of the output (case-insensitive). If a stat is given that is not recognised, SLiMFinder will report an error but continue processing without that stat cut-off. Note that the occurrences/SLiMs that meet the given criteria are removed (filtered).

Warning! Applying > or < to strings (*i.e.* non-numerical attributes) should be used with caution, though Python does seem to process them consistently with alphabetical sorting.

7.5 SLiM calculation/filtering options

Table 7.1 gives a summary of the main SLiM calculation and filtering options. Please check module documentation for latest developments.

Option	Description	Default	Manual
slimcalc=LIST	List of additional attributes to calculate for occurrences - Cons,SA,Hyd,Fold,IUP,Chg,Comp	[]	7.1
winsize=X	Used to define flanking regions for calculations. If negative, will use flanks *only*	[0]	7.1.6
percentile=X	Percentile steps to return in addition to mean	[0]	7.1
usealn=T/F	Whether to search for and use alignemnts where present.	[False]	7.3
alnext=X	File extension of alignment files, AccNum.X (checked before Gopher used).	[False]	7.37.3.1
usegopher=T/F	Use GOPHER to generate missing orthologue alignments.	[False]	7.3.1
gopherdir=PATH	Path from which to call Gopher (and look for PATH/ALN/AccNum.orthaln.fas)	[./]	7.3.1
fullforce=T/F	Whether to force regeneration of alignments using GOPHER.	[False]	7.3.1
orthdb=FILE	File to use as source of orthologues for GOPHER.	[]	7.3.1
conscore=X	Type of conservation score used:<br>- abs = absolute conservation of motif using RegExp over matched region<br>- pos = positional conservation: each position treated independently<br>- prop = conservation of amino acid properties<br>- all = all three methods for comparison purposes	[pos]	7.2
conspec=LIST	List of species codes for conservation analysis. Can be name of file containing list.	[None]	7.2.10
consamb=T/F	Whether to calculate conservation allowing for degeneracy of motif (True) or of fixed variant (False)	[True]	7.2.6
consinfo=T/F	Weight positions by information content (does nothing for conscore=abs)	[True]	7.2.7
consweight=X	Weight given to global percentage identity for conservation, given more weight to closer sequences - 0 gives equal weighting to all. Negative values will upweight distant sequences.	[0]	7.2.87.2.9
alngap=T/F	Whether to count proteins in alignments that have 100% gaps over motif (True) or (False) ignore as putative sequence fragments. (NB. All X regions are ignored as sequence errors.)	[False]	7.2.8
posmatrix=FILE	Score matrix for amino acid combinations used in pos weighting. (conscore=pos builds from propmatrix)	[None]	7.2.27.2.3
aaprop=FILE	Amino Acid property matrix file.	[aaprop.txt]	7.2.3
slimfilter=LIST	List of stats to filter (remove matching) SLiMs on, consisting of X*Y:<br>- X is an output stat (the column header),<br>- * is an operator in the list >, >=, !=, =, >= ,<<br>- Y is a value that X must have, assessed using *.<br>!!! Remember to enclose in "quotes" for <> filtering !!!	[]	7.4
occfilter=LIST	Same as slimfilter but for individual occurrences.	[]	7.4

Table 7.1. SLiM calculation/filtering options.

8 Appendices

8.1 Troubleshooting & FAQ

There are currently no specific Troubleshooting issues arising with SLiMFinder. Please see general items in the PEAT Appendices document and contact me if you experience any problems not covered.

8.2 QuickStart Guide

Download and install python from www.python.org.

Unzip slimfinder.zip in chosen directory. (A slimfinder subdirectory will be created.)

Download and install BLAST (Altschul et al. 1990) from NCBI if you have not already: http://www.ncbi.nlm.nih.gov/blast/download.shtml.

Create a slimfinder.ini file in the slimfinder directory. This should contain any default parameter settings and, most importantly, the path to the BLAST programs in the form: `blastpath=X` (*e.g.* *`blastpath=c:/bioware/blast/`*). If running in Windows, this file should also contain the option *`win32=T`*.

(Open a command-line window and) enter the chosen run directory containing the input files.

Run SLiMFinder, giving the installation path,
E.g. *`python c:\\bioware\\slimfinder\\slimfinder.py`*.
Results will be output to slimfinder.csv and SLiMFinder/*.* with run details in the slimfinder.log log file.

By default, SLiMFinder will run on all *.dat and *.fas files in the directory. To specify a single file, use the **`seqin=FILE`** command. To give an alternate list of files, use **`batch=LIST`** (*e.g.* *`batch=datasets/*.fas`*)

See full details in this manual for explanation of outputs and command-line options.

8.3 References

Altschul SF, Gish W, Miller W, Myers EW & Lipman DJ (1990). "Basic local alignment search tool." *J Mol Biol* **215**(3): 403-10.

Davey NE, Shields DC & Edwards RJ (2006). "Slimdisc: Short, linear motif discovery, correcting for common evolutionary descent." *Nucleic Acids Res.* **34**(12): 3546-54.

Davey NE, Edwards RJ & Shields DC (2007). "The slimdisc server: Short, linear motif discovery in proteins." *Nucleic Acids Res* **35**(Web Server issue): W455-9.

Dosztanyi Z, Csizmok V, Tompa P & Simon I (2005). "Iupred: Web server for the prediction of intrinsically unstructured regions of proteins based on estimated energy content." *Bioinformatics*. **21**(16): 3433-4.

Edgar RC (2004). "Muscle: A multiple sequence alignment method with reduced time and space complexity." *BMC Bioinformatics* **5**(1): 113.

Edwards RJ. (2006). "Gopher: Generation of orthologous proteins using high-throughput evolutionary relationships." from http://bioinformatics.ucd.ie/shields/software/gopher/.

Edwards RJ. (2006). "Presto: Peptide regular expression search tool." from http://bioinformatics.ucd.ie/shields/software/presto/.

Edwards RJ. (2006). "Rje_tree: Phylogenetic tree module." from http://bioinformatics.ucd.ie/shields/software/rje_tree/index.html.

Edwards RJ. (2006). "Rje_seq: DNA/protein sequence module." from http://bioinformatics.ucd.ie/shields/software/rje_seq/.

Edwards RJ & Davey NE. (2006). "Gablam: Global alignment from blast local alignment modules." from http://bioinformatics.ucd.ie/shields/software/gablam/.

Edwards RJ. (2007). "Slimsearch: Short linear motif search tool." from http://bioinformatics.ucd.ie/shields/software/slimsearch/.

Edwards RJ, Davey NE & Shields DC (2007). "Slimfinder: A probabilistic method for identifying over-represented, convergently evolved, short linear motifs in proteins." *PLoS ONE* **2**(10): e967.

Edwards RJ, Davey NE & Shields DC (2008). "Comparimotif: Quick and easy comparisons of sequence motifs." *Bioinformatics* **24**(10): 1307-9.

Eisenberg D, Schwarz E, Komaromy M & Wall R (1984). "Analysis of membrane and surface protein sequences with the hydrophobic moment plot." *J Mol Biol* **179**(1): 125-42.

Felsenstein J (2005). "Phylip (phylogeny inference package) version 3.6." *Distributed by the author. Department of Genome Sciences, University of Washington, Seattle.*

Higgins DG & Sharp PM (1988). "Clustal: A package for performing multiple sequence alignment on a microcomputer." *Gene* **73**(1): 237-44.

Janin J & Wodak S (1978). "Conformation of amino acid side-chains in proteins." *J Mol Biol.* **125**(3): 357-86.

Livingstone CD & Barton GJ (1993). "Protein sequence alignments: A strategy for the hierarchical analysis of residue conservation." *Comput Appl Biosci* **9**(6): 745-56.

Neduva V, Linding R, Su-Angrand I, Stark A, de Masi F, Gibson TJ, Lewis J, Serrano L & Russell RB (2005). "Systematic discovery of new recognition peptides mediating protein interaction networks." *PLoS Biol.* **3**(12): e405.

Prilusky J, Felder CE, Zeev-Ben-Mordehai T, Rydberg EH, Man O, Beckmann JS, Silman I & Sussman JL (2005). "Foldindex: A simple tool to predict whether a given protein sequence is intrinsically unfolded." *Bioinformatics.* **21**(16): 3435-8.

Puntervoll P, Linding R, Gemund C, Chabanis-Davidson S, Mattingsdal M, Cameron S, Martin DM, Ausiello G, Brannetti B, Costantini A, Ferre F, Maselli V, Via A, Cesareni G, Diella F, Superti-Furga G, Wyrwicz L, Ramu C, McGuigan C, Gudavalli R, Letunic I, Bork P, Rychlewski L, Kuster B, Helmer-Citterich M, Hunter WN, Aasland R & Gibson TJ (2003). "Elm server: A new resource for investigating short functional sites in modular eukaryotic proteins." *Nucleic Acids Res* **31**(13): 3625-30.

Rigoutsos I & Floratos A (1998). "Combinatorial pattern discovery in biological sequences: The teiresias algorithm." *Bioinformatics* **14**(1): 55-67.

Shannon CE (1997). "The mathematical theory of communication. 1963." *MD. Comput.* **14**: 306-317.

Shannon P, Markiel A, Ozier O, Baliga NS, Wang JT, Ramage D, Amin N, Schwikowski B & Ideker T (2003). "Cytoscape: A software environment for integrated models of biomolecular interaction networks." *Genome Res* **13**(11): 2498-504.
