# Supplementary material for: Estimation and efficient computation of the true probability of recurrence of short linear protein sequence motifs in unrelated proteins
Source: BMC Bioinformatics. 2010 Jan 7;11:14. doi: 10.1186/1471-2105-11-14 (PMC2819990; doi:10.1186/1471-2105-11-14)
Supplement: Additional file 2 — Supplemental materials. This file contains all supplementary tables and figures. [file 1471-2105-11-14-S2.DOC]

**SUPPLEMENTAL DATA**

**ST1. Off target motifs discovered in the ELM dataset analysis.**

| **Dataset^a^** | **Sig'^b^** | **Sig^c^** | **Motif^d^** | **k (N)^r^** |
| --- | --- | --- | --- | --- |
| LIG_RB | 0 | 0.00 | KRK (1) | 9(25) |
| LIG_NRBOX | 0.01 | 0.04 | IK.E..D (1) | 3(8) |
| LIG_14-3-3_3 | 0.00 | 0.03 | PP.TP..R (1) | 3(7) |
| LIG_PCNA | 0.00 | 0.00 | KRR (13) | 9(19) |

(a) The ELM dataset used. (b) The *Sig'* score of the top ranking motif matching the known interaction motif. (c) The *Sig* score of the top ranking motif matching the known interaction motif. (d) The regular expression of the top ranking motif that matches the known ELM. Significant motifs (*p* <0.01) are shown in bold. (e) The number of proteins in the dataset containing the variant of the motif discovered and the number of proteins in the dataset (in brackets).

**ST2. Runtimes for statistical frameworks.**

| **Dataset**  **Size** | **Motif**  **Discovery** | **Sig**  **Runtime** | **Sigv**  **Runtime** | **Sig’**  **Runtime** | **Sig’v**  **Runtime** |
| --- | --- | --- | --- | --- | --- |
| **5** | **31** | **1.51 (4.9%)** | **2.36 (7.6%)** | **63.67 (205.4%)** | **520.63 (1679.4%)** |
| **10** | **106** | **6.59 (6.2%)** | **54.2 (51.1%)** | **124.07 (117.0%)** | **49242.43 (46455.1%)** |
| **15** | **280** | **22.11(7.8%)** | **1197.61 (427.7%)** | **204.33 (72.9%)** | **>cut-off** |
| **20** | **811** | **50.94 (6.2%)** | **70,995.17 (8754.0%)** | **275.50 (33.6%)** |  |
| **25** | **1143** | **120.3 (10.5%)** | **>cut-off** | **411.57 (36.0%)** |  |
| **30** | **1847** | **200.51(10.8%)** |  | **466.96 (25.2%)** |  |
| **60** | **4125** | **315.87 (7.6%)** |  | **876.75 (21.2%)** |  |

*Dataset size* defines the number of proteins contained in the datasets. *Motif discovery* indicates the time in seconds taken for the SLiMBuild motif discovery portion of the SLiMFinder algorithm. *Sig, Sig_v_, Sig’* and *Sig’_v_* *Runtime* show the time taken for the statistical frameworks described in this paper as well as this runtime as a percentage of the motif discovery step. All times are in seconds. SLiMFinder was run with default settings with the exception of the motif scoring schemes and a cut-off of 1 day (86,400 seconds) was placed on the calculations. Analyses where run performed on an Intel Core 2 duo 2Ghz processor with 1 Gb RAM.

aa = [A,C,D,E, …… Y]

n = len(aa)y

sub_motif = “”

motif_list = []

motif_length = 5

function recursive_motif_build(n,sub_motif,motif_list)

if len(sub_motif) < motif_length

for i in n:0

sub_motif = concatenate sub_motif and aa[i]

recursive_motif_build(i,sub_motif,motif_list)

else

add sub_motif to motif_list

return motif_list

**SF1. Pseudocode to recursively define all non-redundant motif groupings (see Appendix).**

**SF2.** Distribution of binomial *p*-values for the top ranking motifs of lengths 3, 4 and 5 for 100 datasets of 20 proteins.

**
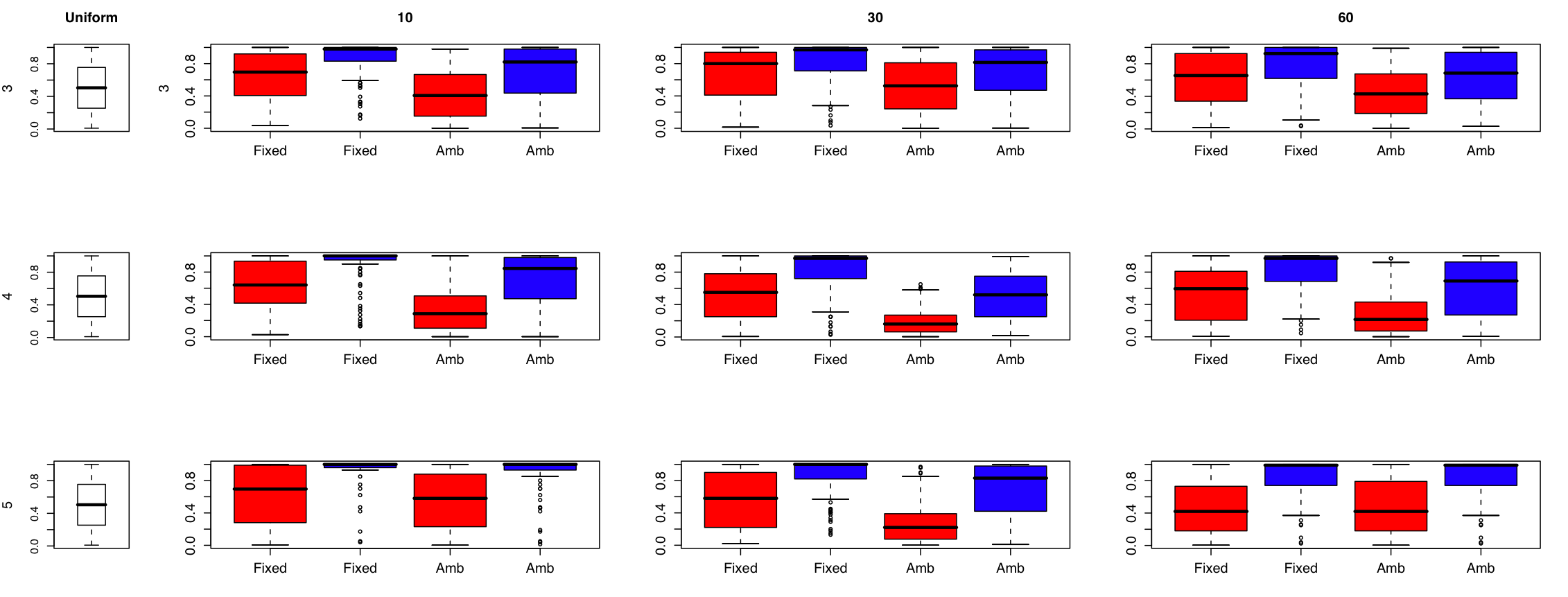
**

**SF3.** The comparison, for each dataset size and motif length, of the distribution of top ranking motifs significance values, for ambiguous and fixed motifs using Sig’_v_ (red) and Sig (blue) scoring schemes. Datasets are as described in “Construction of random datasets”
